# Genome sequencing of the sweetpotato whitefly *Bemisia tabaci* MED/Q

**DOI:** 10.1093/gigascience/gix018

**Published:** 2017-03-15

**Authors:** Wen Xie, Chunhai Chen, Zezhong Yang, Litao Guo, Xin Yang, Dan Wang, Ming Chen, Jinqun Huang, Yanan Wen, Yang Zeng, Yating Liu, Jixing Xia, Lixia Tian, Hongying Cui, Qingjun Wu, Shaoli Wang, Baoyun Xu, Xianchun Li, Xinqiu Tan, Murad Ghanim, Baoli Qiu, Huipeng Pan, Dong Chu, Helene Delatte, M. N. Maruthi, Feng Ge, Xueping Zhou, Xiaowei Wang, Fanghao Wan, Yuzhou Du, Chen Luo, Fengming Yan, Evan L. Preisser, Xiaoguo Jiao, Brad S. Coates, Jinyang Zhao, Qiang Gao, Jinquan Xia, Ye Yin, Yong Liu, Judith K. Brown, Xuguo “Joe” Zhou, Youjun Zhang

**Affiliations:** 1 Institute of Vegetables and Flowers, Chinese Academy of Agricultural Science, Beijing 100081, China; 2 BGI-Shenzhen, Shenzhen 518083, China; 3 Department of Entomology, S-225 Agricultural Science Center North, University of Kentucky, Lexington, KY 40546-0091, USA; 4 School of Plant Sciences, University of Arizona, Tucson, AZ 85721, USA; 5 Institute of Plant Protection, Hunan Academy of Agricultural Sciences, Changsha 410125, China; 6 Department of Entomology, Volcani Center, Bet Dagan 5025001, Israel; 7 Key Lab of Bio-pesticide Creation and Application, South China Agricultural University, Guangzhou 510642, China; 8 College of Agronomy and Plant Protection, Qingdao Agricultural University, Qingdao 266109, China; 9 Cirad, UMR PVBMT, Saint-Pierre, La Réunion, France; 10 Natural Resources Institute, University of Greenwich, Chatham Maritime, Kent ME4 4TB, UK; 11 Institute of Zoology, Chinese Academy of Sciences, Beijing 100101, China; 12 Institute of Plant Protection, Chinese Academy of Agricultural Sciences, Beijing 100193, China; 13 Ministry of Agriculture Key Laboratory of Agricultural Entomology, Institute of Insect Sciences, Zhejiang University, Hangzhou 310058, China; 14 School of Horticulture and Plant Protection and Institute of Applied Entomology, Yangzhou University, Yangzhou 225009, China; 15 Institute of Plant and Environment Protection, Beijing Academy of Agriculture and Forestry Sciences, Beijing 100089, China; 16 Collaborative Innovation Center of Henan Grain Crops, College of Plant Protection, Henan Agricultural University, Zhengzhou 450002, China; 17 Department of Biological Sciences, University of Rhode Island, Kingston, Rhode Island 02881, USA; 18 College of Life Sciences, Hubei University, Wuhan 430062, China; 19 United States Department of Agriculture, Agricultural Research Service, Corn Insects & Crop Genetics Research Unit, Ames, IA 50011, USA

**Keywords:** Whitefly *Bemisia tabaci*, Genomics, Assembly, Annotation

## Abstract

The sweetpotato whitefly *Bemisia tabaci* is a highly destructive agricultural and ornamental crop pest. It damages host plants through both phloem feeding and vectoring plant pathogens. Introductions of *B. tabaci* are difficult to quarantine and eradicate because of its high reproductive rates, broad host plant range, and insecticide resistance. A total of 791 Gb of raw DNA sequence from whole genome shotgun sequencing, and 13 BAC pooling libraries were generated by Illumina sequencing using different combinations of mate-pair and pair-end libraries. Assembly gave a final genome with a scaffold N50 of 437 kb, and a total length of 658 Mb. Annotation of repetitive elements and coding regions resulted in 265.0 Mb TEs (40.3%) and 20 786 protein-coding genes with putative gene family expansions, respectively. Phylogenetic analysis based on orthologs across 14 arthropod taxa suggested that MED/Q is clustered into a hemipteran clade containing *A. pisum* and is a sister lineage to a clade containing both *R. prolixus* and *N. lugens*. Genome completeness, as estimated using the CEGMA and Benchmarking Universal Single-Copy Orthologs pipelines, reached 96% and 79%. These MED/Q genomic resources lay a foundation for future ‘pan-genomic’ comparisons of invasive vs. noninvasive, invasive vs. invasive, and native vs. exotic *Bemisia*, which, in return, will open up new avenues of investigation into whitefly biology, evolution, and management.

## Introduction

### Samples and libraries construction

As a globally invasive species, the phloem-feeding whitefly *Bemisia tabaci* (Genn.; hereafter ‘*Bemisia*’) has been found on all continents except Antarctica [1,2]. Taxonomically, *B. tabaci* is considered a species complex that contains several morphologically indistinguishable but genetically distinct ‘cryptic species’ [2–7]. The *Bemisia* Middle East-Asia Minor 1 (MEAM1, or ‘B’) cryptic species is highly invasive and has emerged as a major pest in the United States, Caribbean Basin, Latin America, Middle East [1], and East Asia [8]. Similarly, the invasive *Bemisia* Mediterranean (MED, or ‘Q’) cryptic species has been introduced into several geographic locations and has become established throughout China [9,10]. Despite substantial research and the recently published whitefly *B. tabaci* MEAM1/B genome [11], however, the genetic or genomic basis of MED/Q remains obscure.

The MED/Q *B. tabaci* adult whitefly females (2n) and males (1n) were initially collected from infested field-grown cucumber plants in Beijing, China during 2011 and used to establish a laboratory colony (MED/Q) at the Institute of Vegetable and Flowers, Chinese Academy of Agriculture Science by transferring adult males and females to caged pepper plants (10–12 leaf stage). Results of mtCOI gene PCR-RFLP assays [12] and direct DNA sequencing followed by phylogenetic evaluation against reference sequences [13] both confirmed that the *Bemisia* in the MED/Q colony belonged to the Q1 haplotype group, or western Mediterranean region clade (data not shown).

The MED/Q whitefly colony was used as the source initial short shotgun Illumina sequencing. Adult whiteflies fed using Parafilm membrane sachets containing a 25% sucrose solution for 48 hours prior to collection of ∼5000 male and female adults (∼50:50). Samples were immediately frozen in liquid nitrogen for 3 hours prior to transfer to a −80°C freezer. This genomic DNA was used to construct Illumina TruSeq paired end (PE) sequencing libraries (170-, 250-, 300-, 500-, and 800-bp insert sizes) and mate pair (MP) libraries (2, 5, 10, 20, and 40 kb in size) according to the manufacturer's instructions. Additionally, two Illumina PE sequencing libraries (∼500-bp and 800-bp inserts) were constructed from whole genome amplification (WGA) reactions carried out on genomic DNA isolated from two adult male whiteflies. We also constructed 13 BAC libraries with pooling of clones and Illumina library construction according to the manufacturer's instructions.

### Genome sequencing and assembly

All libraries were sequenced on an Illumina Hiseq 2000 using 100-bp reads from both fragment ends, and raw data processed and assembled as shown (Supplemental Table S1; Supplemental Fig. S1). Briefly, a series of filtering steps was performed on the raw reads to filter out the following: (1) reads with >10% Ns, >40% low-quality bases, >10 bp overlapping with adapter sequences, allowing no more than 3-bp mismatches; (2) paired-end reads that overlapped >10 bp between two ends, with insert size >200-bp libraries; and (3) duplicated reads generated by PCR amplification during the construction of the large-insert library. Filtered reads were used for K-mer determination within subsequent assembly steps. The frequency of each K-mer was calculated from the genome-sequence reads. K-mer frequencies along the sequence depth gradient follow a Poisson distribution in a given data set except for a high proportion at low frequency due to sequencing errors, as K-mers that contain such sequencing errors may be orphans among all splitting K*-*mers. The genome size, *G*, was estimated as *G = K_*num/*K_*depth, where *K_*num is the total number of K-mers and *K_*depth is the maximal frequency. Initial contigs were assembled from filtered 500- and 800-bp insert-size WGA PE libraries using SOAPdenovo. The sequencing reads obtained for 2-k to 40-kb MP libraries were used to connect the contigs and to generate the scaffolds as described by Li et al. (2010) [14] with a K-mer size of 65.

Individual BAC pools were assembled independently using SOAPdenovo and the whole genome shotgun reads from PE and MP libraries were used to fill gaps in the BAC scaffolds. After sequencing, the raw reads were filtered as described above. In addition, reads representing contamination by *Escherichia coli* or the plasmid vector were filtered. The pooled reads were separated according to the BAC-reads index, and each BAC was assembled using a combination of “hierarchical assembly” and “*de Bruijn* graph assembly.” First, the reads linked to each BAC were assembled using SOAPdenovo [14], with various combinations of parameters with a K-mer range from 27 to 63 and a step size of 6. The assembly with the longest scaffold N50 was defined as the “best” for each BAC. The resulting BACs were mapped with the large shotgun MP read data to optimize the assembly for each BAC.

The final draft assembly was produced by integrating sequences that overlapped among the scaffolds independently assembled from genome shotgun and BAC reads, and in doing so eliminated the redundant scaffolds using the following steps. To integrate the two assemblies, the software *Rabbit* [15] was applied to identify any relationship between scaffolds, to connect the overlapping regions that shared at least 90% similarity, and to remove redundancy based on a 17-mer frequency. Finally, *SSPACE* [16] was used to construct super-scaffolds containing 800-bp to 40-kb whole genome sequence (WGS) reads, and the 170- to 800-bp genome shotgun read data were used to fill the gaps using *GapCloser* [14]. Postassembly processing included removal of contaminating bacterial and viral DNA sequences by aligning all assembled sequences to the genome sequences of viruses and bacteria, obtained from previous local BLASTn alignments and by NCBI upload filter. Aligned sequences that shared >90% identity and were >200 bp in size were filtered from the final assembly. The assembled sequences that were covered by at least one expressed sequence tag (EST) sequence were retained. Process read data were mapped to the draft MED/Q genome using *SOAPaligner* software and read counts were made from .bam files and the average depth was computed from all bases in the window. The relation graph of base pair percentages, and each given sequencing depth along the genome, was obtained.

Using genomic DNA from the MED/Q colony, a total of 20 WGS shotgun sequencing libraries was generated (18 pooled male and female PE and MP libraries, and two haploid male-derived WGA PE libraries), from which sequences were generated on an Illumina Hiseq2500 platform. Library sequencing produced a total of 428.2 Gb or an approximate 594.7-fold genome coverage assuming a 0.72-Gbp genome size (based on 17-mer analysis). For the 10 short-insert PE libraries, there were a total of 229.4 Gb (100-bp or 150-bp read length, approximately 318.6-fold genome coverage). Sequencing the eight large-insert (>1 kb) MP libraries produced 80.3 Gb of reads (49 bp read length, 111.5-fold coverage) for use in scaffold construction (Supplemental Table S1). The two male WGA libraries produced a total of 118.5 Gb of data (Supplemental Table S1) or approximately 164.6-fold genome coverage. Sequencing of 13 BAC pools generated 362.6 Gbp of raw data (288.4 Gbp processed data; results not shown). The subsequent assembly of this sequence data using our pipeline (Supplemental Fig. S1) generated a 658-Mbp draft genome assembly for MED/Q consistent with recent flow cytometry estimates [17]. The mean read depth across 10-kb windows indicated that all genome regions were highly represented within the read data, with <1.5% having a depth of <10× (remaining data not shown).

Through statistical comparison of genome assembly and annotation between MED/Q and MEAM1/B (Table 1), we found the draft genome of MED/Q consisted of a genome size of 658 Mb with contig N50 size 44 kb, while MEAM1/B assembly was 615 Mb with contig N50 of 30 kb. They have similar G+C content of about 39%, while higher TEs existed in MEAM1/B (44%) than MED/Q (40%). After combining several annotation methods, 20 748 genes were predicted in MED/Q, whereas 15 664 genes in MEAM1/B, and about 80% of both two gene sets were supported by several public functional databases.

**Table 1:** Statistics comparison of genome assembly and annotation between MED/Q and MEAM1/B

	MED/Q^a^		MEAM1/B^b^	
Sequencing summary	Scaffold^c^	Contig^c^	Scaffold^c^	Contig^c^
Total number	4954	29 618	19 761	52 036
Total length of (bp)	658 272 463	638 061 971	615 029 878	599 923 598
Gap number (bp)	19 828 575	0	14 380 491	0
Average length (bp)	132 877	21 543	31 123	11 529
N50 length (bp)	436 791	44 366	3 232 964	29 918
N90 length (bp)	111 835	11 504	381 346	6117
Maximum length (bp)	2 857 362	362 835	11 178 615	269 706
Minimum length (bp)	501	500	500	500
GC content (%)	39.46	39.46	39.64	39.64
TEs proportion (%)	265 Mb (0.40)		269 Mb (0.44)	
CEGMA evaluation (%)	96		100	
BUSCO evaluation	78		96.8	
Gene number	20 786		15 664	
Average gene length (bp)	10 065		22 762	
Average CDS length (bp)	1952		1470	
Average exon per gene	6		6	
Average exon length (bp)	351		234	
Average intron length (bp)	1776		3125	
Annotation gene (%)	79.97		81	
Assemble software	SOAPdenovo		Platanus	

a From this study.

b From the published MEAM1/B genome [11].

c Only contigs and scaffolds ≧500 bp were included in the genome assembly.

### Annotation of repetitive elements

Repetitive elements were searched for and identified using *Repbase* [18] implemented in *TRF* software [19], and a *de novo* approach implemented in *Piler* [20]. For the *Repbase*-based method, two software programs named *RepeatMasker* [21] and *RepeatProteinMask* were used to identify repetitive sequences. In the *de novo* approach, *Piler-DF-1.0* [20], *RepeatScout-1.0.5* [22], and *LTR-FINDER-1.0.5* [23] were used to build *de novo* repeat libraries from the genome sequences. Finally, the repeated sequences were searched for and classified using the *RepeatMasker* software. Homology-based annotation of MED/Q repetitive elements was queried against Repbase v.20.05 [18] with RepeatMasker [21]. We found a total of 265.0 Mb TEs, or 40.3% of the MED/Q genome size. This was about 10% higher than the repeat contents of *Acyrthosiphon pisum* and *Rhodnius prolixus*, but similar to that of *Nilaparvata lugens* (39.8%) (Supplemental Table S2). This suggests that long terminal repeat (LTR) (18.5%) are more abundant and contain more nucleotides than all other TE classes. This proliferation of LTR retrotransposons has been found in only one other Hemipteran genome, that of *N. lugens* (12.29%). The MED/Q genome also contains the high proportion of the DNA-transposon TEs (12.92%) found in other fully described Hemipteran genomes. As with both *N. lugens* (0.5%) and *R. prolixus* (0.01%), the MED/Q genome also appears devoid of short interspersed nuclear elements (0.96 %). These other Hemipteran genomes also contain a small amount of long interspersed nuclear elements (*A. pisum*: 2.6%; MED/Q: 3.18%; *R. prolixus*: 3.2%), but *N. lugens* (12.84%). This suggests that MED/Q-specific TEs, especially the LTRs, have evolved relatively recently and contribute to the large number of gene sets.

### Annotation of coding regions

Initial evaluation of the gene coverage rate in the draft MED/Q genome assembly was assessed by comparing against 248 core eukaryotic genes obtained using *CEGMA 2.4* [24] and Benchmarking Universal Single-Copy Orthologs (BUSCO) [25]. Additionally, 105 067 *B. tabaci* transcript sequences, ESTs, of >200 bp were used as BLASTn queries against the assembled genome to estimate the representation (cutoff *E*-value ≥ 10^−40^). Protein-coding gene *de novo* predictions using GENEWISE [26] and *ab initio* gene predictions using GENSCAN [27] and AUGUSTUS [28] were made in combination with 13.7 Gbp of transcriptome (RNA-Seq) data including published MED/Q *B. tabaci* body, guts, and salivary glands [29–31] and additional, previously unpublished data from females and males [32], to obtain consensus gene sets using GLEAN [33].

For homolog-based prediction, protein sequences from nine species (*A. pisum*, *A. mellifera*, *D. melanogaster*, *R. prolixus*, *Z. nevadensis*, *A. gambiae*, *B. mori*, *P. humanus*, and *T. castaneum*) were aligned with the MED/Q genome scaffolds using *TblastN* (E-value <1e-5). Target sequences were used to search for accurate gene structures implementing the *GeneWise* software [26]. For the RNA-Seq datasets, the transcriptome reads were first aligned against the genome using *TopHat* [33] to identify candidate exon regions. Then, the *Cufflinks* software [34] was used to assemble the aligned reads into transcripts, and the open reading frames were predicted to obtain reliable transcripts using a Hidden Markov Model-based training parameter. Finally, *GLEAN* [33] was used to integrate the predicted genes with the *de novo*, homologous, and RNAseq data to produce the final gene set. The functional annotation of genes was performed using *BLASTP* alignment to KEGG [35], SwissProt, and TrEMBL [36] databases. Motifs and domains were determined by *InterProScan* [37] and protein database searches against ProDom, PRINTS, Pfam, SMART, PANTHER, and PROSITE.

Preliminary evaluation of transcribed regions within the draft MED/Q genome assembly coverage found that ∼95.2% of *B. tabaci* ESTs > 200 bp were present, with 90 652 ESTs showing ≥90% length coverage on one scaffold (Supplemental Table S7). This alignment encompassed 92.9% of nucleotides within the EST dataset. Analogously, 229 (96%) of the 248 sequences in the CEGMA gene set and 79% complete and fragmented BUSCOs were present in the MED/Q genome assembly (remaining data not shown). The final GLEAN gene models predicted a reference gene set of 20 786 protein-coding genes, a consensus result derived from *de novo*, orthology, and evidence (RNA-seq)-based prediction methods (Supplemental Table S3) and integrated into GLEAN gene models (Supplemental Table S4). Among the GLEAN gene models, 16 622 (79.97%) received functional gene annotations using the various databases queried in our analysis pipeline (Supplemental Table S5).

### Prediction of gene orthology

Twelve insect species including *B. tabaci* (Genn.) (Gennadius, 1889) (Hemiptera: Aleyrodidae), *Acyrthosiphon pisum* (Harris, 1776) (Hemiptera: Aphididae), *Rhodnius prolixus* (Stal, 1859) (Hemiptera: Triatominae), *Nilaparvata lugens* (Stål, 1854) (Hemiptera: Delphacidae), *Pediculus humanus* (Linnaeus, 1758) (Phthiraptera: Pediculidae), *Apis mellifera* (Linnaeus, 1758) (Hymenoptera, Apidae), *Nasonia vitripennis* (Ashmead, 1904) (Hymenoptera, Pteromalidae), *Tribolium castaneum* (Herbst, 1797) (Coleoptera, Tenebrionidae), *Anopheles gambiae* (Giles, 1902) (Diptera, Culicidae), *Drosophila melanogaster* (Meigen, 1830) (Diptera, Drosophilidae), *Bombyx mori* (Linnaeus, 1758) (Lepidoptera, Bombycidae) and *Danaus plexippus* (Kluk, 1802) (Lepidoptera, Nymphalidae), and two divergent arthropods, *Daphnia pulex* (Müller, 1785) (O. Cladocera, Daphniidae) and *Tetranychus urticae* (C. L. Koch, 1836) (O. Arachnida, Tetranychidae), were used to predict orthologs and to reconstruct the phylogenetic tree. Gene families were identified using *TreeFam* [38,39], and single-copy gene families were assembled to reconstruct phylogenetic relationships. Coding sequences of each single-copy family were concatenated to form one super gene group for each species. All of the nucleotides at codon position 2 of these concatenated genes were extracted to construct the phylogenetic tree by *PhyML* [40], with a gamma distribution across sites and an HKY85 substitution model. The same set of sequences at codon position 2 was used to estimate divergence times among lineages. The fossil calibrations were set with two previous node data [41,42]. The *PAML* mcmctree program (v.4.5) [43,44] was used to compute split times using the approximate likelihood calculation algorithm. The software *Tracer* (v.1.5.0) was utilized to examine the extent of convergence for two independent runs.

**Figure 1:**
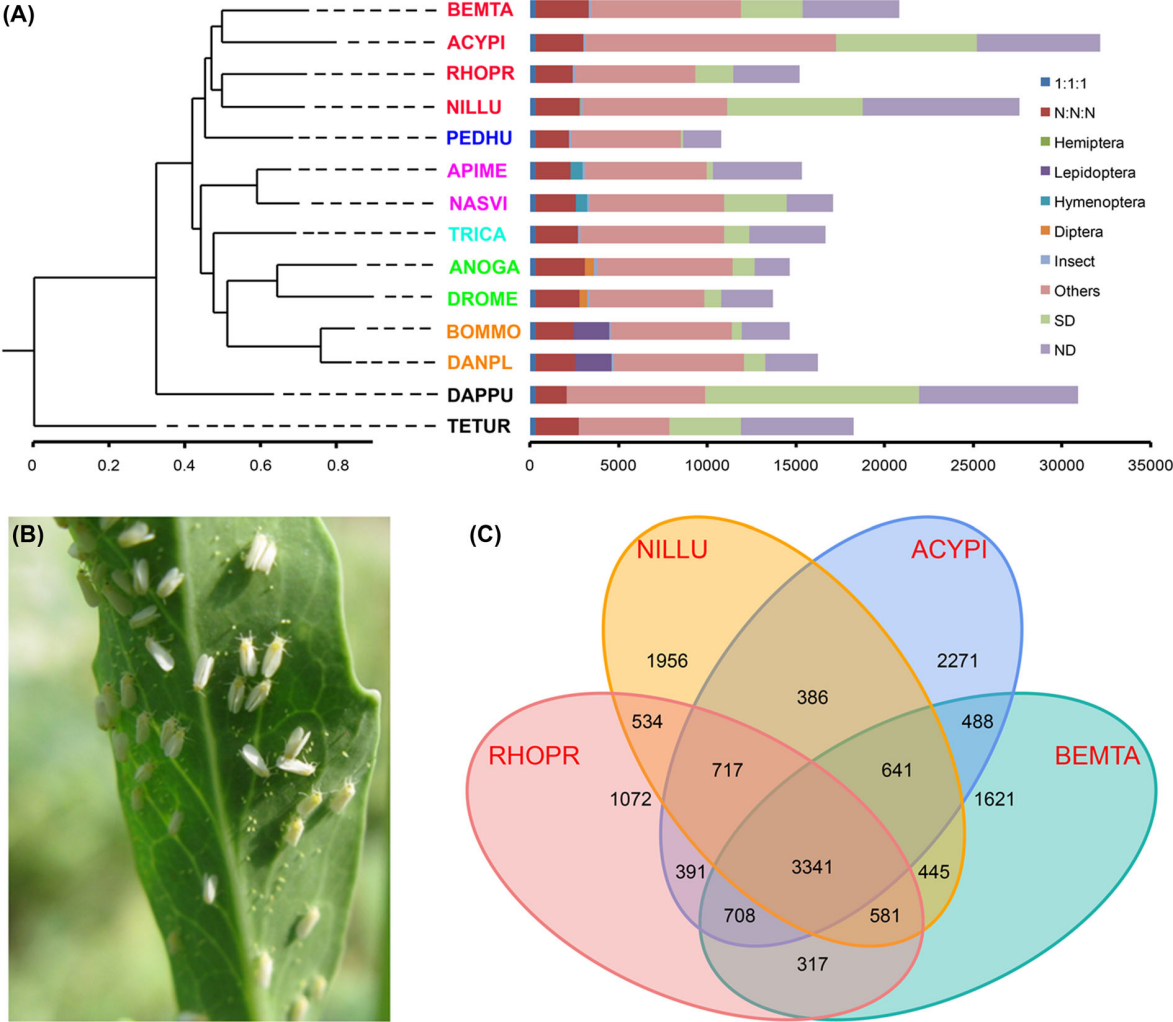
Phylogenetic relationships and genomic comparisons between *Bemisia tabaci* and other insect species (**A**) Phylogenetic relationships of *B. tabaci* (BEMTA) to insects and other arthropods based on single-copy orthologous genes present in their complete genomes. The following 12 insect species were used for this analysis: *Acyrthosiphon pisum* (ACYPI), *Anopheles gambiae* (ANOGA), *Apis mellifera* (APIME), BEMTA, *Bombyx mori* (BOMMO), *Danaus plexippus* (DANPL), *Drosophila melanogaster* (DROME), *Nasonia vitripennis* (NASVI), *Nilaparvata lugens* (NILLU), *Pediculus humanus* (PEDHU), *Rhodnius prolixus* (RHOPR), and *Tribolium castaneum* (TRICA). The two arthropods *Daphnia pulex* (DAPPU) and *Tetranychus urticae* (TETUR) were used as outgroup taxa. Branch lengths represent divergence times estimated for the second codon position of 308 single-copy genes, using *PhyML* with a gamma distribution across sites and a HKY85 substitution model. The branch supports were inferred based on the approximate likelihood ratio test (aLRT). Gene orthology was determined by comparing the genomes of these 14 arthropod species. The use of 1:1:1 refers to single-copy gene orthologs found across all 14 lineages. The use of N:N:N refers to multi-copy gene paralogs found across the 14 lineages. Diptera, Hemiptera, Hymenoptera, Lepidoptera, and Insecta refer to taxon-specific genes present only in the particular lineage. SD indicates species-specific duplicated genes, and ND indicates species-specific unclustered genes. (**B**) Image of adult MED/Q. (**C**) A Venn diagram showing the orthologous groups shared among the hemipteran genomes of *A. pisum*, *B. tabaci*, *N. lugens*, and *R. prolixus*. Our analysis found 3341 gene families common to all four hemipteran genomes, and 2921 common to the genomes of the six vascular (blood and phloem) feeders.

Phylogenetic analysis based on orthologs across 14 arthropod taxa (Supplemental Table S6) suggested that MED/Q is clustered into a hemipteran clade containing *A. pisum* and is a sister lineage to a clade containing both *R. prolixus* and *N. lugens* (Fig. 1A). The range of species-specific genes within the four hemipteran genomes ranged from 38% to 60%, with higher values for the three phloem-feeding specialists. This led us to investigate interspecific changes in the number and diversity of gene family members (orthologs and paralogs) within this group of Hemiptera (Fig. 1C; Supplemental Fig. S2).

In summary, we report the first genome sequencing, assembly, and annotation of the MEQ/Q *B. tabaci*. This genome assembly will provide a valuable resource for studying climatic and host plant adaptations, invasive-invasive and native-exotic interactions, insecticide resistance, vector competence, and its relationships with bacterial endosymbionts.

### Availability of supporting data

This whole genome shotgun project has been deposited at DDBJ/EMBL/GenBank under the accession LIED00000000. The version described in this paper is version LIED01000000 accessible at NCBI. Further data, including annotation files and assembled transcripts, are available in the *GigaScience* GigaDB repository [32].

### Additional files

Figure S1. Schematic illustration of the assembly pipeline for MED/Q genome based on the combined assemblies from WGS and BACs.

Table S1. Statistics of the whole genome sequencing data.

Table S2. Repeat Masker analysis in four hemiptera species.

Table S3. Evidenced use within GLEAN MED/Q protein-coding genes.

Table S4. Summary of GLEAN gene models.

Table S5. Functional annotation of the MED/Q genome.

Table S6. Orthologous gene comparison among genomes of 14 arthropod species.

Table S7. Quality control of assembled genome.

### Abbreviations

BAC: Bacterial artificial chromosome; BUSCO: Benchmarking Universal Single-Copy Orthologs; CEGMA: Core Eukaryotic Genes Mapping Approach; EST: Express sequence tag; HMW: high molecular weight; MED/Q: Mediterranean *Bemisia tabaci* Q; mtCOI: mitochondria cytochrome oxidase I; TEs: transposable elements; WGA: whole-genome amplified; WGS: whole genome shotgun.

### Author contributions

YJZ is the leader of the project and the first corresponding author. WX, YJZ, XGZ, YY, JKB, and YL were involved in the project design. XGZ, BYX, JYZ, QG, XCL, XQT, MG, HPP, SXR, and BLQ coordinated the related research works of the MED/Q genome project. DW performed genome assembly. DW performed protein-coding gene annotation. MC and CHC performed gene orthology and phylogenomics. XY performed insecticide targets annotation. YTL performed putative sex determination genes annotation. WX performed putative phloem specialization genes identification. LTG, LXT, YNW, YZ, QJW, SLW, and HYC performed metabolic detoxification systems annotation. ZZY performed immune signaling pathway components annotation. ZZY, JQX, and JQH performed nutrient partitioning between invasive MED/Q and its primary endosymbiont. LTG performed PCR validation. WX, XGZ, DC, JKB, HD, MNM, FG, XPZ, XWW, FHW, YZD, CL, FMY, ELP, and XGJ were involved in writing and editing. All authors read and approved the final manuscript.

### Competing interests

The authors declare no competing interests defined by *Giga Science*.

## Supplementary Material

GIGA-D-16-00061_Original_Submission.pdf

GIGA-D-16-00061_Revision_1.pdf

GIGA-D-16-00061_Revision_2.pdf

Response_to_reviewer_comments_Original_Submission.pdf

Response_to_reviewer_comments_Revision_1.pdf

Reviewer_1_Report_(Original_Submission).pdf

Reviewer_2_Report_(Original_Submission).pdf

Reviewer_2_Report_(Revision_1).pdf

Reviewer_2_Report_(revision_2).pdf

Figure S1. Schematic illustration of the assembly pipeline for MED/Q genome based on the combined assemblies from WGS and BACs.

Table S1. Statistics of the whole genome sequencing data.

Table S2. Repeat Masker analysis in four hemiptera species.

Table S3. Evidenced use within GLEAN MED/Q protein-coding genes.

Table S4. Summary of GLEAN gene models.

Table S5. Functional annotation of the MED/Q genome.

Table S6. Orthologous gene comparison among genomes of 14 arthropod species.

Table S7. Quality control of assembled genome.

## References

[1] Brown JK, Frohlich DR, Rosell RC The sweetpotato or silverleaf whiteflies: biotypes of *Bemisia tabaci* or a species complex? Ann Rev Entomol 1995;40:511–34, doi: 10.1146/annurev.en.40.010195.002455.

[2] De Barro PJ, Liu SS, Boykin LM, *Bemisia tabaci*: astatement of species status. Ann Rev Entomol 2011;56:1–19, doi: 10.1146/annurev-ento-112408-085504. 20690829 10.1146/annurev-ento-112408-085504

[3] Liu SS, Colvin J, De Barro P Species concepts as applied to the whitefly *Bemisia tabaci* systematics: how many species are there? J Inter Agric 2012;11:176–86, doi: 10.1016/S2095-3119(12)60002-1.

[4] Wang HL, Yang J, Boykin LM Developing conversed microsatellite markers and their implications in evolutionary analysis of the *Bemisia tabaci* complex. Sci Rep 2014;4:6351, doi: 10.1038/srep06351. 25220501 10.1038/srep06351 PMC4163675

[5] Tay WT, Evans GA, Boykin LM, Will the real *Bemisia tabaci* please stand up? PLoS One 2012;7:e50550, doi: 10.1371/journal.pone.0050550. 23209778 10.1371/journal.pone.0050550 PMC3509048

[6] Boykin LM, Armstrong KF, Kubatko L, Species delimitation and global biosecurity. Evol Bioinform Online 2012;8:1–37, doi: 10.4137/EBO.S8532. 22267902 10.4137/EBO.S8532 PMC3256992

[7] Boykin LM *Bemisia tabaci* nomenclature: lessons learned. Pest Manag Sci 2014;70:1454–9, doi: 10.1002/ps.3709. 24338873 10.1002/ps.3709

[8] Zhang LP, Zhang YJ, Zhang WJ, Analysis of genetic diversity among different geographical populations and determination of biotypes of *Bemisia tabaci* in China. J Appl Entomol 2005;129:121–8, doi: 10.1111/j.1439-0418.2005.00950.x.

[9] Pan HP, Preisser EL, Chu D, Insecticides promote viral outbreaks by altering herbivore competition. Ecol Appl 2015;25:1585–95, doi: 10.1890/14-0752.1. 26552266 10.1890/14-0752.1

[10] Liu BM, Yan FM, Chu D, Multiple forms of vector manipulation by a plant-infecting virus: *Bemisia tabaci* and tomato yellow leaf curl virus. J Virol 2013;87:4929–37, doi: 10.1128/JVI.03571-12. 23408638 10.1128/JVI.03571-12 PMC3624301

[11] Chen W, Hasegawa DK, Kaur N, The draft genome of whitefly *Bemisia tabaci* MEAM1, a global crop pest, provides novel insights into virus transmission, host adaptation, and insecticide resistance. BMC Biol 2016;14:110, doi 10.1186/s12915-016-0321-y. 27974049 10.1186/s12915-016-0321-y PMC5157087

[12] Chu D, Hu X, Gao C, Use of mitochondrial cytochrome oxidase I polymerase chain reaction-restriction fragment length polymorphism for identifying subclades of *Bemisia tabaci* Mediterranean group. J Econ Entomol 2012;105:242–51, doi: http://dx.doi.org/10.1603/EC11039. 22420277 10.1603/ec11039

[13] Frohlich DR, Torres-Jerez II, Bedford ID, A phylogeographical analysis of the *Bemisia tabaci* species complex based on mitochondrial DNA markers. Mol Ecol 1999;8:1683–91, doi: 10.1046/j.1365-294x.1999.00754.x. 10583831 10.1046/j.1365-294x.1999.00754.x

[14] Li R, Fan W, Tian G, The sequence and de novo assembly of the giant panda genome. Nature 2010;463:311–7, doi: 10.1038/nature08696. 20010809 10.1038/nature08696 PMC3951497

[15] You M, Yue Z, He W, A heterozygous moth genome provides insights into herbivory and detoxification. Nat Genet 2013;45:220–25, doi: 10.1038/ng.2524. 23313953 10.1038/ng.2524

[16] Boetzer M, Henkel CV, Jansen HJ, Scaffolding pre-assembled contigs using SSPACE. Bioinformatics 2011;27:578–9, doi: 10.1093/bioinformatics/btq683. 21149342 10.1093/bioinformatics/btq683

[17] Guo LT, Wang SL, Wu QJ, Flow cytometry and K-mer analysis estimates of the genome sizes of *Bemisia tabaci* B and Q (Hemiptera: Aleyrodidae). Front Physiol 2015;6:144, doi: 10.3389/fphys.2015.00144. 26042041 10.3389/fphys.2015.00144 PMC4436570

[18] Jurka J, Kapitonov VV, Pavlicek A, Repbase Update, a database of eukaryotic repetitive elements. Cytogenet Genome Res 2005;110:462–7, doi: 10.1159/000084979. 16093699 10.1159/000084979

[19] Benson G. Tandem repeats finder: a program to analyze DNA sequences. Nucleic Acids Res 1999;27:573–80, doi: 10.1093/nar/27.2.573. 9862982 10.1093/nar/27.2.573 PMC148217

[20] Edgar RC, Myers EW PILER: identification and classification of genomic repeats. Bioinformatics 2005;21:152–8, doi: 10.1093/bioinformatics/bti1003. 15961452 10.1093/bioinformatics/bti1003

[21] Smit AFA, Hubley R, Green P RepeatMasker. 1999;http://www.repeatmasker.org.

[22] Price AL, Jones NC, Pevzner PA De novo identification of repeat families in large genomes. Bioinformatics 2005;21:351–8, doi: 10.1093/bioinformatics/bti1018. 10.1093/bioinformatics/bti1018 15961478

[23] Xu Z, Wang H LTR_FINDER: an efficient tool for the prediction of full-length LTR retrotransposons. Nucleic Acids Res 2007;35:265–8, doi: 10.1093/nar/gkm286. 10.1093/nar/gkm286 PMC1933203 17485477

[24] Parra G, Bradnam K, Ning Z, Assessing the gene space in draft genomes. Nucleic Acids Res 2009;37:289–97, doi: 10.1093/nar/gkn916. 19042974 10.1093/nar/gkn916 PMC2615622

[25] Simão FA, Waterhouse RM, Ioannidis P, BUSCO: assessing genome assembly and annotation completeness with single-copy orthologs. Bioinformatics 2015;btv351 doi: 10.1093/bioinformatics/btv351. 10.1093/bioinformatics/btv351 26059717

[26] Birney E, Clamp M, Durbin R GeneWise and Genomewise. Genome Res 2004;14:988–95, doi: 10.1101/gr.1865504. 15123596 10.1101/gr.1865504 PMC479130

[27] Burge C, Karlin S Prediction of complete gene structures in human genomic DNA. J Mol Biol 1997;268:78–94, doi: 10.1006/jmbi.1997.0951. 9149143 10.1006/jmbi.1997.0951

[28] Stanke M, Keller O, Gunduz I, AUGUSTUS: ab initio prediction of alternative transcripts. Nucleic Acids Res 2006;34: W435–9, doi: https://doi.org/10.1093/nar/gkl200. 16845043 10.1093/nar/gkl200 PMC1538822

[29] Wang XW, Luan JB, Li JM, De novo characterization of a whitefly transcriptome and analysis of its gene expression during development. BMC Genom 2010;11:400, doi: 10.1186/1471-2164-11-400. 10.1186/1471-2164-11-400 PMC2898760 20573269

[30] Ye XD, Su YL, Zhao QY, Transcriptomic analyses reveal the adaptive features and biological differences of guts from two invasive whitefly species. BMC Genom 2014;15:370, doi: 10.1186/1471-2164-15-370. 10.1186/1471-2164-15-370 PMC4035086 24885120

[31] Su YL, Li JM, Li M, Transcriptomic analysis of the salivary glands of an invasive whitefly. PLoS One 2012;7:e39303, doi: 10.1371/journal.pone.0039303. 22745728 10.1371/journal.pone.0039303 PMC3379992

[32] Xie W, Chen C, Yang Z, Supporting data for “Genome sequencing of the sweetpotato whitefly Bemisia tabaci MED/Q”. GigaScience Database 2017;http://dx.doi.org/10.5524/100286. 10.1093/gigascience/gix018 PMC5467035 28327996

[33] Elsik CG, Mackey AJ, Reese JT, Creating a honeybee consensus gene set. Genome Biol 2007;8:R13, doi: 10.1186/gb-2007-8-1-r13. 17241472 10.1186/gb-2007-8-1-r13 PMC1839126

[34] Trapnell C, Williams BA, Pertea G, Transcript assembly and quantification by RNA-Seq reveals unannotated transcripts and isoform switching during cell differentiation. Nat Biotechnol 2010;28:511–5, doi: 10.1038/nbt.1621. 20436464 10.1038/nbt.1621 PMC3146043

[35] Kanehisa M, Goto S KEGG: kyoto encyclopedia of genes and genomes. Nucleic Acids Res 2000;28:27–30, doi: 10.1093/nar/28.1.27. 10592173 10.1093/nar/28.1.27 PMC102409

[36] Bairoch A, Apweiler R The SWISS-PROT protein sequence database and its supplement TrEMBL in 2000. Nucleic Acids Res 2000;28:45–8, doi: 10.1093/nar/28.1.45. 10592178 10.1093/nar/28.1.45 PMC102476

[37] Zdobnov EM, Apweiler R InterProScan–an integration platform for the signature-recognition methods in InterPro. Bioinformatics 2001;17:847–8, doi:10.1093/bioinformatics/17.9.847. 11590104 10.1093/bioinformatics/17.9.847

[38] Li H, Coghlan A, Ruan J, TreeFam: a curated database of phylogenetic trees of animal gene families. Nucleic Acids Res 2006;34:572–80, doi: 10.1093/nar/gkj118. 10.1093/nar/gkj118 PMC1347480 16381935

[39] Ruan J, Li H, Chen Z, TreeFam: 2008 update. Nucleic Acids Res 2008;36:735–40, doi: 10.1093/nar/gkm1005. 10.1093/nar/gkm1005 PMC2238856 18056084

[40] Guindon S, Dufayard JF, Lefort V, New algorithms and methods to estimate maximum-likelihood phylogenies: assessing the performance of PhyML 3.0. Syst Biol 2010;59:307–21, doi: 10.1093/sysbio/syq010. 20525638 10.1093/sysbio/syq010

[41] Benton MJ, Donoghue PC Paleontological evidence to date the tree of life. Mol Biol Evol 2007;24:26–53, doi: 10.1093/molbev/msl150. 17047029 10.1093/molbev/msl150

[42] Donoghue PCJ, Benton MJ Rocks and clocks: calibrating the Tree of Life using fossils and molecules. Trends Ecol Evol 2007;22:424–31, doi: 10.1016/j.tree.2007.05.005. 17573149 10.1016/j.tree.2007.05.005

[43] Yang Z. PAML: a program package for phylogenetic analyses by maximum likelihood. Comp Appl BioSci 1997;13:555–6, doi: 10.1099/0022-1317-79-8-1951. 9367129 10.1093/bioinformatics/13.5.555

[44] Yang Z. PAML 4: phylogenetic analysis by maximum likelihood. Mol Biol Evol 2007;24:1586–91, doi: 10.1093/molbev/msm088. 17483113 10.1093/molbev/msm088

