# Supplementary material for: Genome sequencing of the sweetpotato whitefly Bemisia tabaci MED/Q
Source: Gigascience. 2017 Mar 15;6(5):1–7. doi: 10.1093/gigascience/gix018 (PMC5467035; doi:10.1093/gigascience/gix018)
Supplement: GIGA-D-16-00061_Original_Submission.pdf [file gix018_GIGA-D-16-00061_Original_Submission.pdf]

Manuscript

Click here to view linked References

Click here to download Manuscript GigaScience_mainbody.docx

For consideration:

Giga Science

The invasive Q-type *Bemisia tabaci* genome: a tale of gene loss and gene gain

Authors and affiliations

Wen Xie[1]¶, Chunhai Chen[2]¶, Zezhong Yang[1]¶, Litao Guo[1]¶, Dan Wang[2], Ming Chen[2], Jinqun Huang[2], Yanan Wen[1], Yang Zeng[1], Yating Liu[1], Xin Yang[1], Jixing Xia[1], Lixia Tian[1], Hongying Cui[1], Qingjun Wu[1], Shaoli Wang[1], Baoyun Xu[1], Xianchun Li[4], Xinqiu Tan[5], Murad Ghanim[6], Huipeng Pan[3], Shunxiang Ren[7], Baoli Qiu[7], Dong Chu[8], Helene Delatte[9], M. N. Maruthi[10], Feng Ge[11], Xueping Zhou[12], Xiaowei Wang[13], Fanghao Wan[12], Yuzhou Du[14], Chen Luo[15], Fengming Yan[16], Evan L. Preisser[17], Xiaoguo Jiao[18], Brad S. Coates[19], Jinyang Zhao[2], Qiang Gao[2], Jinquan Xia[2], Ye Yin[2]*, Yong Liu[5]*, Judith K. Brown[4]*, Xuguo "Joe" Zhou[3]*, Youjun Zhang[1]*

1 Institute of Vegetables and Flowers, Chinese Academy of Agricultural Science, Beijing 100081, China, **2** BGI-Shenzhen, Shenzhen 518083, China, **3** Department of Entomology, S-225 Agricultural Science Center North, University of Kentucky, Lexington, KY 40546-0091, USA, **4** School of Plant Sciences, University of Arizona, Tucson, AZ 85721, USA, **5** Institute of Plant Protection, Hunan Academy of Agricultural Sciences, Changsha 410125, China, **6** Department of Entomology, Volcani Center, Bet Dagan 5025001, Israel, **7** Key Lab of Bio-pesticide Creation and Application, South China Agricultural University, Guangzhou 510642, China, **8** College of Agronomy and Plant Protection, Qingdao Agricultural University, Qingdao 266109, China, **9** Cirad, UMR PVBMT, Saint-Pierre, La Re´union, France, **10** Natural Resources Institute, University of Greenwich, Chatham Maritime, Kent ME4 4TB, UK, **11** Institute of Zoology, Chinese Academy of Sciences, Beijing 100101, China, **12** Institute of Plant Protection, Chinese Academy of Agricultural Sciences, Beijing 100193,

China, **13** Ministry of Agriculture Key Laboratory of Agricultural Entomology, Institute of Insect Sciences, Zhejiang University, Hangzhou 310058, China, **14** School of Horticulture and Plant Protection and Institute of Applied Entomology, Yangzhou University, Yangzhou 225009, China, **15** Institute of Plant and Environment Protection, Beijing Academy of Agriculture and Forestry Sciences, Beijing 100089, China, **16** Collaborative Innovation Center of Henan Grain Crops, College of Plant Protection, Henan Agricultural University, Zhengzhou 450002, China, **17** Department of Biological Sciences, University of Rhode Island, Kingston, Rhode Island 02881, USA, **18** College of Life Sciences, Hubei University, Wuhan 430062, China. **19** United States Department of Agriculture, Agricultural Research Service, Corn Insects & Crop Genetics Research Unit, Ames, IA 50011, USA.

¶These authors contributed equally to the work.

*To whom correspondence should be addressed. Email: Youjun Zhang (zhangyoujun@caas.cn), Ye Yin (yinye@genomics.cn), Xuguo "Joe" Zhou (xuguozhou@uky.edu), Judith K. Brown (JBrown@ag.arizona.edu), Yong Liu (haoasliu@163.com).

Abstract (233 words)

Background: Invasive whitefly, *Bemisia tabaci*, is a highly destructive agricultural and ornamental crop pest. As a group, *B. tabaci* damages host plants through phloem feeding and vectoring plant pathogens. Introductions of *B. tabaci* are difficult to quarantine and eradicate due to high reproductive rates, broad host plant range, and resistance to chemical insecticides.

Results: A 658 Mb draft genome for the Q-type *B. tabaci* (MED/Q) assembled and annotated with 20,786 protein-coding genes. Metabolic pathways show an expansion in the number of gene family members, in particular, the cytochrome P450 monooxygenases. Additionally, amino acid biosynthesis pathways are partitioning among host and endosymbiont genomes in a manner that is distinct from other hemipteran systems, wherein evidence of horizontal gene transfer to the host genome likely form the basis of obligatory relationships. Putative loss of function of the immune deficiency (IMD) signaling pathway due to gene loss is a shared ancestral trait of hemipteran insects that show competency for hosting endosymbiotic bacteria.

Conclusions: This expansion of P450 gene family member may influence the well-noted capacity of MED/Q to adapt to repeated exposures to chemical insecticides, and furthermore, be related to invasiveness in monoculture cropping systems where such applications are prevalent. This whitefly genome lays the foundation for research into the evolution of endosymbiotic relationships, as well as mechanism(s) that provide a competitive advantage to invasive species, both which likely contribute to the unprecedented worldwide success of *B. tabaci* invasions.

Keywords

Bemisia tabaci, Detoxification enzymes, Genome, Gene gain and loss, Invasive species, Symbiosis

Background

As a globally invasive species, the whitefly *Bemisia tabaci* (Genn.) has been found on all continents except Antarctica [1,2]. Taxonomically, *B. tabaci* are considered sibling species or a 'cryptic' group whose classification at the species level has long been confounded due to the inability to distinguish members based on morphology, despite evidence that there are several biological and genetic differences [2,3]. The group has a marked phylogeographic structure based on relationships between geography and phylogenetic clades reconstructed from a mitochondrial cytochrome c oxidase subunit I, mtCOI, gene fragment [2,4]. The cryptic *B. tabaci* are phloem-feeding insects that colonize predominantly herbaceous eudicot plant species [5], and is reported to infest over 900 species including a large number of food, fiber, and/or ornamental crops (Global Invasive Species Database). As a group, *B. tabaci* also vector over 111 plant viruses across six plant genera, which often leads to more significant impacts on plant health compared to direct phloem feeding [6,7].

The impact of *B. tabaci* on global agriculture has increased substantially due to accidental introductions into previously uninhabited cropping regions, which has propelled *B. tabaci* into one of the World's Worst Invasive Species (Global Invasive Species Database: http://www.issg.org/database/welcome/). Specifically, invasive *B. tabaci* from the Middle East-Asia Minor 1 (MEAM1) sub-species or B-biotype has emerged as a major pest in the United States, Caribbean Basin, Latin America, Middle East [1], and East Asia [8]. Analogously, *B. tabaci* sub-species MED/Q was inadvertently introduced into several geographic locations worldwide, and became established throughout China [9,10]. Despite of the commonality of the invasive natures of MEAM1/B and MED/Q, these two subspecies appear adapted for optimal growth on different host plants and/or virus infected plants [10,11], and genetically differentiated based on the greater competence of MED/Q to vector for *tomato yellow curl leaf virus* ('*TYLCV*') [9,12]. The selective advantage of invasive

MEAM1/B may be related to the capability to increasing the ratio of diploid female to haploid male progeny during range expansions, which leads to numerical and competitive displacement of native *B. tabaci* [13]. Correspondingly, invasive MED/Q has developed resistance to several classes of chemical insecticides, which may lend a selective advantage in comparison to more susceptible endogenous biotypes in agricultural landscapes [9]. Due to the impact of invasions on agricultural production and persistence within introduced geographic ranges, MEAM1/B and MED/Q have arguably become the most extensively studied whitefly groups and have been proposed as models for range expansion through adaptations to invaded habitats. In spite of this, the genetic or genomic basis of the adaptive plasticity and subsequent selective advantages of these invasive *B. tabaci* biotypes remain obscure.

Analogous to other phloem-feeding hemipterans, an obligate bacterial endosymbiont has co-evolved to contribute metabolic end products of essential amino acids and vitamins that are required for survival on nutritionally-incomplete sap. All members of the *B. tabaci* group rely on the primary endosymbiont *Candidatus (Ca.) Portiera aleyrodidarum* (*Portiera*) [14], but can also harbor one or more additional facultative bacterial endosymbionts with unknown contributions to host survival [15,16]. This symbiosis has evolved beyond simple provisioning by intracellular bacteria, but into systems wherein metabolic pathways are intricately interconnected due to complementation of enzymatic components in key metabolic pathways that has occurred though a series of gene loss or gain. The adaptive changes in host immune pathways and pathogen detection, as well as the mechanisms that led to endosymbiont evasion of these defenses remain poorly understood. Moreover, variation in endosymbiont communities within MED/Q is associated with distinct host haplotypes, and increasing evidence suggests that these endosymbiont communities may influence the competency of these *B. tabaci* to vector *TYLCV* [17].

Here we report the first draft genome sequence for the invasive and highly adaptive *B. tabaci* MED/Q-type. Changes in the number and diversity of metabolic gene pathway members and pathogen recognition pathways are examined in order to glean insight into the adaptability of MED/Q to range of plant host ranges and chemical insecticide exposures, as well as facilitating the establishment of endosymbiotic relationships. The MED/Q genome sequence provides a resource for future investigation of climatic and host plant adaptations, insecticide adaptations, vector competence and transmission, and the myriad of endosymbiotic bacterial relationships that may influence the propensity to become invasive. Furthermore, research stemming from this genome resource may and allow for the development of knowledge- based whitefly management tactics and a reference for invasive species research.

Data Description

For details on assemblies, annotations and other analyses see 'methods' section. And this whole genome shotgun project has been deposited at DDBJ/EMBL/GenBank under the accession LIED00000000. The version described in this paper is version LIED01000000. The final, assembly *Portiera* (PRJNA299729/SAMN04214819/LNJY00000000) and *Hamiltonella* (PRJNA299727/SAMN04214805/LNJW00000000) genome of MED/Q, respectively, are accessible at NCBI.

Analyses

Strain selection, genome sequencing and assembly

Results of mtCOI gene PCR-RFLP assays [18], and direct DNA sequencing followed by phylogenetic evaluation against reference sequences [19] both indicated the *B. tabaci* in the MED/Q colony is a member of the Q1 haplotype group, or western Mediterranean region clade (data not shown). Using genomic DNA from the MED/ colony, a total of 20 whole genome sequence (WGS) shotgun sequencing libraries were generated (18 pooled male and female PE and MP libraries, and two haploid-male derived WGA PE libraries), from which sequence was generated on an Illumina Hiseq2500 platform.   In total library sequencing produced 428.2 Gb or an approximate 594.7-fold genome coverage assuming a 0.72 Gbp genome size (based on 17-mer analysis). For the 10 short-insert PE libraries, a total of 229.4 gigabases (Gb) (100 bp or 150 bp read length, approximately 318.6-fold genome coverage). Sequencing of the eight large-insert (>1 kb) MP libraries produced 80.3 Gb of reads (49 bp read length, 111.5-fold coverage) were further obtained and used for scaffold construction (S1 Table). Additionally, the two male WGA libraries produced, a total of 118.5 gigabases (Gb) of data (S1 Table) or approximately 164.6-fold genome coverage Sequencing of 13

BAC pools generated 362.6 Gbp of raw data (288.4 Gbp processed data; results not shown). The subsequent assembly of this sequence data using our pipeline (S1 Fig) generated a 658.0 Mbp draft genome assembly for *B. tabaci*, with a scaffold N50 of 437 kb (Table 1). The assembled 658 Mb MED/Q draft genome size is consistent with recent estimates by flow cytometry [20]. The mean read depth across 10 kb windows indicated that all genome regions were highly represented within the read data, with < 1.5% having a depth of < 10X (remaining data not shown).

Annotation of repetitive elements

Homology-based annotation of *B. tabaci* repetitive elements queried against Repbase v.20.05 [21] with RepeatMasker [22] predicted a total of 299.0 Mbp repetitive DNA or 45.4% of the assembled genome size. These results predict that the number of long terminal repeat (LTR)-like retroelements comprised the greatest proportion and nucleotide contnes compared to all other TE classes (S2 Table). This proliferation of LTR retrotransosons is comparatively unique to other hemipterin genomes, with the exception of *N. lugens*. The MED/Q genome also shows a high proportion of DNA-based TEs, but is consistent with other full genomes from the Order Hemiptera. In contrast, the MED/Q genome appears devoid of short interspersed nuclear elements (SINEs), and is analogous to predictions previously made for the aphid species *N. lugens* and *R. prolixus*.

Gene coverage and annotation of coding regions

Preliminary evaluation transcribed regions represented within the draft MED/Q genome assembly coverage determined that ~95.2% of *B. tabaci* ESTs > 200 bp were present, with 90,652 ESTs showing ≥ 90% length coverage on one scaffold. This alignment encompassed

92.9% of nucleotides within the EST dataset. Analogously, 229 (96%) of the 248 sequences in the CEGMA gene set were present in the MED/Q genome assembly, which is less than the 245 and 236 obtained from *A. pisium* and *R. prolixus* genomes, respectively (remaining data not shown). The final gene GLEAN gene models predicted a reference gene set of 20,786 protein-coding genes, which was a consensus results from the best evidence from *de novo*, orthology, and evidence (RNA-seq)-based prediction methods (S3 Table), and integrated into GLEAN gene models (S4 Table). Among the GLEAN gene models, 16,622 (79.97%) received functional gene annotations using the various databases queried in our analysis pipeline (S5 Table) A among the un-annotated GLEAN gene models 57% were supported by either RNA-seq or EST sequence data (remaining data not shown).

Prediction of gene orthology and gene family expansion

Phylogenetic analysis based on orthologs across 14 arthropod taxa (S6 Table) suggested that *B. tabaci* Q-type is clustered into a hemipteran clade containing the pea aphid, and is a sister lineage to a clade containing the true bug *R. prolixus* and the rice planthopper *N. lugens* lineages (Fig 1A). Within this prediction of orthologs, the range of species-specific genes within the four hemipteran genomes ranged from 38-60%, and was especially pronounced in three plant-phloem sap specialist. This led us to investigate further the overall expansion or contraction among gene family members (orthologs and parlogs) within species found in this group of Hemiptera (Fig 1C; S2 Fig). This assessment suggest that 1978 gene models unique to *B. tabaci* are associated with putative gene family expansions, and the most highly expanded gene families are enriched in the functional categories of transmembrane transport and oxidative-reduction processes (S7 Table). The number of *B. tabaci* gene family members among the UDP glycosyltransferases (UGTs; *n* = 63), carboxyl/choline esterases (COE; *n* = 51), and ATP-binding cassettes transporters (ABC; *n* = 59) was not significantly different

compared to other plant phloem or blood feeding arthropods. In contrast, the cytochrome monooxygenase P450 detoxification gene family is expanded in number (Fig 2A). Specifically, the 56 expansions in the P450s gene family in *B. tabaci* is the highest predicted number among hemipteran genomes, and second only to *T. castaneum* (n = 68) among the 14 fully sequenced arthropod genomes (S8 Table). This expansion across evolutionary time has resulted in a total of 153 predicted *B. tabaci* P450 genes that is the greatest number compared to other arthropods, and show particular expansion in the CYP3 and CYP4 clades (Fig 2B).

On the other hand, 3474 genes belong to groups that show putative reductions in the number of their associated gene family members, and mainly of these were annotated as being involved in RNA-dependent DNA replication and DNA integration (e.g. difference in transposon component of the genomes), and cell surface receptor signaling (e.g. immune response; S9 Table). Further inspection of gene annotation information showed that genes in the immune deficiency (IMD) pathway were absent from the MED/Q genome (Fig 3; S10 Table).

Metagenomics and analysis of MED/Q endosymbiosis

The bacterial metagenome of insects has been shown to contribute to overall insect health and viability, and in many hemipteran insects have evolved specialized structures, bacteriocytes, that house endosymbiotic bacteria. Although genomes for the primary Q-type *B. tabaci* endosymbiont, *Ca. Portiera* aleyrodidarum (CP003867, CP003835 and CP007563) and secondary endosymbiont, *Hamiltonella* (AJLH00000000, AJLH02000000) [14,16], the lack of a corresponding whitefly genome sequence has precluded investigations into this interaction with respect to metabolic and gene pathway partitioning. Using a metagenomics approach, we were able to re-assembled the complete genomes of *Portiera* (0.35 Mb) and *Hamiltonella* (1.8 Mb) from filtered Illumina reads generated from shotgun sequencing

libraries.

Following comparison of gene models with function annotations as putatively involved in amino acids biosynthesis within the *B. tabaci* and endosymbiont genome assemblies and reconstruction of the corresponding enzymatic pathways, a a close interrelationship was revealed between *B. tabaci* and *Portiera*. Specifically, of the 47 and 45 enzymes involved in amino acid biosynthesis respectively encoded in the MED/Q and *Portiera* genomes, and the intact pathway requires enzymatic components encoded by both genomes. Pathway analysis showed that complementation between these two genomes is need for synthesis of 10 essential amino acids; Arg, His, Ile, Leu, Lys, Met, Phe, Try Thr, and Val (Fig 4). The whitefly MED/Q encodes enzymes that contribute precursor substrates for synthesis Trp, Phe and Thr, whereas, and those encoded by *Portiera* complete synthesize of the final amino acids within the bacteriocyte. Conversely, *Portiera* is predicted to provide the intermediates required by MED/Q to synthesized the Arg, His, Ile, Leu, Lys, Met, Tyr, and Val amino acids (Fig 4A). Furthermore, analogous amino acid biosynthesis pathway analysis provided evidence that the *B. tabaci* endosymbiont *Hamiltonella* genome encodes independent Cys, Lys, Pro, and Thr biosynthetic pathways (Fig 5). Comparative genomic pathway analysis additionally showed that MED/Q putatively lacks the capacity to synthesize five B-class vitamins; biotin, folate, NAD, riboflavin, and vitamin B6. Inspection of the genome of the secondary endosymbiont, *Hamiltonella* is predicted to encode enzymes that fully biosynthesize these 6 B vitamins (S11 Table; S12 Table).

The hypothesis that horizontal gene transfer (HGT) may be involved in the gain of certain amino acid biosynthetic pathway componends was tested using a phylogentic approach, which provided evidence that endosymbiotic bacterial genes may have been acquired by the *B. tabaci* MED/Q genomes. Furthermore, the HGT have occurred with *Portiera* as the donor. Based on our phylogenetic pipeline used for detection of HGT, we

identified eleven putative events based on phlogentic clustering of *B. tabaci* genes with bacterial counterparts (S3 Fig). Of these 11 predicted HGT events, ten MED/Q encoded genes clustered and were predicted to have closest relationships to bacterial orthologs. In the other instances, the MED/Q gene for argininosuccinate synthase was at the base of a claded of bacterial origin and adjacent to a second insect-derived clade (S3A Fig). Interestingly, functional annotations predict that six HTG events putatively involving argH, 2 dapF paralogs, lysA, dapB, and E3.1.3.15B, are likely to participate in the complementation of the Arg, His, and Lys biosynthetic pathways (Fig 4A). Six *B. tabaci* genes involved in of these 11 putative HTG events (54.5%) contained introns and 4 (36.4%) have a 5'-untranslated region (UTR), in spite of showing closest evolutionary relationships to prokaryotic orthologs (S13 Table). Comparisons between amino-acid synthesis pathways in the *B. tabaci-Portiera*, *A. pisum-Buchnera* and *N. lugens*-yeast-like host-endosymbiont relationships indicate that , we found that MED/Q and its endosymbionts contributed robustly to the amino acid pool, in that through their symbiosis they contribute 7 of the 10 essential amino acids (Fig 4B). By comparison, the genome sequences of the MED/Q counterpart aphid- and planthopper-endosymbiont study systems encode primarily transaminases (S14 Table), and provide more substrates or intermediates involved in the regulation of amino acid synthesis. While the latter insect-endosymbionts have a role in glycolysis and the pentose phosphate pathway, *Portiera* lacks a detectable role in either pathway (Fig 4A,C).

Discussion

The Q-type *B. tabaci* are agricultural pest insects that cause significant levels of crop damage, and are endemic or invasive across most geographic regions of the world. The species complex has a broad plant host range that spans across most eudicot plant families, high fecundity and reproductive rate, and capacity to adapt to a range of local environmental conditions. Arguably, the evolution of these traits may contribute the observe ability of whiteflies to develop resistance to different chemical insecticide classes, as well as become established and invasive within in novel habitats [1,4]. Evolutionary histories and past exposures to selective pressures can be envisioned to shape the gene complement of a genome, wherein adaptation that allow increased propensity to survive can influence the breath of functions carried out by certain gene families [23]. For instance, cytochrome P450 monooxygenase genes encode enzymes involved in a variety of biosynthetic and metabolic pathways, including xenobiotic detoxification [24,25], and form a repertoire of defensive pathways herbivorous insect have adapted to feeding on plants that contain defensive secondary metabolites [26]. Copy number increases within gene families involved in metabolic detoxification is as a mechanism hypothesized to facilitate survival when exposed to environmental toxins due to increased metabolic rates when the duplicates are co-regulated [27]. In contrast, evolutionary processes can result in diversification of duplicated gene families, such that temporal and spatial variation occurs with respect to gene expression pattern or members derive modified functions [28].

Analysis of full arthropod genome sequence assemblies has identified lineage specific expansions in the P450 gene family, where CYP3 and CYP4 clades have expanded in the armor-tailed scorpion, *Mesobuthus martensii* [29]. Interestingly, expansion in the number of P450s in CYP3 and CYP4 clades also occurred in the genome of *Tribolium castaneum* [30], and here in the *B. tabaci* MED/Q genome (Fig 2). This may not be surprising since CYP3 and

4 members have been implicated with the detoxification and evolution of resistance to insecticides among arthropod species [31-35]. The expansion of these detoxification genes could be hypothesized to contribute towards the observed adaptability and evolution of resistance in of type-Q *B. tabaci*, but future comparative genomic studies are required to fully investigate any correlations between specific gene family member expansions and differing biological/ecological characteristics between subspecies. It is important to understand the genetic and genomic mechanisms that lead towards to propensity of arthropods to withstand broadcast insecticide applications in order to potentially devise practices to circumvent this cycle of recurrent adaptation. It also remains enticing to suggest that introduction and establishment of invasive *B. tabaci*, and concomitant displacement of extant whitefly biotypes could have a genomic basis that is directly correlation to biological success. For instance, following introduction of Q-type *B. tabaci* into China the previously invasive B-type *B. tabaci* was driven from those locales [9,36]. Indeed, it was previously shown that *B. tabaci* P450 genes can be induced under various conditions, including host plant switches and unregulated in insecticide resistant strains [37,26]. However, these genes are also constitutively expressed at a basal level under normal conditions [38], suggesting either the genes are primed for rapid response to stress or they are involved in cellular homeostasis. Additionally, insect UGT are primarily sugar donors that are closely related to various plant allelochemical- and odorant-degrading enzymes [39-41]. In *B. tabaci*, it is shown that UGT have a role in adaptation to feeding on nicotine [42], an abundant plant secondary metabolite, suggesting that UGT may function in whitefly adaptation to certain plants. Our results from genomic comparison of detoxification gene families provides hypothesis regarding the capacity of MED/Q to feed on a relatively wide range of host plant species, develop insecticide resistance, as well as adapt to novel biotic stresses when introduced into non-native environments.

Endosymbiosis between host eukaryotic calls and intracellular microorganism involves a series of adaptive changes that facilitate their co-existence, with the most ancient being the hypothesized acquisition of environmental bacterial and subsequent HGT that led to the current mitochondrion and nuclear-encoded components mitochondrial of the ATP biosynthesis pathway. Insects contain a diversity of intracellular bacteria and fungi, of which a proportion has formed intricate mutualitstic relationships, in particular within the insect order Hemiptera [43,44]. The impact of these types of symbiotic relationships on the metabolic capacity of host genomes are just starting to be revealed through whole-genome analyses [45] In particular, all *B. tabaci* biotypes house the primary obligatory Gram-positive endosymbiont *Portiera aleyrodidarum* [46], as well a variety of biotype-specific secondary endosymbionts including *Arsenophonus*, *Wolbachia*, *Hamiltonella*, *Candidatus*, *Cardinium*, *Fritschea* and *Rickettsia* [47-50]. Previous studies have alluded to potential metabolic complementation between *B. tabaci* and *Portiera*, as well as between *B. tabaci* and the secondary endosymbiont *Hamiltonella* [15,16]. Transcriptome-based analysis of B-type *B. tabaci* comparing bacteriocytes and whole-body samples reported that the host genome may contribute enzymes that complement or duplicate *Portiera*–encoded pathways, and that *Hamiltonella* might contributes multiple cofactors and the essential amino acid, Lys [15]. The genome of the MED/Q primary endosymbiont, *Portiera*, was found to lack genes essential for the biosynthesis of certain amino acids, which was proposed to support previous reports from transcriptome data [14,51]. The extent to any gene reduction or interdependency of metabolic pathways in the symbiotic relationship of Q-type *B. tabaci* has yet been fully assessed until now. Specifically, we determined that *Portiera* contributes towards a complete *B. tabaci* diet from phloem sap by providing pathway components required for synthesis of 10 essential amino acids (Fig 4), and furthermore the secondary endosymbiont *Hamiltonella* likely uniquely contributes B-vitamins that cannot be generated by the anabolic pathways

within the host (Fig 5B).

Of greater interest, we show that many of these pathways are completed only via complementation between host and the obligatory endosymbiotic bacteria, which suggests that evolution has in many cases generated relationships cannot be disentangled without severe consequences on both participants. Complementation could conceivably evolve through gene loss in either the host or endosymbiont due to relaxation of selective constrains when two copies are presents, but has also been shown to occur through HGT. For instance, enzymes involved in carotenoid biosynthesis were derived from fungal genes integrated into the pea aphid *A. pisum* genome [52]. Also, horizontally transferred genes (HTGs) identified in the citrus mealybug, *Planococcus citri* contribute to gene functions that were absent in its endosymbionts, where a completely system has evolved to synthesize amino acids [53]. Our discovery that 11 enzymes within complemented amino acid biosynthetic pathways between *B. tabaci* and *Portiera*, have likely been acquired by the host though HGT suggests that movement of pathway component between genomes may be an initial step in forming obligatory endosymbiotic relationships.

Of further interest is the modification of insect immune systems that facilitate the residency of previously extracellular bacteria that otherwise might be recognized as pathogenic agents. Specifically, acceptance of a foreign bacteria by the host or evasion of host defenses by the bacteria may be consider the seminal event that is required prior to the evolution of all subsequent symbiotic interdependencies. The IMD pathway is activated in *Drosophila melanogaster*, in response to infection by Gram-negative bacteria [54]. Given that *B. tabaci* and *A. pisum*, obligate endosymbionts are both Gram-negative bacteria [54,55], the loss of the all components of the IMB pathway from the insect host may be considered more that incidental. It is possible that loss of the IMB function allowed for the acquisition of endosymbiotic bacteria [56]. Moreover, we estimated the divergence time of the common

ancestor of *B. tabaci*, *A. pisum* and *N. lugens* to be 286 million years ago (MYA) (Fig 1; S4 Fig) which is consistent with recent results showing that the time of divergence of the Auchenorrhyncha (containing *N. lugens*) and Sternorrhyncha (containing *A. pisum* and *B. tabaci*) was approximately 290 MYA [57]. These evolutionary events and recent studies may suggest that the ancestors of *B. tabaci* and *A. pisum* acquired their obligate endosymbionts after separating from the group that also contained *N. lugens*. During this process, it could have been necessary to disarm the IMD system. The absence of a solvent IMD pathway in MED/Q may possibly also have contributed to subsequent acquisition of *Hamiltonella*, and perhaps other facultative endosymbionts that have been detected among whitefly biotypes.

Conclusions

This *B. tabaci* MED/Q genome is the first genome of this whitefly and for a widespread invasive cryptic species. Key findings and insights gained into the genes/proteins involved in metabolic gene expansion, nutrient/nutritional partitioning, and unique immune system reduction, shed new light on the genetic and molecular basis underlying multiple environmental adaptations that are inherent to whiteflies, and may have contributed to the noted invasive behavior of the species. MED/Q-like haplotypes of *B. tabaci* are endemic to the eastern and western Mediterranean regions in agricultural settings, where those populations have evolved moderate to extreme polyphagy and developed resistance to commonly appliedinsecticide, Invasion, establishment, and subsequent range expansions into naïve geogrphic region have been particularly promounced in irrigated, monoculture crop production systems throughout the Mediterranean Basin, parts of the Sahel region of Africa, in coastal areas of portions of the Middle East, and most recently, in eastern China. The application of common control measures across large contiguous areas of these monoculture cropping systems could arguably have facilitated the rapid displacement of other more

susceptible *B. tabaci* biotypes by MED/Q. Although direct linkage has not been established between the cytochrome P450 gene family expansion in the MED/Q genome and increased survivorship when exposed to chemical insecticides, the correlation may arguably not be coincidental since this class of genes is well characterized for their role in detoxification. Thus, this copy number expansion in this gene family may have resulted from historical selection for survival on an array of plant defensive compounds, which have translated into the analogous capacity to adapt and survive exposures to man-made insecticidal compounds.

Juxtaposed to this P450 gene family expansion is the trend towards reductionism in the IMB pathway, which could be interpreted as an adaptive mechanism that has led to acceptance of intracellular endosymbiotic bacteria that is share across several hemipteran genomes which have been sequenced to date. The evolutionary pretext that led in host accommodation of one or more endosymbionts and concurrent adaptation to phloem feed through complementation of pathways required for amino acid and B vitamin biosynthesis remains in question. Regardless that current status of gene pathway complementation in the host could likely have resulted from a combination of gene loss by random genetic drift and gain via horizontal gene transfer from the endosymbiont – a process eerily reminiscent of events hypothesized to have resulted in acquisition of the mitochondrion from a once free-living bacteria by eukaryotes. The known myriad of other endosymbionts and combinations thereof suggests that variation in gene movement and pathway complementation may pervade within and across *B. tabaci* biotypes. Given the evidence that variation in vector competency can be effected by endosymbiont composition of a *B. tabaci* biotype or haplotype, it is intriguing to hypothesize these different repertoires of endosymbionts may influence other life history and/or adaptive traits. Indeed the scope of research likely required to investigate the adaptive and invasive nature, as well competency for transmission of viral pathogens and acquiring endosymbiotic bacteria, is broad. The genomic resource of MED/Q will likely

provide a solid foundation for the near-future "pan-genomic" comparison of invasive and non-invasive, and native and exotic *B. tabaci* haplotypes, and as wll as open new avenues of investigation into whitefly biology, evolution, and possibly even more effective methods for their control.

Potential implications (101)

Members of sibling species of whitefly, *Bemisia tabaci*, are among the most invasive species in the world, and cause significant damages to agricultural and horticultural crops through direct phloem feeding and indirect vectoring of plant pathogens. By sequencing the genome of the Mediterranean Q-type *B. tabaci* (MED/Q) we uncovered molecular signatures of adaptation over evolutionary time which may contribute to the highly invasive and adaptive characters of this pest. Numerical and compositional changes in gene families described therein provides a foundation for future hypothesis testing that will furthering our understanding of ecological adaptation, viral transmission, symbiosis, and plant-insect-pathogen tritrophic interactions.

Methods

Strain selection and Illumina library construction

The Q-type *Bemisia tabaci* was first reported in China when it was identified on *B. tabaci*-infested poinsettia plants imported into the Yunnan Province during 2003 [58]. Within only several years, the Q-type (MED) *B. tabaci* had invaded most of the southeastern and northeastern provinces in China, where it rapidly displaced the exotic B-type (MEAM1), previously established there [36]. Adult whitefly females (2n) and males (1n) were initially collected from infested field-grown cucumber plants in Beijing, China during 2011, and used to establish alaboratory colony (MED/Q) at the Institute of Vegetable and Flowers, Chinese Academy of Agriculture Science by transferring adult males and females to caged pepper plants (10-12 leaf stage).

The MED/Q whitefly colony was used as the source initial short shotgun Illumina sequencing. Adult whiteflies fed using Parafilm ®membrane sachets containing a 25% sucrose solution for 48 hrs prior to collection of ~5,000 male and female adults (~ 50:50). Samples were immediately frozen in liquid nitrogen for three hours prior to transfer to a -80°C freezer. This genomic DNA was used to construct Illumina TruSeq paired end (PE) sequencing libraries (170, 250, 300, 500 and 800 bp insert sizes) and mate pair (MP) libraries (2, 5, 10, 20 and 40 KB in size) according to manufacturer instructions.

Additionally, two Illumina PE sequencing libraries (~500bp and 800bp inserts) were constructed from whole genome amplification (WGA) reactions carried out on genomic DNA isolated from two adult male whiteflies. First, single males were dissected (body fluid and interior unclassified tissues by removing the outer cuticula) into 1x PBS buffer, and frozen for storage and transport. The PBS buffer containing the cells and tissues was thawed, briefly flicked to mix, and pipetted into PCR tubes in equal amounts. After blending and dissolving adequately, WGA of the DNA from a single male was performed according to the

manufacturer's instructions using the REPLI-g Midi Kit (QIAGEN, Inc.), with a "no whitefly tissue" cell reaction as a negative control. Each step during the experiment was performed on a "clean bench" to avoid contamination. To assess the effect of the amplification, the DNA concentration of the WGA products was measured in a Qubit® dsDNA assay (Invitrogen Life Science, Inc.) according to the manufacturer's instructions, and the primary DNA band-size distribution was validated by agarose gel electrophoresis.

BAC library construction, pooling of clones, and Illumina library construction

High molecular weight (HMW) MED/Q total genomic DNA was isolated as previously described [59], then partially digested with *Hind*III , and size selected from pulsed-field gel electrophoresis (1% agarose gel in 0.5 X TBE gels run on BioRad CHEF-DRIII system) run for 16 hours at 14°C with initial and final time switches of 5 sec and 15 sec, respectively, in a voltage gradient of 6 V/cm. Sizes were compared to the Lambda Ladder PFG Marker (New England Biolabs), and ~100 kb genomic fragments exized. Isolated DNA was ligated into the pCC1BAC vector (Epicentre), transformed into EPI300 *E. coli* cells (Epicentre) by electroporation, and selected on LB agar with chloramphenicol, X-gal and Isopropyl-beta-D-thiogalactopyranoside (IPTG) as described by the manufacturer. A Genetix QPIX was used to pick and array clones into 192 384-well plates containing LB freezing media, and then frozen at -80°C. To BAC vector estimate insert sizes, 10 µl aliquots of BAC miniprep DNA were digested with 5 U of *Not*I enzyme for 2 hours at 37°C, and then separated by pulsed-field gel electrophoresis as described above. All clones from 384-well plates were pooled into 13 libraries (8 or 9 384-well plates per pool), and BAC vector DNA was isolated from LB liquid cultures using Qiagen Large Construct Kits. Purified BAC DNA was used to construct indexed Illumina TruSeq PE libraries with ~500 bp insert size according to manufacturer instructions.

Genome sequencing and assembly

All libraries were sequenced on an Illumina Hiseq 2000 using 100 bp reads from both fragment ends, and raw data processed and assembled as shown (S1 Table; S1 Fig). Briefly, a series of filtering steps were performed on the raw reads to filter out the following: (1) reads with >10% Ns, more than 40% low-quality bases, more than 10 bp overlapping with adapter sequences, allowing no more than 3bp mismatches; (2) paired-end reads that overlapped more than 10 bp between two ends, with insert size larger than 200 bp libraries; and (3) duplicated reads generated by PCR amplification during the construction of the large-insert library. Filtered reads were used for K-mer determination within subsequent assembly steps. The frequency of each K-mer was calculated from the genome-sequence reads. K-mer frequencies along the sequence depth gradient follow a Poisson distribution in a given data set except for a high proportion at low frequency due to sequencing errors, as K-mers that contain such sequencing errors may be orphans among all splitting K-mers. The genome size, G, was estimated as $G = K\_num/K\_depth$, where K_num is the total number of K-mers, and K_depth is the maximal frequency. Initial contigs were assembled from filtered 500 and 800 bp insert-size WGA PE libraries using SOAPdenovo. The sequencing reads obtained for 2k-40kb MP libraries were used to connect the contigs and to generate the scaffolds as described by Li et al. (2010) [60] with a K-mer size of 65.

Individual BAC pools were assembled independently using SOAPdenovo and the whole genome shotgun reads from PE and MP libraries were used to fill gaps in the BAC scaffolds. After sequencing, the raw reads were filtered as described above. In addition, reads representing contamination by *E. coli* or the plasmid vector were filtered. The pooled reads were separated according to the BAC-reads index, and each BAC was assembled using a

combination of “hierarchical assembly” and “*de Bruijn* graph assembly”. First, the reads linked to each BAC were assembled using SOAPdenovo [60], with various combinations of parameters with a K-mer range from 27 to 63 and a step size of 6. The assembly with the longest scaffold N50 was defined as the “best” for each BAC. The resulting BACs were mapped with the large shotgun MP read data to optimize the assembly for each BAC.

The final draft assembly was produced by integrating sequences that overlapped among the scaffolds independently assembled from genome shotgun and BAC reads, and in doing so eliminated the redundant scaffolds using the following steps. In order to integrate the two assemblies, the software *Rabbit* [61] was applied to identify any relationship between scaffolds, to connect the overlapping regions that shared at least 90% similarity, and to remove redundancy based on a 17-mer frequency. Finally, *SSPACE* [62] was used to construct super-scaffolds containing 800 bp-40 kb WGS reads, and the 170-800 bp genome shotgun read data were used to fill the gaps using *GapCloser* [60].

Post-assembly processing included removal of contaminating bacterial and viral DNA sequences, by aligning all assembled sequences to the genome sequences of viruses and bacteria, obtained from previous local BLASTn alignments and by NCBI upload filter. Aligned sequences that shared >90% identity and were >200 bp in size were filtered from the final assembly. The assembled sequences that were covered by at least one EST sequence were retained. Process read data was mapped the the draft MED/Q genome using *SOAPaligner* software and read counts were made from .bam files and the average depth was computed from all bases in the window. The relation graph of base pair percentages, and each given sequencing depth along the genome, was obtained.

Annotation of repetitive elements

Repetitive elements were searched for and identified using *Repbase* [21] implemented in *TRF* software [63], and a *de novo* approach implemented in *Piler* [64]. For the *Repbase*-based method, two software programs named *RepeatMasker* [22] and *RepeatProteinMask* were used to identify repetitive sequences. In the *de novo* approach, *Piler-DF-1.0* [64], *RepeatScout-1.0.5* [65], and *LTR-FINDER-1.0.5* [66] were used to build *de novo* repeat libraries from the genome sequences. Finally, the repeated sequences were searched for and classified using the *RepeatMasker* software.

Gene coverage and annotation of coding regions

Initial evaluation of gene coverage rate in the draft MED/Q1-China genome assembly was assessed by comparing against 248 core eukaryotic genes were obtained using *CEGMA 2.4* [67]. Additionally, 105,067 *B. tabaci* transcript sequences, expressed sequence tags (ESTs), of > 200 bpwere used as BLASTn queries against the assembled genome in order to estimate the representation (cutoff E-value ≥ 10^{-40}). Protein-coding gene *de novo* predictions using GENEWISE [68] and *ab initio* gene predictions using GENSCAN [69] and AUGUSTUS [70] were made in combination with 13.7 Gbp of transcriptome (RNA-Seq) data including published Q-type *B.tabaci* body, guts, and salivary glands [71-73] and additional unpublished females and males data (FTP: http://111.203.21.119/download/*.fastq), to obtain consensus gene sets using GLEAN [74].

For homolog-based prediction, protein sequences from nine species (*A. pisum*, *A. mellifera*, *D. melanogaster*, *R. prolixus*, *Z. nevadensis*, *A. gambiae*, *B. mori*, *P. humanus* and *T. castaneum*) were aligned with the MED/Q genome scaffolds using *TblastN* (E-value<1e-5). Target sequences were used to search for accurate gene structures implementing the *GeneWise* software [68]. For the RNA-Seq datasets, the transcriptome reads were first aligned against the genome using *TopHat* [74] to identify candidate exon regions. Then, the

Cufflinks software [75] was used to assemble the aligned reads into transcripts, and the open reading frames (ORFs) were predicted to obtain reliable transcripts using a Hidden Markov Model (HMM)-based training parameter. Finally, *GLEAN* [74] was used to integrate the predicted genes with the *de novo*, homologous, and RNAseq data to produce the final gene set. The functional annotation of genes was performed using *BLASTP* alignment to KEGG [76], SwissProt and TrEMBL [77] databases. Motifs and domains were determined by *InterProScan* [78] and protein database searches against ProDom, PRINTS, Pfam, SMART, PANTHER and PROSITE.

Prediction of gene orthology and gene family expansions

Twelve insect species including *Bemisia tabaci* (Genn.) (Gennadius, 1889) (Hemiptera: Aleyrodidae), *Acyrthosiphon pisum* (Harris, 1776) (Hemiptera: Aphididae), *Rhodnius prolixus* (Stal, 1859) (Hemiptera: Triatominae), *Nilaparvata lugens* (Stål, 1854) (Hemiptera: Delphacidae), *Pediculus humanus* (Linnaeus, 1758) (Phthiraptera: Pediculidae), *Apis mellifera* (Linnaeus, 1758) (Hymenoptera, Apidae), *Nasonia vitripennis* (Ashmead, 1904) (Hymenoptera, Pteromalidae), *Tribolium castaneum* (Herbst, 1797) (Coleoptera, Tenebrionidae), *Anopheles gambiae* (Giles, 1902) (Diptera, Culicidae), *Drosophila melanogaster* (Meigen, 1830) (Diptera, Drosophilidae), *Bombyx mori* (Linnaeus, 1758) (Lepidoptera, Bombycidae) and *Danaus plexippus* (Kluk, 1802) (Lepidoptera, Nymphalidae) and two divergent arthropods, *Daphnia pulex* (Müller, 1785) (O. Cladocera, Daphniidae) and *Tetranychus urticae* (C. L. Koch, 1836) (O. Arachnida, Tetranychidae), were used to predict orthologs and to reconstruct the phylogenetic tree. Gene families were identified using *TreeFam* [79,80], and single-copy gene families were assembled to reconstruct phylogenetic relationships. i) Coding sequences of each single-copy family were concatenated to form one super gene group for each species. ii) All of the nucleotides at codon position 2 of these

concatenated genes were extracted to construct the phylogenetic tree by *PhyML* [81], with a gamma distribution across sites and an HKY85 substitution model. iii) The same set of sequences at codon position 2 was used to estimate divergence times among lineages. iv) The fossil calibrations were set with two previous node data [82,83]. v) The *PAML* mcmctree program (v.4.5) [84,85] was used to compute split times using the approximate likelihood calculation algorithm. The software *Tracer* (v.1.5.0) (http://beast.bio.ed.ac.uk/software/tracer/) was utilized to examine the extent of convergence for two independent runs.

Gene family expansion and contraction analysis were performed using the software *CAFE 2.1* (http://sites.bio.indiana.edu/Bhahnlab/Software.html). In CAFE [86], a random birth and death model was used to predict gene gain and loss among gene families across the species-specific phylogenetic tree. Fisher's exact test (Pr0.01) was used to test for over-represented functional categories among the expanded genes and "genomic background" genes.

To detect orthologs evolving under positive selection in *B. tabaci*, an optimized branch-site model [87] was used to search the 1,299 single-copy ortholog genes of the four available species of Hemiptera (*B. tabaci*, *A. pisum*, *N. lugens* and *R. prolixus*). Briefly, these ortholog genes were first aligned using the molecular evolution tool *PRANK* [88], and then ambiguously aligned blocks revealed by *PRANK* alignments and filtered by *Gblocks* [89].

Detoxification enzymes within the putatively expanded cytochrome P450 monooxygenase gene family were identified using a homology-based strategy that used reference genes in *D. melanogaster, A. pisum*, *A. gambiae*, and *A. mellifera* gene models downloaded from (NCBI, http://www.ncbi.nlm.nih.gov/). First, we identified the detoxifying enzyme genes in MED/Q gene models by querying our gene set and scaffolds data with orthologous sequencing using the BLASTx algorithm (*E*-value $\leq 10^{-5}$). The segments with

hits were linked by the Solar software, and parsed using Genewise software for gene predictions to enable the identification of full-length sequences. The resultant sequences were filtered in searches against the non-redundant (nr) and Interpro databases. After filtering false-positive mating sequences, the genes were manually corrected using the MED/Q transcriptome (mainly to P450 and UGT manual annotation), and phylogenetic trees were constructed using MEGA6.0 [90]. A similar method was applied to identify homologous genes in other selected insects.

The immunity related genes in MED/Q were identified by combining the results from motif-based and homology-based strategies, as previously described [91]. This comparison was accomplished by downloading the query sequences available in ImmunoDB [92], and from the NCBI database for the six insects *D. melanogaster*, *A. gambiae*, *A. aegypt*, *A. mellifera*, *C. quinquefasciatus* and *A. pisum*. The motif-based search, MAFFT [93] was used to align multiple protein sequences, and the software HMMER 3.0 was used to build models against which MED/Q sequences were searched using tblastn with hits linked using the Solar software. Genewise software [68] was used to improve the gene predictions and to obtain full-length gene sequences. The resultant sequences were manually edited and merged into a combined dataset of immune-related genes. The immune-related genes of *A. pisum* and *N. lugens* were used for comparisons with whitefly genes obtained using a similar approach.

Metagenomics and analysis of MED/Q endosymbiosis

To improve the *B. tabaci* MED/Q-*associated Hamiltonella* draft genome (AJLH00000000), sequencing data from 16 Illumina read paired-end libraries, ranging from 170 bp to 40 kb (44 lanes), from the MED/Q genome-sequencing project were used. Four sequences were selected as references to filter candidate reads using SOAPaligner (Version: 2.21). These sequences included a previous draft of the *Hamiltonella* genome (372 scaffolds;

AJLH00000000), the pea aphid *Hamiltonella* genome (CP001277), the *Yersinia pestis* CO92 complete genome (AL590842), and the *Serratia plymuthica* AS9 genome (CP002773). The SOAPaligner parameters were "-v 5" for the short insert size library (<1 kb) data and "-v 3 -R" for the large insert size library (>1 kb) data. SOAPdenovo (version 2.04) was used for genome assembly, using the parameters "-u -d 1 -F -K 45" on the above 170 bp to 40 kilo bp data. Gap filling was performed after scaffold construction, and a super-scaffold was also then obtained using the paired-end reads on >500 bp scaffolds to reduce the scaffold number. Then, the Unique Genome Profile (UGP) pipeline was applied to link the scaffolds using BAC sequences from MED/Q. Briefly, 1) the flanking 20 KB sequences of each scaffold were removed, and then unique tags (31-mer) were constructed; 2) the BAC sequences (<150 kb) were BLAST against unique tags; and 3) BACs that had more than two hits were filtered, and then used to construct link relationships to connect larger scaffolds. In addition, the genome of the MED/Q-associated primary endosymbiont *Portiera* was filtered and assembled (as described above) together with four previously reported *Portiera* genome reference sequences obtained from B-type and Q-type *B.tabaci* (GenBank: CP003708, CP003868, CP003867 and CP003835).

Genes were predicted for the finished *Hamiltonella* and *Portiera* genomes using Glimmer v3.02 (protein-coding genes), tRNAscan-SE (tRNAs) and RNAmmer v1.2 (rRNA). The putative coding sequences were annotated using BlastP similarity searches that showed consensus to the NR database (20121005). The E-value cutoff of 1e-5 and a minimum match percentage of 40% were selected for the analysis. Protein domain searches were conducted using InterProScan v4.8, available at the Pfam database, and the resulting coding sequences were used to search the KEGG database (http://www.genome.jp/tools/kaas/).

The amino acid synthesis-related genes in the MED/Q genome were searched against the NR database, under the scenario that they were not of insect origin. The genes identified in this

way were used to construct a phylogenetic tree. To confirm that the HTGs identified were not contaminants associated with rogue bacterial sequences in the libraries, satisfying at least one of the two following conditions was necessary: 1) the HTGs were located on scaffolds that included coding regions homologous to other insects; and 2) the HTGs' transcripts should be present in alignments to a current transcriptome database (after manual corrections) and also as corresponding genes encoded by the genome.

Figures

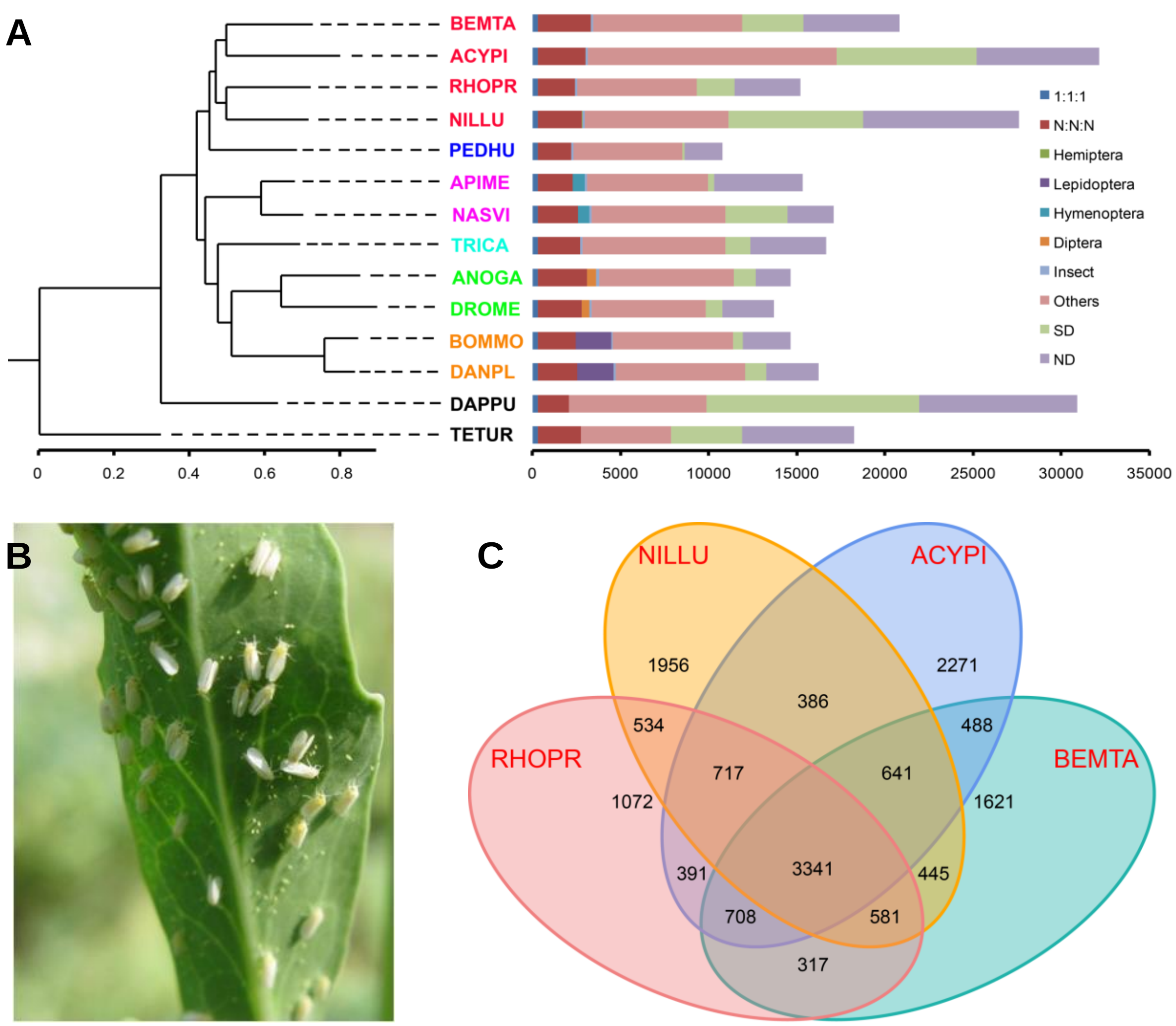

Figure 1

A

	P450	UGT	GST	ABC	COE	Total	
BEMTA*	153	63	21	59	51	347	Phloem feeding
ACYPI*	83	58	32	71	37	281	
NILLU*	65	23	9	57	56	210	
RHOPR	102	15	7	55	49	228	Blood feeding
PEDHU	39	4	12	38	20	113	
ANOGA	115	26	36	59	48	284	
DROME	85	34	32	53	35	239	
APIME	54	12	11	41	29	147	
NASVI	96	23	19	51	46	235	
TRICA*	126	28	30	13	51	248	
BOMMO*	72	33	27	55	89	276	

B

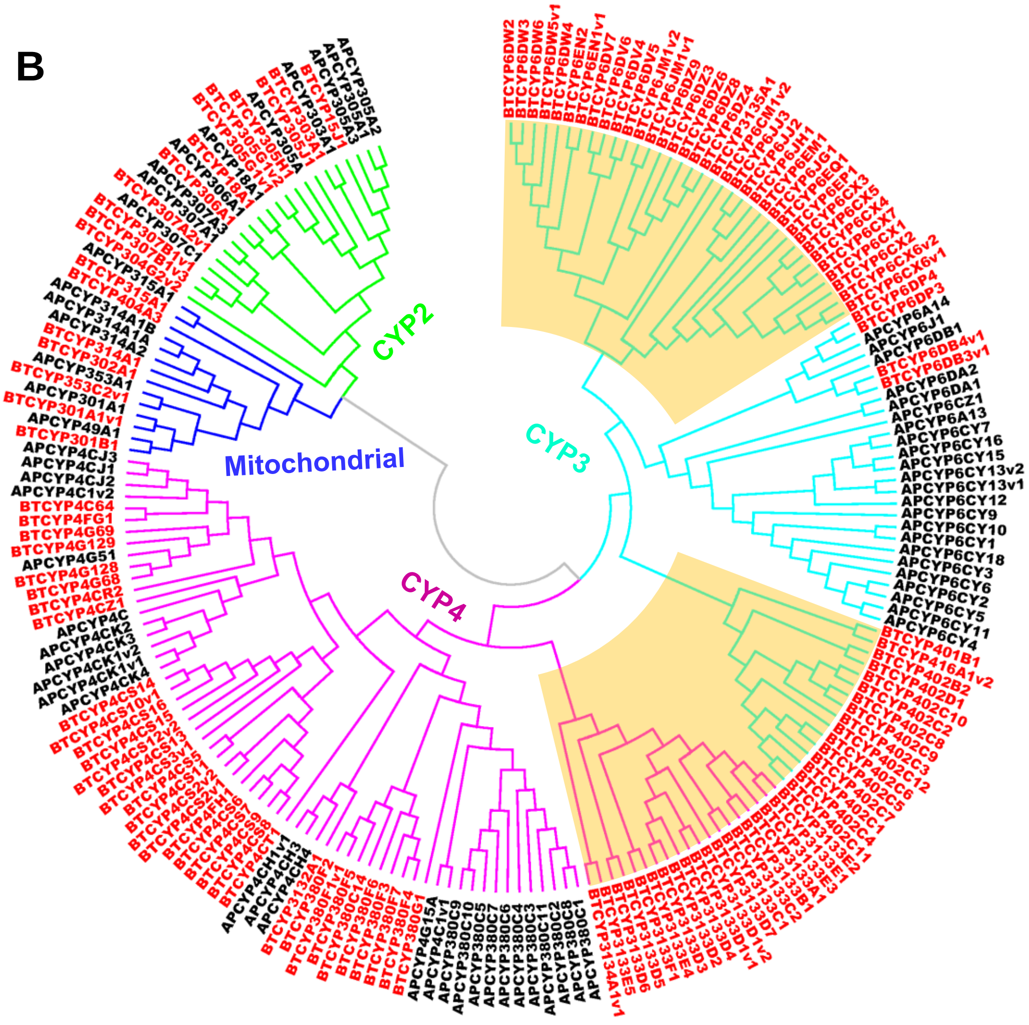

Figure 2

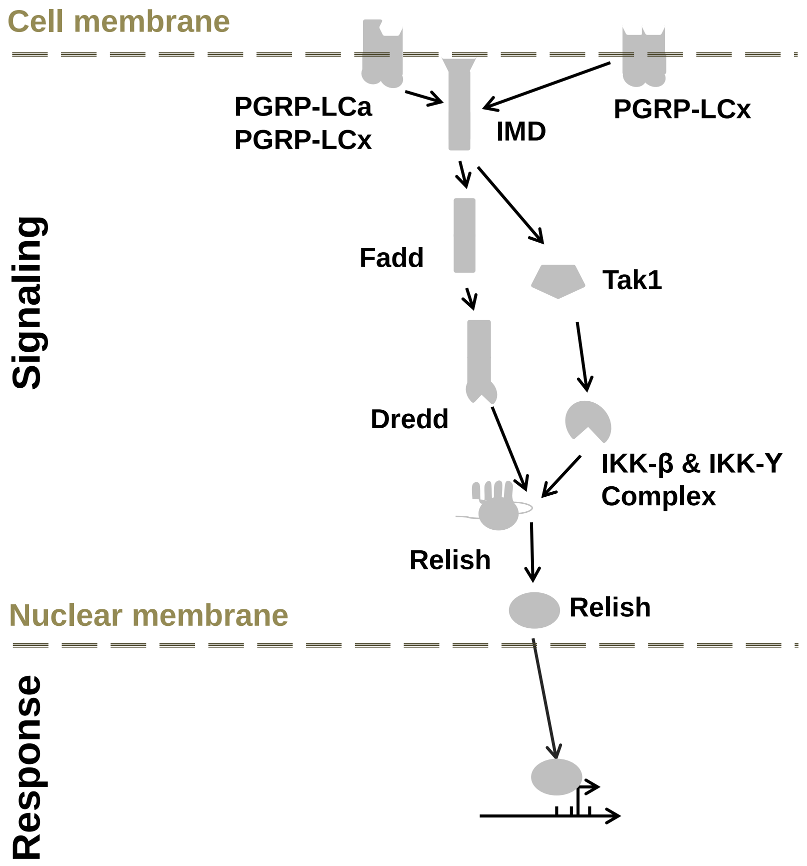

	Bt	Ap	Nl
**IMD**	0	0	1
**Fadd**	0	0	0
**Dredd**	0	0	1
**Tak1**	0	0	1
**Iap2**	0	1	1
**Ubc13**	0	1	1
**Traf**	0	2	2
**Ikk**	0	1	2
**Tab2**	1	1	1
**Relish**	0	0	1

Figure 3

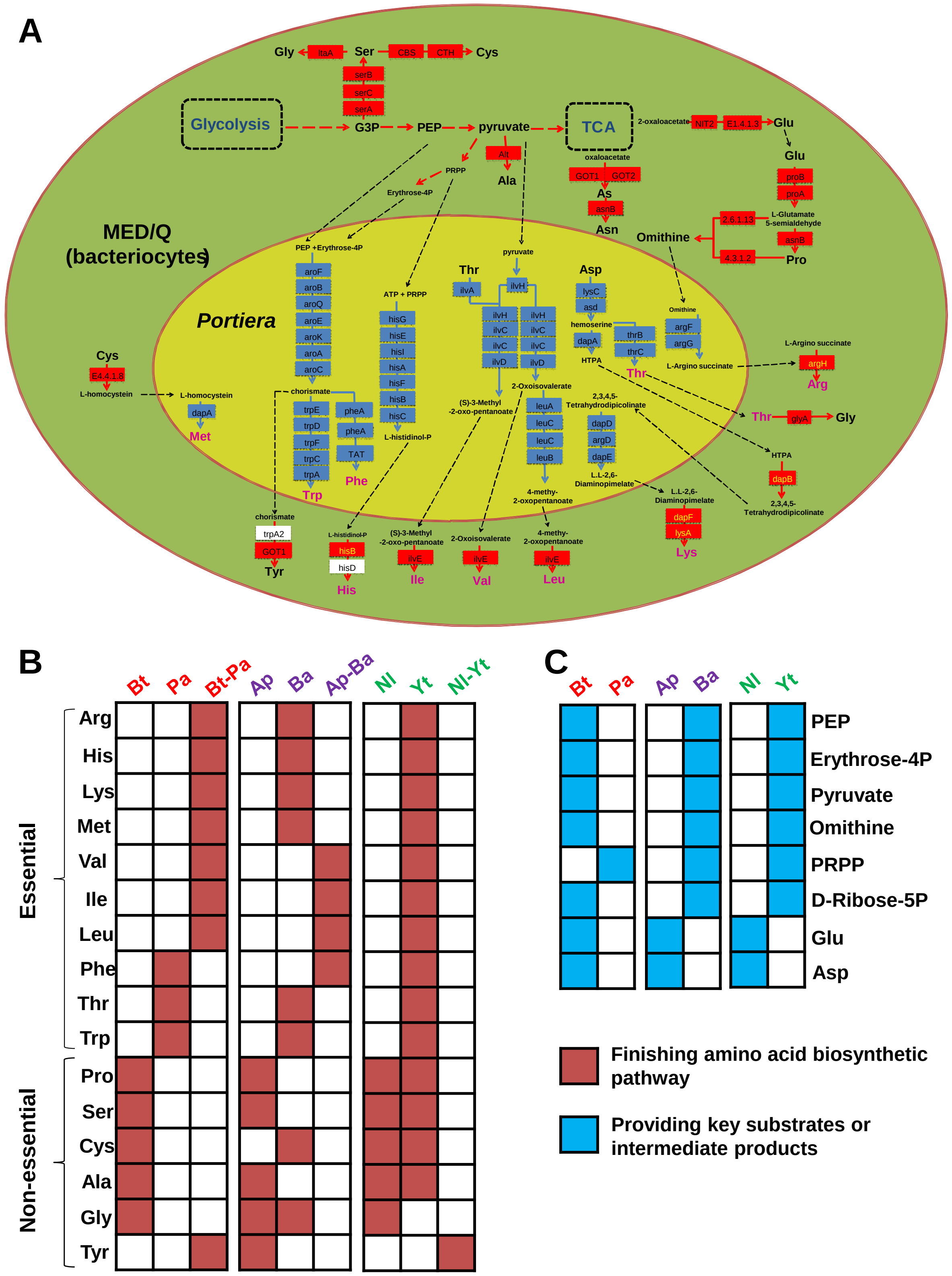
A
Glycolysis
TCA
MED/Q (bacteriocytes)
Portiera
Gly
Ser
Cys
G3P
PEP
pyruvate
Ala
Asn
Glu
Pro
Omithine
Arg
Thr
Lys
Met
Trp
Phe
Tyr
His
Ile
Val
Leu
B
Bt
Pa
Bt-Pa
Ap
Ba
Ap-Ba
Nl
Yt
Nl-Yt
Essential
Non-essential
Arg
His
Lys
Met
Val
Ile
Leu
Phe
Thr
Trp
Pro
Ser
Cys
Ala
Gly
Tyr
C
PEP
Erythrose-4P
Pyruvate
Omithine
PRPP
D-Ribose-5P
Glu
Asp
Finishing amino acid biosynthetic pathway
Providing key substrates or intermediate products

Figure 4

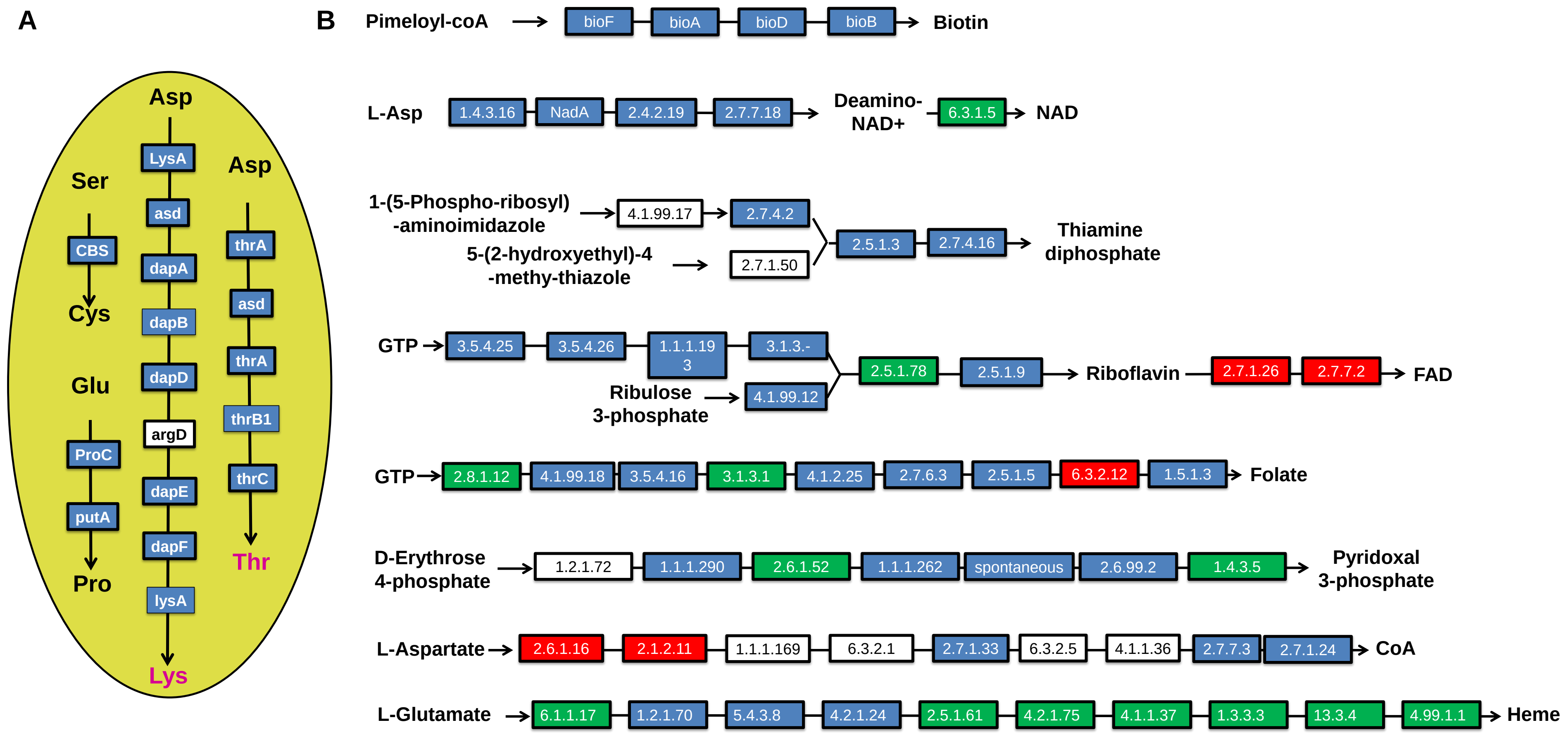
A
Asp
LysA
asd
dapA
dapB
dapD
argD
dapE
dapF
lysA
Lys
Ser
CBS
Cys
Glu
ProC
putA
Pro
Asp
thrA
asd
thrA
thrB1
thrC
Thr
B
Pimeloyl-coA
bioF
bioA
bioD
bioB
Biotin
L-Asp
1.4.3.16
NadA
2.4.2.19
2.7.7.18
Deamino-NAD+
6.3.1.5
NAD
1-(5-Phospho-ribosyl)-aminoimidazole
4.1.99.17
2.7.4.2
5-(2-hydroxyethyl)-4-methy-thiazole
2.7.1.50
2.5.1.3
2.7.4.16
Thiamine diphosphate
GTP
3.5.4.25
3.5.4.26
1.1.1.193
3.1.3.-
Ribulose 3-phosphate
4.1.99.12
2.5.1.78
2.5.1.9
Riboflavin
2.7.1.26
2.7.7.2
FAD
GTP
2.8.1.12
4.1.99.18
3.5.4.16
3.1.3.1
4.1.2.25
2.7.6.3
2.5.1.5
6.3.2.12
1.5.1.3
Folate
D-Erythrose 4-phosphate
1.2.1.72
1.1.1.290
2.6.1.52
1.1.1.262
spontaneous
2.6.99.2
1.4.3.5
Pyridoxal 3-phosphate
L-Aspartate
2.6.1.16
2.1.2.11
1.1.1.169
6.3.2.1
2.7.1.33
6.3.2.5
4.1.1.36
2.7.7.3
2.7.1.24
CoA
L-Glutamate
6.1.1.17
1.2.1.70
5.4.3.8
4.2.1.24
2.5.1.61
4.2.1.75
4.1.1.37
1.3.3.3
13.3.4
4.99.1.1
Heme

Figure 5

Tables

Table 1. Statistics of genome assembly and annotation for MED/Q

Assembled genome size (Mb)	658
Number of scaffolds (>100 bp)	5,003
Total size of assembled scaffolds	658,367,382 bp
N50 (scaffolds)	436,791 bp
Longest scaffold	2,857,362 bp
Number of contigs (>100 bp)	30,873
Total size of assembled contigs	638,748,832 bp
N50 (contigs)	44,388
Longest contig	362,835 bp
GC content	0.38
Number of GLEAN gene models	20,786
Mean transcript length	10,043 bp
Mean coding sequence length	1,504 bp
Mean number of exons per gene	5.12
Mean exon length	294 bp
Mean intron length	1,963 bp
Total size of TEs	265,205,801 bp
TEs proportion of the genome	0.4029

Figure Legend

Figure 1. Phylogenetic relationships and genomic comparisons between *Bemisia tabaci* and other insect species (**A**) Phylogenetic relationships of *B. tabaci* (BEMTA) to insects and other arthropods based on single-copy orthologous genes present in their complete genomes. The following twelve insect species were used for this analysis: *Acyrthosiphon pisum* (ACYPI), *Anopheles gambiae* (ANOGA), *Apis mellifera* (APIME), BEMTA, *Bombyx mori* (BOMMO), *Danaus plexippus* (DANPL), *Drosophila melanogaster* (DROME), *Nasonia vitripennis* (NASVI), *Nilaparvata lugens* (NILLU), *Pediculus humanus* (PEDHU), *Rhodnius prolixus* (RHOPR) and *Tribolium castaneum* (TRICA). The two arthropods *Daphnia pulex* (DAPPU) and *Tetranychus urticae* (TETUR) were used as outgroup taxa. Branch lengths represent divergence times estimated for the second codon position of 308 single-copy genes, using *PhyML* with a gamma distribution across sites and a HKY85 substitution model. The branch supports were inferred based on the approximate likelihood ratio test (aLRT). Gene orthology was determined by comparing the genomes of these 14 arthropod species. The use of 1:1:1 refers to single-copy gene orthologs found across all 14 lineages. The use of N:N:N refers to multi-copy gene paralogs found across the 14 lineages. Diptera, Hemiptera, Hymenoptera, Lepidoptera, and Insecta refer to taxon-specific genes present only in the particular lineage. SD indicates species-specific duplicated genes, and ND indicates species-specific unclustered genes. (**B**) Image of adult MED/Q. (**C**) A Venn diagram showing the orthologous groups shared among the hemipteran genomes of *A. pisum*, *B. tabaci*, *N. lugens* and *R. prolixus*. Based on the analysis, there were 3341 conserved gene families shared by the latter four hemipteran genomes, whereas 2,921 were shared in common by the genomes of the six vascular (blood and phloem) feeders.

Figure 2. Expansion of gene families associated with metabolism and detoxification in MED/Q genome. (**A**) Number of detoxification-related genes in the genomes of eleven selected insects (*denotes herbivorous insects) annotated as UDP glycosyltransferases (UGT), glutathione S-transferase (GST), ATP-binding cassette (ABC) transporter, and, carboxyl/choline esterases (COE). (**B**) Neighbor-joining phylogeny of the cytochrome P450 monooxygenase genes in annotated in the MED/Q genome assembly (red) and orthologs from *A. pisum* (black), with four insect CYP clades indicated as follows: CYP2 (green), CYP3 (blue green), CYP4 (pink), and the mitochondrial clade (blue). Predicted MED/Q-specific expansions within the CYP gene family are indicated (orange boxes).

Figure 3. Predicted orthologues associated with immune deficiency (IMD) within hemipterans. Schematic diagram illustrates the IMD signaling and the corresponding responses. The table shows the number of genes encoding each insect genome; including *B. tabaci* (Bt), *A. pisum* (Ap) and *N. lugens* (Nl).

Figure 4. Comparative analysis of amino-acid biosynthesis and provisioning mechanism among *B. tabaci, A. pisum* and *N. lugens*. (**A**) Unique amino acid biosynthetic and supply mechanisms putatively related to the adaptation of MED/Q. The green and yellow areas represent the bacteriocytes and endosymbiont cells (with respect to the filtered and annotated *Portiera* genome of MED/Q, PRJNA299729), respectively. Essential amino acids are represented in pink, non-essential amino acids in black, and the *Portiera* genes are in blue boxes. The Enzyme Commission numbers (EC) or enzyme names used correspond to those in the Kyoto Encyclopedia of Genes and Genomes (KEGG). MED/Q genes are indicated in red boxes. Black dotted lines represent transport processes between MED/Q and *Portiera*, and red dotted lines represent processes occurring in the bacteriocytes of *Portiera* associated with

MED/Q. Candidate horizontally transferred genes (HTGs) are highlighted in yellow text, and the white boxes with black text represent unidentified genes. (**B**) Comparisons of amino acid biosynthesis in the host-symbiont bacterial systems of *B. tabaci-Portiera*, *A. pisum-Buchnera*, and *N. lugens*-yeast-like organism. Abbreviations: *Bt-Bemisia tabaci*, *Ap-Acyrthosiphon pisum*, *Nl-Nilaparvata lugens*, *Pa-Portiera*, *Ba-Buchnera*, *Yt-yeast-like*. *Bt* (*Ap*, *Nl*, *Pa*, *Ba*, or *Yt*) means it can complete the amino-acid biosynthetic pathway independently. *Bt-Pa* (*Ap-Ba* or *Nl-Yt*) means that both the whitefly host and at least two of its endosymbionts cooperatively complete the amino-acid biosynthetic pathway exhibiting uncannily complex complementarity. (**C**) Comparison of key substrates or intermediate products of the host-endosymbiont systems of *B. tabaci-Portiera*, *A. pisum-Buchnera* and *N. lugens*-yeast-like symbiont, illustrating that phosphoenolpyruvic acid (PEP), erythrose-4P, pyruvate, ornithine and precursor of histidine synthesis (PRPP) are important substrates for amino acid synthesis. Pyruvate and PEP are produced by glycolysis and erythrose-4P by the pentose phosphate pathway. D-ribose-5P is the substrate for PRPP synthesis, and D-ribose-5P was converted based on D-glyceraldehyde 3-phosphate, also a product of glycolysis.

Figure 5. Pathways encoded by *Candidatus hamiltonella* for amino acid and vitamin biosynthesis. (A) The major components of amino acid pathway encoded by *C. hamiltonella*, a facultative endosymbiont of *B. tabaci* (essential amino acids in pink). *Candidatus Hamiltonella* genes are highlighted in blue boxes, with names corresponding to its genome (PRJNA299727). (**B**) Independent and complementation among vitamin synthesis pathways between *B. tabaci* (red box) and *C. hamiltonella* (blue box). White box denotes genes that do not have a match in either genome.

Abbreviations

AMP: anti-microbial peptide; ArK: adaptor protein; MED/Q1: Bemisia tabaci Q1; CEGMA: Core Eukaryotic Genes Mapping Approach; COE: carboxyl/choline esterases; cysLGIC: cys-loop ligand-gated ion channel; dsx: doublesex; EST: Express sequence tag; GABA: γ-aminobutyric acid; GluCls: glutamate-gated chloride channels; GST: glutathione S-transferases; HisCls: histamine-gated chloride channels; HMW: high molecular weight; HTGs: horizontally transferred genes; IMD: immune deficiency; IMP: insulin-like growth factor II mRNA-binding protein; IPTG: Isopropyl-beta-D-thiogalactopyranoside; MED: Mediterranean; mtCOI: mitochondria cytochrome oxidase I; MYA: million years ago; nAChRs: nicotinic acetylcholine receptors; NA-Med-ME: North Africa-Mediterranean-Middle East; OUTs: operational taxonomic units; P450: cytochrome P450 mono-oxygenases; PHCls: pH-sensitive chloride channel; PSI: P-element somatic inhibitor; Sxl: Sex-lethal; TEs: transposable elements; Tra: transformer; Tra2: transformer 2; TYLCV: tomato yellow leaf curl virus; UGTs: UDP glycosyltransferases; WGA: whole-genome amplified; WGS: whole genome shotgun

Acknowledgments and Funding

Authors would like to thank Dr. Paul De Barro for his comments on an earlier draft. This research was supported by the National Natural Science Foundation of China (31420103919 and 31401747), the Beijing Natural Science Foundation (6154034 and 6131002), the Science and Technology Innovation Program of the Chinese Academy of Agricultural Sciences (CAAS-ASTIP-IVFCAAS) and the Beijing Key Laboratory for Pest Control and Sustainable Cultivation of Vegetables. The funders had no role in study design, data collection and analysis, decision to publish, or preparation of the manuscript.

Authors' contributions

YJZ is the leader of the project and the first corresponding author. WX, YJZ, XGZ, YY, JKB and YL were involved in the project design. XGZ, BYX, JYZ, QG, XCL, XQT, MG, HPP, SXR and BLQ coordinated the related research works of the MED/Q genome project. DW performed genome assembly. DW performed protein-coding gene annotation. MC and CHC performed gene orthology and phylogenomics. XY performed insecticide targets annotation. YTL performed putative sex determination genes annotation. WX performed putative phloem specialization genes identification. LTG, LXT, YNW, YZ, QJW, SLW and HYC performed metabolic detoxification systems annotation. ZZY performed immune signaling pathway components annotation. ZZY, JQX, and JQH performed nutrient partitioning between invasive MED/Q and its primary endosymbiont. LTG performed PCR validation. WX, XGZ, DC, JKB, HD, MNM, FG, XPZ, XWW, FHW, YZD, CL, FMY, ELP and XGJ were involved in writing and editing. All authors read and approved the final manuscripts.

Competing interests

The authors declare no competing interests defined by *Giga Science*.

References

1. Brown JK, Frohlich DR, Rosell RC. The sweetpotato or silverleaf whiteflies: biotypes of *Bemisia tabaci* or a species complex? Ann Rev Entomol. 1995; 40:511-534. doi: 10.1146/annurev.en.40.010195.002455.
2. De Barro PJ, Liu SS, Boykin LM, Dinsdale AB. *Bemisia tabaci*: astatement of species status. Ann Rev Entomol. 2011; 56:1-19. doi: 10.1146/annurev-ento-112408-085504.
3. Liu SS, Colvin J, De Barro P. Species concepts as applied to the whitefly *Bemisia tabaci* systematics: how many species are there? J Inter Agric. 2012; 11:176-186. doi: 10.1016/S2095-3119(12)60002-1.
4. Brown JK. Phylogenetic biology of the *Bemisia tabaci* sibling species group. *Bemisia*: Bionomics and Management of a Global Pest. Stansly, P.A. and S. E. Naranjo (eds.), Springer, Dordrecht-Heidelberg-London-New York. 2009; 350pp. p31-67. doi: 10.1007/978-90-481-2460-2_2.
5. Mound LA, Halsey SH. Whitefly of the world. A systematic catalogue of the Aleyrodidae (Homoptera) with host plant and natural enemy data. Chichester: British Museum and John Wiley & Sons, 1978; 340pp John Wiley and Sons.
6. Brown JK, Zerbini FM, Navas-Castillo J, Moriones E, Ramos-Sobrinho R, Silva JC, et al. Revision of *Begomovirus* taxonomy based on pairwise sequence comparisons. Arch Virol. 2015; 160:1593-1619. doi: 10.1007/s00705-015-2398-y.
7. Jones DR. Plant viruses transmitted by whiteflies. Eur J Pl Pathol. 2003; 109:195-219. doi: 10.1023/A:1022846630513.
8. Zhang LP, Zhang YJ, Zhang WJ, Wu QJ, Xu BY, Chu D. Analysis of genetic diversity among different geographical populations and determination of biotypes of *Bemisia tabaci* in China. J Appl Entomol. 2005; 129:121–128. doi: 10.1111/j.1439-0418.2005.00950.x.

9. Pan HP, Preisser EL, Chu D, Wang SL, Wu QJ, Carriere Y, et al. Insecticides promote viral outbreaks by altering herbivore competition. Ecol Appl. 2015; 25:1585-1595. PMID: 26552266.

10. Liu BM, Yan FM, Chu D, Pan HP, Jiao XG, Xie W, et al. Multiple forms of vector manipulation by a plant-infecting virus: *Bemisia tabaci* and tomato yellow leaf curl virus. J Virol. 2013; 87:4929-37. doi:10.1128/JVI.03571-12.

11. Iida H, Kitamura T, Honda K. Comparison of egg-hatching rate, survival rate and development time of the immature stage between B- and Q-biotypes of *Bemisia tabaci* (Gennadius) (Homoptera: Aleyrodidae) on various agricultural crops. Appl Entomol Zool. 2009; 44:267-273. doi: http://doi.org/10.1303/aez.2009.267.

12. Pan HP, Chu D, Yan WQ, Su Q, Liu BM, Wang SL, et al. Rapid spread of tomato yellow leaf curl virus in China is aided differentially by two invasive whiteflies. PLoS One. 2012; 7: e34817. doi:10.1371/journal.pone.0034817.

13. Liu SS, De Barro PJ, Xu J, Luan JB, Zang LS, Ruan YM, et al. Asymmetric mating interactions drive widespread invasion and displacement in a whitefly. Science. 2007; 318:1769-1772. doi: 10.1126/science.1149887.

14. Santos-Garcia D, Farnier PA, Beitia F, Zchori-Fein E, Vavre F, Mouton L, et al. Complete genome sequence of "*Candidatus* Portiera aleyrodidarum" BT-QVLC, an obligate symbiont that supplies amino acids and carotenoids to *Bemisia tabaci*. J Bacteriol. 2012; 194:6654-6655. doi: 10.1128/JB.01793-12

15. Luan JB, Chen W, Hasegawa DK, Simmons AM, Wintermantel WM, Ling KS, et al. Metabolic coevolution in the bacterial symbiosis of whiteflies and related plant sap-feeding insects. Genome Biol Evol. 2015; 7:2635-2647. doi: 10.1093/gbe/evv170.

16. Rao Q, Rollat-Farnier PA, Zhu DT, Santos-Garcia D, Silva FJ, Moya A, et al. Genome reduction and potential metabolic complementation of the dual endosymbionts in the

whitefly *Bemisia tabaci*. BMC Genomics. 2015; 16:226. doi: 10.1186/s12864-015-1379-6.

17. Gottlieb Y, Zchori-Fein E, Mozes-Daube N, Kontsedalov S, Skaljac M, Brumin M, et al. The transmission efficiency of *tomato yellow leaf curl virus* by the whitefly *Bemisia tabaci* is correlated with the presence of a specific symbiotic bacterium species. J Virol. 2010; 84:9310-9317. doi: 10.1128/JVI.00423-10.
18. Chu D, Hu X, Gao C, Zhao H, Nichols RL, Li X. Use of mitochondrial cytochrome oxidase I polymerase chain reaction-restriction fragment length polymorphism for identifying subclades of *Bemisia tabaci* Mediterranean group. J Econ Entomol. 2012; 105:242-251. doi: http://dx.doi.org/10.1603/EC11039.
19. Frohlich DR, Torres-Jerez I I, Bedford ID, Markham PG, Brown JK. A phylogeographical analysis of the *Bemisia tabaci* species complex based on mitochondrial DNA markers. Mol Ecol. 1999; 8:1683-1691. doi: 10.1046/j.1365-294x.1999.00754.x.
20. Guo LT, Wang SL, Wu QJ, Zhou XG, Xie W, Zhang YJ. Flow cytometry and K-mer analysis estimates of the genome sizes of *Bemisia tabaci* B and Q (Hemiptera: Aleyrodidae). Front Physiol. 2015; 6:144. doi: 10.3389/fphys.2015.00144.
21. Jurka J, Kapitonov VV, Pavlicek A, Klonowski P, Kohany O, Walichiewicz J. Repbase Update, a database of eukaryotic repetitive elements. Cytogenet Genome Res. 2005; 110:462-467. doi:10.1159/000084979.
22. Smit AFA, Hubley R, Green P. RepeatMasker. 1999; http://www.repeatmasker.org.
23. Lespinet O, Wolf YI, Koonin EV, Aravind L. The role of lineage-specific gene family expansion in the evolution of eukaryotes. Genome Res. 2002; 12:1048-1059. doi:10.1101/gr.174302.
24. Feyereisen R. Evolution of insect P450. Biochem Soc Trans. 2006; 34:1252-1255. doi:

10.1042/BST0341252.

25. Feyereisen R. 8-Insect CYP genes and P450 enzymes. Insect Mol Biol Biochem. 2012; 236-316. doi: 10.1016/B978-0-12-384747-8.10008-X.

26. Xie W, Wang SL, Wu QJ, Feng YT, Pan HP, Jiao XG, et al. Induction effects of host plants on insecticide susceptibility and detoxification enzymes of *Bemisia tabaci* (Hemiptera: Aleyrodidae). Pest Manag Sci. 2011; 67:87-93. doi: 10.1002/ps.2037.

27. Ffrench-Constant, RH. The molecular genetics of insecticide resistance. Genetics. 2013; 194:807. doi: 10.1534/genetics.112.141895.

28. Force A, Lynch M, Pickett FB, Amores A, Yan YL, Postlethwait J. Preservation of duplicate genes by complementary, degenerative mutations. Genetics. 1999; 151:1531-1545. PMID: 10101175.

29. Cao Z, Yu Y, Wu Y, Hao P, Di Z, He Y, et al. The genome of *Mesobuthus martensii* reveals a unique adaptation model of arthropods. Nat Commun. 2013; 4: 2602. doi: 10.1038/ncomms3602.

30. Zhu F, Moural TW, Shah K, Palli SR. Integrated analysis of cytochrome P450 gene superfamily in the red flour beetle, *Tribolium castaneum*. BMC Genomics. 2013; 14:174. doi: 10.1186/1471-2164-14-174.

31. Yang Y, Chen S, Wu S, Yue L, Wu Y. Constitutive overexpression of multiple cytochrome P450 genes associated with pyrethroid resistance in *Helicoverpa armigera*. J Econ Entomol. 2006; 99:1784-1789. PMID: 17066813.

32. Yamamoto K, Ichinose H, Aso Y, Fujii H. Expression analysis of cytochrome P450s in the silkmoth, *Bombyx mori*. Pestic Biochem Phys. 2010; 97:1-6. doi:10.1016/j.pestbp.2009.11.006.

33. Yoon KS, Strycharz JP, Baek JH, Sun W, Kim JH, Kang JS, et al. Brief exposures of human body lice to sublethal amounts of ivermectin over-transcribes detoxification genes

involved in tolerance. Insect Mol Biol. 2011; 20:687-699. doi: 10.1111/j.1365-2583.2011.01097.x.

34. Pridgeon JW, Zhang L, Liu N. Overexpression of CY4G19 associated with a pyrethroid-resistant strain of the German cockroach, *Blattella germanica* (L.). Gene. 2003; 314:157-163. PMID: 14527728.

35. Scharf ME, Parimi S, Meinke LJ, Chandler LD, Siegfried BD. Expression and induction of three family 4 cytochrome P450 (CYP4) genes identified from insecticide-resistant and susceptible western corn rootworms, *Diabrotica virgifera virgifera*. Insect Mol Biol. 2001; 10:139-146. PMID:11422509.

36. Pan H, Chu D, Ge D, Wang S, Wu Q, Xie W, et al. Further spread of and domination by *Bemisia tabaci* (Hemiptera: Aleyrodidae) biotype Q on field crops in China. J Econ Entomol. 2011; 104:978-985. doi: http://dx.doi.org/10.1603/EC11009.

37. Karunker I, Benting J, Lueke B, Ponge T, Nauen R, Roditakis E, et al. Over-expression of cytochrome P450 CYP6CM1 is associated with high resistance to imidacloprid in the B and Q biotypes of *Bemisia tabaci* (Hemiptera: Aleyrodidae). Insect Biochem Mol Biol. 2008; 38:634-644. doi:10.1016/j.ibmb.2008.03.008.

38. Guo L, Xie W, Wang S, Wu Q, Li R, Yang N, et al. Detoxification enzymes of *Bemisia tabaci* B and Q: biochemical characteristics and gene expression profiles. Pest Manag Sci. 2014; 70:1588-1594. doi: 10.1002/ps.3751.

39. Bozzolan F, Siaussat D, Maria A, Durand N, Pottier MA, Chertemps T, et al. Antennal uridine diphosphate (UDP)-glycosyltransferases in a pest insect: diversity and putative function in odorant and xenobiotics clearance. Insect Mol Biol. 2014; 23:539-549. doi: 10.1111/imb.12100.

40. Luque T, O'Reilly DR. Functional and phylogenetic analyses of a putative *Drosophila melanogaster* UDP-glycosyltransferase gene. Insect Biochem Mol Biol. 2002; 32:1597-

1604. doi:10.1016/S0965-1748(02)00080-2.

41. Sasai H, Ishida M, Murakami K, Tadokoro N, Ishihara A, Nishida R, et al. Species-specific glucosylation of DIMBOA in larvae of the rice armyworm. Biosci Biotech Bioch. 2009; 73:1333-1338. doi: 10.1271/bbb.80903.

42. Kliot A, Kontsedalov S, Ramsey JS, Jander G, Ghanim M. Adaptation to nicotine in the facultative tobacco - feeding hemipteran *Bemisia tabaci*. Pest Manag Sci. 2014; 70:1595-1603. doi: 10.1002/ps.3739.

43. Urban JM, Cryan JR. Two ancient bacterial endosymbionts have coevolved with the planthoppers (Insecta: Hemiptera: Fulgoroidea). BMC Evol Biol. 2012; 12:87. doi: 10.1186/1471-2148-12-87.

44. Kuechler SM, Gibbs G, Burckhardt D, Dettner K, Hartung V. Diversity of bacterial endosymbionts and bacteria–host co - evolution in Gondwanan relict moss bugs (Hemiptera: Coleorrhyncha: Peloridiidae). Environ Microbiol. 2013; 15:2031-2042. doi: 10.1111/1462-2920.12101.

45. Wilson AC, Duncan RP. Signatures of host/symbiont genome coevolution in insect nutritional endosymbioses. Proc Natl Acad Sci U S A. 2015; 112:10255-10261. doi: 10.1073/pnas.1423305112.

46. Thao ML, Baumann P. Evolutionary relationships of primary prokaryotic endosymbionts of whiteflies and their hosts. Appl Environ Microbiol. 2004; 70:3401-3406. PMID: 15184137.

47. Zchori-Fein E, Brown JK. Diversity of prokaryotes associated with *Bemisia tabaci* (Gennadius) (Hemiptera: Aleyrodidae). Ann Entomol Soc Am. 2002; 95:711-718. doi: http://dx.doi.org/10.1603/0013-8746(2002)095[0711:DOPAWB]2.0.CO;2.

48. Ahmed MZ, Ren S, Xue X, Li XX, Jin G, Qiu BL. Prevalence of endosymbionts in Bemisia tabaci populations and their in vivo sensitivity to antibiotics. Curr Microbiol.

2010; 61:322-328. doi: 10.1007/s00284-010-9614-5.

49. Gueguen G, Vavre F, Gnankine O, Peterschmitt M, Charif D, Chiel E, et al. Endosymbiont metacommunities, mtDNA diversity and the evolution of the *Bemisia tabaci* (Hemiptera: Aleyrodidae) species complex. Mol Ecol. 2010; 19: 4365-4378. doi: 10.1111/j.1365-294X.2010.04775.x.

50. Bing XL, Yang J, Zchori-Fein E, Wang XW, Liu SS. Characterization of a newly discovered symbiont of the whitefly *Bemisia tabaci* (Hemiptera: Aleyrodidae). Appl Environ Microb. 2013; 79:569-575. doi: 10.1128/AEM.03030-12.

51. Santos-Garcia D, Vargas-Chavez C, Moya A, Latorre A, Silva FJ. Genome evolution in the primary endosymbiont of whiteflies sheds light on their divergence. Genome Biol Evol. 2015; 7:873-888. doi: 10.1093/gbe/evv038.

52. Moran NA, Jarvik T. Lateral transfer of genes from fungi underlies carotenoid production in aphids. Science. 2010; 328:624-627. doi: 10.1126/science.1187113.

53. Husnik F, Nikoh N, Koga R, Ross L, Duncan RP, Fujie F, et al. Horizontal gene transfer from diverse bacteria to an insect genome enables a tripartite nested mealybug symbiosis. Cell. 2013; 153:1567-1578. doi:10.1016/j.cell.2013.05.040.

54. De Gregorio E, Spellman PT, Tzou P, Rubin GM, Lemaitre B. The Toll and Imd pathways are the major regulators of the immune response in *Drosophila*. EMBO J. 2002; 21:2568-2579. doi: 10.1093/emboj/21.11.2568.

55. Munson MA, Baumann P, Kinsey MG. *Buchnera gen. nov.* and *Buchnera aphidicola sp. nov.*, a taxon consisting of the mycetocyte-associated, primary endosymbionts of aphids. Int J Syst Bacteriol. 1991; 41:566-568. doi: 10.1099/00207713-41-4-566.

56. Gerardo NM, Altincicek B, Anselme C, Atamian H, Barribeau SM, de Vos M, et al. Immunity and other defenses in pea aphids, *Acyrthosiphon pisum*. Genome Biol. 2010; 11:R21. doi: 10.1186/gb-2010-11-2-r21.

57. Misof B, Liu S, Meusemann K, Peters RS, Donath A, Mayer C, et al. Phylogenomics resolves the timing and pattern of insect evolution. Science. 2014; 346:763-767. doi: 10.1126/science.1257570.

58. Chu D, Zhang YJ, Brown JK, Cong B, Xu BY, Wu QJ, et al. The introduction of the exotic Q biotype of *Bemisia tabaci* from the Mediterranean region into China on ornamental crops. Fla Entomol. 2006; 89:168-174. doi: http://dx.doi.org/10.1653/0015-4040(2006)89[168:TIOTEQ]2.0.CO;2.

59. Tao Q, Wang A, Zhang HB. One large-insert plant-transformation-competent BIBAC library and three BAC libraries of Japonica rice forgenome research in rice and other grasses. Theor Appl Genet. 2002; 105:1058-1066. doi: 10.1007/s00122-002-1057-3.

60. Li R, Fan W, Tian G, Zhu H, He L, Cai J, et al. The sequence and de novo assembly of the giant panda genome. Nature. 2010; 463:311-317. doi:10.1038/nature08696.

61. You M, Yue Z, He W, Yang X, Yang G, Xie M, et al. A heterozygous moth genome provides insights into herbivory and detoxification. Nat Genet. 2013; 45:220-225. doi:10.1038/ng.2524.

62. Boetzer M, Henkel CV, Jansen HJ, Butler D, Pirovano W. Scaffolding pre-assembled contigs using SSPACE. Bioinformatics. 2011; 27:578-579. doi:10.1093/bioinformatics/btq683.

63. Benson G. Tandem repeats finder: a program to analyze DNA sequences. Nucleic Acids Res. 1999; 27:573-580. doi: 10.1093/nar/27.2.573.

64. Edgar RC, Myers EW. PILER: identification and classification of genomic repeats. Bioinformatics. 2005; 21:152-158. doi:10.1093/bioinformatics/bti1003.

65. Price AL, Jones NC, Pevzner PA. De novo identification of repeat families in large genomes. Bioinformatics. 2005; 21:351-358. doi:10.1093/bioinformatics/bti1018.

66. Xu Z, Wang H. LTR_FINDER: an efficient tool for the prediction of full-length LTR

retrotransposons. Nucleic Acids Res. 2007; 35:265-268. doi: 10.1093/nar/gkm286.

67. Parra G, Bradnam K, Ning Z, Keane T, Korf I. Assessing the gene space in draft genomes. Nucleic Acids Res. 2009; 37:289-297. doi: 10.1093/nar/gkn916.
68. Birney E, Clamp M, Durbin R. GeneWise and Genomewise. Genome Res. 2004; 14:988-995. doi:10.1101/gr.1865504.
69. Burge C, Karlin S. Prediction of complete gene structures in human genomic DNA. J Mol Biol. 1997; 268:78-94. doi:10.1006/jmbi.1997.0951.
70. Stanke M, Keller O, Gunduz I, Hayes A, Waack S, Morgenstern B. AUGUSTUS: ab initio prediction of alternative transcripts. Nucleic Acids Res. 2006; 34**:** W435-W439. PMID: 16845043.
71. Wang XW, Luan JB, Li JM, Bao YY, Zhang CX, Liu SS. De novo characterization of a whitefly transcriptome and analysis of its gene expression during development. BMC genomics. 2010; 11:400. doi: 10.1186/1471-2164-11-400.
72. Ye XD, Su YL, Zhao QY, Xia WQ, Liu SS Wang XW. Transcriptomic analyses reveal the adaptive features and biological differences of guts from two invasive whitefly species. BMC genomics. 2014; 15:370. doi: 10.1186/1471-2164-15-370.
73. Su YL, Li JM, Li M, Luan JB, Ye XD, Wang XW, et al. Transcriptomic analysis of the salivary glands of an invasive whitefly. PLoS One. 2012; 7:e39303. doi:10.1371/journal.pone.0039303.
74. Elsik CG, Mackey AJ, Reese JT, Milshina NV, Roos DS, Weinstock GM. Creating a honeybee consensus gene set. Genome Biol. 2007; 8:R13. doi: 10.1186/gb-2007-8-1-r13.
75. Trapnell C, Williams BA, Pertea G, Mortazavi A, Kwan G, van Baren MJ, et al. Transcript assembly and quantification by RNA-Seq reveals unannotated transcripts and isoform switching during cell differentiation. Nat Biotechnol. 2010; 28:511-515. doi: 10.1038/nbt.1621.

76. Kanehisa M, Goto S. KEGG: kyoto encyclopedia of genes and genomes. Nucleic Acids Res. 2000; 28:27-30. doi: 10.1093/nar/28.1.27.

77. Bairoch A, Apweiler R. The SWISS-PROT protein sequence database and its supplement TrEMBL in 2000. Nucleic Acids Res. 2000; 28:45-48. doi: 10.1093/nar/28.1.45.

78. Zdobnov EM, Apweiler R. InterProScan--an integration platform for the signature-recognition methods in InterPro. Bioinformatics. 2001; 17:847-848. doi:10.1093/bioinformatics/17.9.847.

79. Li H, Coghlan A, Ruan J, Coin LJ, Hériché JK, Osmotherly L, et al. TreeFam: a curated database of phylogenetic trees of animal gene families. Nucleic Acids Res. 2006; 34: 572-580. doi: 10.1093/nar/gkj118.

80. Ruan J, Li H, Chen Z, Coghlan A, Coin LJ, Guo Y, et al. TreeFam: 2008 update. Nucleic Acids Res. 2008; 36:735-740. doi: 10.1093/nar/gkm1005.

81. Guindon S, Dufayard JF, Lefort V, Anisimova M, Hordijk W, Gascuel O. New algorithms and methods to estimate maximum-likelihood phylogenies: assessing the performance of PhyML 3.0. Syst Biol. 2010; 59:307-321. doi: 10.1093/sysbio/syq010.

82. Benton MJ, Donoghue PC. Paleontological evidence to date the tree of life. Mol Biol Evol. 2007; 24:26-53. doi: 10.1093/molbev/msl150.

83. Donoghue PCJ, Benton MJ. Rocks and clocks: calibrating the Tree of Life using fossils and molecules. Trends Ecol Evol. 2007; 22:424-431. doi: 10.1016/j.tree.2007.05.005.

84. Yang Z. PAML: a program package for phylogenetic analyses by maximum likelihood. Comp Appl BioSci. 1997; 13:555-556. doi: 10.1099/0022-1317-79-8-1951.

85. Yang Z. PAML 4: phylogenetic analysis by maximum likelihood. Mol Biol Evol. 2007; 24:1586-1591. doi: 10.1093/molbev/msm088.

86. De Bie T, Cristianini N, Demuth JP, Hahn MW. CAFE: a computational tool for the study of gene family evolution. Bioinformatics. 2006; 22:1269-1271.

doi:10.1093/bioinformatics/btl097.

87. Zhang J, Nielsen R, Yang Z. Evaluation of an improved branch-site likelihood method for detecting positive selection at the molecular level. Mol Biol Evol. 2005; 22:2472-2479. doi: 10.1093/molbev/msi237.
88. Loytynoja A, Goldman N. Phylogeny-aware gap placement prevents errors in sequence alignment and evolutionary analysis. Science. 2008; 320:1632-1635. doi: 10.1126/science.1158395.
89. Talavera G, Castresana J. Improvement of phylogenies after removing divergent and ambiguously aligned blocks from protein sequence alignments. Syst Biol. 2007; 56:564-577. doi: 10.1080/10635150701472164.
90. Tamura K, Stecher G, Peterson D, Filipski A, Kumar S. MEGA6: molecular evolutionary genetics analysis version 6.0. Mol Biol Evol. 2013; 30:2725-2729. doi: 10.1093/molbev/mst197.
91. Cao X, He Y, Hu Y, Wang Y, Chen YR, Bryant B, et al. The immune signaling pathways of *Manduca sexta*. Insect Biochem Mol Biol. 2015; 62:64-74. doi:10.1016/j.ibmb.2015.03.006.
92. Waterhouse RM, Kriventseva EV, Meister S, Xi Z, Alvarez KS, Bartholomay LC, et al. Evolutionary dynamics of immune-related genes and pathways in disease-vector mosquitoes. Science. 2007; 316:1738-1743. doi: 10.1126/science.1139862.
93. Katoh K, Standley DM. MAFFT multiple sequence alignment software version 7. improvements in performance and usability. Mol Biol Evol. 2013; 30:772-80. doi: 10.1093/molbev/mst010.

Additional files

Supporting Figures

Figure S1. Schematic illustration of the assembly pipeline for *B. tabaci* Q genome based on the combined assemblies from WGS and BACs.

Figure S2. Gene family expansion and contraction in *B. tabaci* Q genome compared to other arthropods.

Figure S3. Phylogenetic trees for 11 horizontally transferred genes (HGTs).

Figure S4. Estimated divergence times among insect genomes using PAML *mcmctree*.

Supporting Tables

Table S1. Statistics of whole genome sequencing data

Table S2. Repeat Masker analysis of repeats in 4 hemiptera species

Table S3. Evidenced use within GLEAN MED/Q protein-coding genes

Table S4. Summary of evidence for the GLEAN gene models

Table S5. Functional annotation of the MED/Q genome

Table S6. Gene orthology comparison among the genomes of 14 arthropod species

Table S7. Gene ontologies for gene families that have expanded number of members on *Bemisia tabaci* branch (FDR<0.05, p<=0.00097087378641)

Table S8. Results of gene family expansion (gene gain) and contraction (gene loss) analysis

Table S9. Gene ontology over-representation of gene families contracted on *Bemisia tabaci* branch (FDR<0.05, p<=0.000572390572)

Table S10. Immune system-related and virus transport gene number in *Bemisia tabaci*, *Nilaparvata lugens*, *Acyrthosiphum pisum*, and comparable with three blood-feeding insects (*Rhodnius prolixus*, *Pediculus humanus* and *Anopheles gambiae*)

Table S11. List of genes involved in B vitamin biosynthesis in MED/Q

Table S12. List of genes involved in B vitamin biosynthesis in *Candidatus* Hamiltonella defense

Table S13. List of horizontally transferred genes involved in amino acid biosynthesis in MED/Q

Table S14. Comparison of transaminase number in three symbiotic systems *Bemisia tabaci*/Portiera, *Acyrthosiphum pisum*/Buchnera and *Nilaparvata lugens*/Yeast-like

Figure 1 Click here to download Figure Fig1.tif

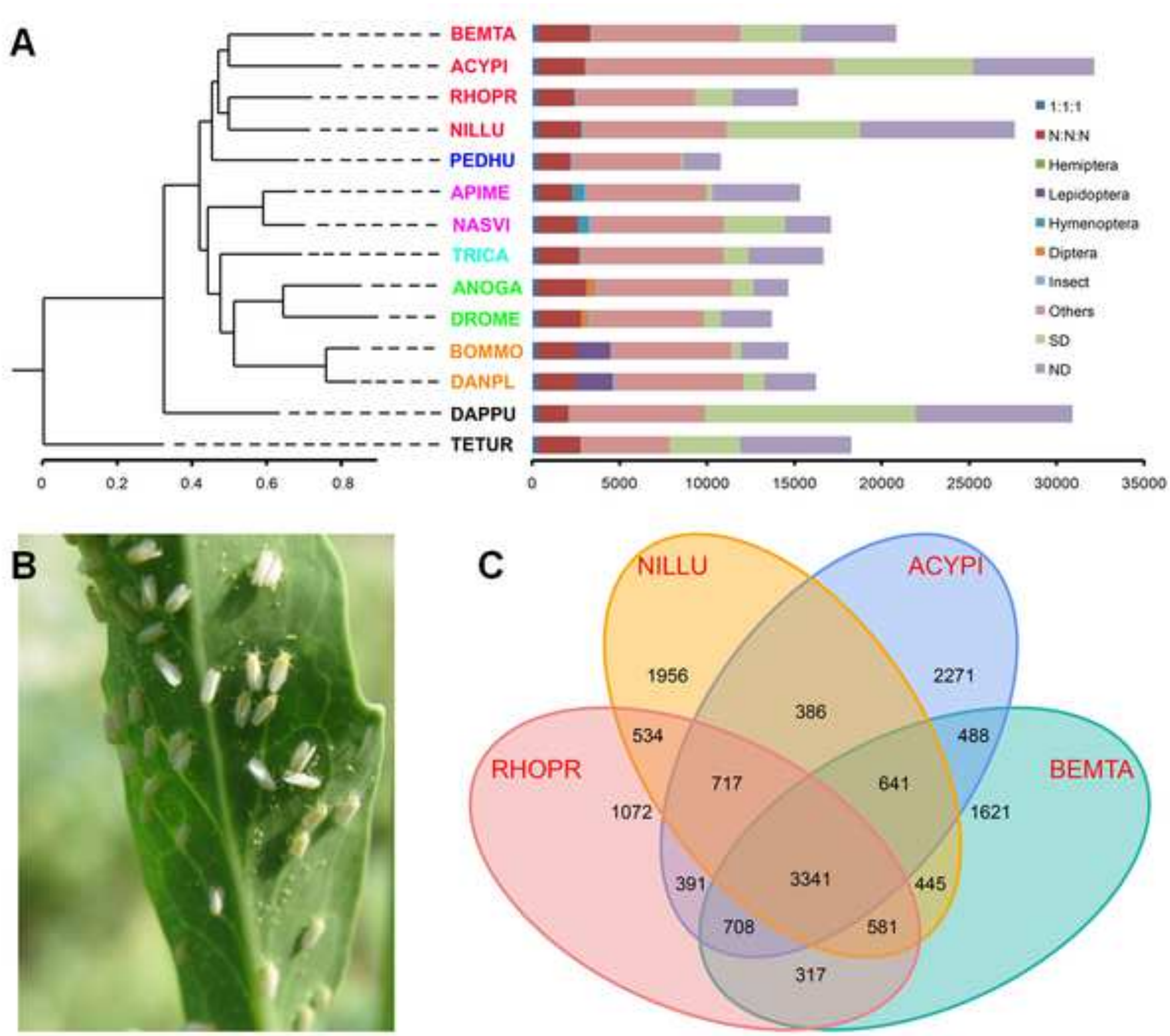

Figure 2

Click here to download Figure Fig2.tif

A

	P450	UGT	GST	ABC	COE	Total	
BEMTA*	153	63	21	59	51	347	Phloem feeding
ACYPI*	83	58	32	71	37	281	Phloem feeding
NILLU*	65	23	9	57	56	210	Phloem feeding
RHOPR	102	15	7	55	49	228	Blood feeding
PEDHU	39	4	12	38	20	113	Blood feeding
ANOGA	115	26	36	59	48	284	Blood feeding
DROME	85	34	32	53	35	239	
APIME	54	12	11	41	29	147	
NASVI	96	23	19	51	46	235	
TRICA*	126	28	30	13	51	248	
BOMMO*	72	33	27	55	89	276	

B

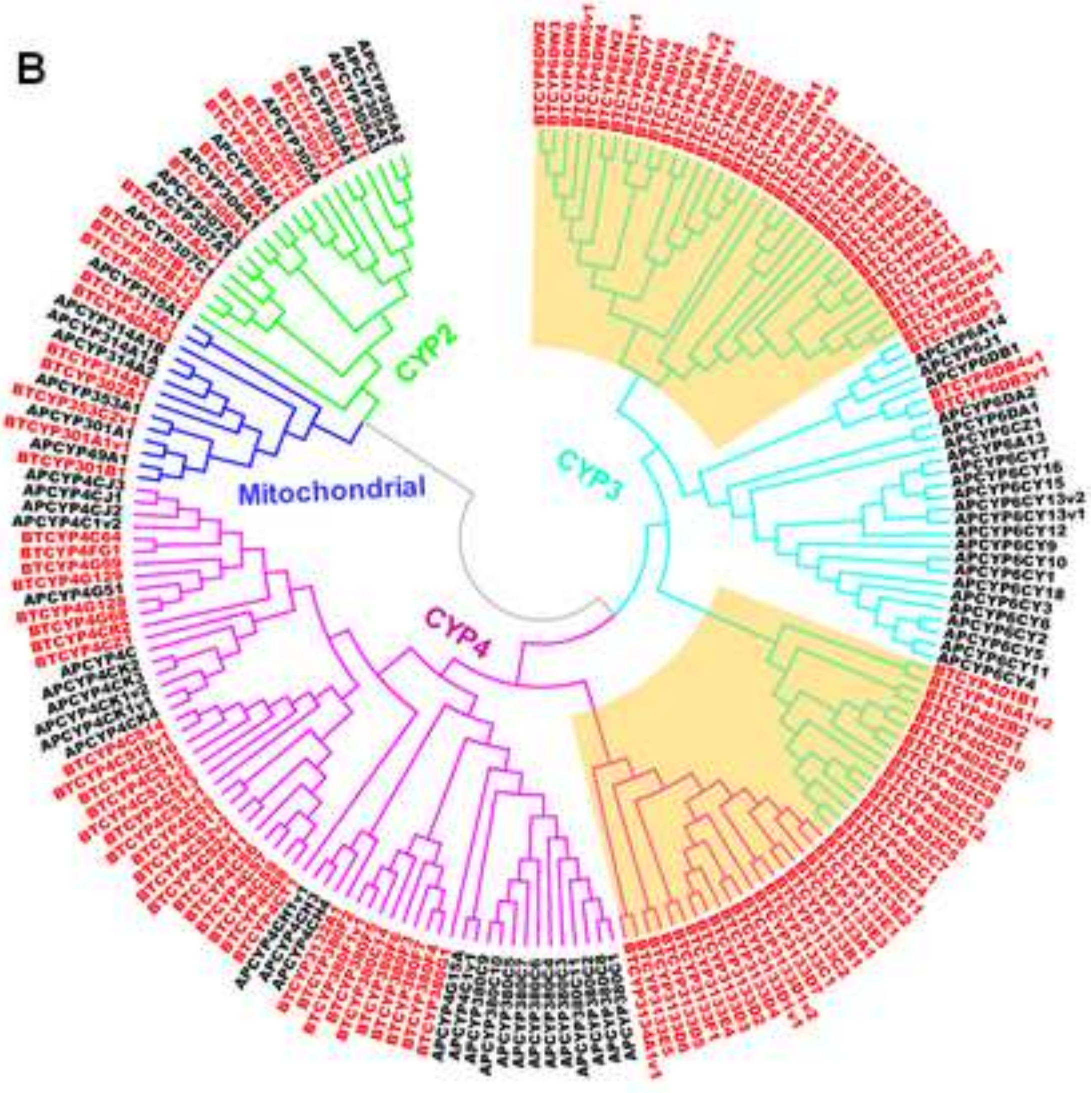

 Click here to download Figure Fig3.tif

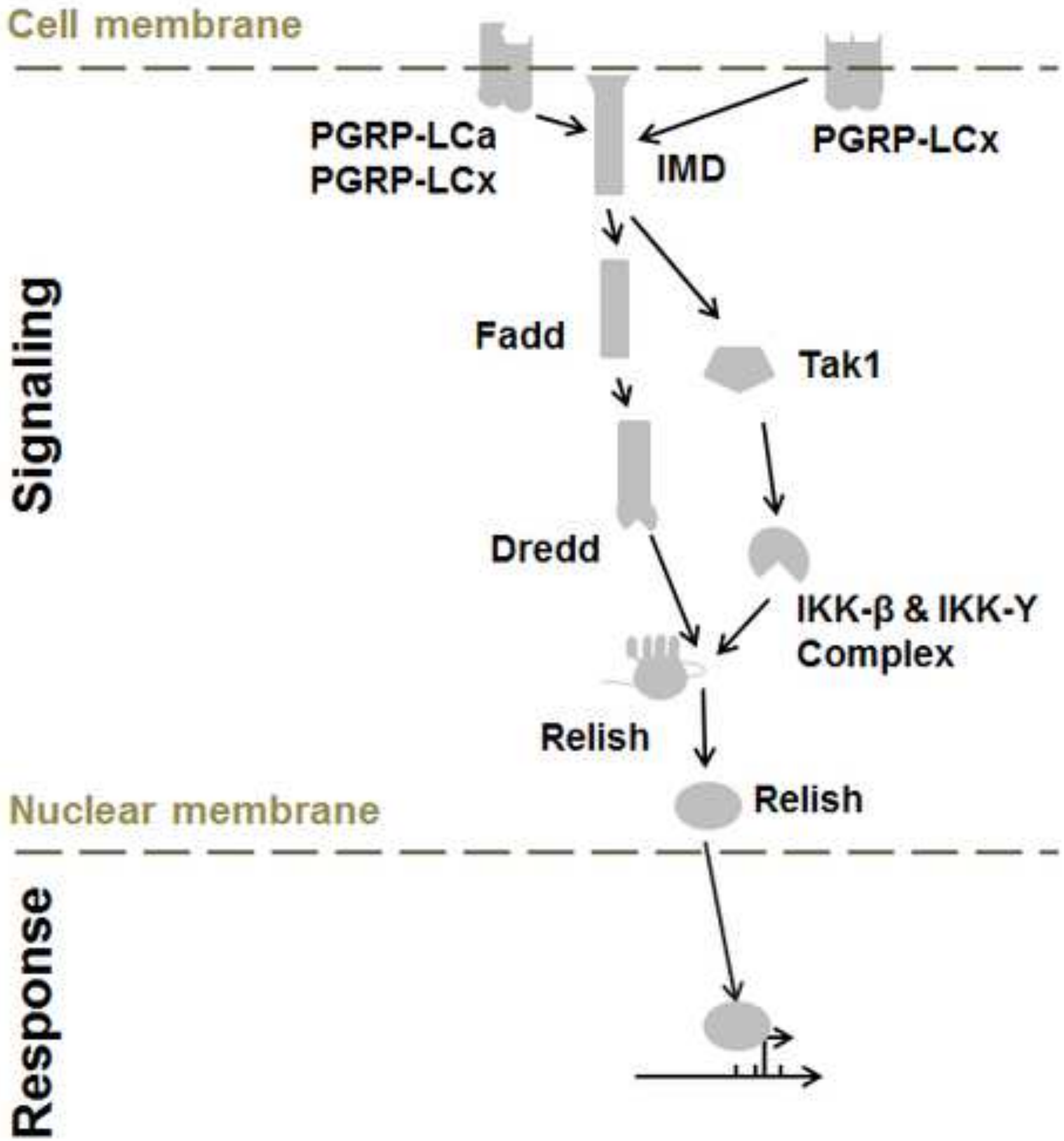

	Bt	Ap	NI
IMD	0	0	1
Fadd	0	0	0
Dredd	0	0	1
Tak1	0	0	1
Iap2	0	1	1
Ubc13	0	1	1
Traf	0	2	2
Ikk	0	1	2
Tab2	1	1	1
Relish	0	0	1

Figure 4 Click here to download Figure Fig4.tif

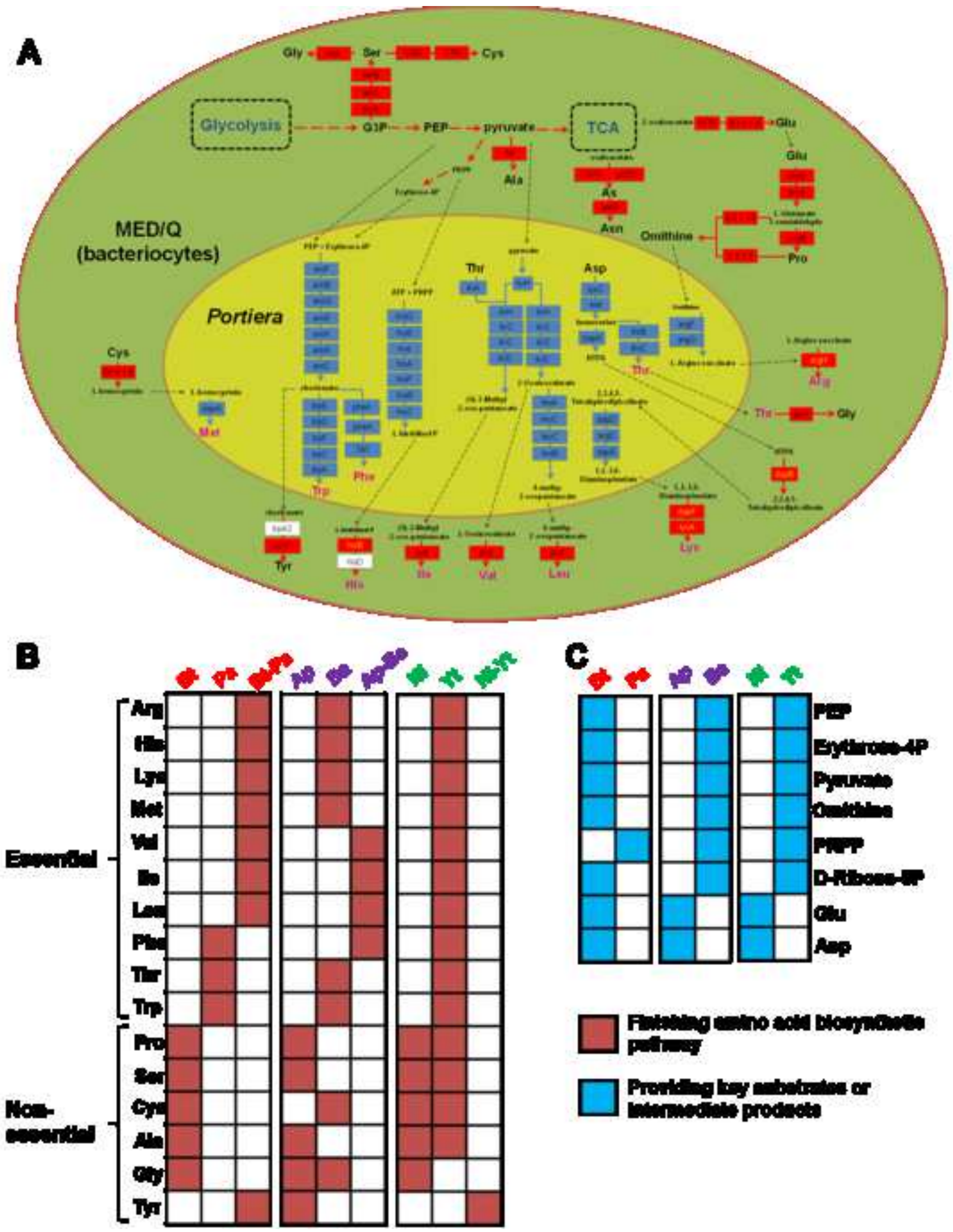

Figure 5

Click here to download Figure Fig5.tif

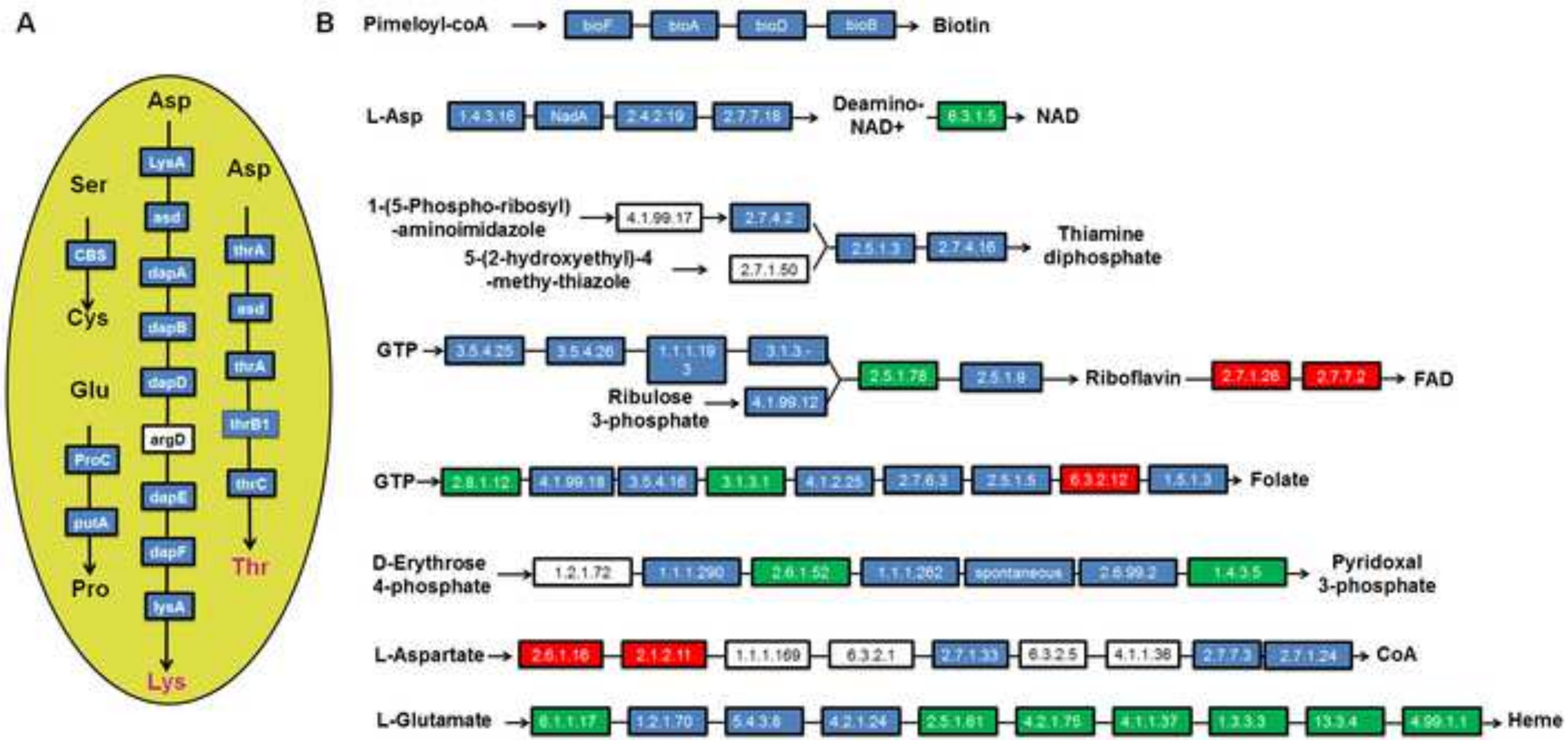

Fig S1

Click here to access/download
Supplementary Material
Figure S1.docx

Fig S2

Click here to access/download
Supplementary Material
Figure S2.docx

Fig S3

Click here to access/download
Supplementary Material
Figure S3.docx

Fig S4

Click here to access/download
Supplementary Material
Figure S4.docx

Table S1

Click here to access/download
Supplementary Material
S1_Table.docx

Table S2

Click here to access/download
Supplementary Material
S2_Table.docx

Table S3

Click here to access/download
Supplementary Material
S3_Table.docx

Table S4

Click here to access/download
Supplementary Material
S4_Table.docx

Table S5

Click here to access/download
Supplementary Material
S5_Table.docx

Table S6

Click here to access/download
Supplementary Material
S6_Table.docx

Table S7

Click here to access/download
Supplementary Material
S7_Table.docx

Table S8

Click here to access/download
Supplementary Material
S8_Table.docx

Table S9

Click here to access/download
Supplementary Material
S9_Table.docx

Table S10

Click here to access/download
Supplementary Material
S10_Table.docx

Table S11

Click here to access/download
Supplementary Material
S11_Table.docx

Table S12

Click here to access/download
Supplementary Material
S12_Table.docx

Table S13

Click here to access/download
Supplementary Material
S13_Table.docx

Table S14

Click here to access/download
Supplementary Material
S14_Table.docx

Personal Cover Click here to download Personal Cover Cover Letter_Wen et al_Zhou_7-25-16_V2.docx

UNIVERSITY OF KENTUCKY

College of Agriculture
Department of Entomology
Office of the State Entomologist
S-225 Agriculture Science Center-N
Lexington, KY 40546-0091

Editorial Board
GigaScience

July 25, 2016

Letter of Submission

Dear Editor,

Please consider the accompanying manuscript, "**The invasive Q-type *Bemisia tabaci* genome: a tale of gene loss and gene gain**" by Wen XIe *et al* for publication as a research article in *GigaScience*.

Invasive whitefly, *Bemisia tabaci*, is a highly destructive agricultural and ornamental crop pest. As a group, *B. tabaci* damages host plants through phloem feeding and vectoring plant pathogens. Introductions of *B. tabaci* are difficult to quarantine and eradicate due to high reproductive rates, broad host plant range, and resistance to chemical insecticides. A 658 Mb draft genome for the Q-type *B. tabaci* (MED/Q) assembled and annotated with 20,786 protein-coding genes. Metabolic pathways show an expansion in the number of gene family members, in particular, the cytochrome P450 monooxygenases. Additionally, amino acid biosynthesis pathways are partitioning among host and endosymbiont genomes in a manner that is distinct from other hemipteran systems, wherein evidence of horizontal gene transfer to the host genome likely form the basis of obligatory relationships. Putative loss of function of the immune deficiency (IMD) signaling pathway due to gene loss is a shared ancestral trait of hemipteran insects that show competency for hosting endosymbiotic bacteria. This expansion of P450 gene family member may influence the well-noted capacity of MED/Q to adapt to repeated exposures to chemical insecticides, and furthermore, be related to invasiveness in monoculture cropping systems where such applications are prevalent.

This sequencing project was a collaborative effort among a consortium of international whitefly researchers. The researchers collectively decided that given the many resources already developed, the global invasion status and the large number of scientists studying them, the Q-type *B. tabaci* (MED/Q) would be the best choice. We expect that fairly extensive studies can be undertaken on other cryptic species of *B. tabaci* through the use of heterologous sequences once the *B. tabaci* Q genome sequence is available. The funding for this sequencing project is primarily supported by a Major International (Regional) Joint Research Project from the Chinese National Science Foundation in 2014 (Award Number: 31420103919). This

UK
UNIVERSITY OF KENTUCKY

College of Agriculture
Department of Entomology
Office of the State Entomologist
S-225 Agriculture Science Center-N
Lexington, KY 40546-0091

sequencing effort, led by PIs Youjun Zhang (China) and Xuguo Zhou (US) and facilitated by BGI, uncovers molecular signatures of adaptation over evolutionary time which may contribute to the highly invasive and adaptive nature of this global pest.

We believe that the manuscript is well organized and will be of interest to the broad readership of *GigaScience*. As a community paper, this manuscript not only will benefit researchers within the International Whitefly Genome Consortium, but also researchers interested in biological invasions and insect-plant interactions. Thank you in advance for your time and effort in evaluating this work; we look forward to hearing from you.

With respect,

X. G. Zhou

Xuguo "Joe" Zhou
Associate Professor, Ph.D.
Insect Integrative Genomics
Department of Entomology
University of Kentucky
E-Mail: xuguozhou@uky.edu
Phone: 859-257-3125
Fax: 859-323-1120
