# Supplementary material for: Genome sequencing of the sweetpotato whitefly Bemisia tabaci MED/Q
Source: Gigascience. 2017 Mar 15;6(5):1–7. doi: 10.1093/gigascience/gix018 (PMC5467035; doi:10.1093/gigascience/gix018)
Supplement: GIGA-D-16-00061_Revision_1.pdf [file gix018_GIGA-D-16-00061_Revision_1.pdf]

mainbody
Click here to download Manuscript GigaScience_main text_research article_12-6-16_V3_JZ.docx

For consideration:

Giga Science

The invasive Q-type *Bemisia tabaci* genome: a tale of gene loss and gene gain

Authors and affiliations

Wen Xie[1]¶, Chunhai Chen[2]¶, Zezhong Yang[1]¶, Litao Guo[1]¶, Xin Yang[1]¶, Dan Wang[2], Ming Chen[2], Jinqun Huang[2], Yanan Wen[1], Yang Zeng[1], Yating Liu[1], Jixing Xia[1], Lixia Tian[1], Hongying Cui[1], Qingjun Wu[1], Shaoli Wang[1], Baoyun Xu[1], Xianchun Li[4], Xinqiu Tan[5], Murad Ghanim[6], Huipeng Pan[3], Shunxiang Ren[7], Baoli Qiu[7], Dong Chu[8], Helene Delatte[9], M. N. Maruthi[10], Feng Ge[11], Xueping Zhou[12], Xiaowei Wang[13], Fanghao Wan[12], Yuzhou Du[14], Chen Luo[15], Fengming Yan[16], Evan L. Preisser[17], Xiaoguo Jiao[18], Brad S. Coates[19], Jinyang Zhao[2], Qiang Gao[2], Jinquan Xia[2], Ye Yin[2]*, Yong Liu[5]*, Judith K. Brown[4]*, Xuguo "Joe" Zhou[3]*, Youjun Zhang[1]*

1 Institute of Vegetables and Flowers, Chinese Academy of Agricultural Science, Beijing 100081, China, **2** BGI-Shenzhen, Shenzhen 518083, China, **3** Department of Entomology, S-225 Agricultural Science Center North, University of Kentucky, Lexington, KY 40546-0091, USA, **4** School of Plant Sciences, University of Arizona, Tucson, AZ 85721, USA, **5** Institute of Plant Protection, Hunan Academy of Agricultural Sciences, Changsha 410125, China, **6** Department of Entomology, Volcani Center, Bet Dagan 5025001, Israel, **7** Key Lab of Bio-pesticide Creation and Application, South China Agricultural University, Guangzhou 510642, China, **8** College of Agronomy and Plant Protection, Qingdao Agricultural University, Qingdao 266109, China, **9** Cirad, UMR PVBMT, Saint-Pierre, La Re´union, France, **10** Natural Resources Institute, University of Greenwich, Chatham Maritime, Kent ME4 4TB, UK, **11** Institute of Zoology, Chinese Academy of Sciences, Beijing 100101, China, **12** Institute of Plant Protection, Chinese Academy of Agricultural Sciences, Beijing 100193,

China, **13** Ministry of Agriculture Key Laboratory of Agricultural Entomology, Institute of Insect Sciences, Zhejiang University, Hangzhou 310058, China, **14** School of Horticulture and Plant Protection and Institute of Applied Entomology, Yangzhou University, Yangzhou 225009, China, **15** Institute of Plant and Environment Protection, Beijing Academy of Agriculture and Forestry Sciences, Beijing 100089, China, **16** Collaborative Innovation Center of Henan Grain Crops, College of Plant Protection, Henan Agricultural University, Zhengzhou 450002, China, **17** Department of Biological Sciences, University of Rhode Island, Kingston, Rhode Island 02881, USA, **18** College of Life Sciences, Hubei University, Wuhan 430062, China. **19** United States Department of Agriculture, Agricultural Research Service, Corn Insects & Crop Genetics Research Unit, Ames, IA 50011, USA.

¶These authors contributed equally to the work.

*To whom correspondence should be addressed. Email: Youjun Zhang (zhangyoujun@caas.cn), Ye Yin (yinye@genomics.cn), Xuguo "Joe" Zhou (xuguozhou@uky.edu), Judith K. Brown (JBrown@ag.arizona.edu), Yong Liu (haoasliu@163.com).

Abstract

Background: The whitefly *Bemisia tabaci* is a highly destructive agricultural and ornamental crop pest. It damages host plants through both phloem feeding and vectoring plant pathogens. Introductions of *B. tabaci* are difficult to quarantine and eradicate because of its high reproductive rates, broad host plant range, and insecticide resistance.

Results: A 658 Mb draft genome for *B. tabaci* MED/Q was assembled and annotated with 20,786 protein-coding genes. The cytochrome P450 monooxygenases and several other metabolic pathways contained an expanded number of gene family members. The amino acid biosynthesis pathways are partitioned among the host and endosymbiont genomes in a manner distinct from other hemipteran genomes. Evidence of horizontal gene transfer to the host genome may explain their obligate relationship. Putative loss-of-function of the immune deficiency signaling pathway due to the gene loss is a shared ancestral trait among hemipteran insects.

Conclusions: The expansion of P450 gene family members may contribute to the insecticide tolerance of MED/Q and facilitate its invasion in intensively-managed crop systems. This whitefly genome provides the foundation for research into the evolution of endosymbiotic relationships, as well as the mechanism(s) providing a competitive advantage to invasive species; both factors likely contribute to the worldwide success of *B. tabaci*.

Keywords

Bemisia tabaci, Detoxification enzymes, Genome, Gene gain and loss, Invasive species, Symbiosis

Background

As a globally invasive species, the phloem-feeding whitefly *Bemisia tabaci* (Genn.; hereafter '*Bemisia*') has been found on all continents except Antarctica [1,2]. Taxonomically, *B. tabaci* is considered a species complex that contains several morphologically-indistinguishable but genetically-distinct 'cryptic species' [2-7]. The members of this complex predominantly feed on herbaceous eudicot plant species [8]; they have been reported on over 900 species, including a large number of food, fiber, and/or ornamental crops (Global Invasive Species Database). While they can inflict significant feeding-related damage, their larger impact on plant health occurs via their role as a vector for over 111 plant viruses [9,10].

Accidental introductions of *Bemisia* into previously whitefly-free regions has harmed agricultural productivity and led to its classification as one of the World's Worst Invasive Species (Global Invasive Species Database: http://www.issg.org/database/welcome/). The *Bemisia* Middle East-Asia Minor 1 (MEAM1, or 'B') cryptic species is highly invasive and has emerged as a major pest in the United States, Caribbean Basin, Latin America, Middle East [1], and East Asia [11]. Similarly, the invasive *Bemisia* Mediterranean (MED, or 'Q') cryptic species has been introduced into several geographic locations and become established throughout China [12,13]. Although both MEAM1/B and MED/Q are successful invasives, they prefer different host species, vary in their response to virally-infected plants [13,14], and differ in their ability to vector *tomato yellow curl leaf virus* ('*TYLCV*') [12,15] and other plant viruses. The success of MEAM1/B as an invasive may be related to its high ratio of diploid female to haploid male progeny, a reproductive advantage which allows it to outnumber and competitively displace native *Bemisia* cryptic species [16]. In contrast, the MED/Q cryptic species is resistant to several classes of chemical insecticides, providing it a substantial advantage in agricultural and other managed landscapes [12]. Because of their invasive nature and impact on agricultural production, the MEAM1/B and MED/Q cryptic species have been extensively studied and proposed as model species for exploring range expansion through adaptations to invaded habitats. Despite substantial research, however, the genetic or genomic basis for the adaptive plasticity and selective advantages of both *Bemisia* cryptic species remain obscure.

Bemisia and other phloem-feeding hemipterans rely upon obligate bacterial endosymbionts to provide the essential amino acids and vitamins lacking in plant sap. While all *Bemisia* cryptic species rely on the primary endosymbiont *Candidatus (Ca.) Portiera aleyrodidarum* (*Portiera*) [17], some also harbor one or more facultative bacterial endosymbionts whose role in host survival is unknown [18,19]. The *Bemisia*-bacteria symbiosis has evolved a cross-linked set of systems for supplying enzymatic components to key metabolic pathways. The adaptive changes in host immune pathways and pathogen detection, and the mechanisms by which endosymbionts evade these defenses, remain poorly understood. Moreover, variation in endosymbiont communities within *Bemisia* is associated with distinct host haplotypes, and increasing evidence suggests that these endosymbiont communities may influence the competency of different *Bemisia* to vector *TYLCV* [20].

We report the first draft genome sequence for *Bemisia* MED/Q. We focus on pathways for pathogen recognition and several metabolic pathways that contain a large and highly diverse gene members to shed light on the polyphagous nature of MED/Q, its resistance to insecticide, and the evolutionary underpinnings of its symbiosis. The MED/Q genome provides a resource for future investigations that address climatic and host plant adaptations, invasive-invasive and native-exotic interactions, insecticide resistance, vector competence, and its relationships with bacterial endosymbionts.

Data Description

For details on assemblies, annotations and other analyses see 'methods' section. This whole genome shotgun project has been deposited at DDBJ/EMBL/GenBank under the accession LIED00000000. The version described in this paper is version LIED01000000. The final, assembly *Portiera* (PRJNA299729/SAMN04214819/LNJY00000000) and *Hamiltonella* (PRJNA299727/SAMN04214805/LNJW00000000) genome of MED/Q, respectively, are accessible at NCBI.

Analyses

Genome sequencing and assembly

Results of mtCOI gene PCR-RFLP assays [21], and direct DNA sequencing followed by phylogenetic evaluation against reference sequences [22] both confirmed that the *Bemisia* in the MED/Q colony belonged to the Q1 haplotype group, or western Mediterranean region clade (data not shown). Using genomic DNA from the MED/ colony, a total of 20 whole genome sequence (WGS) shotgun sequencing libraries were generated (18 pooled male and female PE and MP libraries, and two haploid-male derived WGA PE libraries), from which sequences were generated on an Illumina Hiseq2500 platform. Library sequencing produced a total of 428.2 Gb or an approximate 594.7-fold genome coverage assuming a 0.72 Gbp genome size (based on 17-mer analysis). For the 10 short-insert PE libraries, there were a total of 229.4 gigabases (Gb) (100 bp or 150 bp read length, approximately 318.6-fold genome coverage). Sequencing the eight large-insert (>1 kb) MP libraries produced 80.3 Gb of reads (49 bp read length, 111.5-fold coverage) for use in scaffold construction (S1 Table). The two male WGA libraries produced a total of 118.5 gigabases (Gb) of data (S1 Table) or approximately 164.6-fold genome coverage Sequencing of 13 BAC pools generated 362.6 Gbp of raw data (288.4 Gbp processed data; results not shown). The subsequent assembly of this sequence data using our pipeline (S1 Fig) generated a 658.0 Mbp draft genome assembly for MED/Q, with a scaffold N50 of 437 kb (Table 1). The assembled 658 Mb MED/Q draft genome size is consistent with recent flow cytometry estimates [23]. The mean read depth across 10 kb windows indicated that all genome regions were highly represented within the read data, with < 1.5% having a depth of < 10X (remaining data not shown).

Annotation of repetitive elements

Homology-based annotation of MED/Q repetitive elements was queried against Repbase v.20.05 [24] with RepeatMasker [25]. We found a total of 299.0 Mbp repetitive DNA, or 45.4% of the MED/Q genome size. This was about 10% higher than the repeat contents of *Acyrthosiphon pisum* and *Rhodnius prolixus*, but less than that of *Nilaparvata lugens* (48.6%). As in *N. lugens*, transposon elements (TEs) in MED/Q were primarily responsible for the repeat content difference (48.6% and 45.4%, respectively). This suggests that long terminal repeat (LTR)-like retroelements (18.5%) are more abundant and contain more nucleotides than all other TE classes. This proliferation of LTR retrotransposons has only been found in one other Hemipteran genome, that of *N. lugens* (12.29%). The MED/Q genome also contains the high proportion of the DNA-transposon TEs (12.92%) found in other fully-described Hemipteran genomes. As with both *N. lugens* (0.5%) and *R. prolixus* (0.01%), the MED/Q genome also appears devoid of short interspersed nuclear elements (SINEs; 0.96%). These other Hemipteran genomes also contain a small amount of long interspersed nuclear elements (LINEs; *A. pisum*: 2.6%; MED/Q: 3.18%; *R. prolixus*: 3.2%),

but *N. lugens* (12.84%). This suggests that MED/Q-specific TEs, especially the LTRs, have evolved relatively recently and contribute to the large number of gene sets (S2 Table).

Gene coverage and annotation of coding regions

Preliminary evaluation of transcribed regions within the draft MED/Q genome assembly coverage found that ~95.2% of *B. tabaci* ESTs > 200 bp were present, with 90,652 ESTs showing ≥ 90% length coverage on one scaffold (S15 Table). This alignment encompassed 92.9% of nucleotides within the EST dataset. Analogously, 229 (96%) of the 248 sequences in the CEGMA gene set were present in the MED/Q genome assembly, fewer than the 245 and 236 obtained from *A. pisum* and *R. prolixus* genomes, respectively (remaining data not shown). The final GLEAN gene models predicted a reference gene set of 20,786 protein-coding genes, a consensus result derived from *de novo*, orthology, and evidence (RNA-seq)-based prediction methods (S3 Table) and integrated into GLEAN gene models (S4 Table). Among the GLEAN gene models, 16,622 (79.97%) received functional gene annotations using the various databases queried in our analysis pipeline (S5 Table). 57% of the un-annotated GLEAN gene models were supported by either RNA-seq or EST sequence data (remaining data not shown).

Prediction of gene orthology and gene family expansion

Phylogenetic analysis based on orthologs across 14 arthropod taxa (S6 Table) suggested that MED/Q is clustered into a hemipteran clade containing *A. pisum*, and is a sister lineage to a clade containing both *R. prolixus* and *N. lugens* (Fig 1A). The range of species-specific genes within the four hemipteran genomes ranged from 38-60%, with higher values for the three phloem-feeding specialists. This led us to investigate interspecific changes in the number and diversity of gene family members (orthologs and paralogs) within this group of Hemiptera (Fig 1C; Fig S2). We found that 1,978 gene models unique to MED/Q were associated with putative gene family expansions, with the largest expansions occurring in gene families linked to transmembrane transport and oxidative-reduction processes (S7 Table). The number of MED/Q gene family members among the UDP glycosyltransferases (UGTs; n = 63), carboxyl/choline esterases (COE; n = 51), and ATP-binding cassettes transporters (ABC; n = 59) was not significantly different from those found in other phloem- or blood-feeding arthropods. In contrast, the cytochrome monooxygenase P450 detoxification gene family was significantly expanded (Fig 2A). The 56 expansions in the MED/Q P450s gene family are the most seen in any Hemipteran genome, and second only to *T. castaneum* (n = 68) among the 14 fully-sequenced arthropod genomes (S8 Table). These expansions have produced 153 predicted MED/Q P450 genes, with the most expansion occurring in the CYP3 and CYP4 clades; this is the greatest number found in any arthropod genome (Fig 2B).

We also found 3,474 genes belonging to groups that show putative reductions in the number of gene family members; these were mainly annotated as being involved in RNA-dependent DNA replication and DNA integration (e.g., difference in transposon component of the genomes), and cell surface receptor signaling (e.g., immune response; S9 Table). Further inspection of gene annotation information showed that genes in the immune deficiency (IMD) pathway were absent from the MED/Q genome (Fig 3; S10 Table).

Metagenomics and analysis of MED/Q endosymbiosis

The bacterial metagenome of insects has been shown to contribute to the overall fitness and viability of insects. Many hemipterans have evolved bacteriocytes, the specialized structures

that house endosymbiotic bacteria. Although genomes are available for both the primary MED/Q endosymbiont, *Ca. Portiera* aleyrodidarum (CP003867, CP003835 and CP007563) and secondary endosymbiont, *Hamiltonella* (AJLH00000000, AJLH02000000) [17,19], the lack of a corresponding whitefly genome sequence has precluded investigations into this interaction with respect to metabolic and gene pathway partitioning. We used a metagenomics approach to re-assemble the complete genomes of *Portiera* (0.35 Mb) and *Hamiltonella* (1.8 Mb) from filtered Illumina reads generated from shotgun sequencing libraries.

We used the MED/Q and endosymbiont genomes to compare gene models whose functional annotations suggest their involvement in amino acid biosynthesis and to reconstruct the corresponding enzymatic pathways. This revealed a tight relationship between MED/Q and *Portiera*. There were 47 MED/Q and 45 *Portiera* enzymes involved in amino acid biosynthesis encoded in the two genomes; the intact pathway requires enzymatic components encoded by both genomes. Pathway analysis showed that both genomes are needed to produce ten essential amino acids; Arg, His, Ile, Leu, Lys, Met, Phe, Try Thr, and Val (Fig 4). While MED/Q encodes enzymes that contribute precursor substrates for Trp, Phe and Thr synthesis, *Portiera* encodes enzymes required to complete the synthesis of these amino acids. Conversely, *Portiera* provides intermediates required by MED/Q to synthesize Arg, His, Ile, Leu, Lys, Met, Tyr, and Val (Fig 4A). Our analysis of analogous amino acid biosynthesis pathways found that MED/Q and its *Hamiltonella* endosymbiont are both capable of synthesizing Cys, Lys, Pro, and Thr (Fig 5). Comparative genomic pathway analysis also showed that MED/Q lacks the capacity to synthesize five B-class vitamins; biotin, folate, NAD, riboflavin, and vitamin B6. We predict that the *Hamiltonella* genome encodes enzymes necessary for the biosynthesis of these B vitamins (S11 Table; S12 Table).

We used a phylogenetic approach to test the hypothesis that horizontal gene transfer (HGT) was involved in the gain of certain amino acid biosynthetic pathways. Our results suggest that the MED/Q genome contains *Portiera*-derived endosymbiotic bacterial genes. The phylogenetic pipeline we used for HGT detection identified 11 putative events based on phylogenetic clustering of MED/Q genes with bacterial counterparts (Fig S3). In ten of these 11 predicted HGT events the clusters of MED/Q encoded genes were most closely related to their bacterial orthologs. In the other instance, the MED/Q gene for argininosuccinate synthase was at the base of a bacterial-origin clade and adjacent to a second insect-derived clade (Fig S3A). Functional annotations suggest that six HTG events (those involving argH, 2 dapF paralogs, lysA, dapB, and E3.1.3.15B) are likely involved in the complementation of the Arg, His, and Lys biosynthetic pathways (Fig 4A). Six MED/Q genes involved in these 11 putative HTG events contained introns and four had a 5'-untranslated region (UTR), in spite of the fact that their closest evolutionary relationships were to prokaryotic orthologs (S13 Table). Comparisons between amino-acid synthesis pathways in the MED/Q-*Portiera*, *A. pisum*-*Buchnera* and *N. lugens*-yeast-like host-endosymbiont relationships suggest that MED/Q endosymbionts play an essential role in the production of seven essential amino acids (Fig 4B). By comparison, the genome sequences of the MED/Q counterpart aphid- and planthopper-endosymbiont study systems primarily encode transaminases (S14 Table), and provide either substrates or intermediates involved in the regulation of amino acid synthesis. While aphid and planthopper endosymbionts have a role in glycolysis and the pentose phosphate pathway, *Portiera* lacks a detectable role in either pathway (Fig 4A,C).

Functional validation of genes encoding detoxification enzymes

The numbers of exons in the CYP4 subfamily (CYP304G2, CYP402C9, CYP4R2 and CYP4G69) range from six to ten, but all of the CYP6 subfamily (CYP6CX4, CYP6DB3, CYP6DV6, CYP6DW2 and CYP6EM1) contain six exons (Fig 6A, C). The involvement of

genes encoding 9 CYP450s and 3 GSTs in imidacloprid resistance was validated using *in vivo* dietary RNA interference (RNAi). The efficiency of dietary RNAi was shown in Figure S5. The susceptibility of *B. tabaci* controls and knockouts to 0.2mM imidacloprid was documented in Figure 6. In CYP4 subfamily, the median survival rate of dsEGFP controls was 63%. In contrast, the median survival rate of CYP4 subfamily knockouts, including CYP304G2, CYP402C9, CYP4CR2, and CYP4G69, was 63, 59, 63, and 47%, respectively (Fig 6B). A log-rank test analysis showed that while the survival of CYP304G2 and CYP4CR2 knockouts did not differ from the controls, survival of CY402C9 and CYP4G69 knockouts was significantly lower. In CYP6 subfamily, the median survival rate of CYP6CX4, CYP6DB3, CYP6DV6, CYP6DW2, and CYP6EM1 knockouts was 59, 47, 59, 63, and 41%, respectively (Fig 6D). Log-rank analysis revealed that the silencing of CYP6CX4, CYP6DB3, and CYP6DV6 significantly decreased survivorship, while silencing of CYP6DW2 did not affect survival rate. For GSTs, the median survival rate of GSTM1, GSTD9, and GSTD6 knockouts was 59, 59, and 72% (Fig 6F); suggesting that the silencing of GSTM1 and GSTD9 significantly decreased the survival of imidacloprid-exposed whiteflies.

Discussion

Species in the *Bemisia* complex have a high reproductive rate, broad plant host range that includes many eudicot plant families, and the ability to adapt to a wide range of local environmental conditions. These traits likely contribute to the ability of *Bemisia* to rapidly develop insecticide resistance and become invasive in novel habitats [1]. The evolutionary history of *Bemisia* has altered its genome via adaptations that can influence the breadth of functions carried out by certain gene families [26]. For instance, cytochrome P450 monooxygenase genes encode enzymes involved in a variety of biosynthetic and metabolic pathways, including xenobiotic detoxification [27,28]. These form a repertoire of defensive pathways employed by herbivorous insects that feed on chemically-defended plants [29]. Increases in the number of gene family members involved in metabolic detoxification is hypothesized to decrease the impact of environmental toxins via an increase in metabolic rates when the duplicates are co-regulated [30]. Evolutionary processes can also diversify duplicated gene families such that temporal and spatial variation occurs with respect to gene expression patterns or its members derive innovated functions [31]. In addition, some expanded gene families in B-type *B. tabaci* genome like cathepsins and phosphatidylethanolamine-binding proteins, were found to be associated with virus acquisition and transmission and/or combined insecticide resistance likely contributing to the global invasiveness and efficient virus vectoring capacity of *B. tabaci* [32].

The analysis of multiple arthropod genomes has identified lineage-specific expansions in the P450 gene family, such as the expansion of the CYP3 and CYP4 clades in the scorpion *Mesobuthus martensii* [33]. An increasing number of P450s in the CYP3 and CYP4 clades also occurs in the genome of *T. castaneum* [34], and here in the MED/Q genome (Fig 2). This may reflect the fact that both CYP3 and CYP4 are involved in detoxification and the evolution of insecticide resistance in arthropods [35-39]. The large number of these detoxification genes in MED/Q likely contributes to its high degree of insecticide resistance, but further comparative works is necessary to rigorously assess correlations between specific gene family member expansions and species-level biological/ecological characteristics. Understanding the genetic and genomic mechanisms that underlay the ability of arthropods to rapidly involve insecticide resistance may help devise practices to circumvent this cycle of recurrent adaptation.

Another fascinating topic for future research involves the degree to which the ability of invasive *Bemisia* to establish and exclude native *Bemisia* has a genomic basis. In China, for instance, the introduction of MED/Q led to the extirpation of the formerly-dominant invasive MEAM1/B from many locales [12,40]. P450 genes in *Bemisia* can be induced under various conditions, including host plant switches, and are upregulated in insecticide-resistant strains [41,29]. The fact that these genes are also constitutively expressed under normal conditions [42], however, suggests they are either primed for rapid response to stress or involved in cellular homeostasis. Although neonicotinoid insecticides are effective against phloem-sucking insects, including whiteflies, aphids, and thrips, high levels of resistance, however, have been recently reported for *B. tabaci* in the field worldwide [43-45]. Previous studies showed that some Cytochrome P450s and GSTs are overexpressed in neonicotinoid-resistant *Bemisia* [41, 46-51]. In this study, nine CYP450s and three GSTs were subjected to dietary RNAi-mediated functional analyses. The silencing of a CYP6 gene, CYP6EM1, significantly increased the susceptibility of *B. tabaci* to insecticide treatments, indicating a potential role in imidacloprid resistance. In other insects, UGT are primarily sugar donors that are closely related to various plant allelochemical- and odorant-degrading enzymes [52-54]. Research has found that UGT in *Bemisia* plays a role in allowing whiteflies to feed on nicotine-defended plants [55], suggesting that UGT-linked detoxification of plant defenses may facilitate polyphagy in *Bemisia*. Conducting genome-level comparisons of detoxification-

related gene families allows the testing of hypotheses regarding the capacity of MED/Q to resist insecticides, feed on multiple host plants, and adapt to the novel stressors present in new environments.

Endosymbiosis between host eukaryotic cells and intracellular microorganisms involves a series of adaptive changes. The most ancient of these is likely the acquisition of environmental bacteria and subsequent HGT that led to the current mitochondrion and the nuclear-encoded mitochondrial components of the ATP biosynthesis pathway. Insects contain an array of intracellular bacteria and fungi, a proportion of which have formed mutualistic relationships with their hosts; such partnership appear particularly common in the order Hemiptera [56,57]. The impact of these symbiotic relationships on the metabolic capacity of host genomes are just starting to be revealed through whole-genome analyses [58]. All *Bemisia* species host the primary endosymbiont *Portiera aleyrodidarum* [59] and a variety of species-specific secondary endosymbionts; these include *Arsenophonus*, *Wolbachia*, *Hamiltonella*, *Candidatus*, *Cardinium*, *Fritschea* and *Rickettsia* [60-63]. Previous studies have suggested the potential for metabolic complementarity between *Bemisia* and *Portiera* and between *Bemisia* and *Hamiltonella* [18,19]. Transcriptome-based analysis of MEAM1/B that compared both bacteriocytes and whole-body samples reported that the host genome may contribute enzymes that complement or duplicate *Portiera*–encoded pathways, and that *Hamiltonella* might contribute multiple cofactors and the essential amino acid Lys [18]. Analysis of the *Portiera* genome found that it lacked genes essential for the biosynthesis of certain amino acids, a result supported by the findings of transcriptome-based research [17,64]. Our work provides the first assessment of gene reduction and metabolic pathway complementarity in the MED/Q-symbiont relationship. We found that *Portiera* allows MED/Q to survive on phloem sap by facilitating the synthesis of 10 essential amino acids (Fig 4), and that *Hamiltonella* contributes B-vitamins that its host cannot produce (Fig 5B).

The presence of numerous metabolic pathways that require both the host and its endosymbionts suggests that theirs is an obligate symbiosis. While such complementarity could evolve through gene loss in either the host or endosymbiont via relaxation of selective constraints when multiple copies of a given gene are present, it can also occur through HGT. For instance, enzymes involved in carotenoid biosynthesis were derived from fungal genes integrated into the *A. pisum* genome [65]. Horizontally-transferred genes in the citrus mealybug *Planococcus citri* also contribute to functions absent in its endosymbionts, which evolved a different system of amino acid synthesis [66]. The fact that 11 enzymes require the biosynthetic pathways of both MED/Q and *Portiera* suggests that inter-genome pathway complementarity may be an initial step in such obligatory endosymbiotic relationships.

The insect immune system modifications necessary to facilitate the residency of previously extracellular bacteria that otherwise might be seen as pathogenic agents are also of interest. Acceptance of a foreign bacteria by the host, or evasion of host defenses by the bacteria, is essential for any intracellular symbiosis. In *D. melanogaster*, for instance, infection by Gram-negative bacteria activates the IMD pathway [67]. Since the obligate endosymbionts of both *Bemisia* and *A. pisum* are Gram-negative bacteria [67,68], the loss of IMB pathway components in both species may be more than a coincidence: loss of IMB function may have facilitated the acquisition of endosymbiotic bacteria [69]. We estimate the divergence of *Bemisia*, *A. pisum* and *N. lugens* occurred 286 million years ago (MYA) (Fig 1; Fig S4), an estimate consistent with other research dating the divergence of the Auchenorrhyncha (containing *N. lugens*) and Sternorrhyncha (containing *A. pisum* and *Bemisia*) to 290 MYA [70]. These evolutionary events and recent studies suggest that the ancestors of *Bemisia* and *A. pisum* acquired their obligate endosymbionts after separating from the group containing *N. lugens*, a split that may have involved a loss-of-function event in the IMD system of the former group. The absence of a functioning IMD pathway in

MED/Q may also have contributed to subsequent acquisition of *Hamiltonella* and other facultative endosymbionts that have been detected in members of the *Bemisia* complex.

Conclusions

Our report of the *Bemisia* MED/Q genome is a first for this economically-important invasive species. The insight it provides into metabolic gene expansion, nutrient/nutritional partitioning, and immune system reduction shed new light on the genetic and molecular basis of adaptations likely critical to the success invasion of *Bemisia*. Twelve detoxification genes (e.g., CYP6EM1) derived from MED/Q genome were investigated by both conventional PCR analysis and RNAi-mediated functional validation to assess their potential involvement in imidacloprid resistance. The correlation between the expanded cytochrome P450 gene family, a group well-known for their role in detoxification, and the high degree of insecticide resistance in MED/Q is unlikely to be coincidental. More importantly, this MED/Q genomic resource provides a foundation for future 'pan-genomic' comparisons of invasive vs. non-invasive, invasive vs invasive, and native vs. exotic *Bemisia* that opens new avenues of investigation into whitefly biology, evolution, and management.

Potential implications

Several members of the *Bemisia tabaci* complex are invasive species that cause considerable economic damage to agricultural and horticultural crops via their feeding and their role as plant pathogen vectors. Sequencing the genome of the Mediterranean Q *Bemisia* revealed molecular signatures of adaptation that may contribute to the highly invasive nature of this pest. An array of numerical and compositional changes in multiple gene families provides a foundation for future hypothesis testing that will further our understanding of adaptation, viral transmission, symbiosis, and plant-insect-pathogen tritrophic interactions.

Methods

Illumina library construction

The Q-type *Bemisia tabaci* was first reported in China when it was identified on *B. tabaci*-infested poinsettia plants imported into the Yunnan Province during 2003 [71]. Within only several years, the Q-type (MED) *B. tabaci* had invaded most of the southeastern and northeastern provinces in China, where it rapidly displaced the exotic B-type (MEAM1), previously established there [40]. Adult whitefly females (2n) and males (1n) were initially collected from infested field-grown cucumber plants in Beijing, China during 2011, and used to establish a laboratory colony (MED/Q) at the Institute of Vegetable and Flowers, Chinese Academy of Agriculture Science by transferring adult males and females to caged pepper plants (10-12 leaf stage).

The MED/Q whitefly colony was used as the source initial short shotgun Illumina sequencing. Adult whiteflies fed using Parafilm ®membrane sachets containing a 25% sucrose solution for 48 hrs prior to collection of ~5,000 male and female adults (~ 50:50). Samples were immediately frozen in liquid nitrogen for three hours prior to transfer to a -80°C freezer. This genomic DNA was used to construct Illumina TruSeq paired end (PE) sequencing libraries (170, 250, 300, 500 and 800 bp insert sizes) and mate pair (MP) libraries (2, 5, 10, 20 and 40 KB in size) according to manufacturer instructions.

Additionally, two Illumina PE sequencing libraries (~500bp and 800bp inserts) were constructed from whole genome amplification (WGA) reactions carried out on genomic DNA isolated from two adult male whiteflies. First, single males were dissected (body fluid and interior unclassified tissues by removing the outer cuticula) into 1x PBS buffer, and frozen for storage and transport. The PBS buffer containing the cells and tissues was thawed, briefly flicked to mix, and pipetted into PCR tubes in equal amounts. After blending and dissolving adequately, WGA of the DNA from a single male was performed according to the manufacturer's instructions using the REPLI-g Midi Kit (QIAGEN, Inc.), with a "no whitefly tissue" cell reaction as a negative control. Each step during the experiment was performed on a "clean bench" to avoid contamination. To assess the effect of the amplification, the DNA concentration of the WGA products was measured in a Qubit® dsDNA assay (Invitrogen Life Science, Inc.) according to the manufacturer's instructions, and the primary DNA band-size distribution was validated by agarose gel electrophoresis.

BAC library construction, pooling of clones, and Illumina library construction

High molecular weight (HMW) MED/Q total genomic DNA was isolated as previously described [72], then partially digested with *Hind*III , and size selected from pulsed-field gel electrophoresis (1% agarose gel in 0.5 X TBE gels run on BioRad CHEF-DRIII system) run for 16 hours at 14°C with initial and final time switches of 5 sec and 15 sec, respectively, in a voltage gradient of 6 V/cm. Sizes were compared to the Lambda Ladder PFG Marker (New England Biolabs), and ~100 kb genomic fragments exized. Isolated DNA was ligated into the pCC1BAC vector (Epicentre), transformed into EPI300 *E. coli* cells (Epicentre) by electroporation, and selected on LB agar with chloramphenicol, X-gal and Isopropyl-beta-D-thiogalactopyranoside (IPTG) as described by the manufacturer. A Genetix QPIX was used to pick and array clones into 192 384-well plates containing LB freezing media, and then frozen at -80°C. To BAC vector estimate insert sizes, 10 µl aliquots of BAC miniprep DNA were digested with 5 U of *Not*I enzyme for 2 hours at 37°C, and then separated by pulsed-field gel electrophoresis as described above. All clones from 384-well plates were pooled into 13 libraries (8 or 9 384-well plates per pool), and BAC vector DNA was isolated from LB liquid

cultures using Qiagen Large Construct Kits. Purified BAC DNA was used to construct indexed Illumina TruSeq PE libraries with ~500 bp insert size according to manufacturer instructions.

Genome sequencing and assembly

All libraries were sequenced on an Illumina Hiseq 2000 using 100 bp reads from both fragment ends, and raw data processed and assembled as shown (S1 Table; S1 Fig). Briefly, a series of filtering steps were performed on the raw reads to filter out the following: (1) reads with >10% Ns, more than 40% low-quality bases, more than 10 bp overlapping with adapter sequences, allowing no more than 3bp mismatches; (2) paired-end reads that overlapped more than 10 bp between two ends, with insert size larger than 200 bp libraries; and (3) duplicated reads generated by PCR amplification during the construction of the large-insert library. Filtered reads were used for K-mer determination within subsequent assembly steps. The frequency of each K-mer was calculated from the genome-sequence reads. K-mer frequencies along the sequence depth gradient follow a Poisson distribution in a given data set except for a high proportion at low frequency due to sequencing errors, as K-mers that contain such sequencing errors may be orphans among all splitting K-mers. The genome size, *G*, was estimated as *G* = *K*_num/*K*_depth, where *K*_num is the total number of K-mers, and *K*_depth is the maximal frequency. Initial contigs were assembled from filtered 500 and 800 bp insert-size WGA PE libraries using SOAPdenovo. The sequencing reads obtained for 2k-40kb MP libraries were used to connect the contigs and to generate the scaffolds as described by Li et al. (2010) [73] with a K-mer size of 65.

Individual BAC pools were assembled independently using SOAPdenovo and the whole genome shotgun reads from PE and MP libraries were used to fill gaps in the BAC scaffolds. After sequencing, the raw reads were filtered as described above. In addition, reads representing contamination by *E. coli* or the plasmid vector were filtered. The pooled reads were separated according to the BAC-reads index, and each BAC was assembled using a combination of "hierarchical assembly" and "*de Bruijn* graph assembly". First, the reads linked to each BAC were assembled using SOAPdenovo [73], with various combinations of parameters with a K-mer range from 27 to 63 and a step size of 6. The assembly with the longest scaffold N50 was defined as the "best" for each BAC. The resulting BACs were mapped with the large shotgun MP read data to optimize the assembly for each BAC.

The final draft assembly was produced by integrating sequences that overlapped among the scaffolds independently assembled from genome shotgun and BAC reads, and in doing so eliminated the redundant scaffolds using the following steps. In order to integrate the two assemblies, the software *Rabbit* [74] was applied to identify any relationship between scaffolds, to connect the overlapping regions that shared at least 90% similarity, and to remove redundancy based on a 17-mer frequency. Finally, *SSPACE* [75] was used to construct super-scaffolds containing 800 bp-40 kb WGS reads, and the 170-800 bp genome shotgun read data were used to fill the gaps using *GapCloser* [73].

Post-assembly processing included removal of contaminating bacterial and viral DNA sequences, by aligning all assembled sequences to the genome sequences of viruses and bacteria, obtained from previous local BLASTn alignments and by NCBI upload filter. Aligned sequences that shared >90% identity and were >200 bp in size were filtered from the final assembly. The assembled sequences that were covered by at least one EST sequence were retained. Process read data was mapped the the draft MED/Q genome using *SOAPaligner* software and read counts were made from .bam files and the average depth was computed from all bases in the window. The relation graph of base pair percentages, and each given sequencing depth along the genome, was obtained.

Annotation of repetitive elements

Repetitive elements were searched for and identified using *Repbase* [24] implemented in *TRF* software [76], and a *de novo* approach implemented in *Piler* [77]. For the *Repbase*-based method, two software programs named *RepeatMasker* [25] and *RepeatProteinMask* were used to identify repetitive sequences. In the *de novo* approach, *Piler-DF-1.0* [77], *RepeatScout-1.0.5* [78], and *LTR-FINDER-1.0.5* [79] were used to build *de novo* repeat libraries from the genome sequences. Finally, the repeated sequences were searched for and classified using the *RepeatMasker* software.

Gene coverage and annotation of coding regions

Initial evaluation of gene coverage rate in the draft MED/Q1-China genome assembly was assessed by comparing against 248 core eukaryotic genes were obtained using *CEGMA 2.4* [80]. Additionally, 105,067 *B. tabaci* transcript sequences, expressed sequence tags (ESTs), of > 200 bpwere used as BLASTn queries against the assembled genome in order to estimate the representation (cutoff *E*-value ≥ 10^{-40}). Protein-coding gene *de novo* predictions using GENEWISE [81] and *ab initio* gene predictions using GENSCAN [82] and AUGUSTUS [83] were made in combination with 13.7 Gbp of transcriptome (RNA-Seq) data including published Q-type *B.tabaci* body, guts, and salivary glands [84-86] and additional unpublished females and males data (FTP: http://111.203.21.119/download/*.fastq), to obtain consensus gene sets using GLEAN [87].

For homolog-based prediction, protein sequences from nine species (*A. pisum*, *A. mellifera*, *D. melanogaster*, *R. prolixus*, *Z. nevadensis*, *A. gambiae*, *B. mori*, *P. humanus* and *T. castaneum*) were aligned with the MED/Q genome scaffolds using *TblastN* (E-value<1e-5). Target sequences were used to search for accurate gene structures implementing the *GeneWise* software [81]. For the RNA-Seq datasets, the transcriptome reads were first aligned against the genome using *TopHat* [87] to identify candidate exon regions. Then, the *Cufflinks* software [88] was used to assemble the aligned reads into transcripts, and the open reading frames (ORFs) were predicted to obtain reliable transcripts using a Hidden Markov Model (HMM)-based training parameter. Finally, *GLEAN* [87] was used to integrate the predicted genes with the *de novo*, homologous, and RNAseq data to produce the final gene set. The functional annotation of genes was performed using *BLASTP* alignment to KEGG [89], SwissProt and TrEMBL [90] databases. Motifs and domains were determined by *InterProScan* [91] and protein database searches against ProDom, PRINTS, Pfam, SMART, PANTHER and PROSITE.

Prediction of gene orthology and gene family expansions

Twelve insect species including *Bemisia tabaci* (Genn.) (Gennadius, 1889) (Hemiptera: Aleyrodidae), *Acyrthosiphon pisum* (Harris, 1776) (Hemiptera: Aphididae), *Rhodnius prolixus* (Stal, 1859) (Hemiptera: Triatominae), *Nilaparvata lugens* (Stål, 1854) (Hemiptera: Delphacidae), *Pediculus humanus* (Linnaeus, 1758) (Phthiraptera: Pediculidae), *Apis mellifera* (Linnaeus, 1758) (Hymenoptera, Apidae), *Nasonia vitripennis* (Ashmead, 1904) (Hymenoptera, Pteromalidae), *Tribolium castaneum* (Herbst, 1797) (Coleoptera, Tenebrionidae), *Anopheles gambiae* (Giles, 1902) (Diptera, Culicidae), *Drosophila melanogaster* (Meigen, 1830) (Diptera, Drosophilidae), *Bombyx mori* (Linnaeus, 1758) (Lepidoptera, Bombycidae) and *Danaus plexippus* (Kluk, 1802) (Lepidoptera, Nymphalidae) and two divergent arthropods, *Daphnia pulex* (Müller, 1785) (O. Cladocera, Daphniidae) and *Tetranychus urticae* (C. L. Koch, 1836) (O. Arachnida, Tetranychidae), were used to predict orthologs and to reconstruct the phylogenetic tree. Gene families were identified using *TreeFam* [92,93], and single-copy gene families were assembled to reconstruct phylogenetic

relationships. i) Coding sequences of each single-copy family were concatenated to form one super gene group for each species. ii) All of the nucleotides at codon position 2 of these concatenated genes were extracted to construct the phylogenetic tree by *PhyML* [94], with a gamma distribution across sites and an HKY85 substitution model. iii) The same set of sequences at codon position 2 was used to estimate divergence times among lineages. iv) The fossil calibrations were set with two previous node data [95,96]. v) The *PAML* mcmctree program (v.4.5) [97,98] was used to compute split times using the approximate likelihood calculation algorithm. The software *Tracer* (v.1.5.0) (http://beast.bio.ed.ac.uk/software/tracer/) was utilized to examine the extent of convergence for two independent runs.

Gene family expansion and contraction analysis were performed using the software *CAFE 2.1* (http://sites.bio.indiana.edu/Bhahnlab/Software.html). In CAFE [99], a random birth and death model was used to predict gene gain and loss among gene families across the species-specific phylogenetic tree. Fisher's exact test (Pr0.01) was used to test for over-represented functional categories among the expanded genes and "genomic background" genes.

To detect orthologs evolving under positive selection in *B. tabaci*, an optimized branch-site model [100] was used to search the 1,299 single-copy ortholog genes of the four available species of Hemiptera (*B. tabaci*, *A. pisum*, *N. lugens* and *R. prolixus*). Briefly, these ortholog genes were first aligned using the molecular evolution tool *PRANK* [101], and then ambiguously aligned blocks revealed by *PRANK* alignments and filtered by *Gblocks* [102].

Detoxification enzymes within the putatively expanded cytochrome P450 monooxygenase gene family were identified using a homology-based strategy that used reference genes in *D. melanogaster*, *A. pisum*, *A. gambiae*, and *A. mellifera* gene models downloaded from (NCBI, http://www.ncbi.nlm.nih.gov/). First, we identified the detoxifying enzyme genes in MED/Q gene models by querying our gene set and scaffolds data with orthologous sequencing using the BLASTx algorithm (*E*-value $\leq 10^{-5}$). The segments with hits were linked by the Solar software, and parsed using Genewise software for gene predictions to enable the identification of full-length sequences. The resultant sequences were filtered in searches against the non-redundant (nr) and Interpro databases. After filtering false-positive mating sequences, the genes were manually corrected using the MED/Q transcriptome (mainly to P450 and UGT manual annotation), and phylogenetic trees were constructed using MEGA6.0 [103]. A similar method was applied to identify homologous genes in other selected insects.

The immunity related genes in MED/Q were identified by combining the results from motif-based and homology-based strategies, as previously described [104]. This comparison was accomplished by downloading the query sequences available in ImmunoDB [105], and from the NCBI database for the six insects *D. melanogaster*, *A. gambiae*, *A. aegypt*, *A. mellifera*, *C. quinquefasciatus* and *A. pisum*. The motif-based search, MAFFT [106] was used to align multiple protein sequences, and the software HMMER 3.0 was used to build models against which MED/Q sequences were searched using tblastn with hits linked using the Solar software. Genewise software [81] was used to improve the gene predictions and to obtain full-length gene sequences. The resultant sequences were manually edited and merged into a combined dataset of immune-related genes. The immune-related genes of *A. pisum* and *N. lugens* were used for comparisons with whitefly genes obtained using a similar approach.

Metagenomics and analysis of MED/Q endosymbiosis

To improve the *B. tabaci* MED/Q-*associated Hamiltonella* draft genome (AJLH00000000), sequencing data from 16 Illumina read paired-end libraries, ranging from 170 bp to 40 kb (44 lanes), from the MED/Q genome-sequencing project were used. Four sequences were

selected as references to filter candidate reads using SOAPaligner (Version: 2.21). These sequences included a previous draft of the *Hamiltonella* genome (372 scaffolds; AJLH00000000), the pea aphid *Hamiltonella* genome (CP001277), the *Yersinia pestis* CO92 complete genome (AL590842), and the *Serratia plymuthica* AS9 genome (CP002773). The SOAPaligner parameters were "-v 5" for the short insert size library (<1 kb) data and "-v 3 -R" for the large insert size library (>1 kb) data. SOAPdenovo (version 2.04) was used for genome assembly, using the parameters "-u -d 1 -F -K 45" on the above 170 bp to 40 kilo bp data. Gap filling was performed after scaffold construction, and a super-scaffold was also then obtained using the paired-end reads on >500 bp scaffolds to reduce the scaffold number. Then, the Unique Genome Profile (UGP) pipeline was applied to link the scaffolds using BAC sequences from MED/Q. Briefly, 1) the flanking 20 KB sequences of each scaffold were removed, and then unique tags (31-mer) were constructed; 2) the BAC sequences (<150 kb) were BLAST against unique tags; and 3) BACs that had more than two hits were filtered, and then used to construct link relationships to connect larger scaffolds. In addition, the genome of the MED/Q-associated primary endosymbiont *Portiera* was filtered and assembled (as described above) together with four previously reported *Portiera* genome reference sequences obtained from B-type and Q-type *B.tabaci* (GenBank: CP003708, CP003868, CP003867 and CP003835).

Genes were predicted for the finished *Hamiltonella* and *Portiera* genomes using Glimmer v3.02 (protein-coding genes), tRNAscan-SE (tRNAs) and RNAmmer v1.2 (rRNA). The putative coding sequences were annotated using BlastP similarity searches that showed consensus to the NR database (20121005). The E-value cutoff of 1e-5 and a minimum match percentage of 40% were selected for the analysis. Protein domain searches were conducted using InterProScan v4.8, available at the Pfam database, and the resulting coding sequences were used to search the KEGG database (http://www.genome.jp/tools/kaas/).

The amino acid synthesis-related genes in the MED/Q genome were searched against the NR database, under the scenario that they were not of insect origin. The genes identified in this way were used to construct a phylogenetic tree. To confirm that the HTGs identified were not contaminants associated with rogue bacterial sequences in the libraries, satisfying at least one of the two following conditions was necessary: 1) the HTGs were located on scaffolds that included coding regions homologous to other insects; and 2) the HTGs' transcripts should be present in alignments to a current transcriptome database (after manual corrections) and also as corresponding genes encoded by the genome.

Quantitative real-time PCR

Total RNAs were extracted from 30-40 *B. tabaci* adults (mixed sexes, female: male=1: 1) per strain using a Trizol reagent (Tiangen Corp., Beijing, China) following the manufacturer’s protocol. The total RNA was resuspended in the nuclease-free water and quantified with a spectrophotometer (Thermo Scientific Nanodrop 2000). Subsequently, the first-strand cDNAs were synthesized using the PrimeScript® RT reagent Kit (Takara Biotech, Tokyo, Japan) with gDNA Eraser according to the manufacturer's protocol. Reverse transcription was performed on 1.0 µg of each RNA sample. Synthesis of P450 and GST genes dsRNA and application of RNAi to insect were carried out according to published protocols. In addition, dsRNAs were prepared using the T7 RiboMAX Express RNAi system and protocols (Promega).

A total 120 adults (three biological replicates, n=40) were subjected to qRT-PCR analysis. Nine cytochrome CYP450 and three GST genes sequences were obtained from this MED/Q genomic sequencing project (CYP450 mainly selected from MED/Q-specific expansions in Fig 2B; GSTs selected randomly from our GSTs annotation). Full length primers were designed to analysis the quantitative of genomic genes annotation, and qRT-

PCR primers were designed to amplify a 85- to 250-bp fragment at annealing temperature of 60 ^{o}C (S16 Table). The amplification efficiency of these q-PCR primers are 95-105%. The 20-µl reaction mixture consisted of 1 µl of 300ng cDNA (3 times diluted), 10 µl of the SYBR® Green Real-time PCR Master Mix with ROX (Tiangen Corp., Beijing, China), and 0.6 µl of each primer. qPCR was conducted with the ABI 7500 system by the following protocol: 15 min of activation at 95°C followed by 40 cycles of 10 s at 95°C, 30 s at 60°C, and 30s at 72°C. A 3-fold dilution series of the whitefly cDNA was constructed the relative standard curve to calculate the amplification efficiency of the 12 genes. EF-α of *B. tabaci* were used as reference gene [107]. The RNA interference efficiency in CYP450 and GST genes expression, normalized to reference gene, were calculated using the $2^{-\Delta\Delta Ct}$ method [108].

In vivo dietary RNA interference

Dietary RNAi was carried out in a feeding chamber consisting of a glass tube (20 mm in diameter × 50 mm long, open at both ends), which was covered at the top end by one layer of Parafilm membrane (Alcan Packaging, Chicago, IL, USA) [47]. A 0.2-mL of diet solution containing 5% yeast extract and 30% sucrose (wt/vol) was placed on the outer surface of the Parafilm and was covered with another layer to encase the solution between the Parafilms. Whiteflies were released into the other end of the tube. Then the tube was sealed with a black cotton plug and covered with a shade cloth. The end of the tube with a Parafilm membrane was facing a light source that was approximately 20 cm away. This dietary RNAi system was used to measure the impact of ingested dsRNAs on the susceptibility to imidacloprid insecticide in adult *B. tabaci*. Controls included no-insecticide (-, buffer), vehicle (+, buffer), and control gene (+, EGFP). Buffer was an aqueous solution of artificial diet containing 5% yeast extract and 30% sucrose (wt/vol). Treatments used the same artificial diet solution mixed with dsRNAs of P450s and GSTs (0.5 µg/µL) [47]. dsRNAs were synthesized in vitro using a T7 RiboMAX Express RNAi system following the manufacturer's protocol (Promega, P1700, USA). In the insecticide toxicity assay, *B. tabaci* adults were fed on both treatment and control diets containing 0.2 mM imidacloprid. Mortality was assessed after 2 hours of feeding. Newly emerged adults (24h within hatching, mix sexed) were introduced into the feeding chamber, and placed in an environmental chamber at 25°C, a photoperiod of L14: D10, and 80% RH. Survival data were analyzed with log-rank test using SPSS (SPSS for Windows, Rel. 17.0.0 2009. Chicago: SPSS Inc.).

Figures

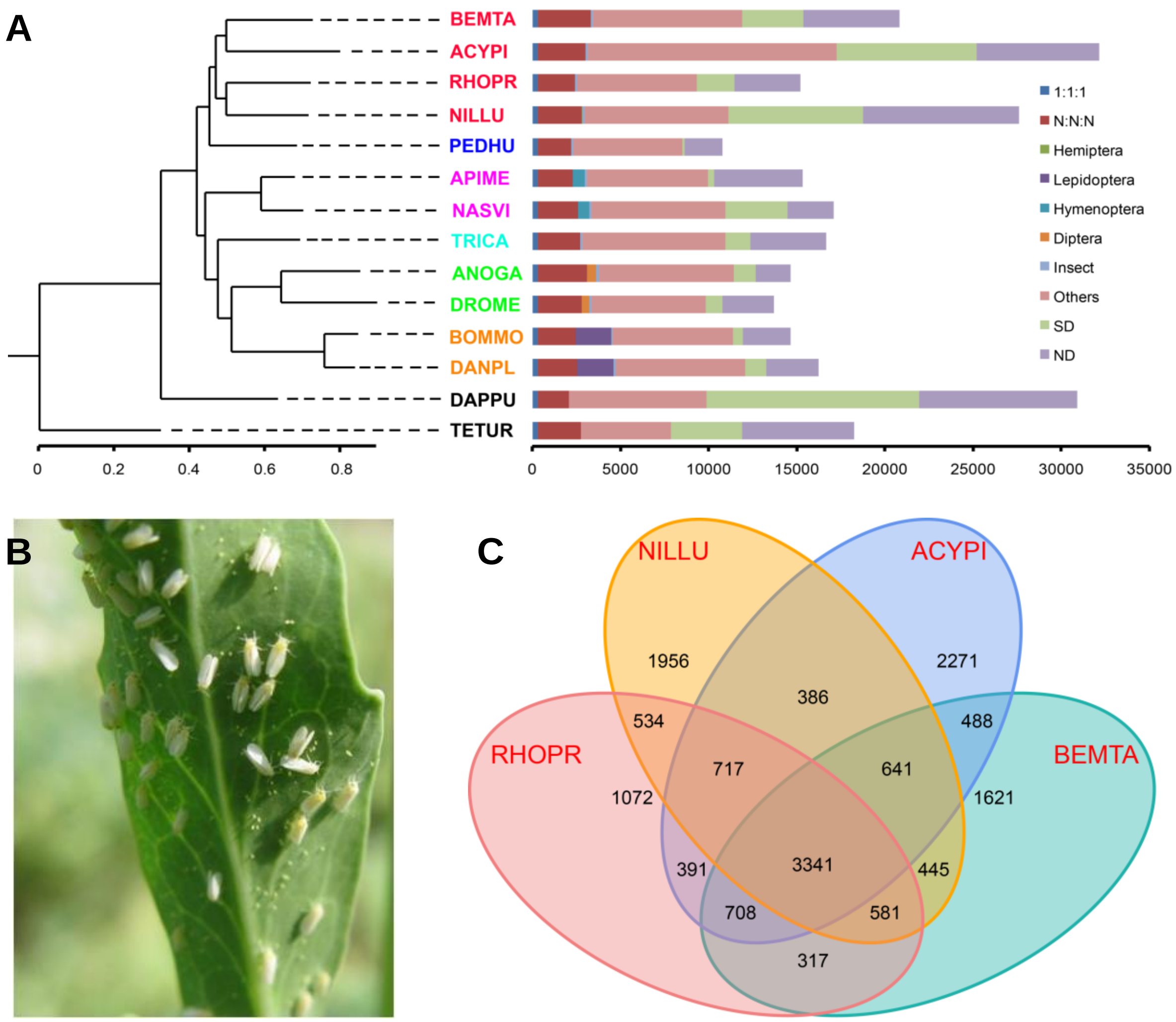

Figure 1

A

	P450	UGT	GST	ABC	COE	Total	
BEMTA*	153	63	21	59	51	347	Phloem feeding
ACYPI*	83	58	32	71	37	281	
NILLU*	65	23	9	57	56	210	
RHOPR	102	15	7	55	49	228	Blood feeding
PEDHU	39	4	12	38	20	113	
ANOGA	115	26	36	59	48	284	
DROME	85	34	32	53	35	239	
APIME	54	12	11	41	29	147	
NASVI	96	23	19	51	46	235	
TRICA*	126	28	30	13	51	248	
BOMMO*	72	33	27	55	89	276	

B

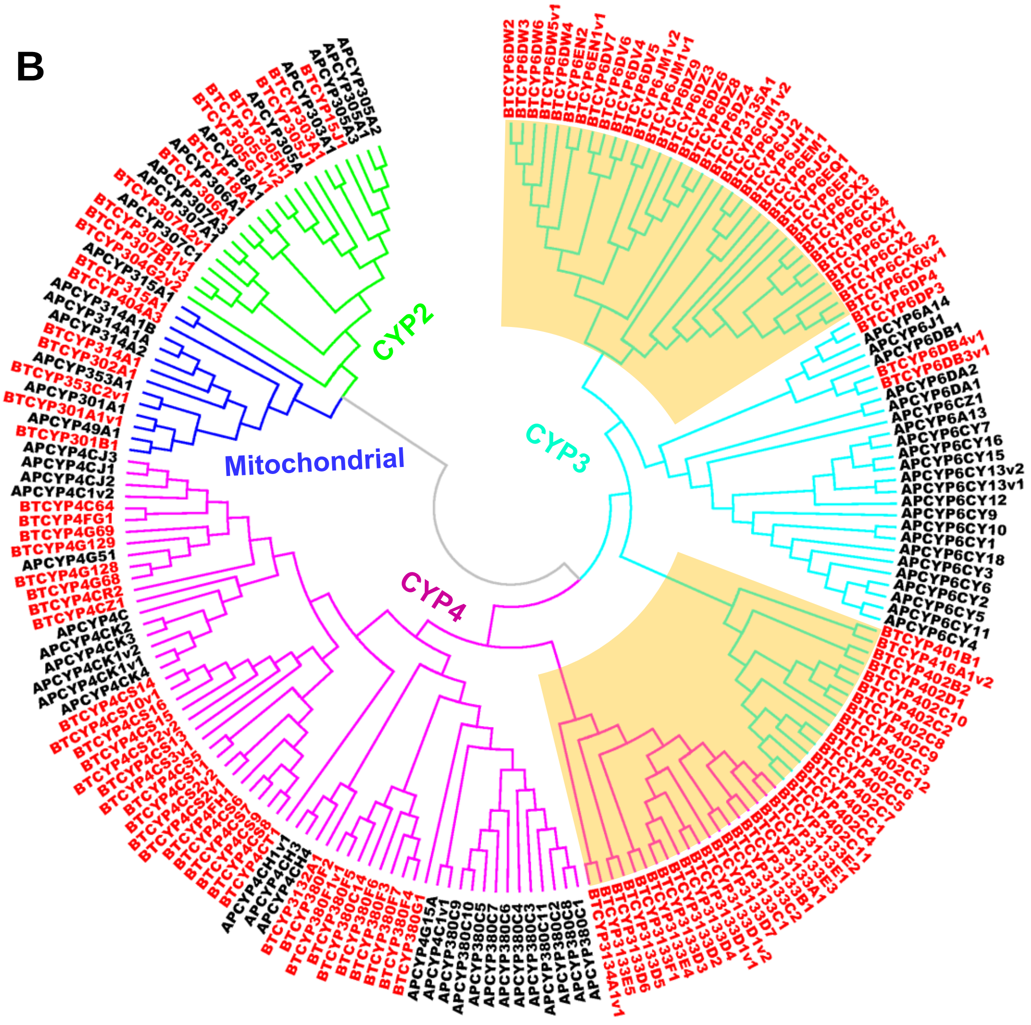

Figure 2

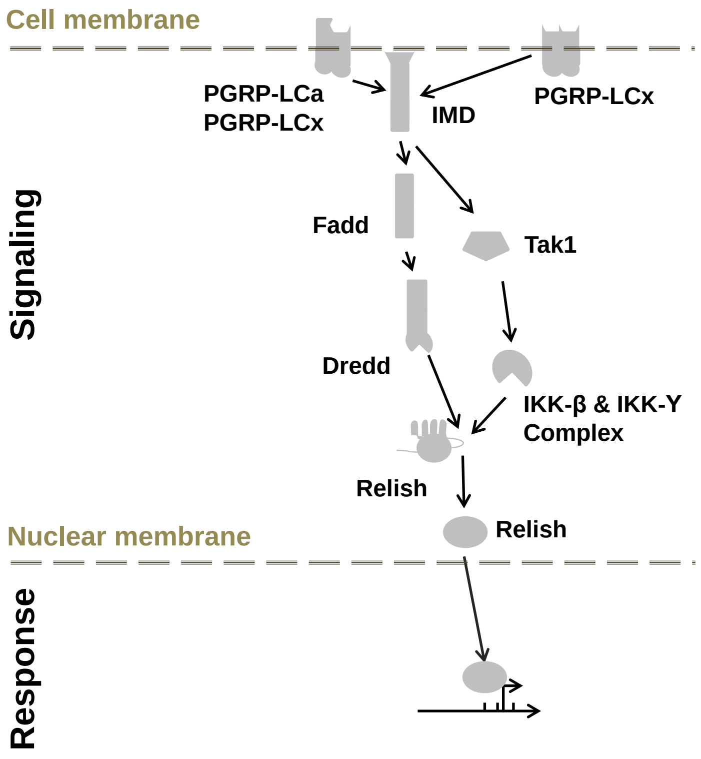

	Bt	Ap	Nl
PGRP-LCa	0	0	0
PGRP-LCx	0	0	1
IMD	0	0	1
Fadd	0	0	0
Dredd	0	0	1
Tak1	0	0	1
IKK-β	0	1	1
IKK-Y	0	0	1
Relish	0	0	1
Relish	0	0	1

Figure 3

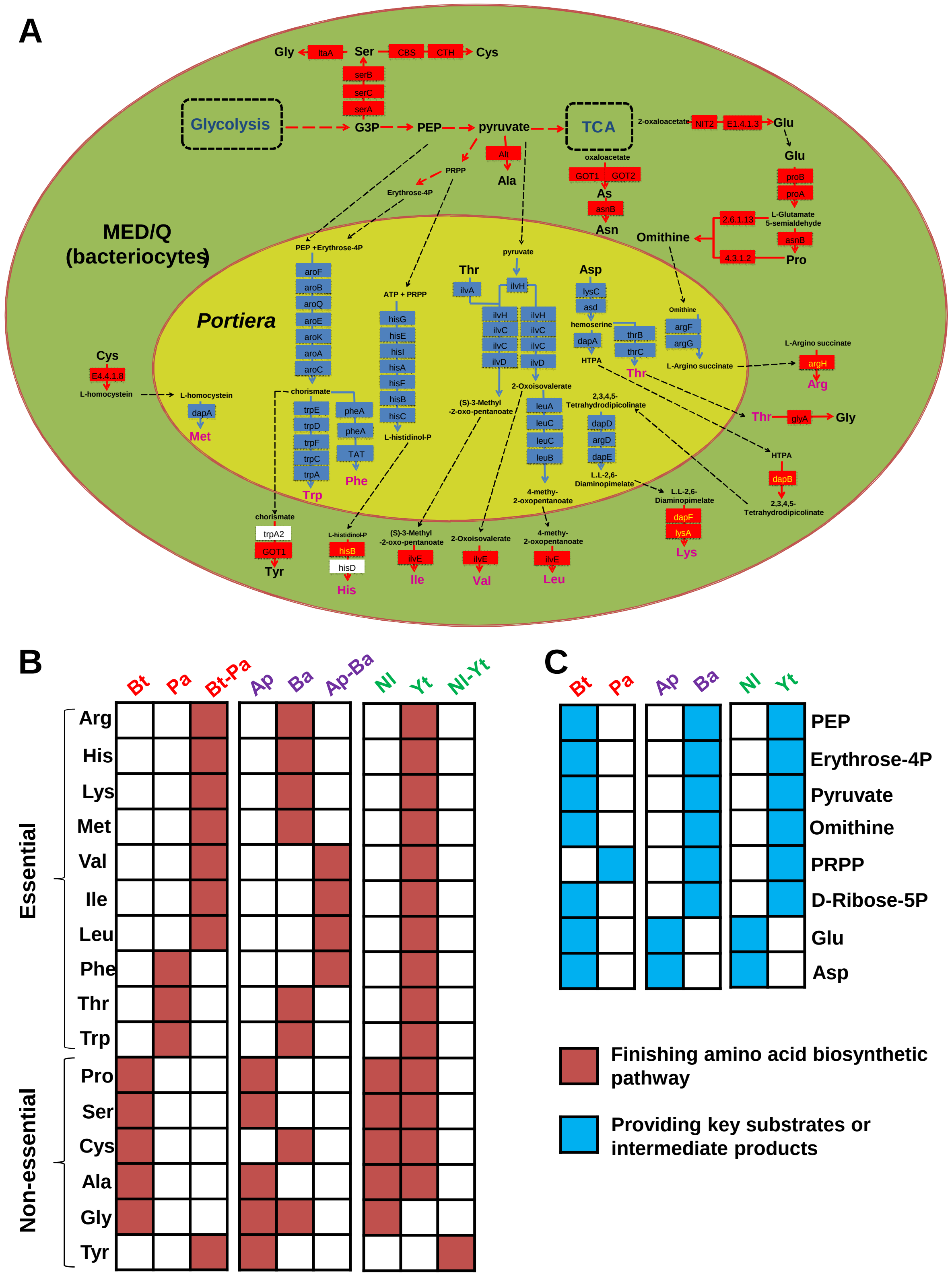

A
Glycolysis
TCA
MED/Q (bacteriocytes)
Portiera
Gly
Ser
Cys
G3P
PEP
pyruvate
Ala
Glu
Asn
Omithine
Pro
Arg
Thr
Lys
Tyr
His
Ile
Val
Leu
Met
Trp
Phe
B
Bt
Pa
Bt-Pa
Ap
Ba
Ap-Ba
Nl
Yt
Nl-Yt
Essential
Non-essential
Arg
His
Lys
Met
Val
Ile
Leu
Phe
Thr
Trp
Pro
Ser
Cys
Ala
Gly
Tyr
C
PEP
Erythrose-4P
Pyruvate
Omithine
PRPP
D-Ribose-5P
Glu
Asp
Finishing amino acid biosynthetic pathway
Providing key substrates or intermediate products

Figure 4

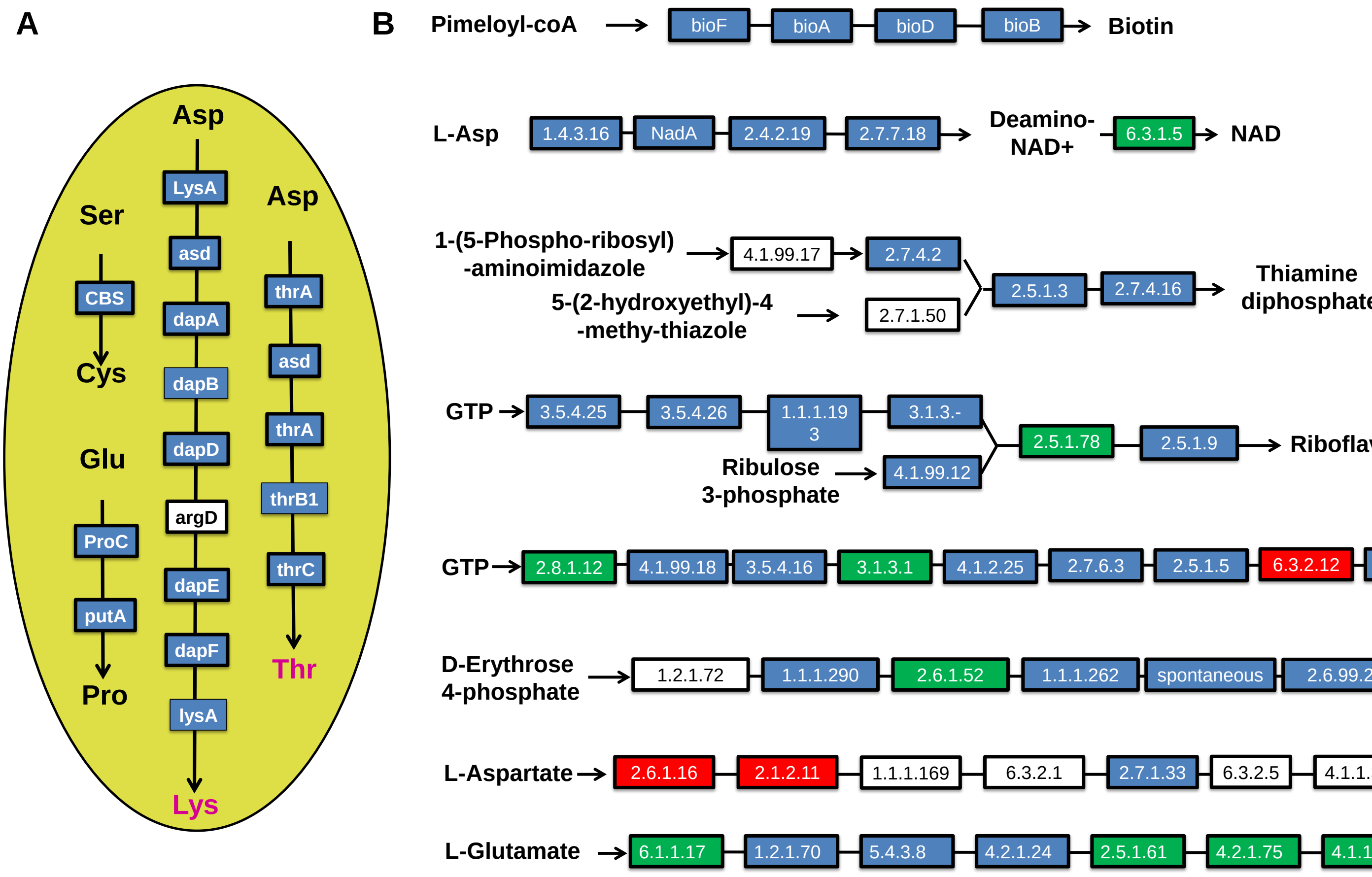
A
Asp
LysA
asd
dapA
dapB
dapD
argD
dapE
dapF
lysA
Lys
Ser
CBS
Cys
Glu
ProC
putA
Pro
Asp
thrA
asd
thrA
thrB1
thrC
Thr
B
Pimeloyl-coA
bioF
bioA
bioD
bioB
Biotin
L-Asp
1.4.3.16
NadA
2.4.2.19
2.7.7.18
Deamino-NAD+
6.3.1.5
NAD
1-(5-Phospho-ribosyl) -aminoimidazole
4.1.99.17
2.7.4.2
5-(2-hydroxyethyl)-4 -methy-thiazole
2.7.1.50
2.5.1.3
2.7.4.16
Thiamine
GTP
3.5.4.25
3.5.4.26
1.1.1.19 3
3.1.3.-
Ribulose 3-phosphate
4.1.99.12
2.5.1.78
2.5.1.9
GTP
2.8.1.12
4.1.99.18
3.5.4.16
3.1.3.1
4.1.2.25
2.7.6.3
2.5.1.5
6.3.2.12
D-Erythrose 4-phosphate
1.2.1.72
1.1.1.290
2.6.1.52
1.1.1.262
spontaneous
L-Aspartate
2.6.1.16
2.1.2.11
1.1.1.169
6.3.2.1
2.7.1.33
6.3.2.5
L-Glutamate
6.1.1.17
1.2.1.70
5.4.3.8
4.2.1.24
2.5.1.61
4.2.1.75

Figure 5

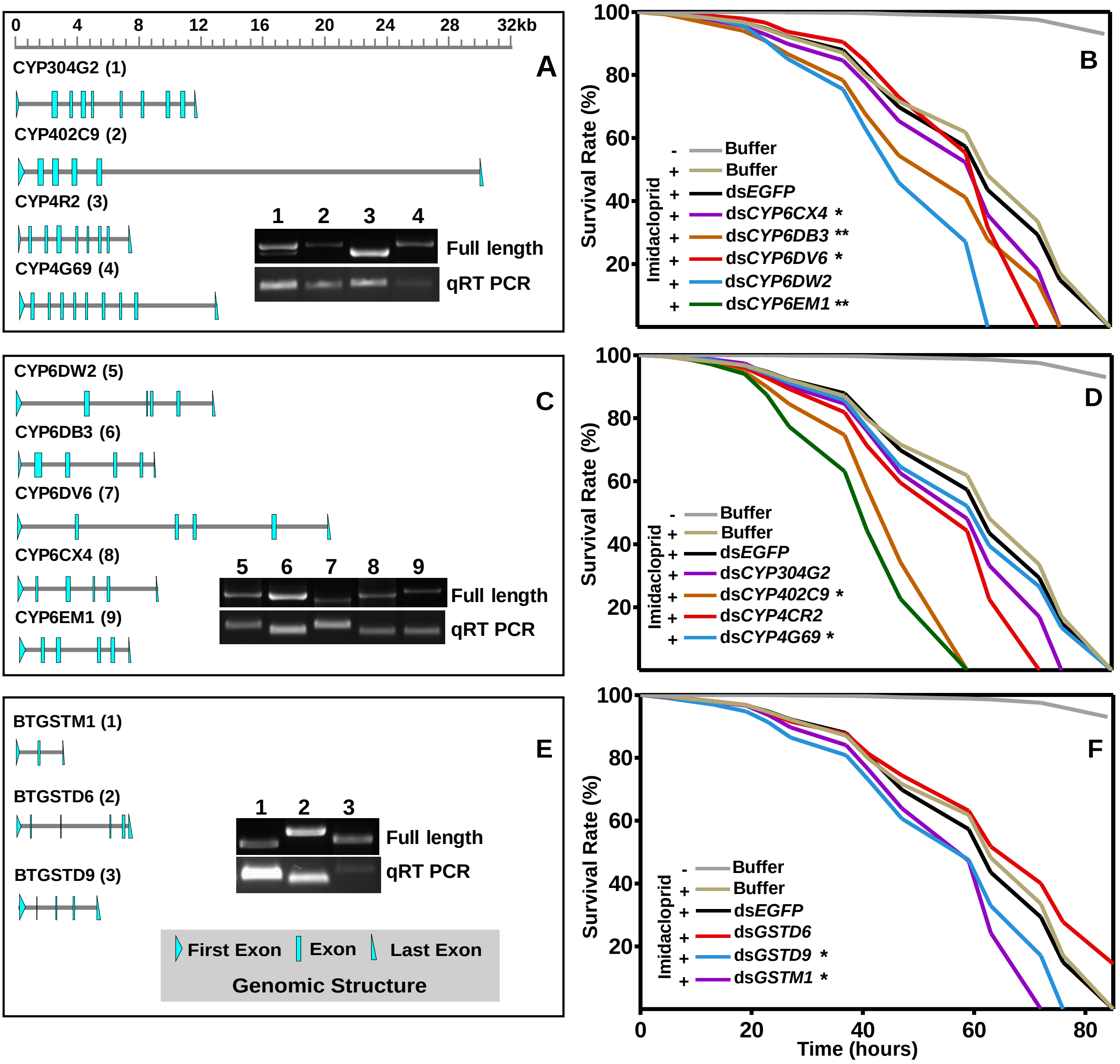

A
0 4 8 12 16 20 24 28 32kb
CYP304G2 (1)
CYP402C9 (2)
CYP4R2 (3)
CYP4G69 (4)
1 2 3 4
Full length
qRT PCR
B
Survival Rate (%)
Imidacloprid
- Buffer
+ Buffer
+ dsEGFP
+ dsCYP6CX4 *
+ dsCYP6DB3 **
+ dsCYP6DV6 *
+ dsCYP6DW2
+ dsCYP6EM1 **
C
CYP6DW2 (5)
CYP6DB3 (6)
CYP6DV6 (7)
CYP6CX4 (8)
CYP6EM1 (9)
5 6 7 8 9
Full length
qRT PCR
D
- Buffer
+ Buffer
+ dsEGFP
+ dsCYP304G2
+ dsCYP402C9 *
+ dsCYP4CR2
+ dsCYP4G69 *
E
BTGSTM1 (1)
BTGSTD6 (2)
BTGSTD9 (3)
1 2 3
Full length
qRT PCR
First Exon
Exon
Last Exon
Genomic Structure
F
- Buffer
+ Buffer
+ dsEGFP
+ dsGSTD6
+ dsGSTD9 *
+ dsGSTM1 *
100 80 60 40 20
0 20 40 60 80
Time (hours)

Figure 6

Tables

Table 1. Statistics of genome assembly and annotation for MED/Q

Assembled genome size (Mb)	658
Number of scaffolds (>100 bp)	5,003
Total size of assembled scaffolds	658,367,382 bp
N50 (scaffolds)	436,791 bp
Longest scaffold	2,857,362 bp
Number of contigs (>100 bp)	30,873
Total size of assembled contigs	638,748,832 bp
N50 (contigs)	44,388
Longest contig	362,835 bp
GC content	0.38
Number of GLEAN gene models	20,786
Mean transcript length	10,043 bp
Mean coding sequence length	1,504 bp
Mean number of exons per gene	5.12
Mean exon length	294 bp
Mean intron length	1,963 bp
Total size of TEs	265,205,801 bp
TEs proportion of the genome	0.4029

Figure Legend

Figure 1. Phylogenetic relationships and genomic comparisons between *Bemisia tabaci* and other insect species (**A**) Phylogenetic relationships of *B. tabaci* (BEMTA) to insects and other arthropods based on single-copy orthologous genes present in their complete genomes. The following twelve insect species were used for this analysis: *Acyrthosiphon pisum* (ACYPI), *Anopheles gambiae* (ANOGA), *Apis mellifera* (APIME), BEMTA, *Bombyx mori* (BOMMO), *Danaus plexippus* (DANPL), *Drosophila melanogaster* (DROME), *Nasonia vitripennis* (NASVI), *Nilaparvata lugens* (NILLU), *Pediculus humanus* (PEDHU), *Rhodnius prolixus* (RHOPR) and *Tribolium castaneum* (TRICA). The two arthropods *Daphnia pulex* (DAPPU) and *Tetranychus urticae* (TETUR) were used as outgroup taxa. Branch lengths represent divergence times estimated for the second codon position of 308 single-copy genes, using *PhyML* with a gamma distribution across sites and a HKY85 substitution model. The branch supports were inferred based on the approximate likelihood ratio test (aLRT). Gene orthology was determined by comparing the genomes of these 14 arthropod species. The use of 1:1:1 refers to single-copy gene orthologs found across all 14 lineages. The use of N:N:N refers to multi-copy gene paralogs found across the 14 lineages. Diptera, Hemiptera, Hymenoptera, Lepidoptera, and Insecta refer to taxon-specific genes present only in the particular lineage. SD indicates species-specific duplicated genes, and ND indicates species-specific unclustered genes. (**B**) Image of adult MED/Q. (**C**) A Venn diagram showing the orthologous groups shared among the hemipteran genomes of *A. pisum*, *B. tabaci*, *N. lugens* and *R. prolixus*. Our analysis found 3,341 gene families common to all four hemipteran genomes, and 2,921 common to the genomes of the six vascular (blood and phloem) feeders.

Figure 2. Expansion of gene families associated with metabolism and detoxification in

MED/Q genome. (**A**) Number of detoxification-related genes in the genomes of eleven selected insects (*denotes herbivorous insects) annotated as UDP glycosyltransferases (UGT), glutathione S-transferase (GST), ATP-binding cassette (ABC) transporter, and, carboxyl/choline esterases (COE). (**B**) Neighbor-joining phylogeny of the cytochrome P450 monooxygenase genes in annotated in the MED/Q genome assembly (red) and orthologs from *A. pisum* (black), with four insect CYP clades indicated as follows: CYP2 (green), CYP3 (blue green), CYP4 (pink), and the mitochondrial clade (blue). Predicted MED/Q-specific expansions within the CYP gene family are indicated (orange boxes).

Figure 3. Predicted orthologues associated with immune deficiency (IMD) within hemipterans. Schematic diagram illustrates the IMD signaling and the corresponding responses. The table shows the number of genes encoding each insect genome; this includes *B. tabaci* (Bt), *A. pisum* (Ap), and *N. lugens* (Nl).

Figure 4. Comparative analysis of amino-acid biosynthesis and provisioning mechanisms in *B. tabaci, A. pisum* and *N. lugens*. (**A**) Unique amino acid biosynthetic and supply mechanisms putatively related to the adaptation of MED/Q. Green and yellow areas denote bacteriocytes and endosymbiont cells (with respect to the filtered and annotated *Portiera* genome of MED/Q, PRJNA299729), respectively. Essential amino acids are represented in pink and non-essential amino acids in black; *Portiera* genes are in blue boxes. The Enzyme Commission numbers (EC) or enzyme names used correspond to those in the Kyoto Encyclopedia of Genes and Genomes (KEGG). MED/Q genes are indicated in red boxes. Black dotted lines represent transport processes between MED/Q and *Portiera*, and red dotted lines represent processes associated with MED/Q that occur within *Portiera* bacteriocytes. Candidate horizontally-transferred genes (HTGs) are highlighted in yellow

text; white boxes with black text represent unidentified genes. (**B**) Comparisons of amino acid biosynthesis in the host-symbiont bacterial systems of *B. tabaci-Portiera*, *A. pisum-Buchnera*, and *N. lugens*-yeast-like organism. Abbreviations: *Bt-Bemisia tabaci*, *Ap-Acyrthosiphon pisum*, *Nl-Nilaparvata lugens, Pa-Portiera, Ba-Buchnera, Yt-yeast-like*. The notation *Bt* (*Ap, Nl, Pa, Ba,* or *Yt*) means that MED/Q alone can complete the amino acid biosynthesis. The notation *Bt-Pa* (*Ap-Ba* or *Nl-Yt*) means that both MED/Q and at least two of its endosymbionts are required to complete the amino acid biosynthetic pathway. (**C**) Comparison of key substrates or intermediate products of the host-endosymbiont systems of *B. tabaci-Portiera, A. pisum-Buchnera* and *N. lugens*-yeast-like symbiont, illustrating that phosphoenolpyruvic acid (PEP), erythrose-4P, pyruvate, ornithine, and the precursor of histidine synthesis (PRPP) are important for amino acid synthesis. Pyruvate and PEP are produced by glycolysis and erythrose-4P by the pentose phosphate pathway. D-ribose-5P is the substrate for PRPP synthesis, and D-ribose-5P was converted based on D-glyceraldehyde 3-phosphate, also a product of glycolysis. Black arrows with dotted lines represent transport processes between MED/Q and *Portiera*.

Figure 5. Pathways encoded by *Candidatus hamiltonella* for amino acid and vitamin biosynthesis. (A) The major components of amino acid pathway encoded by *Hamiltonella*, a facultative *Bemisia* endosymbiont (essential amino acids in pink). *Hamiltonella* genes are highlighted in blue boxes, with names corresponding to its genome (PRJNA299727). (**B**) Independence and complementarity in the vitamin synthesis pathways of MED/Q (red box) and *Hamiltonella* (blue box). Green boxes denote candidate genes encoded by both MED/Q and *Hamiltonella*, while white boxes indicate genes that do not have a match in either genome.

Figure 6. **Effect of imidacloprid on MED/Q survival following dietary RNAi.** Genomic structures of CYP4 subfamily genes (*CYP304G2*, *CYP402C9*, *CYP4CR2* and *CYP4G69*), CYP6 subfamily genes (*CYP6CX4*, *CYP6DB3*, *CYP6DV6*, *CYP6DW2* and *CYP6EM1*), and GST genes (*GSTD6*, *GSTD9* and *GSTM1*) are shown in **A**, **C**, and **E**, respectively. Full length and qRT-PCR analyses are displayed in the inserted gel pictures. Survival rates of CYP4, CYP6, and GST knockouts when exposed to 0.2mM imidacloprid are documented in **B**, **D**, and **F**, respectively (log-rank test, ***** $p < 0.05$; ****** $p < 0.01$). Controls include no-insecticide (-, buffer), vehicle (+, buffer), and control gene (+, EGFP). Buffer refers to an aqueous artificial diet solution containing 5% yeast extract and 30% sucrose (wt/vol).

Abbreviations

AMP: anti-microbial peptide; ArK: adaptor protein; MED/Q1: Bemisia tabaci Q1; CEGMA: Core Eukaryotic Genes Mapping Approach; COE: carboxyl/choline esterases; cysLGIC: cys-loop ligand-gated ion channel; dsx: doublesex; EST: Express sequence tag; GABA: γ-aminobutyric acid; GluCls: glutamate-gated chloride channels; GST: glutathione S-transferases; HisCls: histamine-gated chloride channels; HMW: high molecular weight; HTGs: horizontally transferred genes; IMD: immune deficiency; IMP: insulin-like growth factor II mRNA-binding protein; IPTG: Isopropyl-beta-D-thiogalactopyranoside; MED: Mediterranean; mtCOI: mitochondria cytochrome oxidase I; MYA: million years ago; nAChRs: nicotinic acetylcholine receptors; NA-Med-ME: North Africa-Mediterranean-Middle East; OUTs: operational taxonomic units; P450: cytochrome P450 mono-oxygenases; PHCls: pH-sensitive chloride channel; PSI: P-element somatic inhibitor; Sxl: Sex-lethal; TEs: transposable elements; Tra: transformer; Tra2: transformer 2; TYLCV: tomato yellow leaf curl virus; UGTs: UDP glycosyltransferases; WGA: whole-genome amplified; WGS: whole genome shotgun

Acknowledgments and Funding

The authors would like to thank Dr. Paul De Barro for his comments on an earlier draft. This research was supported by the National Natural Science Foundation of China (31420103919 and 31672032), the Science and Technology Innovation Program of the Chinese Academy of Agricultural Sciences (CAAS-ASTIP-IVFCAAS) and the Beijing Key Laboratory for Pest Control and Sustainable Cultivation of Vegetables. The funders had no role in study design, data collection and analysis, decision to publish, or preparation of the manuscript.

Authors' contributions

YJZ is the leader of the project and the first corresponding author. WX, YJZ, XGZ, YY, JKB and YL were involved in the project design. XGZ, BYX, JYZ, QG, XCL, XQT, MG, HPP, SXR and BLQ coordinated the related research works of the MED/Q genome project. DW performed genome assembly. DW performed protein-coding gene annotation. MC and CHC performed gene orthology and phylogenomics. XY performed insecticide targets annotation. YTL performed putative sex determination genes annotation. WX performed putative phloem specialization genes identification. LTG, LXT, YNW, YZ, QJW, SLW and HYC performed metabolic detoxification systems annotation. ZZY performed immune signaling pathway components annotation. ZZY, JQX, and JQH performed nutrient partitioning between invasive MED/Q and its primary endosymbiont. LTG performed PCR validation. WX, XGZ, DC, JKB, HD, MNM, FG, XPZ, XWW, FHW, YZD, CL, FMY, ELP and XGJ were involved in writing and editing. All authors read and approved the final manuscript.

Competing interests

The authors declare no competing interests defined by *Giga Science*.

References

1. Brown JK, Frohlich DR, Rosell RC. The sweetpotato or silverleaf whiteflies: biotypes of *Bemisia tabaci* or a species complex? Ann Rev Entomol. 1995; 40:511-534. doi: 10.1146/annurev.en.40.010195.002455.
2. De Barro PJ, Liu SS, Boykin LM, Dinsdale AB. *Bemisia tabaci*: astatement of species status. Ann Rev Entomol. 2011; 56:1-19. doi: 10.1146/annurev-ento-112408-085504.
3. Liu SS, Colvin J, De Barro P. Species concepts as applied to the whitefly *Bemisia tabaci* systematics: how many species are there? J Inter Agric. 2012; 11:176-186. doi: 10.1016/S2095-3119(12)60002-1.
4. Wang HL, Yang J, Boykin LM, Zhao QY, Wang YJ, Liu SS, et al. Developing conversed microsatellite markers and their implications in evolutionaryanalysis of the *Bemisia tabaci* complex. Sci Rep. 2014; 4:6351. doi: 10.1038/srep06351.
5. Tay WT, Evans GA, Boykin LM, De Barro PJ. Will the real *Bemisia tabaci* please stand up? PLoS One. 2012; 7:e50550. doi: 10.1371/journal.pone.0050550.
6. Boykin LM, Armstrong KF, Kubatko L, De Barro P. Species delimitation and global biosecurity. Evol Bioinform Online. 2012; 8:1-37. doi: 10.4137/EBO.S8532.
7. Boykin LM. *Bemisia tabaci* nomenclature: lessons learned. Pest Manag Sci. 2014; 70:1454-1459. doi: 10.1002/ps.3709.
8. Mound LA, Halsey SH. Whitefly of the world. A systematic catalogue of the Aleyrodidae (Homoptera) with host plant and natural enemy data. Chichester: British Museum and John Wiley & Sons, 1978; 340pp John Wiley and Sons.
9. Brown JK, Zerbini FM, Navas-Castillo J, Moriones E, Ramos-Sobrinho R, Silva JC, et al. Revision of *Begomovirus* taxonomy based on pairwise sequence comparisons. Arch Virol. 2015; 160:1593-1619. doi: 10.1007/s00705-015-2398-y.
10. Jones DR. Plant viruses transmitted by whiteflies. Eur J Pl Pathol. 2003; 109:195-219.

doi: 10.1023/A:1022846630513.

11. Zhang LP, Zhang YJ, Zhang WJ, Wu QJ, Xu BY, Chu D. Analysis of genetic diversity among different geographical populations and determination of biotypes of *Bemisia tabaci* in China. J Appl Entomol. 2005; 129:121–128. doi: 10.1111/j.1439-0418.2005.00950.x.
12. Pan HP, Preisser EL, Chu D, Wang SL, Wu QJ, Carriere Y, et al. Insecticides promote viral outbreaks by altering herbivore competition. Ecol Appl. 2015; 25:1585-1595. PMID: 26552266.
13. Liu BM, Yan FM, Chu D, Pan HP, Jiao XG, Xie W, et al. Multiple forms of vector manipulation by a plant-infecting virus: *Bemisia tabaci* and tomato yellow leaf curl virus. J Virol. 2013; 87:4929-37. doi:10.1128/JVI.03571-12.
14. Iida H, Kitamura T, Honda K. Comparison of egg-hatching rate, survival rate and development time of the immature stage between B- and Q-biotypes of *Bemisia tabaci* (Gennadius) (Homoptera: Aleyrodidae) on various agricultural crops. Appl Entomol Zool. 2009; 44:267-273. doi: http://doi.org/10.1303/aez.2009.267.
15. Pan HP, Chu D, Yan WQ, Su Q, Liu BM, Wang SL, et al. Rapid spread of tomato yellow leaf curl virus in China is aided differentially by two invasive whiteflies. PLoS One. 2012; 7: e34817. doi:10.1371/journal.pone.0034817.
16. Liu SS, De Barro PJ, Xu J, Luan JB, Zang LS, Ruan YM, et al. Asymmetric mating interactions drive widespread invasion and displacement in a whitefly. Science. 2007; 318:1769-1772. doi: 10.1126/science.1149887.
17. Santos-Garcia D, Farnier PA, Beitia F, Zchori-Fein E, Vavre F, Mouton L, et al. Complete genome sequence of “*Candidatus* Portiera aleyrodidarum” BT-QVLC, an obligate symbiont that supplies amino acids and carotenoids to *Bemisia tabaci*. J Bacteriol. 2012; 194:6654-6655. doi: 10.1128/JB.01793-12

18. Luan JB, Chen W, Hasegawa DK, Simmons AM, Wintermantel WM, Ling KS, et al. Metabolic coevolution in the bacterial symbiosis of whiteflies and related plant sap-feeding insects. Genome Biol Evol. 2015; 7:2635-2647. doi: 10.1093/gbe/evv170.

19. Rao Q, Rollat-Farnier PA, Zhu DT, Santos-Garcia D, Silva FJ, Moya A, et al. Genome reduction and potential metabolic complementation of the dual endosymbionts in the whitefly *Bemisia tabaci*. BMC Genomics. 2015; 16:226. doi: 10.1186/s12864-015-1379-6.

20. Gottlieb Y, Zchori-Fein E, Mozes-Daube N, Kontsedalov S, Skaljac M, Brumin M, et al. The transmission efficiency of *tomato yellow leaf curl virus* by the whitefly *Bemisia tabaci* is correlated with the presence of a specific symbiotic bacterium species. J Virol. 2010; 84:9310-9317. doi: 10.1128/JVI.00423-10.

21. Chu D, Hu X, Gao C, Zhao H, Nichols RL, Li X. Use of mitochondrial cytochrome oxidase I polymerase chain reaction-restriction fragment length polymorphism for identifying subclades of *Bemisia tabaci* Mediterranean group. J Econ Entomol. 2012; 105:242-251. doi: http://dx.doi.org/10.1603/EC11039.

22. Frohlich DR, Torres-Jerez I I, Bedford ID, Markham PG, Brown JK. A phylogeographical analysis of the *Bemisia tabaci* species complex based on mitochondrial DNA markers. Mol Ecol. 1999; 8:1683-1691. doi: 10.1046/j.1365-294x.1999.00754.x.

23. Guo LT, Wang SL, Wu QJ, Zhou XG, Xie W, Zhang YJ. Flow cytometry and K-mer analysis estimates of the genome sizes of *Bemisia tabaci* B and Q (Hemiptera: Aleyrodidae). Front Physiol. 2015; 6:144. doi: 10.3389/fphys.2015.00144.

24. Jurka J, Kapitonov VV, Pavlicek A, Klonowski P, Kohany O, Walichiewicz J. Repbase Update, a database of eukaryotic repetitive elements. Cytogenet Genome Res. 2005; 110:462-467. doi:10.1159/000084979.

25. Smit AFA, Hubley R, Green P. RepeatMasker. 1999; http://www.repeatmasker.org.

26. Lespinet O, Wolf YI, Koonin EV, Aravind L. The role of lineage-specific gene family expansion in the evolution of eukaryotes. Genome Res. 2002; 12:1048-1059. doi:10.1101/gr.174302.

27. Feyereisen R. Evolution of insect P450. Biochem Soc Trans. 2006; 34:1252-1255. doi: 10.1042/BST0341252.

28. Feyereisen R. 8-Insect CYP genes and P450 enzymes. Insect Mol Biol Biochem. 2012; 236-316. doi: 10.1016/B978-0-12-384747-8.10008-X.

29. Xie W, Wang SL, Wu QJ, Feng YT, Pan HP, Jiao XG, et al. Induction effects of host plants on insecticide susceptibility and detoxification enzymes of *Bemisia tabaci* (Hemiptera: Aleyrodidae). Pest Manag Sci. 2011; 67:87-93. doi: 10.1002/ps.2037.

30. Ffrench-Constant, RH. The molecular genetics of insecticide resistance. Genetics. 2013; 194:807. doi: 10.1534/genetics.112.141895.

31. Force A, Lynch M, Pickett FB, Amores A, Yan YL, Postlethwait J. Preservation of duplicate genes by complementary, degenerative mutations. Genetics. 1999; 151:1531-1545. PMID: 10101175.

32. Chen W, Hasegawa DK, Kaur N, Kliot A, Pinheiro PV, Luan JB, et al. The draft genome of whitefly *Bemisia tabaci* MEAM1, a global crop pest, provides novel insights into virus transmission, host adaptation, and insecticide resistance. In Press. 2016. BMC Biology.

33. Cao Z, Yu Y, Wu Y, Hao P, Di Z, He Y, et al. The genome of *Mesobuthus martensii* reveals a unique adaptation model of arthropods. Nat Commun. 2013; 4: 2602. doi: 10.1038/ncomms3602.

34. Zhu F, Moural TW, Shah K, Palli SR. Integrated analysis of cytochrome P450 gene superfamily in the red flour beetle, *Tribolium castaneum*. BMC Genomics. 2013; 14:174. doi: 10.1186/1471-2164-14-174.

35. Yang Y, Chen S, Wu S, Yue L, Wu Y. Constitutive overexpression of multiple cytochrome P450 genes associated with pyrethroid resistance in *Helicoverpa armigera*. J Econ Entomol. 2006; 99:1784-1789. PMID: 17066813.

36. Yamamoto K, Ichinose H, Aso Y, Fujii H. Expression analysis of cytochrome P450s in the silkmoth, *Bombyx mori*. Pestic Biochem Phys. 2010; 97:1-6. doi:10.1016/j.pestbp.2009.11.006.

37. Yoon KS, Strycharz JP, Baek JH, Sun W, Kim JH, Kang JS, et al. Brief exposures of human body lice to sublethal amounts of ivermectin over-transcribes detoxification genes involved in tolerance. Insect Mol Biol. 2011; 20:687-699. doi: 10.1111/j.1365-2583.2011.01097.x.

38. Pridgeon JW, Zhang L, Liu N. Overexpression of CY4G19 associated with a pyrethroid-resistant strain of the German cockroach, *Blattella germanica* (L.). Gene. 2003; 314:157-163. PMID: 14527728.

39. Scharf ME, Parimi S, Meinke LJ, Chandler LD, Siegfried BD. Expression and induction of three family 4 cytochrome P450 (CYP4) genes identified from insecticide-resistant and susceptible western corn rootworms, *Diabrotica virgifera virgifera*. Insect Mol Biol. 2001; 10:139-146. PMID:11422509.

40. Pan H, Chu D, Ge D, Wang S, Wu Q, Xie W, et al. Further spread of and domination by *Bemisia tabaci* (Hemiptera: Aleyrodidae) biotype Q on field crops in China. J Econ Entomol. 2011; 104:978-985. doi: http://dx.doi.org/10.1603/EC11009.

41. Karunker I, Benting J, Lueke B, Ponge T, Nauen R, Roditakis E, et al. Over-expression of cytochrome P450 CYP6CM1 is associated with high resistance to imidacloprid in the

B and Q biotypes of *Bemisia tabaci* (Hemiptera: Aleyrodidae). Insect Biochem Mol Biol. 2008; 38:634-644. doi:10.1016/j.ibmb.2008.03.008.

42. Guo L, Xie W, Wang S, Wu Q, Li R, Yang N, et al. Detoxification enzymes of *Bemisia tabaci* B and Q: biochemical characteristics and gene expression profiles. Pest Manag Sci. 2014; 70:1588-1594. doi: 10.1002/ps.3751.
43. Nauen R, Denholm I. Resistance of insect pests to neonicotinoid insecticides: current status and future prospects. Arch Insect Biochem Physiol. 2005; 58: 200-215. doi: 10.1002/arch.20043.
44. Elbert A, Nauen R. Resistance of *Bemisia tabaci* (Homoptera: Aleyrodidae) to insecticides in southern Spain with special reference to neonicotinoids. Pest Manag Sci. 2000; 56: 60-64. doi: 10.1002/(SICI)15264998(200001)56:1<60::AID-PS88>3.0.CO;2.
45. Wang ZY, Yan HF, Yang YH, Wu YD. Biotype and insecticide resistance status of the whitefly *Bemisia tabaci* from China. Pest Manag Sci. 2010; 66: 1360-1366. doi: 10.1002/ps.2023.
46. Bass C, Denholm I, Williamson MS, Nauen R. The global status of insect resistance to neonicotinoid insecticides. Pestic Biochem Physiol. 2015; 121: 78-87. doi.org/10.1016/j.pestbp.2015.04.004.
47. Yang X, Xie W, Wang SL, Wu QJ, Pan HP, Liu BM, et al. Two cytochrome P450 genes are involved in imidacloprid resistance in field populations of the whitefly, *Bemisia tabaci*, in China. Pestic Biochem Physiol. 2013; 107: 343-350. doi.org/10.1016/j.pestbp.2013.10.002.
48. Yang NN, Xie W, Jones CM, Jiao XG, Yang X, Liu BM, et al. Transcriptome profiling of the whitefly *Bemisia tabaci* reveals stage-specific gene expression signatures for thiamethoxam resistance. Insect Mol Biol. 2013; 22: 485-496. doi:10.1111/imb.12038View/save citation.

49. Xie W, Yang X, Wang SL, Wu QJ, Yang NN, Li RM, et al. Gene expression profiling in the thiamethoxam resistant and susceptible B-biotype sweetpotato whitefly, *Bemisia tabaci*. J. Insect Sci. 2012; 12: 46-50. doi.org/10.1673/031.012.4601.

50. Yang X, He C, Xie W, Liu Y, Xia J, Yang, ZZ et al. Glutathione S-transferases are involved in thiamethoxam resistance in the field whitefly *Bemisia tabaci* Q (Hemiptera: Aleyrodidae). Pestic Biochem Phys. 2016; doi: 10.1016/j.pestbp.2016.04.003.

51. Ilias A, Lagnel J, Kapantaidaki DE, Roditakis E, Tsigenopoulos CS, Vontas, J, et al. Transcription analysis of neonicotinoid resistance in Mediterranean (MED) populations of *B. tabaci* reveal novel cytochrome P450s, but no nAChR mutations associated with the phenotype. BMC Genomics. 2015; 16: 939. doi: 10.1186/s12864-015-2161-5.

52. Bozzolan F, Siaussat D, Maria A, Durand N, Pottier MA, Chertemps T, et al. Antennal uridine diphosphate (UDP)-glycosyltransferases in a pest insect: diversity and putative function in odorant and xenobiotics clearance. Insect Mol Biol. 2014; 23:539-549. doi: 10.1111/imb.12100.

53. Luque T, O'Reilly DR. Functional and phylogenetic analyses of a putative *Drosophila melanogaster* UDP-glycosyltransferase gene. Insect Biochem Mol Biol. 2002; 32:1597-1604. doi:10.1016/S0965-1748(02)00080-2.

54. Sasai H, Ishida M, Murakami K, Tadokoro N, Ishihara A, Nishida R, et al. Species-specific glucosylation of DIMBOA in larvae of the rice armyworm. Biosci Biotech Bioch. 2009; 73:1333-1338. doi: 10.1271/bbb.80903.

55. Kliot A, Kontsedalov S, Ramsey JS, Jander G, Ghanim M. Adaptation to nicotine in the facultative tobacco - feeding hemipteran *Bemisia tabaci*. Pest Manag Sci. 2014; 70:1595-1603. doi: 10.1002/ps.3739.

56. Urban JM, Cryan JR. Two ancient bacterial endosymbionts have coevolved with the planthoppers (Insecta: Hemiptera: Fulgoroidea). BMC Evol Biol. 2012; 12:87. doi:

10.1186/1471-2148-12-87.

57. Kuechler SM, Gibbs G, Burckhardt D, Dettner K, Hartung V. Diversity of bacterial endosymbionts and bacteria–host co-evolution in Gondwanan relict moss bugs (Hemiptera: Coleorrhyncha: Peloridiidae). Environ Microbiol. 2013; 15:2031-2042. doi: 10.1111/1462-2920.12101.

58. Wilson AC, Duncan RP. Signatures of host/symbiont genome coevolution in insect nutritional endosymbioses. Proc Natl Acad Sci U S A. 2015; 112:10255-10261. doi: 10.1073/pnas.1423305112.

59. Thao ML, Baumann P. Evolutionary relationships of primary prokaryotic endosymbionts of whiteflies and their hosts. Appl Environ Microbiol. 2004; 70:3401-3406. PMID: 15184137.

60. Zchori-Fein E, Brown JK. Diversity of prokaryotes associated with *Bemisia tabaci* (Gennadius) (Hemiptera: Aleyrodidae). Ann Entomol Soc Am. 2002; 95:711-718. doi: http://dx.doi.org/10.1603/0013-8746(2002)095[0711:DOPAWB]2.0.CO;2.

61. Ahmed MZ, Ren S, Xue X, Li XX, Jin G, Qiu BL. Prevalence of endosymbionts in Bemisia tabaci populations and their in vivo sensitivity to antibiotics. Curr Microbiol. 2010; 61:322-328. doi: 10.1007/s00284-010-9614-5.

62. Gueguen G, Vavre F, Gnankine O, Peterschmitt M, Charif D, Chiel E, et al. Endosymbiont metacommunities, mtDNA diversity and the evolution of the *Bemisia tabaci* (Hemiptera: Aleyrodidae) species complex. Mol Ecol. 2010; 19: 4365-4378. doi: 10.1111/j.1365-294X.2010.04775.x.

63. Bing XL, Yang J, Zchori-Fein E, Wang XW, Liu SS. Characterization of a newly discovered symbiont of the whitefly *Bemisia tabaci* (Hemiptera: Aleyrodidae). Appl Environ Microb. 2013; 79:569-575. doi: 10.1128/AEM.03030-12.

64. Santos-Garcia D, Vargas-Chavez C, Moya A, Latorre A, Silva FJ. Genome evolution in

the primary endosymbiont of whiteflies sheds light on their divergence. Genome Biol Evol. 2015; 7:873-888. doi: 10.1093/gbe/evv038.

65. Moran NA, Jarvik T. Lateral transfer of genes from fungi underlies carotenoid production in aphids. Science. 2010; 328:624-627. doi: 10.1126/science.1187113.

66. Husnik F, Nikoh N, Koga R, Ross L, Duncan RP, Fujie F, et al. Horizontal gene transfer from diverse bacteria to an insect genome enables a tripartite nested mealybug symbiosis. Cell. 2013; 153:1567-1578. doi:10.1016/j.cell.2013.05.040.

67. De Gregorio E, Spellman PT, Tzou P, Rubin GM, Lemaitre B. The Toll and Imd pathways are the major regulators of the immune response in *Drosophila*. EMBO J. 2002; 21:2568-2579. doi: 10.1093/emboj/21.11.2568.

68. Munson MA, Baumann P, Kinsey MG. *Buchnera gen. nov.* and *Buchnera aphidicola sp. nov.*, a taxon consisting of the mycetocyte-associated, primary endosymbionts of aphids. Int J Syst Bacteriol. 1991; 41:566-568. doi: 10.1099/00207713-41-4-566.

69. Gerardo NM, Altincicek B, Anselme C, Atamian H, Barribeau SM, de Vos M, et al. Immunity and other defenses in pea aphids, *Acyrthosiphon pisum*. Genome Biol. 2010; 11:R21. doi: 10.1186/gb-2010-11-2-r21.

70. Misof B, Liu S, Meusemann K, Peters RS, Donath A, Mayer C, et al. Phylogenomics resolves the timing and pattern of insect evolution. Science. 2014; 346:763-767. doi: 10.1126/science.1257570.

71. Chu D, Zhang YJ, Brown JK, Cong B, Xu BY, Wu QJ, et al. The introduction of the exotic Q biotype of *Bemisia tabaci* from the Mediterranean region into China on ornamental crops. Fla Entomol. 2006; 89:168-174. doi: http://dx.doi.org/10.1653/0015-4040(2006)89[168:TIOTEQ]2.0.CO;2.

72. Tao Q, Wang A, Zhang HB. One large-insert plant-transformation-competent BIBAC library and three BAC libraries of Japonica rice forgenome research in rice and other

grasses. Theor Appl Genet. 2002; 105:1058-1066. doi: 10.1007/s00122-002-1057-3.

73. Li R, Fan W, Tian G, Zhu H, He L, Cai J, et al. The sequence and de novo assembly of the giant panda genome. Nature. 2010; 463:311-317. doi:10.1038/nature08696.

74. You M, Yue Z, He W, Yang X, Yang G, Xie M, et al. A heterozygous moth genome provides insights into herbivory and detoxification. Nat Genet. 2013; 45:220-225. doi:10.1038/ng.2524.

75. Boetzer M, Henkel CV, Jansen HJ, Butler D, Pirovano W. Scaffolding pre-assembled contigs using SSPACE. Bioinformatics. 2011; 27:578-579. doi:10.1093/bioinformatics/btq683.

76. Benson G. Tandem repeats finder: a program to analyze DNA sequences. Nucleic Acids Res. 1999; 27:573-580. doi: 10.1093/nar/27.2.573.

77. Edgar RC, Myers EW. PILER: identification and classification of genomic repeats. Bioinformatics. 2005; 21:152-158. doi:10.1093/bioinformatics/bti1003.

78. Price AL, Jones NC, Pevzner PA. De novo identification of repeat families in large genomes. Bioinformatics. 2005; 21:351-358. doi:10.1093/bioinformatics/bti1018.

79. Xu Z, Wang H. LTR_FINDER: an efficient tool for the prediction of full-length LTR retrotransposons. Nucleic Acids Res. 2007; 35:265-268. doi: 10.1093/nar/gkm286.

80. Parra G, Bradnam K, Ning Z, Keane T, Korf I. Assessing the gene space in draft genomes. Nucleic Acids Res. 2009; 37:289-297. doi: 10.1093/nar/gkn916.

81. Birney E, Clamp M, Durbin R. GeneWise and Genomewise. Genome Res. 2004; 14:988-995. doi:10.1101/gr.1865504.

82. Burge C, Karlin S. Prediction of complete gene structures in human genomic DNA. J Mol Biol. 1997; 268:78-94. doi:10.1006/jmbi.1997.0951.

83. Stanke M, Keller O, Gunduz I, Hayes A, Waack S, Morgenstern B. AUGUSTUS: ab initio prediction of alternative transcripts. Nucleic Acids Res. 2006; 34**:** W435-W439. PMID: 16845043.

84. Wang XW, Luan JB, Li JM, Bao YY, Zhang CX, Liu SS. De novo characterization of a whitefly transcriptome and analysis of its gene expression during development. BMC genomics. 2010; 11:400. doi: 10.1186/1471-2164-11-400.

85. Ye XD, Su YL, Zhao QY, Xia WQ, Liu SS Wang XW. Transcriptomic analyses reveal the adaptive features and biological differences of guts from two invasive whitefly species. BMC genomics. 2014; 15:370. doi: 10.1186/1471-2164-15-370.

86. Su YL, Li JM, Li M, Luan JB, Ye XD, Wang XW, et al. Transcriptomic analysis of the salivary glands of an invasive whitefly. PLoS One. 2012; 7:e39303. doi:10.1371/journal.pone.0039303.

87. Elsik CG, Mackey AJ, Reese JT, Milshina NV, Roos DS, Weinstock GM. Creating a honeybee consensus gene set. Genome Biol. 2007; 8:R13. doi: 10.1186/gb-2007-8-1-r13.

88. Trapnell C, Williams BA, Pertea G, Mortazavi A, Kwan G, van Baren MJ, et al. Transcript assembly and quantification by RNA-Seq reveals unannotated transcripts and isoform switching during cell differentiation. Nat Biotechnol. 2010; 28:511-515. doi: 10.1038/nbt.1621.

89. Kanehisa M, Goto S. KEGG: kyoto encyclopedia of genes and genomes. Nucleic Acids Res. 2000; 28:27-30. doi: 10.1093/nar/28.1.27.

90. Bairoch A, Apweiler R. The SWISS-PROT protein sequence database and its supplement TrEMBL in 2000. Nucleic Acids Res. 2000; 28:45-48. doi: 10.1093/nar/28.1.45.

91. Zdobnov EM, Apweiler R. InterProScan--an integration platform for the signature-recognition methods in InterPro. Bioinformatics. 2001; 17:847-848. doi:10.1093/bioinformatics/17.9.847.

92. Li H, Coghlan A, Ruan J, Coin LJ, Hériché JK, Osmotherly L, et al. TreeFam: a curated database of phylogenetic trees of animal gene families. Nucleic Acids Res. 2006; 34: 572-580. doi: 10.1093/nar/gkj118.

93. Ruan J, Li H, Chen Z, Coghlan A, Coin LJ, Guo Y, et al. TreeFam: 2008 update. Nucleic Acids Res. 2008; 36:735-740. doi: 10.1093/nar/gkm1005.

94. Guindon S, Dufayard JF, Lefort V, Anisimova M, Hordijk W, Gascuel O. New algorithms and methods to estimate maximum-likelihood phylogenies: assessing the performance of PhyML 3.0. Syst Biol. 2010; 59:307-321. doi: 10.1093/sysbio/syq010.

95. Benton MJ, Donoghue PC. Paleontological evidence to date the tree of life. Mol Biol Evol. 2007; 24:26-53. doi: 10.1093/molbev/msl150.

96. Donoghue PCJ, Benton MJ. Rocks and clocks: calibrating the Tree of Life using fossils and molecules. Trends Ecol Evol. 2007; 22:424-431. doi: 10.1016/j.tree.2007.05.005.

97. Yang Z. PAML: a program package for phylogenetic analyses by maximum likelihood. Comp Appl BioSci. 1997; 13:555-556. doi: 10.1099/0022-1317-79-8-1951.

98. Yang Z. PAML 4: phylogenetic analysis by maximum likelihood. Mol Biol Evol. 2007; 24:1586-1591. doi: 10.1093/molbev/msm088.

99. De Bie T, Cristianini N, Demuth JP, Hahn MW. CAFE: a computational tool for the study of gene family evolution. Bioinformatics. 2006; 22:1269-1271. doi:10.1093/bioinformatics/btl097.

100. Zhang J, Nielsen R, Yang Z. Evaluation of an improved branch-site likelihood method for detecting positive selection at the molecular level. Mol Biol Evol. 2005; 22:2472-2479. doi: 10.1093/molbev/msi237.

101. Loytynoja A, Goldman N. Phylogeny-aware gap placement prevents errors in sequence alignment and evolutionary analysis. Science. 2008; 320:1632-1635. doi: 10.1126/science.1158395.

102. Talavera G, Castresana J. Improvement of phylogenies after removing divergent and ambiguously aligned blocks from protein sequence alignments. Syst Biol. 2007; 56:564-577. doi: 10.1080/10635150701472164.

103. Tamura K, Stecher G, Peterson D, Filipski A, Kumar S. MEGA6: molecular evolutionary genetics analysis version 6.0. Mol Biol Evol. 2013; 30:2725-2729. doi: 10.1093/molbev/mst197.

104. Cao X, He Y, Hu Y, Wang Y, Chen YR, Bryant B, et al. The immune signaling pathways of *Manduca sexta*. Insect Biochem Mol Biol. 2015; 62:64-74. doi:10.1016/j.ibmb.2015.03.006.

105. Waterhouse RM, Kriventseva EV, Meister S, Xi Z, Alvarez KS, Bartholomay LC, et al. Evolutionary dynamics of immune-related genes and pathways in disease-vector mosquitoes. Science. 2007; 316:1738-1743. doi: 10.1126/science.1139862.

106. Katoh K, Standley DM. MAFFT multiple sequence alignment software version 7. improvements in performance and usability. Mol Biol Evol. 2013; 30:772-80. doi: 10.1093/molbev/mst010.

107. Li R, Xie W, Wang S, Wu Q, Yang N, Yang X, et al. Reference gene selection for qRT-PCR analysis in the sweetpotato whitefly, *Bemisia tabaci* (Hemiptera: Aleyrodidae). PLoS One. 2013; 8: e53006. doi.org/10.1371/journal.pone.0053006.

108. Livak KJ, Schmittgen TD. Analysis of relative gene expression data using real-time quantitative PCR and the $2^{-\Delta\Delta CT}$ method. Methods. 2001; 25: 402-408. doi:10.1006/meth.2001.1262.

Additional files

Supporting Figures

Figure S1. Schematic illustration of the assembly pipeline for *B. tabaci* Q genome based on the combined assemblies from WGS and BACs.

Figure S2. Gene family expansion and contraction in *B. tabaci* Q genome compared to other arthropods.

Figure S3. Phylogenetic trees for 11 horizontally transferred genes (HGTs).

Figure S4. Estimated divergence times among insect genomes using PAML *mcmctree.*

Figure S5. The RNA interference efficiency of nine CYP450 and three GST gene. mRNA levels of these genes were quantified by qRT-PCR in 96 hours of feeding on a diet containing dsEGFP and dsCYP450/dsGST. The mRNA levels are shown as a ratio relative to the levels for the reference gene (EF1α). Values are means ± SEMs (n=3).

Supporting Tables

Table S1. Statistics of the whole genome sequencing data

Table S2. Repeat Masker analysis in 4 Hemiptera species

Table S3. Evidenced use within GLEAN MED/Q protein-coding genes

Table S4. Summary of GLEAN gene models

Table S5. Functional annotation of the MED/Q genome

Table S6. Orthologous gene comparison among genomes of 14 arthropod species

Table S7. Gene ontologies for gene families showing expansion on *Bemisia tabaci* branch (FDR<0.05, p<=0.00097087378641)

Table S8. Results of gene family expansion (gene gain) and contraction (gene loss) analysis

Table S9. Gene ontology over-representation of gene families contracted on *Bemisia tabaci* branch (FDR<0.05, p<=0.000572390572)

Table S10. Immune system-related and virus transport genes in phloem- and blood-feeding insects

Table S11. Genes involved in B vitamin biosynthesis in MED/Q

Table S12. Genes involved in B vitamin biosynthesis in *Candidatus Hamiltonella* defense

Table S13. Horizontally transferred genes involved in amino acid biosynthesis in MED/Q

Table S14. Comparison of transaminases in three symbiotic systems *Bemisia tabaci*/Portiera, *Acyrthosiphum pisum*/Buchnera and *Nilaparvata lugens*/Yeast-like

Table S15. Quality control of assembled genome

Table S16. Primers used in this study.

Click here to download Table Table 1.docx

Table 1. Statistics of genome assembly and annotation for MED/Q

Assembled genome size (Mb)	658
Number of scaffolds (>100 bp)	5,003
Total size of assembled scaffolds	658,367,382 bp
N50 (scaffolds)	436,791 bp
Longest scaffold	2,857,362 bp
Number of contigs (>100 bp)	30,873
Total size of assembled contigs	638,748,832 bp
N50 (contigs)	44,388
Longest contig	362,835 bp
GC content	0.38
Number of GLEAN gene models	20,786
Mean transcript length	10,043 bp
Mean coding sequence length	1,504 bp
Mean number of exons per gene	5.12
Mean exon length	294 bp
Mean intron length	1,963 bp
Total size of TEs	265,205,801 bp
TEs proportion of the genome	0.4029

Figure 1

Click here to download Figure Fig1.tif

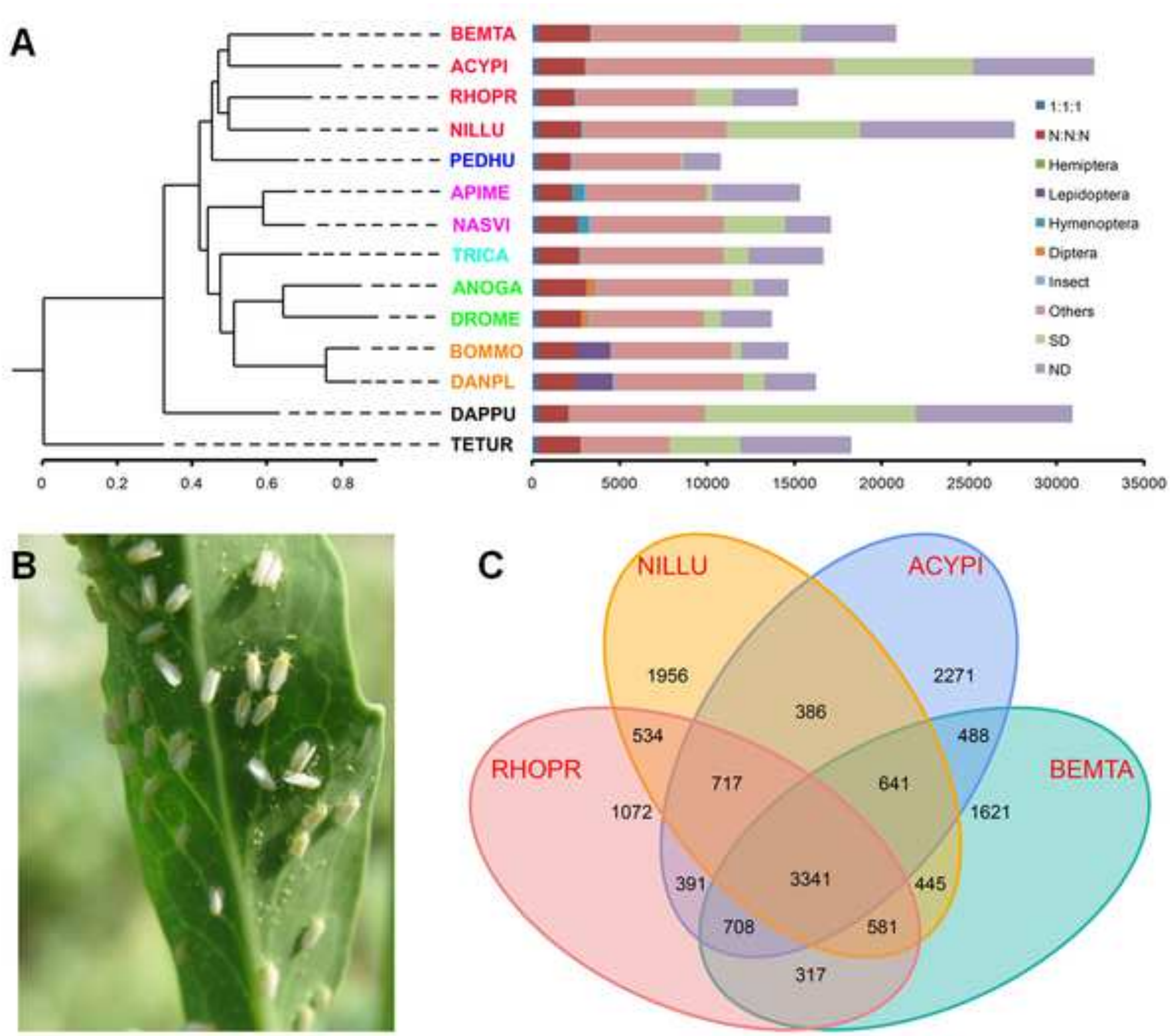

Figure 2

Click here to download Figure Fig2.tif

A

	P450	UGT	GST	ABC	COE	Total	
BEMTA*	153	63	21	59	51	347	Phloem feeding
ACYPI*	83	58	32	71	37	281	Phloem feeding
NILLU*	65	23	9	57	56	210	Phloem feeding
RHOPR	102	15	7	55	49	228	Blood feeding
PEDHU	39	4	12	38	20	113	Blood feeding
ANOGA	115	26	36	59	48	284	Blood feeding
DROME	85	34	32	53	35	239	
APIME	54	12	11	41	29	147	
NASVI	96	23	19	51	46	235	
TRICA*	126	28	30	13	51	248	
BOMMO*	72	33	27	55	89	276	

B

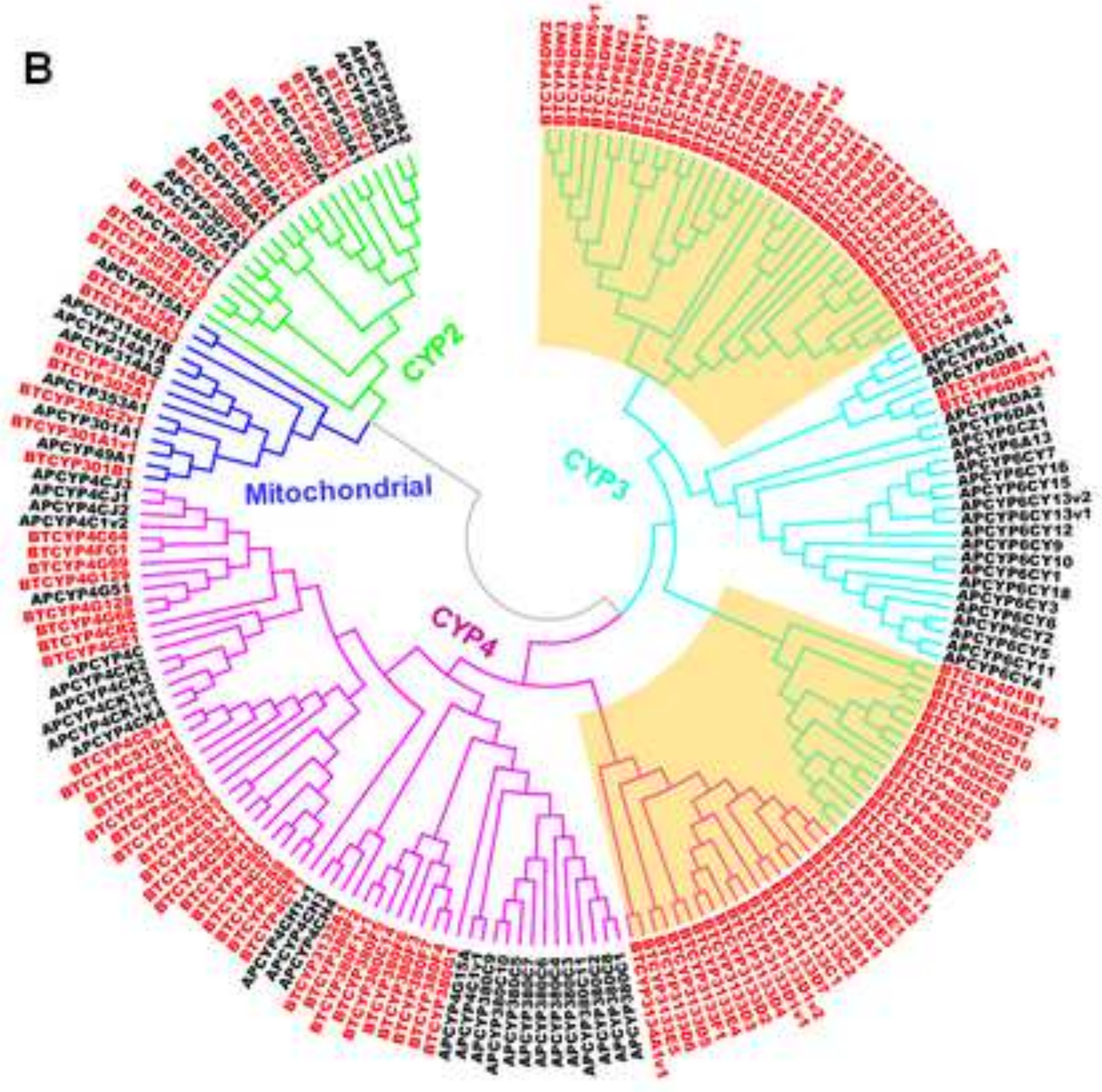

Figure 3

Click here to download Figure Fig3_updated.tif

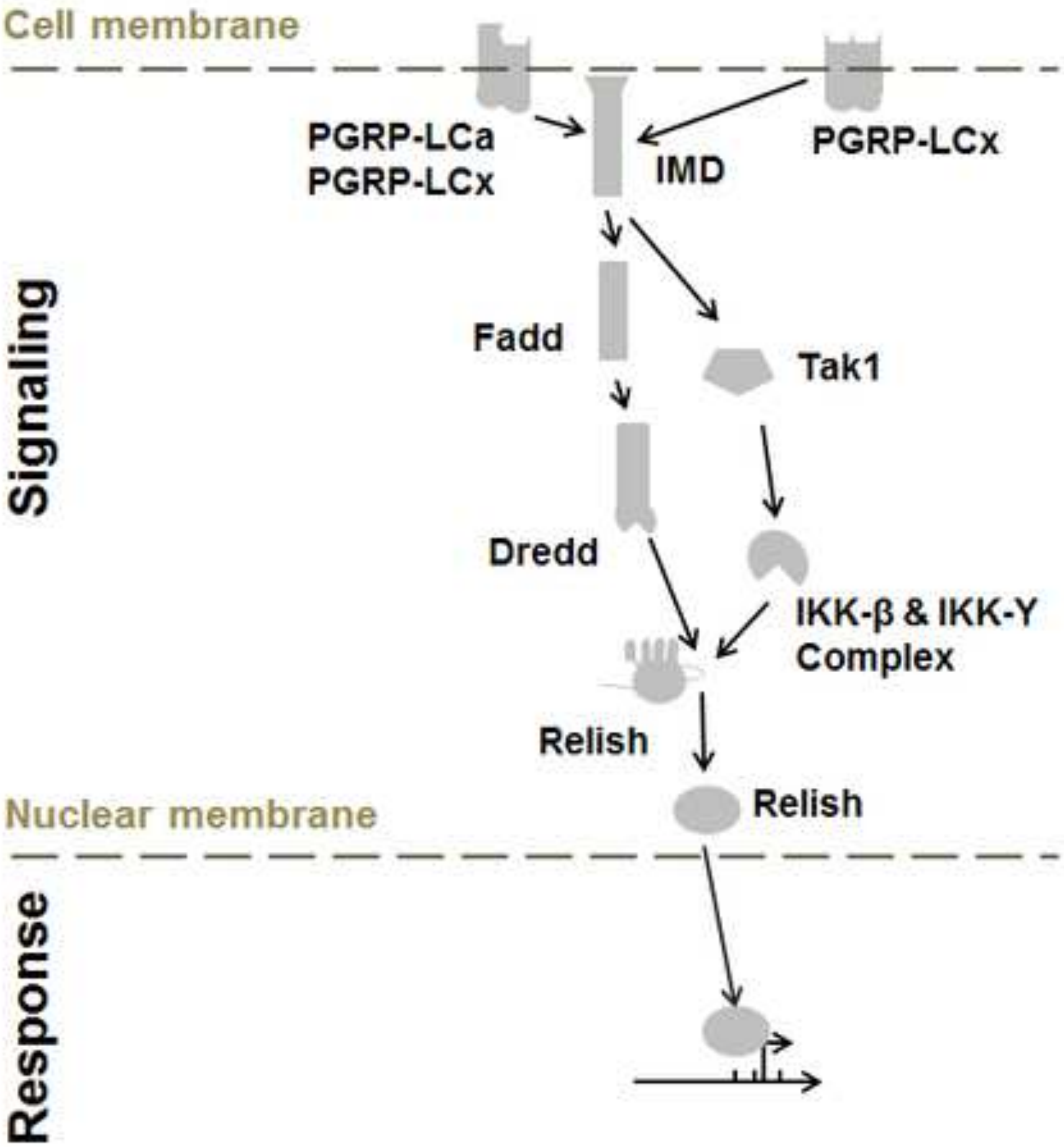

	Bt	Ap	NI
PGRP-LCa	0	0	0
PGRP-LCx	0	0	1
IMD	0	0	1
Fadd	0	0	0
Dredd	0	0	1
Tak1	0	0	1
IKK-β	0	1	1
IKK-Y	0	0	1
Relish	0	0	1
Relish	0	0	1

Figure 4

Click here to download Figure Fig4.tif

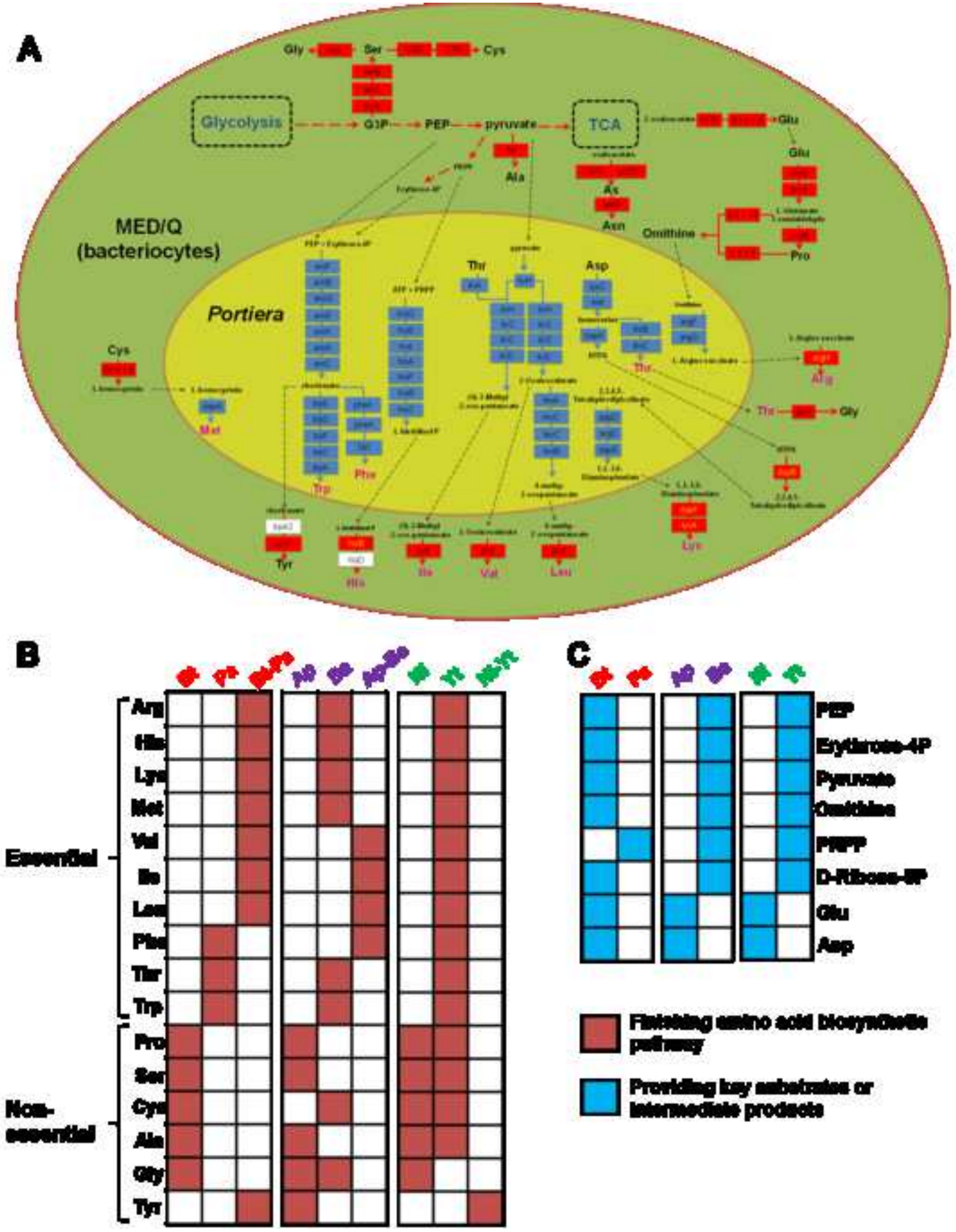

Figure 5

Click here to download Figure Fig5.tif

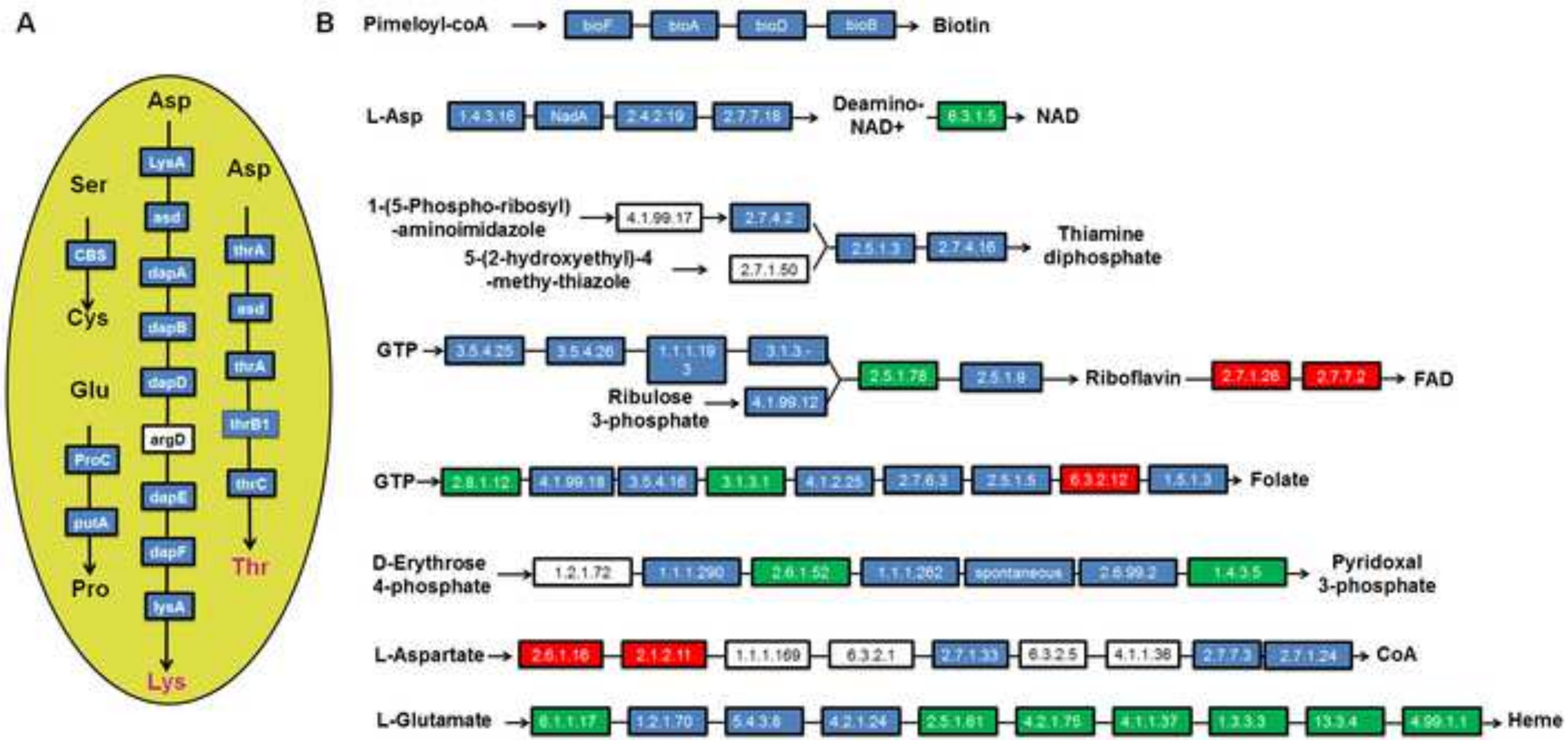

Figure 6

Click here to download Figure Fig6.TIF

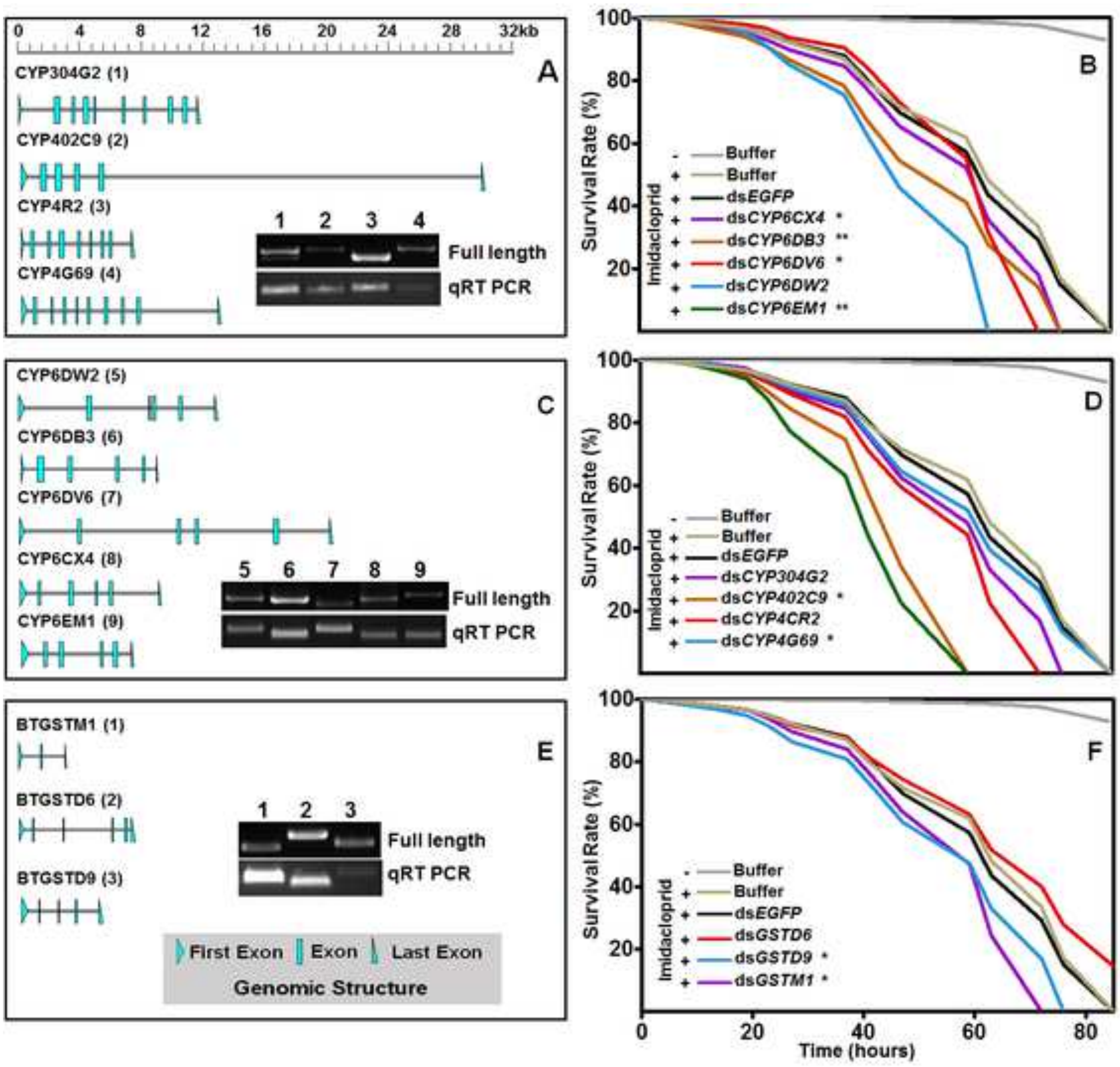

Figure S1

Click here to access/download
Supplementary Material
S1_Fig.tif

Figure S2

Click here to access/download
Supplementary Material
S2_Fig.tif

Figure S3

Click here to access/download
Supplementary Material
S3_Fig.tif

Figure S4

Click here to access/download
Supplementary Material
S4_Fig.tif

Figure S5

Click here to access/download
Supplementary Material
S5_Fig.tif

Table S1

Click here to access/download
Supplementary Material
S1_Table.docx

Table S2

Click here to access/download
Supplementary Material
S2_Table.docx

Table S3

Click here to access/download
Supplementary Material
S3_Table.docx

Table S4

Click here to access/download
Supplementary Material
S4_Table.docx

Table S5

Click here to access/download
Supplementary Material
S5_Table.docx

Table S6

Click here to access/download
Supplementary Material
S6_Table.docx

Table S7

Click here to access/download
Supplementary Material
S7_Table.docx

Table S8

Click here to access/download
Supplementary Material
S8_Table.docx

Table S9

Click here to access/download
Supplementary Material
S9_Table.docx

Table S10

Click here to access/download
Supplementary Material
S10_Table.docx

Table S11

Click here to access/download
Supplementary Material
S11_Table.docx

Table S12

Click here to access/download
Supplementary Material
S12_Table.docx

Table S13

Click here to access/download
Supplementary Material
S13_Table.docx

Table S14

Click here to access/download
Supplementary Material
S14_Table.docx

Table S15

Click here to access/download
Supplementary Material
S15_Table.doc

Table S16

Click here to access/download
Supplementary Material
S16_Table.docx

Personal Cover
Click here to download Personal Cover Response to Reviewers_Xie et al_11-27-2016_JZ.docx

UNIVERSITY OF KENTUCKY

College of Agriculture
Department of Entomology
Office of the State Entomologist
S-225 Agriculture Science Center-N
Lexington, KY 40546-0091

RE: MS No. GIGA-D-16-00061

December 8 2016

Hans Zauner
Assistant Editor
GigaScience

Dear Hans:

Below, please find our descriptions of revisions to manuscript GIGA-D-16-00061 entitled "The invasive Q-type *Bemisia tabaci* genome: a tale of gene loss and gene gain". We have followed the vast majority of reviewer suggestions, and have no absolute disagreements/rebuttals to any reviewer comments. As a result, we now submit what is hopefully a significantly improved manuscript that provides a more convincing depiction of our results and conclusions. We are hopeful that the work will now meet your standards for publication as a "Research Article".

EDITOR'S COMMENTS

Your manuscript "The invasive Q-type *Bemisia tabaci* genome: a tale of gene loss and gene gain" (GIGA-D-16-00061) has been assessed by two reviewers. Although it is of interest, we are unable to consider it for publication in its current form. The reviewers have raised a number of points which we believe would improve the manuscript and may allow a revised version to be published in GigaScience.

Reviewer 2, Denis Tagu, feels the genome data are of interest, but more biological experiments would be necessary to test hypotheses. Along the same lines, referee 1, Laura Boykin, feels the manuscript lacks defined scientific questions.

If you are confident that you can provide sufficient additional biological data to convince the referees, we would be able to consider a revised manuscript as "Research Article".

However, you may prefer to revise the submission as a "Data Note" - see http://gigascience.biomedcentral.com/submission-guidelines/preparing-your-manuscript/data-note

If you decide to revise your manuscript as a "Data Note", you should concentrate on providing a useful dataset, transparently described in detail for future users and the research community.

In any case, please also include BUSCO analysis in a revised manuscript, as suggested by Denis Tagu.

I notice that Laura Boykin refers to the nomenclature issues surrounding the *B. tabaci* species complex. I do not think that this controversial topic should be at the forefront of a Gigascience article, which is about the genome sequence. Nevertheless, I feel that it is appropriate to cite and briefly outline the relevant recent literature dealing with *B. tabaci* nomenclature. Our readers

College of Agriculture
Department of Entomology
Office of the State Entomologist
S-225 Agriculture Science Center-N
Lexington, KY 40546-0091

should get a brief, but complete and unbiased overview of the published arguments that have been put forward in this debate.

The reports are below. Please also take a moment to check our website at http://giga.edmgr.com/ for any additional comments that were saved as attachments.

If you are able to fully address these points, we would encourage you to submit a revised manuscript to GigaScience. Please note that we will seek further advice from referees before making a decision on your revised submission.

Once you have made the necessary corrections, please submit online at:

http://giga.edmgr.com/

If you have forgotten your username or password please use the "Send Login Details" link to get your login information. For security reasons, your password will be reset.

Please include a point-by-point within the 'Response to Reviewers' box in the submission system. Please ensure you describe additional experiments that were carried out and include a detailed rebuttal of any criticisms or requested revisions that you disagreed with. Please also ensure that your revised manuscript conforms to the journal style, which can be found in the Instructions for Authors on the journal homepage.

The due date for submitting the revised version of your article is 08 Dec 2016.

I look forward to receiving your revised manuscript soon.

Best wishes,

Hans Zauner
GigaScience
www.gigasciencejournal.com

RESPONSE: Reviewer and editor's constructive criticisms and suggestions are well received. Based on Drs Denis Tagu and Laura Boykin's suggestions, additional empirical experiments have been carried out to examine the hypotheses derived from this genome sequencing effort and to address a specific biological question. Generally, most of the suggested revisions involving minor changes have been incorporated into the revised manuscript. The following is a point-to-point response to reviewers' comments:

UNIVERSITY OF KENTUCKY

College of Agriculture
Department of Entomology
Office of the State Entomologist
S-225 Agriculture Science Center-N
Lexington, KY 40546-0091

REVIEWERS' COMMENTS

Reviewer: 1
The article needs to be greatly improved but very important data. After reading the article I'm left asking myself- why did the team sequence the genome?

The title is using incorrect nomenclature for this species complex. All references to biotype are obsolete. Please see the literature below and revise.

Key literature surrounding the nomenclature of the species complex are missing. References 9 and 10 are outdated. I recommend a complete literature search of the topic but read

1. Wang, H.L., J. Yang, L. M. Boykin, Q.Y. Zhao, Y.J. Wang, S.S. Liu, X.W. Wang. 2014. Development, characterization and analysis of microsatellite markers from the transcriptomes of three whitefly *Bemisia tabaci* species. Scientific Reports. doi:10.1038/srep06351.
2. Tay, W.T., G. A. Evans, L.M. Boykin, P.J. De Barro. 2012. Will the real *Bemisia tabaci* please stand up? PLoS ONE 7(11): e50550. doi:10.1371/journal.pone.0050550.
3. Boykin, L.M., K.F. Armstrong, L. Kubatko, and P. De Barro. 2012. Species Delimitation and Global Biosecurity. Evolutionary Bioinformatics 8: 1-37.
4. Boykin, L.M. 2014. *Bemisia tabaci* nomenclature: Lessons learned. Pest Management Science 70:1454-59.

Avoid the "sibling" species terminology. Remove all reference to biotype throughout the manuscript. What is "strain selection"? Later in the paper MED/Q is used. Be consistent with the naming.
RESPONSE: We echo Dr. Laura Boykin’s sentiments about the nomenclature of the *Bemisia tabaci* species complex. According to her suggestion, we eliminated “biotype” throughout the manuscript and replaced “sibling” with “cryptic” species. “Strain selection” has been removed to avoid confusion.

As for the references, we included all four publications recommended by the reviewer. The debate over *Bemisia tabaci* as a complex species or species complex has been over a half century. To acknowledge this part of the history, and also to show “MED/Q was inadvertently introduced into several geographic locations worldwide, and became established throughout China”, we would like to not exclude the references referring B. *tabaci* as biotypes. References 9 and 10 (listed here) represent some of the major discoveries from our research group, and were published recently in well-respected peer-reviewed journals. The nomenclature in these publications is debatable; however, the contents are relevant and up-to-date.

9. **Pan HP, Preisser EL, Chu D, Wang SL, Wu QJ, Carriere Y, et al. Insecticides promote viral outbreaks by altering herbivore competition. Ecol Appl. 2015; 25:1585-1595. PMID: 26552266.**
10. **Liu BM, Yan FM, Chu D, Pan HP, Jiao XG, Xie W, et al. Multiple forms of vector manipulation by a plant-infecting virus: *Bemisia tabaci* and tomato yellow leaf curl virus. J Virol. 2013; 87:4929-37. doi:10.1128/JVI.03571-12.**

UNIVERSITY OF KENTUCKY

College of Agriculture
Department of Entomology
Office of the State Entomologist
S-225 Agriculture Science Center-N
Lexington, KY 40546-0091

The introduction does not properly review the literature or set up the read for the study that has been conducted or why it is important to have a genome for this particular *B. tabaci* species.

The discussion need to be rewritten completely. There are no scientific questions that were set out to be answered with this genome paper. I recommend reading: http://bfg.oxfordjournals.org/content/early/2016/06/22/bfgp.elw026.long and paying attention to the reference: http://www.sciencedirect.com/science/article/pii/S1471492214000762

The days of "sequence-first-ask-questions-later" are over and this paper needs to be greatly improved with well defined research questions relevant to *Bemisia tabaci* species before it can be published anywhere.
RESPONSE: Based on reviewer's suggestion, we carried out additional experiments to address specific biological questions. We totally agree that genome sequencing should have clear biological purposes. With the additional RNAi-based functional data, we elected "insecticide resistance" as the focal point to reorganize and rewrite the discussion in the revised manuscript.

Reviewer: 2
I have reviewed this *Bemisia* genome paper with interest: this is a long time that the community is expecting the release of the genome of this Hemipteran pest, and I am satisfied to see that a consortium tackled the difficulty. This is a regular genome paper whose aim I guess is to provide basic data of an annotated genome and a few analyses. There is thus an interest to publish it, if the community has access to a well-structured genome database of *B. tabacci*, so that the community will still improve annotation and provide new knowledge with other analyses. My first recommendation is thus to provide this access, more than from NBCI. I suggest the authors to contact the i5k community who developed a dedicated database for insects, with a nice interface allowing search, blast and web Apollo annotation (I am not member of this i5k database!).
RESPONSE: To share the genome information using i5K database platform is a great idea, we will certainly contact i5K community after the conclusion of this publication. In the meantime, we have already uploaded the genome sequence onto NCBI and GigaDB. NCBI maintains genome sequences of plants, animals and microbes, and can be readily accessed through user-friendly interfaces, including blast, Map Viewer, and CD Tree. GigaDB contains 268 discoverable, tractable and citable databases that are available for public download and use. Therefore, we are comfortable using these two databases to share *B. tabacci* genomic resources.

As I said before, the general analyses are global, and centered on specific gene families such as detoxification (in relation to insecticide resistance and host plant interactions) and immune system (in relation to endosymbiont relationship). There are thus many other gene families that would deserve analyses but I understand that this might not be essential for the paper. But as the paper focuses on a small number of family genes, I would expect more biological

College of Agriculture
Department of Entomology
Office of the State Entomologist
S-225 Agriculture Science Center-N
Lexington, KY 40546-0091

experiments that would allow testing some of the hypotheses suggested by the authors. For instance, authors could provide some RNA expression data of candidate genes (e.g. P450) on different host plants or insecticides, or from different Bemisia populations with others insecticide resistance profiles. Or some experiments on the IMD pathways such as the one provided for the A. pisum paper. I don't say the authors should provide all these analyses, but at least put more biological data.
RESPONSE: Based on Dr Denis Tagu's suggestion, we selected one of the main interests from our group, insecticide resistance, as the focal point for the discussion section. A total of 12 genes encoding detoxification enzymes, including 9 P450s and 3 GSTs, were subjected to RNAi-based functional validation studies to investigate their potential involvement in the imidacloprid resistance.

The hypothesis of HGT is also interesting, but it is known that final demonstration is complicated. So to lower the fact that this is an HGT. It could be, but this remains to be demonstrated. please revise a bit the text
RESPONSE: Based on reviewer's suggestion, we toned down the HGT hypothesis.

Another trait of *Bemisia* is the transmission of plant viruses, as the authors several times mention it in the text. I would expect some gene family analysis of proteins that possibly play roles in virus transport (vesicle processes?).
RESPONSE: Most recently, a group at the Cornell University published *Bemisia* B genome (Chen et al., 2016) with a focus on the genomic signatures contributing to virus transmission. We cited this work in the revised manuscript.

Chen et al. 2016. The draft genome of whitefly *Bemisia tabaci* MEAM1, a global crop pest, provides novel insights into virus transmission, host adaptation, and insecticide resistance. BMC Biology.

The text needs strong English editing. Some parts are OK, but others are different to follow. I suggest the English-native co-authors carefully check all the manuscript, including figure and table legends.
RESPONSE: Revisions have been made according to reviewer's suggestion.

Other minor points:
Does the strain that have been sequenced disseminate plant viruses?
RESPONSE: Yes, *B. tabaci Q* is notorious for its ability to transmit plant viruses.

Males are haploid. For Hymenoptera genome projects, males are usually used for sequencing in order to get rid off heterozygocity. I am not a specialist of whitefly biology, but why did not you use only male individuals for this genome project?
RESPONSE: We are aware of the strategies to minimizing the heterozygocity. However, for *B. tabaci, it is* difficult to distinguish the adult sex with the naked eyes, its size is too small to generate enough genetic materials individually for the genome sequencing. We, therefore, used unsexed, mass collection of *B. tabaci* adults for the sequencing.

UNIVERSITY OF KENTUCKY

College of Agriculture
Department of Entomology
Office of the State Entomologist
S-225 Agriculture Science Center-N
Lexington, KY 40546-0091

The authors used CEGMA for quality control of sequencing and assembly. I would suggest using BUSCO which proposed a larger set of conserved proteins for Insects or Arthropods. The authors will thus have a better assessment of their genome I guess.
RESPONSE: Based on reviewer's suggestion, we used BUSCO to evaluate the quality of *B. tabaci* genome, gene set, and transcriptome. There are 2,675 conserved arthropod proteins in total. As shown in the following table, we have detected 79% complete and fragmented BUSCOs in *B. tabaci* genome, 88% in gene set, and 70% in transcriptome.

Pattern	Genome		Gene set		Transcriptome	
	Number	%	Number	%	Number	%
Complete BUSCOs	1399	52%	2105	78%	1276	47%
Complete and single-copy BUSCOs	1118	42%	1434	54%	985	10%
Complete and duplicated BUSCOs	281	10%	671	25%	291	11%
Fragmented BUSCOs	706	26%	257	9.6%	595	22%
All (Complete and Fragmented)	2105	79%	2362	88%	1871	70%
Missing BUSCOs	570	21%	313	11%	804	30%

Based on this assessment, we would like to stay with the CEGMA analysis in this manuscript.

The authors could check within the non-assembled reads whether some missing genes that are not present in the assembly might be there, or even other bacterial sequences/genomes.
RESPONSE: 1) We mapped all WGS clean data covering different insert libs from 170bp to 40kb into *B. tabaci* MED/Q-type genome by SOAPaligner/soap2 V2.21t. Then non-assembled reads were filtered to assemble through software SOAPdenovo, and a draft sequence with 385Mb genome size was constructed. 2) In order to check whether any sequence existed in previous *B. tabaci* MED/Q-type genome, the 385Mb assembled sequence was again aligned to *B. tabaci* MED/Q-type genome with software blast and filtered 65Mb unmapped-assembled sequences (333,000 sequences). 3) Functional analysis was utilized to analyze the composition of these unmapped sequences through mapping them to NT database by software blast. On one hand, we mapped these unmapped-assembled sequences into the transcript sequences to search EST sequences by software blat. Secondly, a homolog based alignment with *Acyrthosiphon pisum* was ran by software Blast and Genewise. And then, we merged the gene set though Glean and got nine genes. Finally, we aligned these nine genes into *B. tabaci* MED/Q-type coding genes and into *B. tabaci* MED/Q-type genome by blast. Results shown four genes were successfully mapped into the *B. tabaci* MED/Q coding genes, and five not. That is, we got five unmapped genes, which might be missing gene in the present assemble. Functional analysis was again to search their function in NR database from NCBI by blast, and all these five genes were all annotated as gene indeed belong to *B. tabaci* (Tables 1 and 3). One the other hand, we found these unmapped-assembled sequences mainly include bacteria sequences from the following species (Table 2).

UNIVERSITY OF KENTUCKY

College of Agriculture
Department of Entomology
Office of the State Entomologist
S-225 Agriculture Science Center-N
Lexington, KY 40546-0091

Table 1. Function analysis with NR database of missing genes

Gene ID	Description	Max score	Total score	Query cover	E value	Identity	Accession
Gene1	PREDICTED: rho GTPase-activating protein 190 isoform X1 [*Bemisia tabaci*]	179	179	0.81	2E-50	0.97	XP_018903139.1
Gene2	PREDICTED: DNA polymerase epsilon catalytic subunit A [*Bemisia tabaci*]	272	272	0.94	5E-82	0.98	XP_018915083.1
Gene5	PREDICTED: lysosomal alpha-glucosidase-like [*Bemisia tabaci*]	259	259	0.99	2E-83	0.99	XP_018911552.1
Gene6	PREDICTED: acetylcholinesterase [*Bemisia tabaci*]	209	209	1	2E-61	1	XP_018906011.1
Gene7	PREDICTED: MIF4G domain-containing protein isoform X2 [*Bemisia tabaci*]	106	106	1	3E-26	1	XP_018899706.1

Table 2. The organisms of unmapped-assemble sequences

Organism	Mapped sequence number
Candidatus Hamiltonella	931
Pseudomonas fluorescens	928
Pseudomonas sp.	767
Pseudomonas trivialis	609
Flavobacterium johnsoniae	553
Methylotenera mobilis	236
Cardinium endosymbiont	183
Pseudomonas poae	69
Sphingobacterium sp.	60
Pseudomonas brassicacearum	42
Pseudomonas chlororaphis	34
Pseudomonas fragi	33
Pseudomonas mandelii	25
Pseudomonas putida	25
Chryseobacterium sp.	21

An Equal Opportunity University

UNIVERSITY OF KENTUCKY

College of Agriculture
Department of Entomology
Office of the State Entomologist
S-225 Agriculture Science Center-N
Lexington, KY 40546-0091

Table 3. Missing sequences

**>Gene7 locus=C60404577:235:387:-**
**ATGGACCAGATGCGCATTCAATTTTTAAAGCAAACCACTTCGCCTAGTTTTCGGAAAACTTTGCTG<br>CAAATGATCGAACTAAGAGCAAGCAAGTGGGCTCTTCCAGTTGAAAGTATTATTTACTACTATCCT<br>AGCAATAGCCAAAAAAAGTAA**
**>Gene5 locus=C60410339:723:1079:+**
**ATGACATCAACTAATTGGGCGCTTGCTGATGAATGTGCCATTGTTCCAAATGAAGACAGATTTGAC<br>TGTTTCCCACGCGGACCTTCCAACGAATCGGTTTGTACTCAACGAGGCTGTTGCTGGAAACCCACT<br>GAGAGCCGCACTGAAATGGATGGATTTAAGAAACTGGATGTCCCTTGGTGTTACTACCCAGTTGCT<br>TTTAAGTCGTACGAGCAGGTGAATAAAACAGTAGCCAATCATGTCACCACTGTCTTTCTGAGAAAT<br>GTTATTAAGTCACCCTATCCTGATGATGTGCCTCTTCTCAGAATGGTCATCAAAGCGGAATCCAAC<br>TTCAGGGTTCATGTTAAGGTGAGTTAA**
**>Gene1 locus=C60412988:80:1241:+**
**ATGAATAATATAGATGTATTTTTCTACGTGCAATTTTTTCAATTTCTCTCCTTGCTTTTTCAGTCTGAC<br>TTCAGCGGAAGAGTCGTTAATAACGACCATTTCCTGTATTGGGGTGAAGTATCCAAAGCAACAGA<br>GGAAGGAGCGGAAATTCAGTTTCAAGTCATCGAGCAAACTGAATTCATCGATGACGCATCCTTTCA<br>GCCATTTAAAGGTGGCAAAATGGAGCCTTATATTAAACGATGTGCGGCAGTCAAGTTGACATCAG<br>CAGAGAAACTCATGTACATTTGTAAAAATCAATTAGGTAAGTTCAGATTGTTGTAA**
**>Gene2 locus=C60415092:393:1815:-**
**GCTAAAAAACAGGTTGCAGAAGCTCTTGAAAAAAATGATGCTGGAGAGATAAAATCTGCGAAAAA<br>TCGTGAAGTTTTGTATGACTCTTTACAAATAGCTCACAAATGCATCCTGAACTCTTTTTATGGTTAC<br>GTCATGCGGAAAGGCGCCAGATGGCATAGTATGGAGATGGCTGGAATAGTGTGTCACACTGGAG<br>CTAATATCATCACAAGAGCTAGAGAAATCATTGAAAAAGTTGGTCGACCACTTGAATTAGATACAG<br>ATGGTATTTGGTGTGTTTTACCTGCATCTTTTCCAGAAAATTATGTGATTAATTCCACACATCCGGG<br>CAAAAGTAAAATTACCATCTCTTACCCAAATGCTGTACTGAATTCCATGGTGAAGGTAATTTTTGTT<br>GTGGTACATATATGTTCTTGCTAA**
**>Gene6 locus=C60415266:261:957:+**
**ATGTTGTCAGACTTTCTCTTCAGAGCTCCTGTTGATCACATCGTAAAACTCCTCGTCAGTCAAGACG<br>TTCCTACATATATGTACGTCATGAATACAACGGTCGAAGCTTTGCGCCTGCCTGAATGGAGGAAAT<br>ACCCTCACAACATCGAACATTACTTCCTCACTGGAGCACCCTTCATGGACACAGAATTTTTCCCTC<br>CAAGCGCCCATCTTGAAAGGAATATGTGGACGGACAATGATAGGAACATGAGTCACTTTTTCATGA<br>AAGCTTATTCAAACTTTGCAAAATATGGGTAA**

Repetitive element analysis is a bit poor. No possibility to describe a bit more the different families of transposons?
RESPONSE: Revisions have been made according to reviewer’s suggestion.

The gene coverage section is short and difficult to follow (page 11 lines 10 and following).
RESPONSE: Revisions have been made according to reviewer’s suggestion (Table S15).

In the text, comparison of insect-symbionts system is very difficult to follow too.
RESPONSE: Revisions have been made according to reviewer’s suggestion (Figure 4).

Conclusion (at least as it is today) is not necessary: too long and redundant with the text.
RESPONSE: Revisions have been made according to reviewer’s suggestion.

UNIVERSITY OF KENTUCKY

College of Agriculture
Department of Entomology
Office of the State Entomologist
S-225 Agriculture Science Center-N
Lexington, KY 40546-0091

Figure 3: any possibility to put all the proteins present in the table within the figure/flow chart?
RESPONSE: Revisions have been made according to reviewer's suggestion (Figure 3).

Figure 4: I guess that the arrows showing the transfer of metabolites are not demonstrated but suggested by this work? Please mention it.
RESPONSE: Please see revised figure legend.

Figure 5: please improve the legends that are not clear and incomplete (e.g. what are the green boxes in 5B?).
RESPONSE: Revisions have been made according to reviewer's suggestion.

Figure S4, Table S3, Table S7, Table S9; not sure they are necessary
RESPONSE: Figure S4, Table S3, Table S7 and Table S9 supported the statements in the manuscript. We, therefore, would like to keep these figure and tables in the supplementary materials.

Please also take a moment to check our website at for any additional comments that were saved as attachments. Please note that as GigaScience has a policy of open peer review, you will be able to see the names of the reviewers.
RESPONSE: According to reviewer's suggestion, we carried out additional experiments, reorganized and rewrote the manuscript.

Thank you for your consideration and evaluation of this manuscript. We appreciate the opportunity to revise this manuscript for re-consideration, and we also thank the reviewers and editor for their careful review and constructive comments on the first manuscript draft.

With respect,

Xuguo "Joe" Zhou
Associate Professor, Ph.D.
Insect Integrative Genomics
Department of Entomology
University of Kentucky
E-Mail: xuguozhou@uky.edu

Phone: 859-257-3125
Fax: 859-323-1120
