# Supplementary material for: Genome sequencing of the sweetpotato whitefly Bemisia tabaci MED/Q
Source: Gigascience. 2017 Mar 15;6(5):1–7. doi: 10.1093/gigascience/gix018 (PMC5467035; doi:10.1093/gigascience/gix018)
Supplement: GIGA-D-16-00061_Revision_2.pdf [file gix018_GIGA-D-16-00061_Revision_2.pdf]

Manuscript
Click here to download Manuscript GigaScience_Note_2-3-17_JZ (1) (1).docx

Genome sequencing of the sweetpotato whitefly *Bemsia tabaci* MED/Q

Authors and affiliations

Wen Xie[1]¶, Chunhai Chen[2]¶, Zezhong Yang[1]¶, Litao Guo[1]¶, Xin Yang[1], Dan Wang[2], Ming Chen[2], Jinqun Huang[2], Yanan Wen[1], Yang Zeng[1], Yating Liu[1], Jixing Xia[1], Lixia Tian[1], Hongying Cui[1], Qingjun Wu[1], Shaoli Wang[1], Baoyun Xu[1], Xianchun Li[4], Xinqiu Tan[5], Murad Ghanim[6], Baoli Qiu[7], Huipeng Pan[7], Dong Chu[8], Helene Delatte[9], M. N. Maruthi[10], Feng Ge[11], Xueping Zhou[12], Xiaowei Wang[13], Fanghao Wan[12], Yuzhou Du[14], Chen Luo[15], Fengming Yan[16], Evan L. Preisser[17], Xiaoguo Jiao[18], Brad S. Coates[19], Jinyang Zhao[2], Qiang Gao[2], Jinquan Xia[2], Ye Yin[2]*, Yong Liu[5]*, Judith K. Brown[4]*, Xuguo "Joe" Zhou[3]*, Youjun Zhang[1]*

1 Institute of Vegetables and Flowers, Chinese Academy of Agricultural Science, Beijing 100081, China, **2** BGI-Shenzhen, Shenzhen 518083, China, **3** Department of Entomology, S-225 Agricultural Science Center North, University of Kentucky, Lexington, KY 40546-0091, USA, **4** School of Plant Sciences, University of Arizona, Tucson, AZ 85721, USA, **5** Institute of Plant Protection, Hunan Academy of Agricultural Sciences, Changsha 410125, China, **6** Department of Entomology, Volcani Center, Bet Dagan 5025001, Israel, **7** Key Lab of Bio-pesticide Creation and Application, South China Agricultural University, Guangzhou 510642, China, **8** College of Agronomy and Plant Protection, Qingdao Agricultural University, Qingdao 266109, China, **9** Cirad, UMR PVBMT, Saint-Pierre, La Re´union, France, **10** Natural Resources Institute, University of Greenwich, Chatham Maritime, Kent ME4 4TB, UK, **11** Institute of Zoology, Chinese Academy of Sciences, Beijing 100101, China, **12** Institute of Plant Protection, Chinese Academy of Agricultural Sciences, Beijing 100193, China, **13** Ministry of Agriculture Key Laboratory of Agricultural Entomology, Institute of Insect Sciences, Zhejiang University, Hangzhou 310058, China, **14** School of Horticulture and Plant Protection and Institute of Applied Entomology, Yangzhou University, Yangzhou 225009, China, **15** Institute of Plant and Environment Protection, Beijing Academy of Agriculture and Forestry Sciences, Beijing 100089, China, **16** Collaborative Innovation Center of Henan Grain Crops, College of Plant Protection, Henan Agricultural University, Zhengzhou 450002, China, **17** Department of Biological Sciences, University of Rhode Island, Kingston, Rhode Island 02881, USA, **18** College of Life Sciences, Hubei University, Wuhan 430062, China. **19** United States Department of Agriculture, Agricultural Research Service, Corn Insects & Crop Genetics Research Unit, Ames, IA 50011, USA.

¶These authors contributed equally to the work.

*To whom correspondence should be addressed. Email: Youjun Zhang (zhangyoujun@caas.cn), Ye Yin (yinye@genomics.cn), Xuguo "Joe" Zhou (xuguozhou@uky.edu), Judith K. Brown (JBrown@ag.arizona.edu), Yong Liu (haoasliu@163.com).

Abstract

Background: The sweetpotato whitefly *Bemisia tabaci* is a highly destructive agricultural and ornamental crop pest. It damages host plants through both phloem feeding and vectoring plant pathogens. Introductions of *B. tabaci* are difficult to quarantine and eradicate because of its high reproductive rates, broad host plant range, and insecticide resistance.

Findings: A total of 791 Gb of raw DNA sequence from whole genome shotgun sequencing, and 13 BAC pooling libraries were generated by Illumina sequencing using different combinations of mate-pair and pair-end libraries. Assembly gave a final genome with a scaffold N50 of 437 kb, and a total length of 658 Mb. Annotation of repetitive elements and coding regions resulted in 265.0 Mb TEs (40.3%) and 20,786 protein-coding genes with putative gene family expansions, respectively. Phylogenetic analysis based on orthologs across 14 arthropod taxa suggested that MED/Q is clustered into a hemipteran clade containing *A. pisum*, and is a sister lineage to a clade containing both *R. prolixus* and *N. lugens*. Genome completeness, as estimated using the CEGMA and BUSCO pipelines, reached 96% and 79%.

Conclusions: This MED/Q genomic resources lay a foundation for future 'pan-genomic' comparisons of invasive vs. non-invasive, invasive vs invasive, and native vs. exotic *Bemisia*, which, in return, will open up new avenues of investigation into whitefly biology, evolution, and management.

Keywords: Whitefly *Bemisia tabaci*, Genomics, Assembly, Annotation

Data Description

Samples and libraries construction

As a globally invasive species, the phloem-feeding whitefly *Bemisia tabaci* (Genn.; hereafter '*Bemisia*') has been found on all continents except Antarctica [1,2]. Taxonomically, *B. tabaci* is considered a species complex that contains several morphologically-indistinguishable but genetically-distinct 'cryptic species' [2-7]. The *Bemisia* Middle East-Asia Minor 1 (MEAM1, or 'B') cryptic species is highly invasive and has emerged as a major pest in the United States, Caribbean Basin, Latin America, Middle East [1], and East Asia [8]. Similarly, the invasive *Bemisia* Mediterranean (MED, or 'Q') cryptic species has been introduced into several geographic locations and become established throughout China [9,10]. Despite substantial research and published whitefly *Bemisia tabaci* MEAM1/B genome recently [11], however, the genetic or genomic basis of MED/Q remain obscure.

The MED/Q *Bemisia tabaci* adult whitefly females (2n) and males (1n) were initially collected from infested field-grown cucumber plants in Beijing, China during 2011, and used to establish a laboratory colony (MED/Q) at the Institute of Vegetable and Flowers, Chinese Academy of Agriculture Science by transferring adult males and females to caged pepper plants (10-12 leaf stage). Results of mtCOI gene PCR-RFLP assays [12], and direct DNA sequencing followed by phylogenetic evaluation against reference sequences [13] both confirmed that the *Bemisia* in the MED/Q colony belonged to the Q1 haplotype group, or western Mediterranean region clade (data not shown).

The MED/Q whitefly colony was used as the source initial short shotgun Illumina sequencing. Adult whiteflies fed using Parafilm ®membrane sachets containing a 25% sucrose solution for 48 hrs prior to collection of ~5,000 male and female adults (~ 50:50). Samples were immediately frozen in liquid nitrogen for three hours prior to transfer to a -80°C freezer. This genomic DNA was used to construct Illumina TruSeq paired end (PE) sequencing libraries (170, 250, 300, 500 and 800 bp insert sizes) and mate pair (MP) libraries (2, 5, 10, 20 and 40 KB in size) according to manufacturer instructions. Additionally, two Illumina PE sequencing libraries (~500bp and 800bp inserts) were constructed from whole genome amplification (WGA) reactions carried out on genomic DNA isolated from two adult male whiteflies. We also construct 13 BAC libraries with pooling of clones and Illumina library construction according to manufacturer instructions.

Genome sequencing and assembly

All libraries were sequenced on an Illumina Hiseq 2000 using 100 bp reads from both fragment ends, and raw data processed and assembled as shown (S1 Table; S1 Fig). Briefly, a series of filtering steps were performed on the raw reads to filter out the following: (1) reads with >10% Ns, more than 40% low-quality bases, more than 10 bp overlapping with adapter sequences, allowing no more than 3bp mismatches; (2) paired-end reads that overlapped more than 10 bp between two ends, with insert size larger than 200 bp libraries; and (3) duplicated reads generated by PCR amplification during the construction of the large-insert library. Filtered reads were used for K-mer determination within subsequent assembly steps. The frequency of each K-mer was calculated from the genome-sequence reads. K-mer frequencies along the sequence depth gradient follow a Poisson distribution in a given data set except for a high proportion at low frequency due to sequencing errors, as K-mers that contain such sequencing errors may be orphans among all splitting K-mers. The genome size, G, was estimated as $G = K\_num/K\_depth$, where K_num is the total number of K-mers, and K_depth is the maximal frequency. Initial contigs were assembled from filtered 500 and 800 bp insert-size WGA PE libraries using SOAPdenovo. The sequencing reads obtained for 2k-40kb MP libraries were used to connect the contigs and to generate the scaffolds as described

by Li et al. (2010) [14] with a K-mer size of 65.

Individual BAC pools were assembled independently using SOAPdenovo and the whole genome shotgun reads from PE and MP libraries were used to fill gaps in the BAC scaffolds. After sequencing, the raw reads were filtered as described above. In addition, reads representing contamination by *E. coli* or the plasmid vector were filtered. The pooled reads were separated according to the BAC-reads index, and each BAC was assembled using a combination of "hierarchical assembly" and "*de Bruijn* graph assembly". First, the reads linked to each BAC were assembled using SOAPdenovo [14], with various combinations of parameters with a K-mer range from 27 to 63 and a step size of 6. The assembly with the longest scaffold N50 was defined as the "best" for each BAC. The resulting BACs were mapped with the large shotgun MP read data to optimize the assembly for each BAC.

The final draft assembly was produced by integrating sequences that overlapped among the scaffolds independently assembled from genome shotgun and BAC reads, and in doing so eliminated the redundant scaffolds using the following steps. In order to integrate the two assemblies, the software *Rabbit* [15] was applied to identify any relationship between scaffolds, to connect the overlapping regions that shared at least 90% similarity, and to remove redundancy based on a 17-mer frequency. Finally, *SSPACE* [16] was used to construct super-scaffolds containing 800 bp-40 kb WGS reads, and the 170-800 bp genome shotgun read data were used to fill the gaps using *GapCloser* [14]. Post-assembly processing included removal of contaminating bacterial and viral DNA sequences, by aligning all assembled sequences to the genome sequences of viruses and bacteria, obtained from previous local BLASTn alignments and by NCBI upload filter. Aligned sequences that shared >90% identity and were >200 bp in size were filtered from the final assembly. The assembled sequences that were covered by at least one EST sequence were retained. Process read data was mapped the the draft MED/Q genome using *SOAPaligner* software and read counts were made from .bam files and the average depth was computed from all bases in the window. The relation graph of base pair percentages, and each given sequencing depth along the genome, was obtained.

Using genomic DNA from the MED/Q colony, a total of 20 whole genome sequence (WGS) shotgun sequencing libraries were generated (18 pooled male and female PE and MP libraries, and two haploid-male derived WGA PE libraries), from which sequences were generated on an Illumina Hiseq2500 platform. Library sequencing produced a total of 428.2 Gb or an approximate 594.7-fold genome coverage assuming a 0.72 Gbp genome size (based on 17-mer analysis). For the 10 short-insert PE libraries, there were a total of 229.4 gigabases (Gb) (100 bp or 150 bp read length, approximately 318.6-fold genome coverage). Sequencing the eight large-insert (>1 kb) MP libraries produced 80.3 Gb of reads (49 bp read length, 111.5-fold coverage) for use in scaffold construction (S1 Table). The two male WGA libraries produced a total of 118.5 gigabases (Gb) of data (S1 Table) or approximately 164.6-fold genome coverage Sequencing of 13 BAC pools generated 362.6 Gbp of raw data (288.4 Gbp processed data; results not shown). The subsequent assembly of this sequence data using our pipeline (S1 Fig) generated a 658 Mbp draft genome assembly for MED/Q consistent with recent flow cytometry estimates [17]. The mean read depth across 10 kb windows indicated that all genome regions were highly represented within the read data, with < 1.5% having a depth of < 10X (remaining data not shown).

Through statistics comparison of genome assembly and annotation between MED/Q and MEAM1/B (Table 1), we found the draft genome of MED/Q consisted of a genome size of 658Mb with contig N50 size 44Kb, while MEAM1/B assembly of 615Mb with contig N50 of 30Kb. They have similar G+C content of about 39%, while existed higher TEs in MEAM1/B (44%) than MED/Q (40%). After combining several annotation methods, 20,748

genes were predicted in MED/Q whereas 15,664 genes in MEAM1/B, and about 80% of both two gene sets were supported by several public functional databases.

Annotation of repetitive elements

Repetitive elements were searched for and identified using *Repbase* [18] implemented in *TRF* software [19], and a *de novo* approach implemented in *Piler* [20]. For the *Repbase*-based method, two software programs named *RepeatMasker* [21] and *RepeatProteinMask* were used to identify repetitive sequences. In the *de novo* approach, *Piler-DF-1.0* [20], *RepeatScout-1.0.5* [22], and *LTR-FINDER-1.0.5* [23] were used to build *de novo* repeat libraries from the genome sequences. Finally, the repeated sequences were searched for and classified using the *RepeatMasker* software. Homology-based annotation of MED/Q repetitive elements was queried against Repbase v.20.05 [18] with RepeatMasker [21]. We found a total of 265.0 Mb TEs, or 40.3% of the MED/Q genome size. This was about 10% higher than the repeat contents of *Acyrthosiphon pisum* and *Rhodnius prolixus*, but similar with than that of *Nilaparvata lugens* (39.8%) (S2 Table). This suggests that long terminal repeat (LTR) (18.5%) are more abundant and contain more nucleotides than all other TE classes. This proliferation of LTR retrotransposons has only been found in one other Hemipteran genome, that of *N. lugens* (12.29%). The MED/Q genome also contains the high proportion of the DNA-transposon TEs (12.92%) found in other fully-described Hemipteran genomes. As with both *N. lugens* (0.5%) and *R. prolixus* (0.01%), the MED/Q genome also appears devoid of short interspersed nuclear elements (SINEs; 0.96%). These other Hemipteran genomes also contain a small amount of long interspersed nuclear elements (LINEs; *A. pisum*: 2.6%; MED/Q: 3.18%; *R. prolixus*: 3.2%), but *N. lugens* (12.84%). This suggests that MED/Q-specific TEs, especially the LTRs, have evolved relatively recently and contribute to the large number of gene sets.

Annotation of coding regions

Initial evaluation of gene coverage rate in the draft MED/Q genome assembly was assessed by comparing against 248 core eukaryotic genes were obtained using *CEGMA 2.4* [24] and Benchmarking Universal Single-Copy Orthologs (BUSCO) [25]. Additionally, 105,067 *B. tabaci* transcript sequences, expressed sequence tags (ESTs), of > 200 bpwere used as BLASTn queries against the assembled genome in order to estimate the representation (cutoff *E*-value $\geq 10^{-40}$). Protein-coding gene *de novo* predictions using GENEWISE [26] and *ab initio* gene predictions using GENSCAN [27] and AUGUSTUS [28] were made in combination with 13.7 Gbp of transcriptome (RNA-Seq) data including published MED/Q *B.tabaci* body, guts, and salivary glands [29-31] and additional, previously unpublished data from females and males [32], to obtain consensus gene sets using GLEAN [33].

For homolog-based prediction, protein sequences from nine species (*A. pisum*, *A. mellifera*, *D. melanogaster*, *R. prolixus*, *Z. nevadensis*, *A. gambiae*, *B. mori*, *P. humanus* and *T. castaneum*) were aligned with the MED/Q genome scaffolds using *TblastN* (E-value<1e-5). Target sequences were used to search for accurate gene structures implementing the *GeneWise* software [26]. For the RNA-Seq datasets, the transcriptome reads were first aligned against the genome using *TopHat* [33] to identify candidate exon regions. Then, the *Cufflinks* software [34] was used to assemble the aligned reads into transcripts, and the open reading frames (ORFs) were predicted to obtain reliable transcripts using a Hidden Markov Model (HMM)-based training parameter. Finally, *GLEAN* [33] was used to integrate the predicted genes with the *de novo*, homologous, and RNAseq data to produce the final gene set. The functional annotation of genes was performed using *BLASTP* alignment to KEGG [35], SwissProt and TrEMBL [36] databases. Motifs and domains were determined by *InterProScan* [37] and protein database searches against ProDom, PRINTS, Pfam, SMART, PANTHER and PROSITE.

Preliminary evaluation of transcribed regions within the draft MED/Q genome assembly coverage found that ~95.2% of *B. tabaci* ESTs > 200 bp were present, with 90,652 ESTs showing ≥ 90% length coverage on one scaffold (S7 Table). This alignment encompassed 92.9% of nucleotides within the EST dataset. Analogously, 229 (96%) of the 248 sequences in the CEGMA gene set and 79% complete and fragmented BUSCOs were present in the MED/Q genome assembly (remaining data not shown). The final GLEAN gene models predicted a reference gene set of 20,786 protein-coding genes, a consensus result derived from *de novo*, orthology, and evidence (RNA-seq)-based prediction methods (S3 Table) and integrated into GLEAN gene models (S4 Table). Among the GLEAN gene models, 16,622 (79.97%) received functional gene annotations using the various databases queried in our analysis pipeline (S5 Table).

Prediction of gene orthology

Twelve insect species including *Bemisia tabaci* (Genn.) (Gennadius, 1889) (Hemiptera: Aleyrodidae), *Acyrthosiphon pisum* (Harris, 1776) (Hemiptera: Aphididae), *Rhodnius prolixus* (Stal, 1859) (Hemiptera: Triatominae), *Nilaparvata lugens* (Stål, 1854) (Hemiptera: Delphacidae), *Pediculus humanus* (Linnaeus, 1758) (Phthiraptera: Pediculidae), *Apis mellifera* (Linnaeus, 1758) (Hymenoptera, Apidae), *Nasonia vitripennis* (Ashmead, 1904) (Hymenoptera, Pteromalidae), *Tribolium castaneum* (Herbst, 1797) (Coleoptera, Tenebrionidae), *Anopheles gambiae* (Giles, 1902) (Diptera, Culicidae), *Drosophila melanogaster* (Meigen, 1830) (Diptera, Drosophilidae), *Bombyx mori* (Linnaeus, 1758) (Lepidoptera, Bombycidae) and *Danaus plexippus* (Kluk, 1802) (Lepidoptera, Nymphalidae) and two divergent arthropods, *Daphnia pulex* (Müller, 1785) (O. Cladocera, Daphniidae) and *Tetranychus urticae* (C. L. Koch, 1836) (O. Arachnida, Tetranychidae), were used to predict orthologs and to reconstruct the phylogenetic tree. Gene families were identified using *TreeFam* [38,39], and single-copy gene families were assembled to reconstruct phylogenetic relationships. i) Coding sequences of each single-copy family were concatenated to form one super gene group for each species. ii) All of the nucleotides at codon position 2 of these concatenated genes were extracted to construct the phylogenetic tree by *PhyML* [40], with a gamma distribution across sites and an HKY85 substitution model. iii) The same set of sequences at codon position 2 was used to estimate divergence times among lineages. iv) The fossil calibrations were set with two previous node data [41,42]. v) The *PAML* mcmctree program (v.4.5) [43,44] was used to compute split times using the approximate likelihood calculation algorithm. The software *Tracer* (v.1.5.0) (http://beast.bio.ed.ac.uk/software/tracer/) was utilized to examine the extent of convergence for two independent runs.

Phylogenetic analysis based on orthologs across 14 arthropod taxa (S6 Table) suggested that MED/Q is clustered into a hemipteran clade containing *A. pisum*, and is a sister lineage to a clade containing both *R. prolixus* and *N. lugens* (Fig 1A). The range of species-specific genes within the four hemipteran genomes ranged from 38-60%, with higher values for the three phloem-feeding specialists. This led us to investigate interspecific changes in the number and diversity of gene family members (orthologs and paralogs) within this group of Hemiptera (Fig 1C; Fig S2).

In summary, we report the first genome sequencing, assembly, and annotation of the MEQ/Q *B.tabaci*. This genome assembly will provide a valuable resource for studying climatic and host plant adaptations, invasive-invasive and native-exotic interactions, insecticide resistance, vector competence, and its relationships with bacterial endosymbionts.

Figures

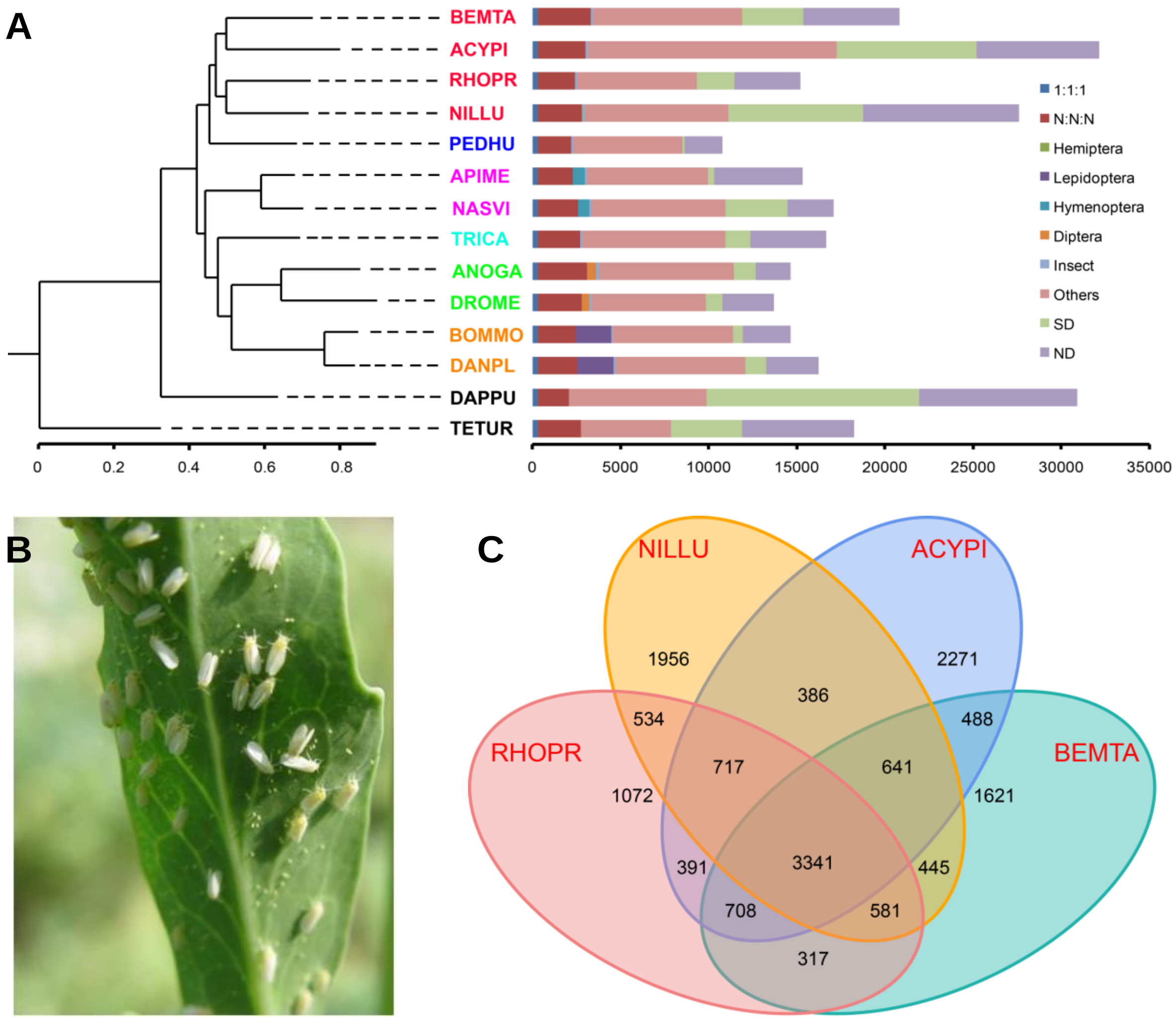

Figure 1

Tables

Table 1. Statistics comparison of genome assembly and annotation between MED/Q and MEAM1/B

Sequencing Summary	MED/Q[a]		MEAM1/B[b]	
	Scaffold[c]	Contig[c]	Scaffold[c]	Contig[c]
Total number	4,954	29,618	19,761	52,036
Total length of (bp)	658,272,463	638,061,971	615,029,878	599,923,598
Gap number(bp)	19,828,575	0	14,380,491	0
Average length (bp)	132,877	21,543	31,123	11,529
N50 length (bp)	436,791	44,366	3,232,964	29,918
N90 length (bp)	111,835	11,504	381,346	6,117
Maximum length (bp)	2,857,362	362,835	11,178,615	269,706
Minimum length (bp)	501	500	500	500
GC content (%)	39.46	39.46	39.64	39.64
TEs proportion (%)	265Mb (0.40)		269Mb (0.44)	
CEGMA evaluation	96%		100%	
BUSCO evaluation	78		96.8	
Gene Number	20,786		15,664	
Average gene length (bp)	10,065		22,762	
Average CDS length (bp)	1,952		1,470	
Average exon per gene	6		6	
Average exon length (bp)	351		234	
Average intron length (bp)	1,776		3,125	
Annotation gene (%)	79.97		81	
Assemble software	SOAPdenovo		Platanus	

[a]From this study.

[b]From the published MEAM1/B genome [11].

[c]Only contigs and scaffolds>=500 bp were included in the genome assembly.

Figure Legend

Figure 1. Phylogenetic relationships and genomic comparisons between *Bemisia tabaci* and other insect species (**A**) Phylogenetic relationships of *B. tabaci* (BEMTA) to insects and other arthropods based on single-copy orthologous genes present in their complete genomes. The following twelve insect species were used for this analysis: *Acyrthosiphon pisum* (ACYPI), *Anopheles gambiae* (ANOGA), *Apis mellifera* (APIME), BEMTA, *Bombyx mori* (BOMMO), *Danaus plexippus* (DANPL), *Drosophila melanogaster* (DROME), *Nasonia vitripennis* (NASVI), *Nilaparvata lugens* (NILLU), *Pediculus humanus* (PEDHU), *Rhodnius prolixus* (RHOPR) and *Tribolium castaneum* (TRICA). The two arthropods *Daphnia pulex* (DAPPU) and *Tetranychus urticae* (TETUR) were used as outgroup taxa. Branch lengths represent divergence times estimated for the second codon position of 308 single-copy genes, using *PhyML* with a gamma distribution across sites and a HKY85 substitution model. The branch supports were inferred based on the approximate likelihood ratio test (aLRT). Gene orthology was determined by comparing the genomes of these 14 arthropod species. The use of 1:1:1 refers to single-copy gene orthologs found across all 14 lineages. The use of N:N:N refers to multi-copy gene paralogs found across the 14 lineages. Diptera, Hemiptera, Hymenoptera, Lepidoptera, and Insecta refer to taxon-specific genes present only in the particular lineage. SD indicates species-specific duplicated genes, and ND indicates species-specific unclustered genes. (**B**) Image of adult MED/Q. (**C**) A Venn diagram showing the orthologous groups shared among the hemipteran genomes of *A. pisum*, *B. tabaci*, *N. lugens* and *R. prolixus*. Our analysis found 3,341 gene families common to all four hemipteran genomes, and 2,921 common to the genomes of the six vascular (blood and phloem) feeders.

Availability of supporting data

This whole genome shotgun project has been deposited at DDBJ/EMBL/GenBank under the accession LIED00000000. The version described in this paper is version LIED01000000 accessible at NCBI. Further data, including annotation files and assembled transcripts, are available in the *GigaScience* GigaDB repository [32].

Abbreviations

BAC: Bacterial artificial chromosome; BUSCO: Benchmarking Universal Single-Copy Orthologs; CEGMA: Core Eukaryotic Genes Mapping Approach; EST: Express sequence tag; HMW: high molecular weight; MED/Q: Mediterranean *Bemisia tabaci* Q; mtCOI: mitochondria cytochrome oxidase I; TEs: transposable elements; WGA: whole-genome amplified; WGS: whole genome shotgun.

Acknowledgments and Funding

The authors would like to thank Dr. Paul De Barro for his comments on an earlier draft. This research was supported by the National Natural Science Foundation of China (31420103919 and 31672032), the Science and Technology Innovation Program of the Chinese Academy of Agricultural Sciences (CAAS-ASTIP-IVFCAAS) and the Beijing Key Laboratory for Pest Control and Sustainable Cultivation of Vegetables. The funders had no role in study design, data collection and analysis, decision to publish, or preparation of the manuscript.

Authors' contributions

YJZ is the leader of the project and the first corresponding author. WX, YJZ, XGZ, YY, JKB and YL were involved in the project design. XGZ, BYX, JYZ, QG, XCL, XQT, MG, HPP, SXR and BLQ coordinated the related research works of the MED/Q genome project. DW performed genome assembly. DW performed protein-coding gene annotation. MC and CHC performed gene orthology and phylogenomics. XY performed insecticide targets annotation. YTL performed putative sex determination genes annotation. WX performed putative phloem specialization genes identification. LTG, LXT, YNW, YZ, QJW, SLW and HYC performed metabolic detoxification systems annotation. ZZY performed immune signaling pathway components annotation. ZZY, JQX, and JQH performed nutrient partitioning between invasive MED/Q and its primary endosymbiont. LTG performed PCR validation. WX, XGZ, DC, JKB, HD, MNM, FG, XPZ, XWW, FHW, YZD, CL, FMY, ELP and XGJ were involved in writing and editing. All authors read and approved the final manuscript.

Competing interests

The authors declare no competing interests defined by *Giga Science*.

References

1. Brown JK, Frohlich DR, Rosell RC. The sweetpotato or silverleaf whiteflies: biotypes of *Bemisia tabaci* or a species complex? Ann Rev Entomol. 1995; 40:511-534. doi: 10.1146/annurev.en.40.010195.002455.
2. De Barro PJ, Liu SS, Boykin LM, Dinsdale AB. *Bemisia tabaci*: astatement of species status. Ann Rev Entomol. 2011; 56:1-19. doi: 10.1146/annurev-ento-112408-085504.
3. Liu SS, Colvin J, De Barro P. Species concepts as applied to the whitefly *Bemisia tabaci* systematics: how many species are there? J Inter Agric. 2012; 11:176-186. doi: 10.1016/S2095-3119(12)60002-1.
4. Wang HL, Yang J, Boykin LM, Zhao QY, Wang YJ, Liu SS, et al. Developing conversed microsatellite markers and their implications in evolutionary analysis of the *Bemisia tabaci* complex. Sci Rep. 2014; 4:6351. doi: 10.1038/srep06351.
5. Tay WT, Evans GA, Boykin LM, De Barro PJ. Will the real *Bemisia tabaci* please stand up? PLoS One. 2012; 7:e50550. doi: 10.1371/journal.pone.0050550.
6. Boykin LM, Armstrong KF, Kubatko L, De Barro P. Species delimitation and global biosecurity. Evol Bioinform Online. 2012; 8:1-37. doi: 10.4137/EBO.S8532.
7. Boykin LM. *Bemisia tabaci* nomenclature: lessons learned. Pest Manag Sci. 2014; 70:1454-1459. doi: 10.1002/ps.3709.
8. Zhang LP, Zhang YJ, Zhang WJ, Wu QJ, Xu BY, Chu D. Analysis of genetic diversity among different geographical populations and determination of biotypes of *Bemisia tabaci* in China. J Appl Entomol. 2005; 129:121–128. doi: 10.1111/j.1439-0418.2005.00950.x.
9. Pan HP, Preisser EL, Chu D, Wang SL, Wu QJ, Carriere Y, et al. Insecticides promote viral outbreaks by altering herbivore competition. Ecol Appl. 2015; 25:1585-1595. PMID: 26552266.
10. Liu BM, Yan FM, Chu D, Pan HP, Jiao XG, Xie W, et al. Multiple forms of vector manipulation by a plant-infecting virus: *Bemisia tabaci* and tomato yellow leaf curl virus. J Virol. 2013; 87:4929-37. doi:10.1128/JVI.03571-12.
11. Chen W, Hasegawa DK, Kaur N, Kliot A, Pinheiro PV, Luan JB, et al. The draft genome of whitefly *Bemisia tabaci* MEAM1, a global crop pest, provides novel insights into virus transmission, host adaptation, and insecticide resistance. BMC Biology. 2016; 14:110. doi 10.1186/s12915-016-0321-y.
12. Chu D, Hu X, Gao C, Zhao H, Nichols RL, Li X. Use of mitochondrial cytochrome oxidase I polymerase chain reaction-restriction fragment length polymorphism for identifying subclades of *Bemisia tabaci* Mediterranean group. J Econ Entomol. 2012; 105:242-251. doi: http://dx.doi.org/10.1603/EC11039.
13. Frohlich DR, Torres-Jerez I I, Bedford ID, Markham PG, Brown JK. A phylogeographical analysis of the *Bemisia tabaci* species complex based on mitochondrial DNA markers. Mol Ecol. 1999; 8:1683-1691. doi: 10.1046/j.1365-294x.1999.00754.x.
14. Li R, Fan W, Tian G, Zhu H, He L, Cai J, et al. The sequence and de novo assembly of the giant panda genome. Nature. 2010; 463:311-317. doi:10.1038/nature08696.
15. You M, Yue Z, He W, Yang X, Yang G, Xie M, et al. A heterozygous moth genome provides insights into herbivory and detoxification. Nat Genet. 2013; 45:220-225. doi:10.1038/ng.2524.
16. Boetzer M, Henkel CV, Jansen HJ, Butler D, Pirovano W. Scaffolding pre-assembled contigs using SSPACE. Bioinformatics. 2011; 27:578-579. doi:10.1093/bioinformatics/btq683.
17. Guo LT, Wang SL, Wu QJ, Zhou XG, Xie W, Zhang YJ. Flow cytometry and K-mer

analysis estimates of the genome sizes of *Bemisia tabaci* B and Q (Hemiptera: Aleyrodidae). Front Physiol. 2015; 6:144. doi: 10.3389/fphys.2015.00144.
18. Jurka J, Kapitonov VV, Pavlicek A, Klonowski P, Kohany O, Walichiewicz J. Repbase Update, a database of eukaryotic repetitive elements. Cytogenet Genome Res. 2005; 110:462-467. doi:10.1159/000084979.
19. Benson G. Tandem repeats finder: a program to analyze DNA sequences. Nucleic Acids Res. 1999; 27:573-580. doi: 10.1093/nar/27.2.573.
20. Edgar RC, Myers EW. PILER: identification and classification of genomic repeats. Bioinformatics. 2005; 21:152-158. doi:10.1093/bioinformatics/bti1003.
21. Smit AFA, Hubley R, Green P. RepeatMasker. 1999; http://www.repeatmasker.org.
22. Price AL, Jones NC, Pevzner PA. De novo identification of repeat families in large genomes. Bioinformatics. 2005; 21:351-358. doi:10.1093/bioinformatics/bti1018.
23. Xu Z, Wang H. LTR_FINDER: an efficient tool for the prediction of full-length LTR retrotransposons. Nucleic Acids Res. 2007; 35:265-268. doi: 10.1093/nar/gkm286.
24. Parra G, Bradnam K, Ning Z, Keane T, Korf I. Assessing the gene space in draft genomes. Nucleic Acids Res. 2009; 37:289-297. doi: 10.1093/nar/gkn916.
25. Simão FA, Waterhouse RM, Ioannidis P, Kriventseva EV and Zdobnov EM. BUSCO: assessing genome assembly and annotation completeness with single-copy orthologs. Bioinformatics, 2015; btv351. doi:10.1093/bioinformatics/btv351.
26. Birney E, Clamp M, Durbin R. GeneWise and Genomewise. Genome Res. 2004; 14:988-995. doi:10.1101/gr.1865504.
27. Burge C, Karlin S. Prediction of complete gene structures in human genomic DNA. J Mol Biol. 1997; 268:78-94. doi:10.1006/jmbi.1997.0951.
28. Stanke M, Keller O, Gunduz I, Hayes A, Waack S, Morgenstern B. AUGUSTUS: ab initio prediction of alternative transcripts. Nucleic Acids Res. 2006; 34: W435-W439. PMID: 16845043.
29. Wang XW, Luan JB, Li JM, Bao YY, Zhang CX, Liu SS. De novo characterization of a whitefly transcriptome and analysis of its gene expression during development. BMC genomics. 2010; 11:400. doi: 10.1186/1471-2164-11-400.
30. Ye XD, Su YL, Zhao QY, Xia WQ, Liu SS Wang XW. Transcriptomic analyses reveal the adaptive features and biological differences of guts from two invasive whitefly species. BMC genomics. 2014; 15:370. doi: 10.1186/1471-2164-15-370.
31. Su YL, Li JM, Li M, Luan JB, Ye XD, Wang XW, et al. Transcriptomic analysis of the salivary glands of an invasive whitefly. PLoS One. 2012; 7:e39303. doi:10.1371/journal.pone.0039303.
32. Xie W, Chen C, Yang Z, Guo L, Yang X, Wang D, Chen M, Huang J, Wen Y, Zeng Y, Liu Y, Xia J, Tian, Cui H, Wu Q, Wang S, Xu B, Li X, Tan X, Ghanim M, Qiu B, Pan H, Chu D, Delatte H, Maruthi MN, Ge F, Zhou X, Wang X, Wan F, Du Y, Luo C, Yan F, Preisser, EL, Jiao X, Coates BS, Zhao J, Gao Q, Xia J, YinY, Liu Y, Brown JK, Zhou XJ, Zhang, Y (2017). Supporting data for "Genome sequencing of the sweetpotato whitefly Bemisia tabaci MED/Q". *GigaScience Database*. http://dx.doi.org/10.5524/100286
33. Elsik CG, Mackey AJ, Reese JT, Milshina NV, Roos DS, Weinstock GM. Creating a honeybee consensus gene set. Genome Biol. 2007; 8:R13. doi: 10.1186/gb-2007-8-1-r13.
34. Trapnell C, Williams BA, Pertea G, Mortazavi A, Kwan G, van Baren MJ, et al. Transcript assembly and quantification by RNA-Seq reveals unannotated transcripts and isoform switching during cell differentiation. Nat Biotechnol. 2010; 28:511-515. doi: 10.1038/nbt.1621.
35. Kanehisa M, Goto S. KEGG: kyoto encyclopedia of genes and genomes. Nucleic Acids Res. 2000; 28:27-30. doi: 10.1093/nar/28.1.27.
36. Bairoch A, Apweiler R. The SWISS-PROT protein sequence database and its supplement

TrEMBL in 2000. Nucleic Acids Res. 2000; 28:45-48. doi: 10.1093/nar/28.1.45.

37. Zdobnov EM, Apweiler R. InterProScan--an integration platform for the signature-recognition methods in InterPro. Bioinformatics. 2001; 17:847-848. doi:10.1093/bioinformatics/17.9.847.

38. Li H, Coghlan A, Ruan J, Coin LJ, Hériché JK, Osmotherly L, et al. TreeFam: a curated database of phylogenetic trees of animal gene families. Nucleic Acids Res. 2006; 34: 572-580. doi: 10.1093/nar/gkj118.

39. Ruan J, Li H, Chen Z, Coghlan A, Coin LJ, Guo Y, et al. TreeFam: 2008 update. Nucleic Acids Res. 2008; 36:735-740. doi: 10.1093/nar/gkm1005.

40. Guindon S, Dufayard JF, Lefort V, Anisimova M, Hordijk W, Gascuel O. New algorithms and methods to estimate maximum-likelihood phylogenies: assessing the performance of PhyML 3.0. Syst Biol. 2010; 59:307-321. doi: 10.1093/sysbio/syq010.

41. Benton MJ, Donoghue PC. Paleontological evidence to date the tree of life. Mol Biol Evol. 2007; 24:26-53. doi: 10.1093/molbev/msl150.

42. Donoghue PCJ, Benton MJ. Rocks and clocks: calibrating the Tree of Life using fossils and molecules. Trends Ecol Evol. 2007; 22:424-431. doi: 10.1016/j.tree.2007.05.005.

43. Yang Z. PAML: a program package for phylogenetic analyses by maximum likelihood. Comp Appl BioSci. 1997; 13:555-556. doi: 10.1099/0022-1317-79-8-1951.

44. Yang Z. PAML 4: phylogenetic analysis by maximum likelihood. Mol Biol Evol. 2007; 24:1586-1591. doi: 10.1093/molbev/msm088.

Additional files

Supporting Figures

Figure S1. Schematic illustration of the assembly pipeline for MED/Q genome based on the combined assemblies from WGS and BACs.

Supporting Tables

Table S1. Statistics of the whole genome sequencing data

Table S2. Repeat Masker analysis in 4 hemiptera species

Table S3. Evidenced use within GLEAN MED/Q protein-coding genes

Table S4. Summary of GLEAN gene models

Table S5. Functional annotation of the MED/Q genome

Table S6. Orthologous gene comparison among genomes of 14 arthropod species

Table S7. Quality control of assembled genome

Table 1. Statistics comparison of genome assembly and annotation between MED/Q and MEAM1/B

Sequencing Summary	MED/Q[a]		MEAM1/B[b]	
	Scaffold[c]	Contig[c]	Scaffold[c]	Contig[c]
Total number	4,954	29,618	19,761	52,036
Total length of (bp)	658,272,463	638,061,971	615,029,878	599,923,598
Gap number(bp)	19,828,575	0	14,380,491	0
Average length (bp)	132,877	21,543	31,123	11,529
N50 length (bp)	436,791	44,366	3,232,964	29,918
N90 length (bp)	111,835	11,504	381,346	6,117
Maximum length (bp)	2,857,362	362,835	11,178,615	269,706
Minimum length (bp)	501	500	500	500
GC content (%)	39.46	39.46	39.64	39.64
TEs proportion (%)	265Mb (0.40)		269Mb (0.44)	
CEGMA evaluation	96%		100%	
BUSCO evaluation	78		96.8	
Gene Number	20,786		15,664	
Average gene length (bp)	10,065		22,762	
Average CDS length (bp)	1,952		1,470	
Average exon per gene	6		6	
Average exon length (bp)	351		234	
Average intron length (bp)	1,776		3,125	
Annotation gene (%)	79.97		81	
Assemble software	SOAPdenovo		Platanus	

[a]From this study.

[b]From the published MEAM1/B genome [11].

[c]Only contigs and scaffolds>=500 bp were included in the genome assembly.

Figure 1

Click here to download Figure Fig1.tif

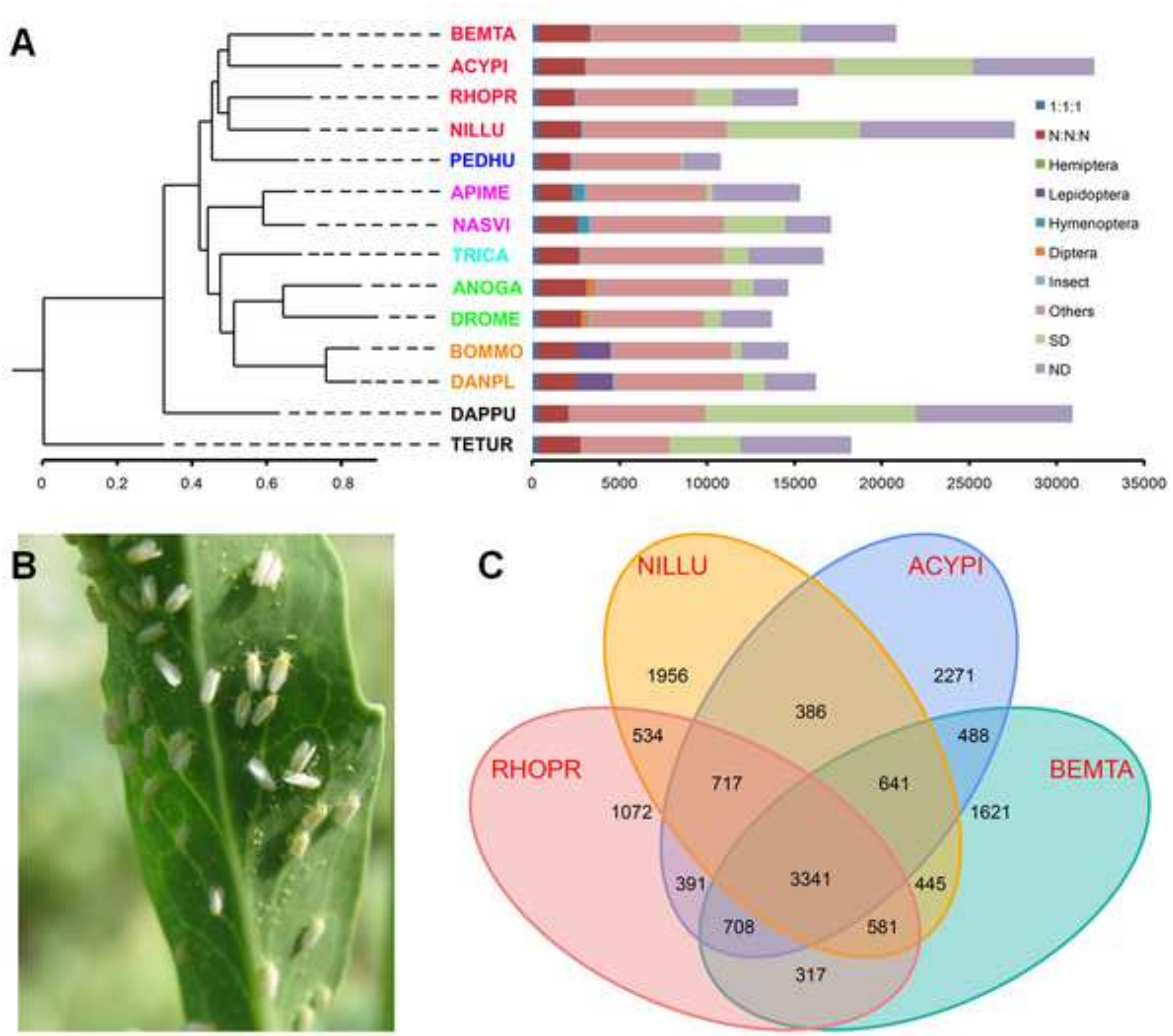

Figure S1

Click here to access/download
Supplementary Material
S1_Fig.tif

Table S1

Click here to access/download
Supplementary Material
S1_Table.docx

Table S2

Click here to access/download
Supplementary Material
S2_Table.docx

Table S3

Click here to access/download
Supplementary Material
S3_Table.docx

Table S4

Click here to access/download
Supplementary Material
S4_Table.docx

Table S5

Click here to access/download
Supplementary Material
S5_Table.docx

Table S6

Click here to access/download
Supplementary Material
S6_Table.docx

Table S7

Click here to access/download
Supplementary Material
S7_Table.doc

UNIVERSITY OF KENTUCKY

College of Agriculture
Department of Entomology
Office of the State Entomologist
S-225 Agriculture Science Center-N
Lexington, KY 40546-0091

RE: MS No. GIGA-D-16-00061

February 4 2017

Hans Zauner
Assistant Editor
GigaScience

Dear Dr. Zauner:

Below, please find our response to your most recent comments and suggestions. Again, I highly appreciate the constructive criticisms and efforts of the reviewer and editor. I sent you a ***point-to-point response*** (file name: Response to Reviewers_Xie et al_12-31-2016_JZ) at the end of last year; however, I have not gotten any reply. Based on your suggestion, we are now converting our manuscript "The invasive Q-type *Bemisia tabaci* genome: a tale of gene loss and gene gain" (GIGA-D-16-00061R1) into the Data Notes format and renamed it to "Genome sequencing of the sweetpotato whitefly *Bemsia tabaci* MED/Q".

EDITOR'S COMMENTS

Thank you for submitting your revised manuscript "The invasive Q-type *Bemisia tabaci* genome: a tale of gene loss and gene gain" (GIGA-D-16-00061R1).

We have discussed your revised manuscript with the previous reviewers and they have alerted us to the recently published B. tabaci genome in BMC Biology:

https://bmcbiol.biomedcentral.com/articles/10.1186/s12915-016-0321-y

Please see the advice from one of the reviewers below.

As some of the authors are co-authors of both, the BMC Biology paper and the submission to Gigascience, I do understand the reviewer's surprise why two separate sequencing projects have been undertaken, rather than one collaborative effort.

After discussing your submission with both previous referees, we agree that it does not present sufficiently novel research to merit publication as a "Research Article", especially in light of the BMC Biology paper.

RESPONSE: We are fully aware of the sequencing efforts of *Bemisia tabaci* B genome. We are excited to see the publication of the B genome, and have cited this work in our previous revision (Ref 32. Chen W, Hasegawa DK, Kaur N, Kliot A, Pinheiro PV, Luan JB, et al. The draft genome of whitefly *Bemisia tabaci* MEAM1, a global crop pest, provides novel insights into virus transmission, host adaptation, and insecticide resistance. In Press. 2016. BMC Biology). Please see our responses to reviewer's comments.

However, we still can consider the submission as a "Data Note". To resubmit as a Data Note,

UNIVERSITY OF KENTUCKY
College of Agriculture
Department of Entomology
Office of the State Entomologist
S-225 Agriculture Science Center-N
Lexington, KY 40546-0091

please revise the manuscript to fit the criteria for this article type (for details, see https://gigascience.biomedcentral.com/submission-guidelines/preparing-your-manuscript/data-note). In short, Data notes highlight and help to contextualise exceptional datasets to encourage reuse. Data Notes provide detailed methodology on data production, validation, and potential reuse.

Please also cite the BMC Biology paper in your data note, and discuss and compare these two data sets of the same species.

RESPONSE: We certainly would not oppose to the suggestion of a data note. As we mentioned in our last response, we already cited BMC Biology paper in our previous revision. We now have compared the sequencing results between the B and Q genomes.

Please also add the BUSCO results mentioned in your response to the reviewer in addition to CEGMA - for our genomic data notes, BUSCO is a standard analysis we now ask authors to include.
RESPONSE: We have incorporated both analyses in the revised manuscript.

Once you have made the necessary changes, please submit online at:

http://giga.edmgr.com/

If you have forgotten your username or password please use the "Send Login Details" link to get your login information. For security reasons, your password will be reset.

I look forward to receiving your revised manuscript.

Best wishes,

Hans Zauner
GigaScience
www.gigasciencejournal.com

RESPONSE: We have converted our manuscript into the Data Notes format.

UK
UNIVERSITY OF KENTUCKY
College of Agriculture
Department of Entomology
Office of the State Entomologist
S-225 Agriculture Science Center-N
Lexington, KY 40546-0091

REVIEWER'S COMMENTS

Reviewer: 2
Dear authors and editors,
With the recent publication of a *Bemisia tabacci* genome paper (BMC Biology, 2016 14:110) by a concurrent group, we have now two *Bemisia* genomes, released by two different consortia. More complicated, some authors are signing both papers! This situation is not well appropriate for the community. To which reference genome will the community refer to? It is really a pity that the two consortia did not work together to share means and ideas instead of duplicating sequencing and analyses.
I am thus forced to consider the submitted paper as a Data Note, since it does not afford strong novelty compare to the other genome. That's the stupid and unfortunate result of competition, instead of collaboration.
And at least, the two consortia should now communicate in order to define a reference genome, and to help the community to find their way. I do consider i5k as a central point for proposing access to insect genomes and I clearly invite the authors to contact them once/if the paper is accepted.

RESPONSE: We certainly respect reviewer's opinions regarding the two independent *Bemisia* genome-sequencing efforts. The following are additional information for reviewer and editor to consider:
1. MED/Q and MEAM1/B are the two most invasive cryptic species within the *Bemisia tabaci* species complex. Although they share similar ecology niche, B and Q have distinctively different biological traits, including host range, virus transmission, endosymbiont composition and insecticide resistance (De Barro et al. 2011; Liu et al. 2012; Pan et al. 2015). Therefore, having these two genomes can greatly benefit the whitefly research community instead of confusing it. Some specific biological distinctions are as follows:

MEAM1/B and MED/Q vary in their mating behavior and prefer different host plants (Crowder et al. 2010, 2011, Elbaz et al. 2011, Tsueda and Tsuchida 2011).

Although both can vectoring viruses, the feeding behavior of MED/Q renders it more competent vector than MEAM1/B for acquiring and transmitting TYLCV and other viruses (Jiang et al. 2000; Pan et al. 2012).

Differential susceptibility to insecticides drives the *B. tabaci* colony displacement in China (Pan et al., 2015). MEAM1/B invaded China in the early 1990s, it rapidly replaced indigenous *B. tabaci* and became the most dominant species throughout China (Luo et al., 2002). MED/Q was first found in Yunnan Province, China, in 2003 (Chu et al., 2006), and has now displaced the well-established MEAM1/B populations in most parts of China (Chu et al., 2010; Teng et al., 2010; Pan et al., 2011; Pan et al., 2015). The key to this drastic displacement event has been attributed to the higher tolerance of MED/Q than MEAM1/B to nearly all commonly used insecticides (Horowitz et al., 2005; Dennehy et al., 2010; Xie et al., 2014; Pan et al., 2015).

Finally, MED/Q originated from the Mediterranean region, and it was widely distributed in many Mediterranean countries, Asia, Canada and America. Phylogenetic analyses of mitochondrial cytochrome oxidase I (mtCOI) sequences suggest that the MED group can be divided into three subclades, known as Q1, Q2 and Q3 (Tsagkarakou

UNIVERSITY OF KENTUCKY

College of Agriculture
Department of Entomology
Office of the State Entomologist
S-225 Agriculture Science Center-N
Lexington, KY 40546-0091

et al. 2007; Ahmed et al. 2009; McKenzie et al. 2012). In China, Q1 is the only haplotype identified so far, and the most dominant *B. tabaci* species (Zheng et al. 2016). In contrast, all three distinct mitochondrial haplotypes of *B. tabaci* Q (Q1, Q2 and Q3) were detected in North America (McKenzie et al. 2012).

2. We are fully aware of the sequencing efforts of *Bemisia tabaci* B genome. We are excited to see the final publication of the B genome, and have cited this work in our previous revision (Ref 32. Chen W, Hasegawa DK, Kaur N, Kliot A, Pinheiro PV, Luan JB, et al. The draft genome of whitefly *Bemisia tabaci* MEAM1, a global crop pest, provides novel insights into virus transmission, host adaptation, and insecticide resistance. In Press. 2016. BMC Biology).

Drs. Youjun Zhang and Zhangjun Fei, the principle investigators for *Bemisia tabaci* Q and B genome sequencing consortia, respectively, have been communicating throughout the sequencing and submission processes. As a result, we have shared some of the expertise from our co-authors. Dr. Fei paid a visit to Dr. Zhang's research group in 2015, and they agreed to coordinate the submission process. Therefore, the initial submission date of *B. tabaci* Q and B genome manuscripts were almost the same. During the review and revision processes, however, the two manuscripts go the separate ways. For example, we spent almost three months to carry out additional experiments to empirically examine the hypothesis derived from the *B. tabaci* Q genome. In contrast, BMC Biology did not ask for the biological validation for the *B. tabaci* B genome, which translates into an earlier publication. Nevertheless, there is clearly no animosities between the two genome consortia. Within the *Bemisia tabaci* community, we share each other's expertise. In fact, Dr. Wenbo Chen, the first author of the *B. tabaci* B genome paper, had previously worked in Dr. Youjun Zhang's research group as a visiting scientist for six month.

Concerning the re-submission, I thank the authors for having evaluated my suggestions. The addition of RNAi on some genes potentially involved in insecticide resistance is a clear added-value.
RESPONSE: We appreciated reviewer giving us the opportunity to improve this work, and we believe the revised version is a clearly better manuscript.

CEGMA versus BUSCO: I still think BUSCO is better than CEGMA but it is not a critical issue. The authors can stand with CEGMA, even if I suggest giving both analyses (CEGMA and BUSCO) to help the readers to estimate the chance to get a full sequence of genes.
RESPONSE: Following reviewer's suggestion, we now incorporated both analyses in the revised manuscript.

So my rejection concerns a publication in GigaScience as a Research Report, but is an acception for a Data Note. Sorry for being so severe, but I feel really bad in front of these situations.
RESPONSE: We hope the new information regarding the biological distinctions between B and Q, in conjunction with the fact that the two *Bemisia* genome consortia have been

UNIVERSITY OF KENTUCKY

College of Agriculture
Department of Entomology
Office of the State Entomologist
S-225 Agriculture Science Center-N
Lexington, KY 40546-0091

communicated throughout the sequencing and submission processes and shared the expertise between the two groups can let reviewer to reconsider his decision.

Thank you again for your consideration and evaluation of this manuscript. We appreciate the opportunity to revise this manuscript for re-consideration, and we also thank the reviewers and editor for their careful review and constructive comments on the first manuscript draft.

With respect,

Xuguo "Joe" Zhou
Associate Professor, Ph.D.
Insect Integrative Genomics
Department of Entomology
University of Kentucky
E-Mail: xuguozhou@uky.edu

Phone: 859-257-3125
Fax: 859-323-1120

Reference

1. De Barro PJ, Liu SS, Boykin LM, Dinsdale AB. *Bemisia tabaci*: a statement of species status. Ann Rev Entomol. 2011; 56:1-19.
2. Liu SS, Colvin J, De Barro PJ. Species concepts as applied to the whitefly *Bemisia tabaci* systematics: how many species are there? J Integr Agric. 2012; 11:176-186.
3. Crowder DW, Sitvarin MI, Carrie` re Y. Plasticity in mating behaviour drives asymmetric reproductive interference in whiteflies. Anim Behav. 2010b; 79:579–587.
4. Elbaz M, Weiser M, Morin S. Asymmetry in thermal tolerance trade-offs between the B and Q sibling species of *Bemisia tabaci* (Hemiptera: Aleyrodidae). J Evolution Biol. 2011; 24:1099–1109.
5. Tsueda H, Tsuchida K. Reproductive differences between Q and B whiteflies, *Bemisia tabaci*, on three host plants and negative interactions in mixed cohorts. Entomol Exp Appl. 2011; 141:197–207.
6. Jiang YC, de Blas C, Barrios L, Fereres A. Correlation between whitefly (Homoptera: Aleyrodidae) feeding behavior and transmission of tomato yellow leaf curl virus. Ann Entomol Soc Am. 2000; 93:573–579.
7. Pan HP, Chu D, Yan WQ, Su Q, Liu BM, Wang SL. et al. Rapid spread of tomato yellow leaf curl virus in China is aided differentially by two invasive whiteflies. PLoS ONE. 2012; 7:e34817.
8. Luo C, Yao Y, Wang RJ, Yan FM, Hu DX, Zhang ZL. The use of mitochondrial cytochrome

UNIVERSITY OF KENTUCKY

College of Agriculture
Department of Entomology
Office of the State Entomologist
S-225 Agriculture Science Center-N
Lexington, KY 40546-0091

oxidase I (mtCO I) gene sequences for the identification of biotype of *Bemisia tabaci* (Gennadius) in China. Acta Entomol Sin. 2002; 45:759-763.
9. Chu D, Zhang YJ, Brown JK, Cong B, Xu BY, Wu QJ. et al. The introduction of the exotic Q biotype of *Bemisia tabaci* from the Mediterranean region into China on ornamental crops. Fla Entomol. 2006; 89:168-174.
10. Chu D, Wan FH, Zhang YJ, Brown JK. Change in the biotype composition of *Bemisia tabaci* in shandong province of China from 2005 to 2008. Environ Entomol. 2010; 39:1028-1036.
11. Teng X, Wan FH, Chu D. *Bemisia tabaci* biotype Q dominates other biotypes across China. Fla Entomol. 2010; 93:363-368.
12. Pan HP, Chu D, Ge D, Wang SL, Wu QJ, Xie W. et al. Further Spread of and domination by *Bemisia tabaci* (Hemiptera: Aleyrodidae) biotype Q on field crops in China. J Econ Entomol. 2011; 104:978-985.
13. Pan HP, Preisser EL, Chu D, Wang SL, Wu QJ, Carriere Y. et al. Insecticides promote viral outbreaks by altering herbivore competition. Ecol Appl. 2015; 25:1585-1595.
14. Horowitz AR, Kontsedalov S, Khasdan V, Ishaaya I. Biotypes band Q of *Bemisia tabaci* and their relevance to neonicotinoid and pyriproxyfen resistance. Arch Insect Biochem. 2005; 58:216-225.
15. Dennehy TJ, Degain BA, Harpold VS, Zaborac M, Morin S, Fabrick JA. et al. Extraordinary resistance to insecticides reveals exotic Q biotype of *Bemisia tabaci* in the New World. J Econ Entomol. 2010; 103:2174-2186.
16. Xie W, Liu Y, Wang SL, Wu QJ, Pan HP, Yang X. et al. Sensitivity of *Bemisia tabaci* (Hemiptera: Aleyrodidae) to several new insecticides in China: effects of insecticide type and whitefly species, strain, and stage. J Insect Sci. 2014; 14:261.
17. Tsagkarakou A, Tsigenopoulous CS, Gorman K, Lagnel J, Bedford ID. Biotype status and genetic polymorphism of the whitefly *Bemisia tabaci* (Hemiptera: Aleyrodidae) in Greece: mitochondrial DNA and microsatellites. Bull Entomol Res. 2007; 97:29-40.
18. Ahmed MZ, Shatters RG, Ren SX, Jin GH, Mandour NS, Qiu BL. Genetic distinctions among the Mediterranean and Chinese populations of *Bemisia tabaci* Q biotype and their endosymbiont Wolbachia populations. J Appl Entomol. 2009; 133:733-741.
19. Zheng HX, Xie W, Wang SL, Wu QJ, Zhou XM, Zhang YJ. Dynamic monitoring (B versus Q) and further resistance status of Q-type *Bemisia tabaci* in China. Crop Prot. 2016. Doi: 10.1016/j.cropro.2016.11.035.
20. McKenzie CL, Bethke JA, Byrne FJ, et al. Distribution of Bemisia tabaci (Hemiptera: Aleyrodidae) biotypes in North America after the Q invasion. J Econ Entom. 2012; 105: 753-766.
