# Supplementary figures and images for: Genome sequencing of the sweetpotato whitefly Bemisia tabaci MED/Q
Source: Gigascience. 2017 Mar 15;6(5):1–7. doi: 10.1093/gigascience/gix018 (PMC5467035; doi:10.1093/gigascience/gix018)

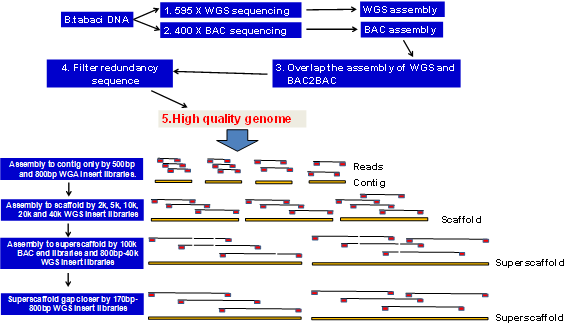

Supplement: Figure S1. — Schematic illustration of the assembly pipeline for MED/Q genome based on the combined assemblies from WGS and BACs. [file gix018_S1_Fig.png]
