# Supplementary material for: Genome sequencing of the sweetpotato whitefly Bemisia tabaci MED/Q
Source: Gigascience. 2017 Mar 15;6(5):1–7. doi: 10.1093/gigascience/gix018 (PMC5467035; doi:10.1093/gigascience/gix018)
Supplement: Table S1. — Statistics of the whole genome sequencing data. [file gix018_S1_Table.docx]

**Table S1. Statistics of whole genome sequencing data**

| **Pair-end Libraies** | **Read length (bp)** | **Insert size (bp)** | **Data (Gb)** | **Sequence depth (X)** | **Physical Depth** |
| --- | --- | --- | --- | --- | --- |
| WHAIPI003160-17 (WGA) | 100 | 500 | 78.3 | 117.6 | 293.9 |
| WHAMPI005110-15 (WGA) | 100 | 800 | 40.2 | 60.4 | 241.4 |
| Total WGA |  |  | 118.5 | 177.9 | 535.4 |
| SZABPI016318-44 | 100 | 170 | 26.2 | 39.3 | 33.4 |
| SZABPI016319-43 | 100 | 170 | 24.1 | 36.2 | 30.8 |
| SZAXPI024841-20 | 150 | 250 | 16.0 | 24.0 | 20.0 |
| SZAXPI024840-21 | 150 | 250 | 24.6 | 36.9 | 30.8 |
| SZAXPI016317-45 | 100 | 300 | 21.8 | 32.7 | 49.1 |
| SZAXPI016322-46 | 100 | 300 | 25.8 | 38.7 | 58.1 |
| SZAIPI015534-20 | 100 | 500 | 19.1 | 28.7 | 71.7 |
| SZAIPI015533-21 | 100 | 500 | 24.0 | 36.0 | 90.1 |
| SZAMPI015532-22 | 100 | 800 | 22.1 | 33.2 | 132.7 |
| SZAMPI015531-23 | 100 | 800 | 25.7 | 38.6 | 154.4 |
| Short insert libraries |  |  | 229.4 | 344.5 | 671.1 |
| BEMebzDAEDWAAPEI-31 | 49 | 2000 | 15.9 | 23.9 | 487.2 |
| BEMebzDAEDWABPEI-37 | 49 | 2000 | 16.5 | 24.8 | 505.6 |
| BEMebzDAEDLBAPEI-35 | 49 | 5000 | 11.4 | 17.1 | 873.3 |
| BEMebzDAEDLAAPEI-34 | 49 | 5000 | 15.0 | 22.5 | 1149.1 |
| BEMebzDAEDTBAPEI-44 | 49 | 10000 | 9.2 | 13.7 | 1401.9 |
| BEMebzDAEDTAAPEI-41 | 49 | 10000 | 7.8 | 11.8 | 1199.7 |
| BEMebzDAEDUAAPEI-37 | 49 | 20000 | 2.4 | 3.7 | 744.6 |
| BEMebzDAEDVAAPEI-41 | 49 | 40000 | 2.0 | 3.1 | 1250.2 |
| Large insert libraries |  |  | 80.3 | 120.5 | 7611.7 |
| Total | - | - | 428.2 | 642.9 | 8818.1 |
