# Supplementary material for: Genome sequencing of the sweetpotato whitefly Bemisia tabaci MED/Q
Source: Gigascience. 2017 Mar 15;6(5):1–7. doi: 10.1093/gigascience/gix018 (PMC5467035; doi:10.1093/gigascience/gix018)
Supplement: Table S2. — Repeat Masker analysis in four hemiptera species. [file gix018_S2_Table.docx]

**Table S2.** Repeat Masker analysis in 4 hemiptera species

|  |  | ***Acyrthosiphon pisum*** | | ***Bemisia tabaci*** | | ***Nilaparvata lugens*** | | ***Rhodnius prolixus*** | |
| --- | --- | --- | --- | --- | --- | --- | --- | --- | --- |
|  |  | **Length (bp)** | **Percent (%)** | **Length (bp)** | **Percent (%)** | **Length (bp)** | **Percent (%)** | **Length (bp)** | **Percent (%)** |
| DNA transposon | | 66,456,504 | 14.31 | 85,077,858 | 12.92 | 130,676,362 | 11.45 | 141,501,804 | 20.14 |
|  | hAT-Tip100 | 8,290,507 | 1.79 | 14,241,345 | 2.16 | 1,643,289 | 0.14 | 169,925 | 0.02 |
|  | hAT-hAT5 | 25,907 | 0.01 | 12,686,393 | 1.93 | 25,046 | 0 | 1,349 | 0 |
|  | CMC-EnSpm | 663,535 | 0.14 | 8,733,777 | 1.33 | 20,987,157 | 1.84 | 994,495 | 0.14 |
|  | Zator | 463 | 0 | 8,069,056 | 1.23 | 32,745 | 0 | 35,999 | 0.01 |
|  | TcMar-Mariner | 47,419 | 0.01 | 4,813,514 | 0.73 | 1,601,321 | 0.14 | 125,840,811 | 17.91 |
|  | Maverick | 801,635 | 0.17 | 2,702,328 | 0.41 | 13,189,471 | 1.16 | 1,029,661 | 0.15 |
|  | hAT-Charlie | 1,836,751 | 0.4 | 2,094,311 | 0.32 | 3,391,556 | 0.3 | 2,837,122 | 0.4 |
|  | hAT | 6,167,504 | 1.33 | 840,979 | 0.13 | 1,877,074 | 0.16 | 528,363 | 0.08 |
|  | TcMar-Tc1 | 694,913 | 0.15 | 148,354 | 0.02 | 12,828,284 | 1.12 | 3,390,081 | 0.48 |
|  | DNA | 5,954,658 | 1.28 | 23,599 | 0 | 109,613 | 0.01 | 46,528 | 0.01 |
|  | Novosib | 542,658 | 0.12 | 2,380 | 0 | 32,834,519 | 2.88 | 2,743 | 0 |
| LTR |  | 14,475,552 | 3.12 | 121,814,430 | 18.5 | 140,195,792 | 12.29 | 34,814,221 | 4.95 |
|  | Gypsy | 3,447,085 | 0.74 | 12,384,080 | 1.88 | 34,617,150 | 3.03 | 3,705,736 | 0.53 |
|  | Copia | 347,907 | 0.07 | 8,082,810 | 1.23 | 1,875,164 | 0.16 | 603,113 | 0.09 |
|  | Pao | 1,557,493 | 0.34 | 5,048,369 | 0.77 | 14,539,028 | 1.27 | 747,762 | 0.11 |
|  | ERVK | 90,886 | 0.02 | 844,377 | 0.13 | 2,242,354 | 0.2 | 141,309 | 0.02 |
| LINE | | 12,077,243 | 2.6 | 20,920,803 | 3.18 | 146,443,403 | 12.84 | 22,469,588 | 3.2 |
|  | RTE-BovB | 1,396,522 | 0.3 | 6,861,136 | 1.04 | 30,599,713 | 2.68 | 2,082,445 | 0.3 |
|  | Jockey | 3,692,974 | 0.8 | 3,465,455 | 0.53 | 10,525,547 | 0.92 | 6,376,921 | 0.91 |
|  | L1 | 65,327 | 0.01 | 1,961,798 | 0.3 | 1,156,554 | 0.1 | 272,916 | 0.04 |
|  | R1 | 1,323,220 | 0.28 | 1,554,306 | 0.24 | 1,673,083 | 0.15 | 832,413 | 0.12 |
|  | L2 | 1,933,444 | 0.42 | 1,490,226 | 0.23 | 79,979,576 | 7.01 | 1,883,301 | 0.27 |
|  | CR1 | 1,668,550 | 0.36 | 1,046,353 | 0.16 | 6,655,361 | 0.58 | 678,294 | 0.1 |
|  | LOA | 25,821 | 0.01 | 132,575 | 0.02 | 619,768 | 0.05 | 3,156,365 | 0.45 |
|  | RTE-X | 17,061 | 0 | 70,166 | 0.01 | 2,350,725 | 0.21 | 2,506,889 | 0.36 |
| SINE |  | 31,623,176 | 6.81 | 6,308,561 | 0.96 | 5,755,889 | 0.5 | 57,025 | 0.01 |
|  | 5S | 8,845 | 0 | 1,629,774 | 0.25 | 3,192,847 | 0.28 | 3,881 | 0 |
|  | tRNA-Lys | 971 | 0 | 328,492 | 0.05 | 15,598 | 0 | 719 | 0 |
|  | Deu | 0 | 0 | 222,763 | 0.03 | 374 | 0 | 552 | 0 |
|  | ID | 62,832 | 0.01 | 64,929 | 0.01 | 1,403,679 | 0.12 | 1,900 | 0 |
|  | tRNA-Deu-L2 | 0 | 0 | 4,762 | 0 | 0 | 0 | 0 | 0 |
|  | tRNA-L2 | 0 | 0 | 4,430 | 0 | 0 | 0 | 0 | 0 |
|  | 7SL | 542 | 0 | 1,513 | 0 | 653,501 | 0.06 | 778 | 0 |
|  | SINE | 3,199,835 | 0.69 | 78 | 0 | 847 | 0 | 757 | 0 |
|  | C | 543 | 0 | 0 | 0 | 1,419 | 0 | 1,977 | 0 |
| ARTEFACT | | 0 | 0 | 0 | 0 | 7,719 | 0 | 0 | 0 |
| RC |  | 1,337,285 | 0.29 | 18,012,658 | 2.74 | 12,773,145 | 1.12 | 653,344 | 0.09 |
| Other | | 4,798 | 0 | 663 | 0 | 18,981 | 0 | 3,271 | 0 |
| Unknown | | 24,800,394 | 5.34 | 13,070,828 | 1.99 | 18,021,260 | 1.58 | 6,049,944 | 0.86 |
| **Total TE** |  | 150774952 | 32.47 | 265205801 | 40.29 | 453892551 | 39.78 | 205549197 | 29.25 |
| Simple_repeat | | 451,496 | 0.1 | 10,687,627 | 1.62 | 52,615,747 | 4.61 | 4,483,785 | 0.64 |
| Satellite |  | 54,676 | 0.01 | 3,483,412 | 0.53 | 7,743,059 | 0.68 | 153,586 | 0.02 |
| TRF |  | 3,413,952 | 0.74 | 19,578,623 | 2.97 | 40,148,516 | 3.52 | 9,992,159 | 1.42 |
| **Total** |  | 154,695,076 | 33.32 | 298,955,463 | 45.41 | 554,399,873 | 48.6 | 220,178,727 | 31.34 |

Note: LTR represents long terminal repeat, LINE represents long interspersed nuclear elements, SINE represents short interspersed nuclear elements. Total TE includes DNA transposon, LTR, LINE, SINE, RC, ARTEFACT, Unknown and Other.
