# Supplementary material for: Genome sequencing of the sweetpotato whitefly Bemisia tabaci MED/Q
Source: Gigascience. 2017 Mar 15;6(5):1–7. doi: 10.1093/gigascience/gix018 (PMC5467035; doi:10.1093/gigascience/gix018)
Supplement: Table S3. — Evidenced use within GLEAN MED/Q protein-coding genes. [file gix018_S3_Table.docx]

**Table S3. Evidenced use within GLEAN MED/Q protein-coding genes**

|  | **Gene set** | **Number** | **Average gene length (bp)** | **Average CDS length (bp)** | **Average exon per gene** | **Average exon length (bp)** | **Average intron length (bp)** |
| --- | --- | --- | --- | --- | --- | --- | --- |
| *De novo* | augustus | 28253 | 7400.6 | 1282.5 | 4.3 | 298.3 | 1854.7 |
|  | genscan | 21112 | 11608.6 | 1784.7 | 7.3 | 244.3 | 1558.0 |
| Orthology | *Acyrthosiphon pisum* | 19673 | 5266.1 | 975.7 | 3.4 | 285.4 | 1774.1 |
|  | *Anopheles gambiae* | 12760 | 6528.0 | 915.5 | 4.0 | 228.8 | 1869.8 |
|  | *Apis mellifera* | 13285 | 6491.2 | 917.7 | 4.0 | 228.2 | 1844.4 |
|  | *Pediculus humanus* | 13586 | 6870.9 | 1027.5 | 4.3 | 240.5 | 1785.6 |
|  | *Rhodnius prolixus* | 18886 | 4641.1 | 806.2 | 3.2 | 249.0 | 1713.3 |
|  | *Tribolium castaneum* | 17008 | 5995.5 | 1053.0 | 3.8 | 280.6 | 1795.6 |
|  | *Zootermopsis nevadensis* | 17626 | 5559.5 | 855.4 | 3.6 | 236.5 | 1798.2 |
|  | *Bombyx mori* | 15688 | 4708.9 | 811.8 | 3.3 | 248.4 | 1717.7 |
|  | *Drosophila melanogaster* | 12817 | 6517.1 | 917.6 | 4.0 | 227.6 | 1847.6 |
| RNA-Seq |  | 18951 | 10476.5 | 1584.5 | 5.5 | 287.6 | 1972.0 |
| GLEAN |  | 20786 | 10049.0 | 1504.6 | 5.1 | 293.5 | 1962.7 |
