# Supplementary material for: Genome sequencing of the sweetpotato whitefly Bemisia tabaci MED/Q
Source: Gigascience. 2017 Mar 15;6(5):1–7. doi: 10.1093/gigascience/gix018 (PMC5467035; doi:10.1093/gigascience/gix018)
Supplement: Table S4. — Summary of GLEAN gene models. [file gix018_S4_Table.docx]

**Table S4. Summary of GLEAN gene models**

|  | **≥20% overlap** | | **≥50% overlap** | | **≥80% overlap** | |
| --- | --- | --- | --- | --- | --- | --- |
|  | No. | Ratio (%) | No. | Ratio (%) | No. | Ratio (%) |
| C (single) | 250 | 1.20% | 320 | 1.54% | 583 | 2.80% |
| C (more) | 0 | 0.00% | 0 | 0.00% | 0 | 0.00% |
| H (single) | 138 | 0.66% | 190 | 0.91% | 217 | 1.04% |
| H (more) | 442 | 2.13% | 337 | 1.62% | 158 | 0.76% |
| P (single) | 287 | 1.38% | 887 | 4.27% | 3025 | 14.55% |
| P (more) | 3663 | 17.62% | 4714 | 22.68% | 5377 | 25.87% |
| H+C | 229 | 1.10% | 213 | 1.02% | 167 | 0.80% |
| P+C | 2078 | 10.00% | 3160 | 15.20% | 4317 | 20.77% |
| P+H | 4080 | 19.63% | 3211 | 15.45% | 2049 | 9.86% |
| P+H+C | 8666 | 41.69% | 6714 | 32.30% | 3581 | 17.23% |

The evidence for the gene prediction results based on de novo (P) and homolog-based (H) and EST/RNA-seq date (C) is presented. The "single" and "more" symbols represent the availability of single or multiple pieces of evidence. "Overlap" indicates the overlapping region of CDS between the gene sets of GLEAN and both prediction methods. The table below shows the combined results of both prediction methods.
