# Supplementary material for: Genome sequencing of the sweetpotato whitefly Bemisia tabaci MED/Q
Source: Gigascience. 2017 Mar 15;6(5):1–7. doi: 10.1093/gigascience/gix018 (PMC5467035; doi:10.1093/gigascience/gix018)
Supplement: Table S5. — Functional annotation of the MED/Q genome. [file gix018_S5_Table.docx]

**Table S5. Functional annotation of the MED/Q genome**

| **Total** |  | **Number** | **% of genes** |
| --- | --- | --- | --- |
| Annotated | Swissprot | 13384 | 64.39 |
|  | TrEMBL | 16146 | 77.68 |
|  | KEGG | 10951 | 52.68 |
|  | InterPro | 13622 | 65.53 |
|  | GO | 10910 | 52.49 |
|  | NR | 16299 | 78.41 |
|  | All annotations | 16622 | 79.97 |
| Unknown |  | 4164 | 20.03 |

The proteomic prediction of the MED/Q genome was based on the functional protein databases of SwissPort and TrENBL from the UniProt (http://www.uniprot.org), InterPro (http://www.ebi.ac.uk/interpro/) and its associated GO annotation, and KEGG pathways for metabolism and celluar processes and NCBI non-rudundant datebase. A total set of (20786) protein were investigated, (16622) of which could be well annotated.
