# Supplementary material for: Genome sequencing of the sweetpotato whitefly Bemisia tabaci MED/Q
Source: Gigascience. 2017 Mar 15;6(5):1–7. doi: 10.1093/gigascience/gix018 (PMC5467035; doi:10.1093/gigascience/gix018)
Supplement: Table S6. — Orthologous gene comparison among genomes of 14 arthropod species. [file gix018_S6_Table.docx]

**Table S6. Orthologous gene comparison among genomes of 14 arthropod species**

| **Species** | **BEMTA** | **ACYPI** | **RHOPR** | **NILLU** | **PEDHU** | **APIME** |  | **NASVI** |  | **TRICA** | **ANOGA** | **DROME** | **BOMMO** | **DANPL** | **DAPPU** | **TETUR** |
| --- | --- | --- | --- | --- | --- | --- | --- | --- | --- | --- | --- | --- | --- | --- | --- | --- |
| 1:1:1 | 308 | 308 | 308 | 308 | 308 | 308 |  | 308 |  | 308 | 308 | 308 | 308 | 308 | 308 | 308 |
| N:N:N | 2987 | 2681 | 2097 | 2499 | 1893 | 1960 |  | 2250 |  | 2389 | 2786 | 2469 | 2125 | 2209 | 1759 | 2431 |
| Hemiptera | 23 | 13 | 14 | 17 | 0 | 0 |  | 0 |  | 0 | 0 | 0 | 0 | 0 | 0 | 0 |
| Lepidoptera | 0 | 0 | 0 | 0 | 0 | 0 |  | 0 |  | 0 | 0 | 0 | 2015 | 2052 | 0 | 0 |
| Hymenoptera | 0 | 0 | 0 | 0 | 0 | 701 |  | 678 |  | 0 | 0 | 0 | 0 | 0 | 0 | 0 |
| Diptera | 0 | 0 | 0 | 0 | 0 | 0 |  | 0 |  | 0 | 509 | 427 | 0 | 0 | 0 | 0 |
| Insect | 125 | 132 | 125 | 130 | 106 | 127 |  | 131 |  | 120 | 180 | 141 | 119 | 131 | 0 | 0 |
| Other | 8429 | 14126 | 6769 | 8173 | 6200 | 6837 |  | 7573 |  | 8103 | 7610 | 6456 | 6803 | 7343 | 7794 | 5129 |
| SD | 3481 | 7924 | 2148 | 7624 | 107 | 347 |  | 3515 |  | 1413 | 1279 | 950 | 571 | 1205 | 12069 | 4006 |
| ND | 5433 | 6925 | 3725 | 8820 | 2155 | 5034 |  | 2629 |  | 4298 | 1961 | 2938 | 2675 | 2971 | 8969 | 6342 |
| total | 20786 | 32109 | 15186 | 27571 | 10769 | 15314 |  | 17084 |  | 16631 | 14633 | 13689 | 14616 | 16219 | 30899 | 18216 |
| Species-specific (SD+ND) | 8914 | 14849 | 5873 | 16444 | 2262 | 5381 |  | 6144 |  | 5711 | 3240 | 3888 | 3246 | 4176 | 21038 | 10348 |
| # With orthologs | 11872 | 17260 | 9313 | 11127 | 8507 | 9933 |  | 10940 |  | 10920 | 11393 | 9801 | 11370 | 12043 | 9861 | 7868 |
| Species-specific% | 42.88% | 46.25% | 38.67% | 59.64% | 21.00% | 35.14% |  | 35.96% |  | 34.34% | 22.14% | 28.40% | 22.21% | 25.75% | 68.09% | 56.81% |
| With orthologs% | 57.12% | 53.75% | 61.33% | 40.36% | 79.00% | 64.86% |  | 64.04% |  | 65.66% | 77.86% | 71.60% | 77.79% | 74.25% | 31.91% | 43.19% |

1:1:1 refers to single-copy gene orthologs found across all 14 lineages. N:N:N refers to multi-copy gene paralogs found across all 14 lineages. Hemiptera, Lepidoptera, Hymenoptera, Diptera and Insect refer to taxon-specific genes that are present only in the relevant lineage. SD indicates species-specific duplicated genes. ND indicates species-specific un-clustered genes. Abbreviatoin: MED/Q (BEMTA), Acyrthosiphon pisum (ACYPI), Anopheles gambiae (ANOGA), Apis mellifera (APIME), BEMTA, Bombyx mori (BOMMO), Danaus plexippus (DANPL), Drosophila melanogaster (DROME), Nasonia vitripennis (NASVI), Nilaparvata lugens (NILLU), Pediculus humanus (PEDHU), Rhodnius prolixus (RHOPR) and Tribolium castaneum (TRICA), Daphnia pulex (DAPPU), Tetranychus urticae (TETUR).
