# Supplementary material for: Genome sequencing of the sweetpotato whitefly Bemisia tabaci MED/Q
Source: Gigascience. 2017 Mar 15;6(5):1–7. doi: 10.1093/gigascience/gix018 (PMC5467035; doi:10.1093/gigascience/gix018)
Supplement: Table S7. — Quality control of assembled genome. [file gix018_S7_Table.doc]

**Table S7. Quality control of assembled genome.** Assembly quality validation was calculated by expressed sequence tags coverage against transcriptome assembled unigene sequences

| **Dataset** | **Number** | **Total length (bp)** | **Bases covered by assembly (%)** | **Sequences covered by assembly (%)** | **>90% sequence in one scaffold** | | **>50% sequence in one scaffold** | |
| --- | --- | --- | --- | --- | --- | --- | --- | --- |
|  |  |  |  |  | **Number** | **Percent** | **Number** | **Percent** |
| >0bp | 196627 | 64443253 | 92.22602248 | 93.67228305 | 165144 | 83.98847 | 180712 | 91.90599 |
| >200bp | 105067 | 51703292 | 92.88031795 | 95.21543396 | 90652 | 86.28018 | 97783 | 93.06728 |
| >500bp | 28988 | 28823107 | 93.58265575 | 96.89871671 | 25108 | 86.61515 | 27256 | 94.02511 |
| >1000bp | 9394 | 15425829 | 93.84311858 | 97.93485203 | 7957 | 84.703 | 8883 | 94.56036 |
