# Supplementary material for: Genome sequencing of the sweetpotato whitefly Bemisia tabaci MED/Q
Source: Gigascience. 2017 Mar 15;6(5):1–7. doi: 10.1093/gigascience/gix018 (PMC5467035; doi:10.1093/gigascience/gix018)
Supplement: Response_to_reviewer_comments_Original_Submission.pdf [file gix018_Response_to_reviewer_comments_Original_Submission.pdf]

RE: MS No. GIGA-D-16-00061

December 8 2016

Hans Zauner
Assistant Editor
GigaScience

Dear Hans:

Below, please find our descriptions of revisions to manuscript GIGA-D-16-00061 entitled “The invasive Q-type Bemisia tabaci genome: a tale of gene loss and gene gain”. We have followed the vast majority of reviewer suggestions, and have no absolute disagreements/rebuttals to any reviewer comments. As a result, we now submit what is hopefully a significantly improved manuscript that provides a more convincing depiction of our results and conclusions. We are hopeful that the work will now meet your standards for publication as a "Research Article".

EDITOR'S COMMENTS

Your manuscript "The invasive Q-type Bemisia tabaci genome: a tale of gene loss and gene gain" (GIGA-D-16-00061) has been assessed by two reviewers. Although it is of interest, we are unable to consider it for publication in its current form. The reviewers have raised a number of points which we believe would improve the manuscript and may allow a revised version to be published in GigaScience.

Reviewer 2, Denis Tagu, feels the genome data are of interest, but more biological experiments would be necessary to test hypotheses. Along the same lines, referee 1, Laura Boykin, feels the manuscript lacks defined scientific questions.

If you are confident that you can provide sufficient additional biological data to convince the referees, we would be able to consider a revised manuscript as "Research Article".

However, you may prefer to revise the submission as a "Data Note" - see http://gigascience.biomedcentral.com/submission-guidelines/preparing-your-manuscript/data-note

If you decide to revise your manuscript as a "Data Note", you should concentrate on providing a useful dataset, transparently described in detail for future users and the research community.

In any case, please also include BUSCO analysis in a revised manuscript, as suggested by Denis Tagu.

I notice that Laura Boykin refers to the nomenclature issues surrounding the B. tabaci species complex. I do not think that this controversial topic should be at the forefront of a Gigascience article, which is about the genome sequence. Nevertheless, I feel that it is appropriate to cite and briefly outline the relevant recent literature dealing with B. tabaci nomenclature. Our readers should get a brief, but complete and unbiased overview of the published arguments that have been put forward in this debate.

The reports are below. Please also take a moment to check our website at http://giga.edmgr.com/ for any additional comments that were saved as attachments.

If you are able to fully address these points, we would encourage you to submit a revised manuscript to GigaScience. Please note that we will seek further advice from referees before making a decision on your revised submission.

Once you have made the necessary corrections, please submit online at:

http://giga.edmgr.com/

If you have forgotten your username or password please use the "Send Login Details" link to get your login information. For security reasons, your password will be reset.

Please include a point-by-point within the 'Response to Reviewers' box in the submission system. Please ensure you describe additional experiments that were carried out and include a detailed rebuttal of any criticisms or requested revisions that you disagreed with. Please also ensure that your revised manuscript conforms to the journal style, which can be found in the Instructions for Authors on the journal homepage.

The due date for submitting the revised version of your article is 08 Dec 2016.

I look forward to receiving your revised manuscript soon.

Best wishes,

Hans Zauner
GigaScience
www.gigasciencejournal.com

RESPONSE: Reviewer and editor's constructive criticisms and suggestions are well received. Based on Drs Denis Tagu and Laura Boykin's suggestions, additional empirical experiments have been carried out to examine the hypotheses derived from this genome sequencing effort and to address a specific biological question. Generally, most of the suggested revisions involving minor changes have been incorporated into the revised manuscript. The following is a point-to-point response to reviewers' comments:

REVIEWERS' COMMENTS

Reviewer: 1
The article needs to be greatly improved but very important data. After reading the article I'm left asking myself- why did the team sequence the genome?

The title is using incorrect nomenclature for this species complex. All references to biotype are obsolete. Please see the literature below and revise.

Key literature surrounding the nomenclature of the species complex are missing. References 9 and 10 are outdated. I recommend a complete literature search of the topic but read
1. Wang, H.L., J. Yang, L. M. Boykin, Q.Y. Zhao, Y.J. Wang, S.S. Liu, X.W. Wang. 2014. Development, characterization and analysis of microsatellite markers from the transcriptomes of three whitefly Bemisia tabaci species. Scientific Reports. doi:10.1038/srep06351.
2. Tay, W.T., G. A. Evans, L.M. Boykin, P.J. De Barro. 2012. Will the real Bemisia tabaci please stand up? PLoS ONE 7(11): e50550. doi:10.1371/journal.pone.0050550.
3. Boykin, L.M., K.F. Armstrong, L. Kubatko, and P. De Barro. 2012. Species Delimitation and Global Biosecurity. Evolutionary Bioinformatics 8: 1-37.
4. Boykin, L.M. 2014. Bemisia tabaci nomenclature: Lessons learned. Pest Management Science 70:1454-59.
Avoid the "sibling" species terminology. Remove all reference to biotype throughout the manuscript. What is "strain selection"? Later in the paper MED/Q is used. Be consistent with the naming.
RESPONSE: We echo Dr. Laura Boykin's sentiments about the nomenclature of the Bemisia tabaci species complex. According to her suggestion, we eliminated "biotype" throughout the manuscript and replaced "sibling" with "cryptic" species. "Strain selection" has been removed to avoid confusion.

As for the references, we included all four publications recommended by the reviewer. The debate over Bemisia tabaci as a complex species or species complex has been over a half century. To acknowledge this

part of the history, and also to show “MED/Q was inadvertently introduced into several geographic locations worldwide, and became established throughout China”, we would like to not exclude the references referring B. tabaci as biotypes. References 9 and 10 (listed here) represent some of the major discoveries from our research group, and were published recently in well-respected peer-reviewed journals. The nomenclature in these publications is debatable; however, the contents are relevant and up-to-date.
9. Pan HP, Preisser EL, Chu D, Wang SL, Wu QJ, Carriere Y, et al. Insecticides promote viral outbreaks by altering herbivore competition. Ecol Appl. 2015; 25:1585-1595. PMID: 26552266.
10. Liu BM, Yan FM, Chu D, Pan HP, Jiao XG, Xie W, et al. Multiple forms of vector manipulation by a plant-infecting virus: Bemisia tabaci and tomato yellow leaf curl virus. J Virol. 2013; 87:4929-37. doi:10.1128/JVI.03571-12.

The introduction does not properly review the literature or set up the read for the study that has been conducted or why it is important to have a genome for this particular B. tabaci species.

The discussion need to be rewritten completely. There are no scientific questions that were set out to be answered with this genome paper. I recommend reading:
http://bfg.oxfordjournals.org/content/early/2016/06/22/bfgp.elw026.long and paying attention to the reference: http://www.sciencedirect.com/science/article/pii/S1471492214000762

The days of "sequence-first-ask-questions-later" are over and this paper needs to be greatly improved with well defined research questions relevant to Bemisia tabaci species before it can be published anywhere.
RESPONSE: Based on reviewer’s suggestion, we carried out additional experiments to address specific biological questions. We totally agree that genome sequencing should have clear biological purposes. With the additional RNAi-based functional data, we elected “insecticide resistance” as the focal point to reorganize and rewrite the discussion in the revised manuscript.

Reviewer: 2
I have reviewed this Bemisia genome paper with interest: this is a long time that the community is expecting the release of the genome of this Hemipteran pest, and I am satisfied to see that a consortium tackled the difficulty. This is a regular genome paper whose aim I guess is to provide basic data of an annotated genome and a few analyses. There is thus an interest to publish it, if the community has access to a well-structured genome database of B. tabacci, so that the community will still improve annotation and provide new knowledge with other analyses. My first recommendation is thus to provide this access, more than from NBCI. I suggest the authors to contact the i5k community who developed a dedicated database for insects, with a nice interface allowing search, blast and web Apollo annotation (I am not member of this i5k database!).
RESPONSE: To share the genome information using i5K database platform is a great idea, we will certainly contact i5K community after the conclusion of this publication. In the meantime, we have already uploaded the genome sequence onto NCBI and GigaDB. NCBI maintains genome sequences of plants, animals and microbes, and can be readily accessed through user-friendly interfaces, including blast, Map Viewer, and CD Tree. GigaDB contains 268 discoverable, tractable and citable databases that are available for public download and use. Therefore, we are comfortable using these two databases to share B. tabacci genomic resources.

As I said before, the general analyses are global, and centered on specific gene families such as detoxification (in relation to insecticide resistance and host plant interactions) and immune system (in relation to endosymbiont relationship). There are thus many other gene families that would deserve analyses but I understand that this might not be essential for the paper. But as the paper focuses on a small number of family genes, I would expect more biological experiments that would allow testing some of the hypotheses suggested by the authors. For instance, authors could provide some RNA expression data of candidate genes (e.g. P450) on
different host plants or insecticides, or from different Bemisia populations with others insecticide resistance profiles. Or some experiments on the IMD pathways such as the one provided for the A. pisum paper. I don't say the authors should provide all these analyses, but at least put more biological data.

RESPONSE: Based on Dr Denis Tagu's suggestion, we selected one of the main interests from our group, insecticide resistance, as the focal point for the discussion section. A total of 12 genes encoding detoxification enzymes, including 9 P450s and 3 GSTs, were subjected to RNAi-based functional validation studies to investigate their potential involvement in the imidacloprid resistance.

The hypothesis of HGT is also interesting, but it is known that final demonstration is complicated. So to lower the fact that this is an HGT. It could be, but this remains to be demonstrated. please revise a bit the text
RESPONSE: Based on reviewer's suggestion, we toned down the HGT hypothesis.

Another trait of Bemisia is the transmission of plant viruses, as the authors several times mention it in the text. I would expect some gene family analysis of proteins that possibly play roles in virus transport (vesicle processes?).
RESPONSE: Most recently, a group at the Cornell University published Bemisia B genome (Chen et al., 2016) with a focus on the genomic signatures contributing to virus transmission. We cited this work in the revised manuscript.

Chen et al. 2016. The draft genome of whitefly Bemisia tabaci MEAM1, a global crop pest, provides novel insights into virus transmission, host adaptation, and insecticide resistance. BMC Biology.

The text needs strong English editing. Some parts are OK, but others are different to follow. I suggest the English-native co-authors carefully check all the manuscript, including figure and table legends.
RESPONSE: Revisions have been made according to reviewer's suggestion.

Other minor points:
Does the strain that have been sequenced disseminate plant viruses?
RESPONSE: Yes, B. tabaci Q is notorious for its ability to transmit plant viruses.

Males are haploid. For Hymenoptera genome projects, males are usually used for sequencing in order to get rid off heterozygocity. I am not a specialist of whitefly biology, but why did not you use only male individuals for this genome project?
RESPONSE: We are aware of the strategies to minimizing the heterozygocity. However, for B. tabaci, it is difficult to distinguish the adult sex with the naked eyes, its size is too small to generate enough genetic materials individually for the genome sequencing. We, therefore, used unsexed, mass collection of B. tabaci adults for the sequencing.

The authors used CEGMA for quality control of sequencing and assembly. I would suggest using BUSCO which proposed a larger set of conserved proteins for Insects or Arthropods. The authors will thus have a better assessment of their genome I guess.
RESPONSE: Based on reviewer's suggestion, we used BUSCO to evaluate the quality of B. tabaci genome, gene set, and transcriptome. There are 2,675 conserved arthropod proteins in total. As shown in the following table, we have detected 79% complete and fragmented BUSCOs in B. tabaci genome, 88% in gene set, and 70% in transcriptome.

Pattern	Genome		Gene set		Transcriptome	
	Number	%	Number	%	Number	%
Complete BUSCOs	1399	52%	2105	78%	1276	47%
Complete and single-copy BUSCOs	1118	42%	1434	54%	985	10%
Complete and duplicated BUSCOs	281	10%	671	25%	291	11%
Fragmented BUSCOs	706	26%	257	9.6%	595	22%
All (Complete and Fragmented)	2105	79%	2362	88%	1871	70%
Missing BUSCOs	570	21%	313	11%	804	30%

Based on this assessment, we would like to stay with the CEGMA analysis in this manuscript.

The authors could check within the non-assembled reads whether some missing genes that are not present in the assembly might be there, or even other bacterial sequences/genomes.

RESPONSE: 1) We mapped all WGS clean data covering different insert libs from 170bp to 40kb into B. tabaci MED/Q-type genome by SOAPaligner/soap2 V2.21t. Then non-assembled reads were filtered to assemble through software SOAPdenovo, and a draft sequence with 385Mb genome size was constructed. 2) In order to check whether any sequence existed in previous B. tabaci MED/Q-type genome, the 385Mb assembled sequence was again aligned to B. tabaci MED/Q-type genome with software blast and filtered 65Mb unmapped-assembled sequences (333,000 sequences). 3) Functional analysis was utilized to analyze the composition of these unmapped sequences through mapping them to NT database by software blast. On one hand, we mapped these unmapped-assembled sequences into the transcript sequences to search EST sequences by software blat. Secondly, a homolog based alignment with Acyrthosiphon pisum was ran by software Blast and Genewise. And then, we merged the gene set though Glean and got nine genes. Finally, we aligned these nine genes into B. tabaci MED/Q-type coding genes and into B. tabaci MED/Q-type genome by blast. Results shown four genes were successfully mapped into the B. tabaci MED/Q coding genes, and five not. That is, we got five unmapped genes, which might be missing gene in the present assemble. Functional analysis was again to search their function in NR database from NCBI by blast, and all these five genes were all annotated as gene indeed belong to B. tabaci (Tables 1 and 3). One the other hand, we found these unmapped-assembled sequences mainly include bacteria sequences from the following species (Table 2).

Table 1. Function analysis with NR database of missing genes

Gene ID	Description	Max score	Total score	Query cover	E value	Identity	Accession
Gene1	PREDICTED: rho GTPase-activating protein 190 isoform X1 [Bemisia tabaci]	179	179	0.81	2E-50	0.97	XP_018903139.1
Gene2	PREDICTED: DNA polymerase epsilon catalytic subunit A [Bemisia tabaci]	272	272	0.94	5E-82	0.98	XP_018915083.1
Gene5	PREDICTED: lysosomal alpha-glucosidase-like [Bemisia tabaci]	259	259	0.99	2E-83	0.99	XP_018911552.1
Gene6	PREDICTED: acetylcholinesterase [Bemisia tabaci]	209	209	1	2E-61	1	XP_018906011.1
Gene7	PREDICTED: MIF4G domain-containing protein isoform X2 [Bemisia tabaci]	106	106	1	3E-26	1	XP_018899706.1

Table 2. The organisms of unmapped-assemble sequences

Organism	Mapped sequence number
Candidatus Hamiltonella	931
Pseudomonas fluorescens	928
Pseudomonas sp.	767
Pseudomonas trivialis	609
Flavobacterium johnsoniae	553
Methylotenera mobilis	236
Cardinium endosymbiont	183
Pseudomonas poae	69

Sphingobacterium sp. 60
Pseudomonas brassicacearum 42
Pseudomonas chlororaphis 34
Pseudomonas fragi 33
Pseudomonas mandelii 25
Pseudomonas putida 25
Chryseobacterium sp. 21

Table 3. Missing sequences
>Gene7 locus=C60404577:235:387:-
ATGGACCAGATGCGCATTCAATTTTTAAAGCAAACCACTTCGCCTAGTTTTCGGAAAACTTTGCTGCAAATGATCGAACT
AAGAGCAAGCAAGTGGGCTCTTCCAGTTGAAAGTATTATTTACTACTATCCTAGCAATAGCCAAAAAAAGTAA
>Gene5 locus=C60410339:723:1079:+
ATGACATCAACTAATTGGGCGCTTGCTGATGAATGTGCCATTGTTCCAAATGAAGACAGATTTGACTGTTTCCCACGCG
GACCTTCCAACGAATCGGTTTGTACTCAACGAGGCTGTTGCTGGAAACCCACTGAGAGCCGCACTGAAATGGATGGATT
TAAGAAACTGGATGTCCCTTGGTGTTACTACCCAGTTGCTTTTAAGTCGTACGAGCAGGTGAATAAAACAGTAGCCAAT
CATGTCACCACTGTCTTTCTGAGAAATGTTATTAAGTCACCCTATCCTGATGATGTGCCTCTTCTCAGAATGGTCATCAAA
GCGGAATCCAACTTCAGGGTTCATGTTAAGGTGAGTTAA
>Gene1 locus=C60412988:80:1241:+
ATGAATAATATAGATGTATTTTTCTACGTGCAATTTTTTCAATTTCTCTCCTTGCTTTTTCAGTCTGACTTCAGCGGAAGA
GTCGTTAATAACGACCATTTCCTGTATTGGGGTGAAGTATCCAAAGCAACAGAGGAAGGAGCGGAAATTCAGTTTCAAG
TCATCGAGCAAACTGAATTCATCGATGACGCATCCTTTCAGCCATTTAAAGGTGGCAAAATGGAGCCTTATATTAAACGA
TGTGCGGCAGTCAAGTTGACATCAGCAGAGAAACTCATGTACATTTGTAAAAATCAATTAGGTAAGTTCAGATTGTTGTA
A
>Gene2 locus=C60415092:393:1815:-
GCTAAAAAACAGGTTGCAGAAGCTCTTGAAAAAAATGATGCTGGAGAGATAAAATCTGCGAAAAATCGTGAAGTTTTGT
ATGACTCTTTACAAATAGCTCACAAATGCATCCTGAACTCTTTTTATGGTTACGTCATGCGGAAAGGCGCCAGATGGCAT
AGTATGGAGATGGCTGGAATAGTGTGTCACACTGGAGCTAATATCATCACAAGAGCTAGAGAAATCATTGAAAAAGTTG
GTCGACCACTTGAATTAGATACAGATGGTATTTGGTGTGTTTTACCTGCATCTTTTCCAGAAAATTATGTGATTAATTCCA
CACATCCGGGCAAAAGTAAAATTACCATCTCTTACCCAAATGCTGTACTGAATTCCATGGTGAAGGTAATTTTTGTTGTG
GTACATATATGTTCTTGCTAA
>Gene6 locus=C60415266:261:957:+
ATGTTGTCAGACTTTCTCTTCAGAGCTCCTGTTGATCACATCGTAAAACTCCTCGTCAGTCAAGACGTTCCTACATATATG
TACGTCATGAATACAACGGTCGAAGCTTTGCGCCTGCCTGAATGGAGGAAATACCCTCACAACATCGAACATTACTTCC
TCACTGGAGCACCCTTCATGGACACAGAATTTTTCCCTCCAAGCGCCCATCTTGAAAGGAATATGTGGACGGACAATGA
TAGGAACATGAGTCACTTTTTCATGAAAGCTTATTCAAACTTTGCAAAATATGGGTAA

Repetitive element analysis is a bit poor. No possibility to describe a bit more the different families of transposons?
RESPONSE: Revisions have been made according to reviewer’s suggestion.

The gene coverage section is short and difficult to follow (page 11 lines 10 and following).
RESPONSE: Revisions have been made according to reviewer’s suggestion (Table S15).

In the text, comparison of insect-symbionts system is very difficult to follow too.
RESPONSE: Revisions have been made according to reviewer’s suggestion (Figure 4).

Conclusion (at least as it is today) is not necessary: too long and redundant with the text.
RESPONSE: Revisions have been made according to reviewer’s suggestion.

Figure 3: any possibility to put all the proteins present in the table within the figure/flow chart?
RESPONSE: Revisions have been made according to reviewer’s suggestion (Figure 3).

Figure 4: I guess that the arrows showing the transfer of metabolites are not demonstrated but suggested

by this work? Please mention it.
RESPONSE: Please see revised figure legend.

Figure 5: please improve the legends that are not clear and incomplete (e.g. what are the green boxes in 5B?).
RESPONSE: Revisions have been made according to reviewer's suggestion.

Figure S4, Table S3, Table S7, Table S9; not sure they are necessary
RESPONSE: Figure S4, Table S3, Table S7 and Table S9 supported the statements in the manuscript. We, therefore, would like to keep these figure and tables in the supplementary materials.

Please also take a moment to check our website at for any additional comments that were saved as attachments. Please note that as GigaScience has a policy of open peer review, you will be able to see the names of the reviewers.
RESPONSE: According to reviewer's suggestion, we carried out additional experiments, reorganized and rewrote the manuscript.

Thank you for your consideration and evaluation of this manuscript. We appreciate the opportunity to revise this manuscript for re-consideration, and we also thank the reviewers and editor for their careful review and constructive comments on the first manuscript draft.

With respect,

Xuguo "Joe" Zhou
Associate Professor, Ph.D.
Insect Integrative Genomics

Department of Entomology
University of Kentucky
E-Mail: xuguozhou@uky.edu

Phone: 859-257-3125
Fax: 859-323-1120
