# Supplementary material for: Genome sequencing of the sweetpotato whitefly Bemisia tabaci MED/Q
Source: Gigascience. 2017 Mar 15;6(5):1–7. doi: 10.1093/gigascience/gix018 (PMC5467035; doi:10.1093/gigascience/gix018)
Supplement: Response_to_reviewer_comments_Revision_1.pdf [file gix018_Response_to_reviewer_comments_Revision_1.pdf]

REVIEWER'S COMMENTS

Reviewer: 2
Dear authors and editors,
With the recent publication of a Bemisia tabacci genome paper (BMC Biology, 2016 14:110) by a concurrent group, we have now two Bemisia genomes, released by two different consortia. More complicated, some authors are signing both papers! This situation is not well appropriate for the community. To which reference genome will the community refer to? It is really a pity that the two consortia did not work together to share means and ideas instead of duplicating sequencing and analyses.
I am thus forced to consider the submitted paper as a Data Note, since it does not afford strong novelty compare to the other genome. That's the stupid and unfortunate result of competition, instead of collaboration.
And at least, the two consortia should now communicate in order to define a reference genome, and to help the community to find their way. I do consider i5k as a central point for proposing access to insect genomes and I clearly invite the authors to contact them once/if the paper is accepted.

RESPONSE: We certainly respect reviewer's opinions regarding the two independent Bemisia genome-sequencing efforts. The following are additional information for reviewer and editor to consider:
1. MED/Q and MEAM1/B are the two most invasive cryptic species within the Bemisia tabaci species complex. Although they share similar ecology niche, B and Q have distinctively different biological traits, including host range, virus transmission, endosymbiont composition and insecticide resistance (De Barro et al. 2011; Liu et al. 2012; Pan et al. 2015). Therefore, having these two genomes can greatly benefit the whitefly research community instead of confusing it. Some specific biological distinctions are as follows:
MEAM1/B and MED/Q vary in their mating behavior and prefer different host plants (Crowder et al. 2010, 2011, Elbaz et al. 2011, Tsueda and Tsuchida 2011).
Although both can vectoring viruses, the feeding behavior of MED/Q renders it more competent vector than MEAM1/B for acquiring and transmitting TYLCV and other viruses (Jiang et al. 2000; Pan et al. 2012).
Differential susceptibility to insecticides drives the B. tabaci colony displacement in China (Pan et al., 2015). MEAM1/B invaded China in the early 1990s, it rapidly replaced indigenous B. tabaci and became the most dominant species throughout China (Luo et al., 2002). MED/Q was first found in Yunnan Province, China, in 2003 (Chu et al., 2006), and has now displaced the well-established MEAM1/B populations in most parts of China (Chu et al., 2010; Teng et al., 2010; Pan et al., 2011; Pan et al., 2015). The key to this drastic displacement event has been attributed to the higher tolerance of MED/Q than MEAM1/B to nearly all commonly used insecticides (Horowitz et al., 2005; Dennehy et al., 2010; Xie et al., 2014; Pan et al., 2015).
Finally, MED/Q originated from the Mediterranean region, and it was widely distributed in many Mediterranean countries, Asia, Canada and America. Phylogenetic analyses of mitochondrial cytochrome oxidase I (mtCOI) sequences suggest that the MED group can be divided into three subclades, known as Q1, Q2 and Q3 (Tsagkarakou et al. 2007; Ahmed et al. 2009; McKenzie et al. 2012). In China, Q1 is the only haplotype identified so far, and the most dominant B. tabaci species (Zheng et al. 2016). In contrast, all three distinct mitochondrial haplotypes of B. tabaci Q (Q1, Q2 and Q3) were detected in North America (McKenzie et al. 2012).

2. We are fully aware of the sequencing efforts of Bemisia tabaci B genome. We are excited to see the final publication of the B genome, and have cited this work in our previous revision (Ref 32. Chen W, Hasegawa DK, Kaur N, Kliot A, Pinheiro PV, Luan JB, et al. The draft genome of whitefly Bemisia tabaci MEAM1, a global crop pest, provides novel insights into virus transmission, host adaptation, and insecticide resistance. In Press. 2016. BMC Biology).
Drs. Youjun Zhang and Zhangjun Fei, the principle investigators for Bemisia tabaci Q and B genome sequencing consortia, respectively, have been communicating throughout the sequencing and submission processes. As a result, we have shared some of the expertise from our co-authors. Dr. Fei paid a visit to Dr. Zhang's research group in 2015, and they agreed to coordinate the submission process. Therefore, the initial submission date of B. tabaci Q and B genome manuscripts were almost the same. During the review and revision processes, however, the two manuscripts go the separate

ways. For example, we spent almost three months to carry out additional experiments to empirically examine the hypothesis derived from the B. tabaci Q genome. In contrast, BMC Biology did not ask for the biological validation for the B. tabaci B genome, which translates into an earlier publication. Nevertheless, there is clearly no animosities between the two genome consortia. Within the Bemisia tabaci community, we share each other's expertise. In fact, Dr. Wenbo Chen, the first author of the B. tabaci B genome paper, had previously worked in Dr. Youjun Zhang's research group as a visiting scientist for six month.

Concerning the re-submission, I thank the authors for having evaluated my suggestions. The addition of RNAi on some genes potentially involved in insecticide resistance is a clear added-value.
RESPONSE: We appreciated reviewer giving us the opportunity to improve this work, and we believe the revised version is a clearly better manuscript.

CEGMA versus BUSCO: I still think BUSCO is better than CEGMA but it is not a critical issue. The authors can stand with CEGMA, even if I suggest giving both analyses (CEGMA and BUSCO) to help the readers to estimate the chance to get a full sequence of genes.
RESPONSE: Following reviewer's suggestion, we now incorporated both analyses in the revised manuscript.

So my rejection concerns a publication in GigaScience as a Research Report, but is an acception for a Data Note. Sorry for being so severe, but I feel really bad in front of these situations.
RESPONSE: We hope the new information regarding the biological distinctions between B and Q, in conjunction with the fact that the two Bemisia genome consortia have been communicated throughout the sequencing and submission processes and shared the expertise between the two groups can let reviewer to reconsider his decision.

Reference

1. De Barro PJ, Liu SS, Boykin LM, Dinsdale AB. Bemisia tabaci: a statement of species status. Ann Rev Entomol. 2011; 56:1-19.
2. Liu SS, Colvin J, De Barro PJ. Species concepts as applied to the whitefly Bemisia tabaci systematics: how many species are there? J Integr Agric. 2012; 11:176-186.
3. Crowder DW, Sitvarin MI, Carrie` re Y. Plasticity in mating behaviour drives asymmetric reproductive interference in whiteflies. Anim Behav. 2010b; 79:579–587.
4. Elbaz M, Weiser M, Morin S. Asymmetry in thermal tolerance trade-offs between the B and Q sibling species of Bemisia tabaci (Hemiptera: Aleyrodidae). J Evolution Biol. 2011; 24:1099–1109.
5. Tsueda H, Tsuchida K. Reproductive differences between Q and B whiteflies, Bemisia tabaci, on three host plants and negative interactions in mixed cohorts. Entomol Exp Appl. 2011; 141:197–207.
6. Jiang YC, de Blas C, Barrios L, Fereres A. Correlation between whitefly (Homoptera: Aleyrodidae) feeding behavior and transmission of tomato yellow leaf curl virus. Ann Entomol Soc Am. 2000; 93:573–579.
7. Pan HP, Chu D, Yan WQ, Su Q, Liu BM, Wang SL. et al. Rapid spread of tomato yellow leaf curl virus in China is aided differentially by two invasive whiteflies. PLoS ONE. 2012; 7:e34817.
8. Luo C, Yao Y, Wang RJ, Yan FM, Hu DX, Zhang ZL. The use of mitochondrial cytochrome oxidase I (mtCO I) gene sequences for the identification of biotype of Bemisia tabaci (Gennadius) in China. Acta Entomol Sin. 2002; 45:759-763.
9. Chu D, Zhang YJ, Brown JK, Cong B, Xu BY, Wu QJ. et al. The introduction of the exotic Q biotype of Bemisia tabaci from the Mediterranean region into China on ornamental crops. Fla Entomol. 2006; 89:168-174.
10. Chu D, Wan FH, Zhang YJ, Brown JK. Change in the biotype composition of Bemisia tabaci in shandong province of China from 2005 to 2008. Environ Entomol. 2010; 39:1028-1036.
11. Teng X, Wan FH, Chu D. Bemisia tabaci biotype Q dominates other biotypes across China. Fla Entomol. 2010; 93:363-368.
12. Pan HP, Chu D, Ge D, Wang SL, Wu QJ, Xie W. et al. Further Spread of and domination by Bemisia tabaci (Hemiptera: Aleyrodidae) biotype Q on field crops in China. J Econ Entomol. 2011; 104:978-985.
13. Pan HP, Preisser EL, Chu D, Wang SL, Wu QJ, Carriere Y. et al. Insecticides promote viral outbreaks by altering herbivore competition. Ecol Appl. 2015; 25:1585-1595.
14. Horowitz AR, Kontsedalov S, Khasdan V, Ishaaya I. Biotypes band Q of Bemisia tabaci and their

relevance to neonicotinoid and pyriproxyfen resistance. Arch Insect Biochem. 2005; 58:216-225.
15. Dennehy TJ, Degain BA, Harpold VS, Zaborac M, Morin S, Fabrick JA. et al. Extraordinary resistance to insecticides reveals exotic Q biotype of Bemisia tabaci in the New World. J Econ Entomol. 2010; 103:2174-2186.
16. Xie W, Liu Y, Wang SL, Wu QJ, Pan HP, Yang X. et al. Sensitivity of Bemisia tabaci (Hemiptera: Aleyrodidae) to several new insecticides in China: effects of insecticide type and whitefly species, strain, and stage. J Insect Sci. 2014; 14:261.
17. Tsagkarakou A, Tsigenopoulous CS, Gorman K, Lagnel J, Bedford ID. Biotype status and genetic polymorphism of the whitefly Bemisia tabaci (Hemiptera: Aleyrodidae) in Greece: mitochondrial DNA and microsatellites. Bull Entomol Res. 2007; 97:29-40.
18. Ahmed MZ, Shatters RG, Ren SX, Jin GH, Mandour NS, Qiu BL. Genetic distinctions among the Mediterranean and Chinese populations of Bemisia tabaci Q biotype and their endosymbiont Wolbachia populations. J Appl Entomol. 2009; 133:733-741.
19. Zheng HX, Xie W, Wang SL, Wu QJ, Zhou XM, Zhang YJ. Dynamic monitoring (B versus Q) and further resistance status of Q-type Bemisia tabaci in China. Crop Prot. 2016. Doi: 10.1016/j.cropro.2016.11.035.
20. McKenzie CL, Bethke JA, Byrne FJ, et al. Distribution of Bemisia tabaci (Hemiptera: Aleyrodidae) biotypes in North America after the Q invasion. J Econ Entom. 2012; 105: 753-766.
