# Supplementary material for: Genome sequencing of the sweetpotato whitefly Bemisia tabaci MED/Q
Source: Gigascience. 2017 Mar 15;6(5):1–7. doi: 10.1093/gigascience/gix018 (PMC5467035; doi:10.1093/gigascience/gix018)
Supplement: Reviewer_1_Report_(Original_Submission).pdf [file gix018_Reviewer_1_Report_(Original_Submission).pdf]

Reviewer Report

Title: "Genome sequencing of the sweetpotato whitefly Bemisia tabaci MED/Q"

Version: Original Submission **Date:** 8/19/2016

Reviewer name: Laura Boykin

Reviewer Comments to Author:

The article needs to be greatly improved but very important data. After reading the article I'm left asking myself- why did the team sequence the genome?

The title is using incorrect nomenclature for this species complex. All references to biotype are obsolete. Please see the literature below and revise.

Key literature surrounding the nomenclature of the species complex are missing. References 9 and 10 are outdated. I recommend a complete literature search of the topic but read Wang, H.L., J. Yang, L. M. Boykin, Q.Y. Zhao, Y.J. Wang, S.S. Liu, X.W. Wang. 2014. Development,

characterization and analysis of microsatellite markers from the transcriptomes of three whitefly Bemisia tabaci species. Scientific Reports. doi:10.1038/srep06351. Tay, W.T., G. A. Evans, L.M. Boykin, P.J. De Barro. 2012. Will the real Bemisia tabaci please stand up? PLoS ONE 7(11): e50550. doi:10.1371/journal.pone.0050550. Boykin, L.M., K.F. Armstrong, L. Kubatko, and P. De Barro. 2012. Species Delimitation and Global Biosecurity. Evolutionary Bioinformatics 8: 1-37. Boykin, L.M. 2014. Bemisia tabaci nomenclature: Lessons learned. Pest Management Science 70:1454-59.

Avoid the "sibling" species terminology. Remove all reference to biotype throughout the manuscript. What is "strain selection"? Later in the paper MED/Q is used. Be consistent with the naming.

The introduction does not properly review the literature or set up the read for the study that has been conducted or why it is important to have a genome for this particular B. tabaci species.

The discussion need to be rewritten completely. There are no scientific questions that were set out to be answered with this genome paper. I recommend reading: http://bfg.oxfordjournals.org/content/early/2016/06/22/bfgp.elw026.long and paying attention to the reference: http://www.sciencedirect.com/science/article/pii/S1471492214000762

The days of "sequence-first-ask-questions-later" are over and this paper needs to be greatly improved with well defined research questions relevant to Bemisia tabaci species before it can be published anywhere.

Methods

Are the methods appropriate to the aims of the study, are they well described, and are necessary controls included? No

Conclusions

Are the conclusions adequately supported by the data shown? No

Reporting Standards

Does the manuscript adhere to the journal's guidelines on minimum standards of reporting? Yes

Statistics

Are you able to assess all statistics in the manuscript, including the appropriateness of statistical tests used? Yes, and I have assessed the statistics in my report.

Quality of Written English

Please indicate the quality of language in the manuscript: Needs some language corrections before being published

Declaration of Competing Interests

Please complete a declaration of competing interests, considering the following questions:

- Have you in the past five years received reimbursements, fees, funding, or salary from an organisation that may in any way gain or lose financially from the publication of this manuscript, either now or in the future?
- Do you hold any stocks or shares in an organisation that may in any way gain or lose financially from the publication of this manuscript, either now or in the future?
- Do you hold or are you currently applying for any patents relating to the content of the manuscript?
- Have you received reimbursements, fees, funding, or salary from an organization that holds or has applied for patents relating to the content of the manuscript?
- Do you have any other financial competing interests?
- Do you have any non-financial competing interests in relation to this paper?

If you can answer no to all of the above, write 'I declare that I have no competing interests' below. If your reply is yes to any, please give details below.

I have no competing interests

I agree to the open peer review policy of the journal. I understand that my name will be included on my report to the authors and, if the manuscript is accepted for publication, my named report including any attachments I upload will be posted on the website along with the authors' responses. I agree for my report to be made available under an Open Access Creative Commons CC-BY license (http://creativecommons.org/licenses/by/4.0/). I understand that any comments which I do not wish to be included in my named report can be included as confidential comments to the editors, which will not be published.

I agree to the open peer review policy of the journal

To further support our reviewers, we have joined with Publons, where you can gain additional credit to further highlight your hard work (see: https://publons.com/journal/530/gigascience). On publication of this paper, your review will be automatically added to Publons, you can then choose whether or not to claim your Publons credit. I understand this statement.

Yes
