# Supplementary material for: Genome sequencing of the sweetpotato whitefly Bemisia tabaci MED/Q
Source: Gigascience. 2017 Mar 15;6(5):1–7. doi: 10.1093/gigascience/gix018 (PMC5467035; doi:10.1093/gigascience/gix018)
Supplement: Reviewer_2_Report_(Original_Submission).pdf [file gix018_Reviewer_2_Report_(Original_Submission).pdf]

Reviewer Report

Title: "Genome sequencing of the sweetpotato whitefly Bemisia tabaci MED/Q"

Version: Original Submission **Date:** 9/4/2016

Reviewer name: Denis Tagu

Reviewer Comments to Author:

I have reviewed this Bemisia genome paper with interest: this is a long time that the community is expecting the release of the genome of this Hemipteran pest, and I am satisfied to see that a consortium tackled the difficulty.

This is a regular genome paper whose aim I guess is to provide basic data of an annotated genome and a few analyses. There is thus an interest to publish it, if the community has access to a well-structured genome database of B. tabacci, so that the community will still improve annotation and provide new knowledge with other analyses. My first recommendation is thus to provide this access, more than from NBCI. I suggest the authors to contact the i5k community who developed a dedicated database for insects, with a nice interface allowing search, blast and web Apollo annotation (I am not member of this i5k database!).

As I said before, the general analyses are global, and centered on specific gene families such as detoxification (in relation to insecticide resistance and host plant interactions) and immune system (in relation to endosymbiont relationship). There are thus many other gene families that would deserve analyses but I understand that this might not be essential for the paper. But as the paper focuses on a small number of family genes, I would expect more biological experiments that would allow testing some of the hypotheses suggested by the authors. For instance, authors could provide some RNA expression data of candidate genes (e.g. P450) on different host plants or insecticides, or from different Bemisia populations with others insecticide resistance profiles. Or some experiments on the IMD pathways such as the one provided for the A. pisum paper. I don't say the authors should provide all these analyses, but at least put more biological data.

The hypothesis of HGT is also interesting, but it is known that final demonstration is complicated. So please revise a bit the text to lower the fact that this is an HGT. It could be, but this remains to be demonstrated.

Another trait of Bemisia is the transmission of plant viruses, as the authors several times mention it in the text. I would expect some gene family analysis of proteins that possibly play roles in virus transport (vesicle processes?).

The text needs strong English editing. Some parts are OK, but others are different to follow. I suggest the English-native co-authors carefully check all the manuscript, including figure and table legends.

Other minor points:

Does the strain that have been sequenced disseminate plant viruses?

Males are haploid. For Hymenoptera genome projects, males are usually used for sequencing in order to get rid off heterozygocity. I am not a specialist of whitefly biology, but why did not you use only male individuals for this genome project?

The authors used CEGMA for quality control of sequencing and assembly. I would suggest using BUSCO which proposed a larger set of conserved proteins for Insects or Arthropods. The authors will thus have a better assessment of their genome I guess.

The authors could check within the non-assembled reads whether some missing genes that are not present in the assembly might be there, or even other bacterial sequences/genomes.

Repetitive element analysis is a bit poor. No possibility to describe a bit more the different families of transposons?

The gene coverage section is short and difficult to follow (page 11 lines 10 and following).

In the text, comparison of insect-symbionts system is very difficult to follow too.

Conclusion (at least as it is today) is not necessary: too long and redundant with the text.

Figure 3: any possibility to put all the proteins present in the table within the figure/flow chart?

Figure 4: I guess that the arrows showing the transfer of metabolites are not demonstrated but suggested by this work? Please mention it.

Figure 5: please improve the legends that are not clear and incomplete (e.g. what are the green boxes in 5B?).

Figure S4, Table S3, Table S7, Table S9; not sure they are necessary

Methods

Are the methods appropriate to the aims of the study, are they well described, and are necessary controls included? Yes

Conclusions

Are the conclusions adequately supported by the data shown? Yes

Reporting Standards

Does the manuscript adhere to the journal's guidelines on minimum standards of reporting? Yes

Statistics

Are you able to assess all statistics in the manuscript, including the appropriateness of statistical tests used? No, and I do not feel adequately qualified to assess the statistics.

Quality of Written English

Please indicate the quality of language in the manuscript: Not suitable for publication unless extensively edited

Declaration of Competing Interests

Please complete a declaration of competing interests, considering the following questions:

- Have you in the past five years received reimbursements, fees, funding, or salary from an organisation that may in any way gain or lose financially from the publication of this manuscript, either now or in the future?
- Do you hold any stocks or shares in an organisation that may in any way gain or lose financially from the publication of this manuscript, either now or in the future?
- Do you hold or are you currently applying for any patents relating to the content of the manuscript?
- Have you received reimbursements, fees, funding, or salary from an organization that holds or has applied for patents relating to the content of the manuscript?
- Do you have any other financial competing interests?
- Do you have any non-financial competing interests in relation to this paper?

If you can answer no to all of the above, write 'I declare that I have no competing interests' below. If your reply is yes to any, please give details below.

I declare that I have no competing interests

I agree to the open peer review policy of the journal. I understand that my name will be included on my report to the authors and, if the manuscript is accepted for publication, my named report including any attachments I upload will be posted on the website along with the authors' responses. I agree for my report to be made available under an Open Access Creative Commons CC-BY license (http://creativecommons.org/licenses/by/4.0/). I understand that any comments which I do not wish to be included in my named report can be included as confidential comments to the editors, which will not be published.

I agree to the open peer review policy of the journal

To further support our reviewers, we have joined with Publons, where you can gain additional credit to further highlight your hard work (see: https://publons.com/journal/530/gigascience). On publication of this paper, your review will be automatically added to Publons, you can then choose whether or not to claim your Publons credit. I understand this statement.

Yes
