# Supplementary material for: Genome sequencing of the sweetpotato whitefly Bemisia tabaci MED/Q
Source: Gigascience. 2017 Mar 15;6(5):1–7. doi: 10.1093/gigascience/gix018 (PMC5467035; doi:10.1093/gigascience/gix018)
Supplement: Reviewer_2_Report_(Revision_1).pdf [file gix018_Reviewer_2_Report_(Revision_1).pdf]

Reviewer Report

Title: "Genome sequencing of the sweetpotato whitefly Bemisia tabaci MED/Q"

Version: Revision 1 **Date:** 12/19/2016

Reviewer name: Denis Tagu

Reviewer Comments to Author:

Dear authors and editors,

With the recent publication of a Bemisia tabacci genome paper (BMC Biology, 2016 14:110) by a concurrent group, we have now two Bemisia genomes, released by two different consortia. More complicated, some authors are signing both papers! This situation is not well appropriate for the community. To which reference genome will the community refer to? It is really a pity that the two consortia did not work together to share means and ideas instead of duplicating sequencing and analyses.

I am thus forced to consider the submitted paper as a Data Note, since it does not afford strong novelty compare to the other genome. That's the stupid and unfortunate result of competition, instead of collaboration.

And at least, the two consortia should now communicate in order to define a reference genome, and to help the community to find their way. I do consider i5k as a central point for proposing access to insect genomes and I clearly invite the authors to contact them once/if the paper is accepted.

Concerning the re-submission, I thank the authors for having evaluating my suggestions. The addition of RNAi on some genes potentially involved in insecticide resistance is a clear added-value.

CEGMA versus BUSCO: I still think BUSCO is better than SEGMA but it is not a critical issue. The authors can stand with CEGMA, even if I suggest giving both analyses (CEGMA and BUSCO) to help the readers to estimate the chance to get a full sequence of genes.

So my rejection concerns a publication in GigaScience as a Research Report, but is an acceptation for a Data Note.

Sorry for being so severe, but I feel really bad in front of these situations.

All the best

Denis

Methods

Are the methods appropriate to the aims of the study, are they well described, and are necessary controls included? Yes

Conclusions

Are the conclusions adequately supported by the data shown? Yes

Reporting Standards

Does the manuscript adhere to the journal's guidelines on minimum standards of reporting? Yes

Statistics

Are you able to assess all statistics in the manuscript, including the appropriateness of statistical tests used? Yes, and I have assessed the statistics in my report.

Quality of Written English

Please indicate the quality of language in the manuscript: Acceptable

Declaration of Competing Interests

Please complete a declaration of competing interests, considering the following questions:

- Have you in the past five years received reimbursements, fees, funding, or salary from an organisation that may in any way gain or lose financially from the publication of this manuscript, either now or in the future?
- Do you hold any stocks or shares in an organisation that may in any way gain or lose financially from the publication of this manuscript, either now or in the future?
- Do you hold or are you currently applying for any patents relating to the content of the manuscript?
- Have you received reimbursements, fees, funding, or salary from an organization that holds or has applied for patents relating to the content of the manuscript?
- Do you have any other financial competing interests?
- Do you have any non-financial competing interests in relation to this paper?

If you can answer no to all of the above, write 'I declare that I have no competing interests' below. If your reply is yes to any, please give details below.

I declare that I have no competing interests

I agree to the open peer review policy of the journal. I understand that my name will be included on my report to the authors and, if the manuscript is accepted for publication, my named report including any attachments I upload will be posted on the website along with the authors' responses. I agree for my report to be made available under an Open Access Creative Commons CC-BY license (http://creativecommons.org/licenses/by/4.0/). I understand that any comments which I do not wish to be included in my named report can be included as confidential comments to the editors, which will not be published.

I agree to the open peer review policy of the journal

To further support our reviewers, we have joined with Publons, where you can gain additional credit to further highlight your hard work (see: https://publons.com/journal/530/gigascience). On publication of this paper, your review will be automatically added to Publons, you can then choose whether or not to claim your Publons credit. I understand this statement.

Yes
