# Supplementary material for: Genome sequencing of the sweetpotato whitefly Bemisia tabaci MED/Q
Source: Gigascience. 2017 Mar 15;6(5):1–7. doi: 10.1093/gigascience/gix018 (PMC5467035; doi:10.1093/gigascience/gix018)
Supplement: Reviewer_2_Report_(revision_2).pdf [file gix018_Reviewer_2_Report_(revision_2).pdf]

Reviewer Report

Title: "Genome sequencing of the sweetpotato whitefly Bemisia tabaci MED/Q"

Version: Revision 2 **Date:** 2/22/2017

Reviewer name: Denis Tagu

Reviewer Comments to Author:

Dear authors,

I am OK with the very last version of your paper now as a note.

I still apologize that the genome is badly available. We can of course download the data, but biologists will need a database to browse, search for annotated genes and so on and so forth. And again, i5K@NAL provides very easily this service. It's up to you to follow or not this advice, but integratinfg in i5k database will give more visibility to your genome.

Table 1 indicates differences in the two genomes that are mainly due, to my opinion, to the different approaches used for sequencing, assembling and annotating the genomes.

Now that those two strains have been sequenced, this opens new tracks for further studies on population genomics and phylogeograpghy, which is great.

All the best

Level of Interest

Please indicate how interesting you found the manuscript: An article of limited interest

Quality of Written English

Please indicate the quality of language in the manuscript: Acceptable

Declaration of Competing Interests

Please complete a declaration of competing interests, considering the following questions:

- Have you in the past five years received reimbursements, fees, funding, or salary from an organisation that may in any way gain or lose financially from the publication of this manuscript, either now or in the future?
- Do you hold any stocks or shares in an organisation that may in any way gain or lose financially from the publication of this manuscript, either now or in the future?
- Do you hold or are you currently applying for any patents relating to the content of the manuscript?
- Have you received reimbursements, fees, funding, or salary from an organization that holds or has applied for patents relating to the content of the manuscript?
- Do you have any other financial competing interests?
- Do you have any non-financial competing interests in relation to this paper?

If you can answer no to all of the above, write 'I declare that I have no competing interests' below. If your reply is yes to any, please give details below.

I declare that I have no competing interest

I agree to the open peer review policy of the journal. I understand that my name will be included on my report to the authors and, if the manuscript is accepted for publication, my named report including any attachments I upload will be posted on the website along with the authors' responses. I agree for my report to be made available under an Open Access Creative Commons CC-BY license (http://creativecommons.org/licenses/by/4.0/). I understand that any comments which I do not wish to be included in my named report can be included as confidential comments to the editors, which will not be published.

I agree to the open peer review policy of the journal
